# WR279,396, a Third Generation Aminoglycoside Ointment for the Treatment of *Leishmania major* Cutaneous Leishmaniasis: A Phase 2, Randomized, Double Blind, Placebo Controlled Study

**DOI:** 10.1371/journal.pntd.0000432

**Published:** 2009-05-05

**Authors:** Afif Ben Salah, Pierre A. Buffet, Gloria Morizot, Nathalie Ben Massoud, Amor Zâatour, Nissaf Ben Alaya, Nabil Bel Haj Hamida, Zaher El Ahmadi, Matthew T. Downs, Philip L. Smith, Koussay Dellagi, Max Grögl

**Affiliations:** 1 Institut Pasteur de Tunis, Tunis, Tunisia; 2 Institut Pasteur, Paris, France; 3 Direction Régionale de la Santé de Sidi Bouzid, Sidi Bouzid, Tunisia; 4 Statistics Collaborative, Washington, D.C., United States of America; 5 United States Army Medical Materiel Development Activity, Fort Detrick, Maryland, United States of America; 6 Walter Reed Army Institute of Research, Silver Spring, Maryland, United States of America; London School of Hygiene and Tropical Medicine, United Kingdom

## Abstract

**Background:**

Cutaneous leishmaniasis (CL) is a disfiguring disease that confronts clinicians with a quandary: leave patients untreated or engage in a complex or toxic treatment. Topical treatment of CL offers a practical and safe option. Accordingly, the treatment of CL with WR279,396, a formulation of paromomycin and gentamicin in a hydrophilic base, was investigated in a phase 2 clinical study in Tunisia and France.

**Methods:**

A phase 2, randomized, double blind, vehicle-controlled study was conducted to assess the safety and efficacy of topical WR279,396 when applied twice a day for 20 days as treatment for parasitologically confirmed CL. The study protocol established the primary efficacy end point as complete clinical response (CCR) defined as 50% or greater reduction in the ulceration size of an index lesion by day 50 (D50) followed by complete re-epithelialization by D100, and no relapse through D180.

**Results:**

Ninety-two subjects were randomized. *Leishmania major* was identified in 66 of 68 isolates typed (97%). In the intent-to-treat population, 47 of 50 WR279,396 treated participants (94%) met the definition of CCR, compared with 30 of 42 vehicle-placebo participants (71%) [p = 0.0045]. Erythema occurred in 30% and 24% of participants receiving WR279,396 and placebo, respectively [p = 0.64]. There was no clinical or laboratory evidence of systemic toxicity.

**Conclusion:**

Application of WR279,396 for 20 days was found to be safe and effective in treating *L. major* CL, and offers great potential as a new, simple, easily applicable, and inexpensive topical therapy for this neglected disease.

**Trial Registration:**

ClinicalTrials.gov NCT00703924

## Author Summary

Cutaneous leishmaniasis is due to a small parasite (*Leishmania*) that creates disfiguring sores, and affects more than one million persons (mainly children) each year. Treating lesions with a cream—instead of with injections as currently done—would greatly improve the well-being of affected patients. No cream formulation that would be efficient and would not create important skin irritation has been identified yet. Here, we tested a new cream formulation (WR279,396) containing paromomycin and gentamicin, two members of a well-known family of antibacterial antibiotics (aminoglycosides). Injectable paromomycin is efficient in other forms of the disease (visceral leishmaniasis). This was a carefully monitored study (phase 2) involving mainly children in Tunisia and France. The cream was applied twice a day for 20 days. The proportion of patients treated with the paromomycin-containing cream (active formulation) that cured (94%) was higher than that observed (71%) in patients treated with a cream that did not contain the active product (placebo formulation). Local irritation affected less than one-third of the patients and was usually mild. This new cream formulation was safe and effective in treating cutaneous leishmaniasis, thereby providing a new, simple, easily applicable, and inexpensive treatment for this neglected disease.

## Introduction

The incidence of cutaneous leishmaniasis (CL) globally is 1.0–1.5 million cases annually [1]. There are several available therapeutic options, but none is optimal [2]. In Tunisia, the standard treatment is with intralesional injections of pentavalent antimonials [2] the recommended drugs used for the treatment of both visceral leishmaniasis and cutaneous leishmaniasis, first introduced 60 years ago.

Intralesional injections are painful, and they are difficult to administer to children, to patients with multiple lesions, or when lesions are located on the extremities [3],[4]. In such cases systemic antimony is often administered, despite its cost (US$50–200 per course), variable efficacy [2],[5], and potential or frank toxicity [2],[6].

Topical therapy of CL is an approach that is potentially efficient, practical and safe [7], yet a product fulfilling all those requirements has not yet been identified [8]. The aminoglycoside paromomycin is the most studied compound as a potential topical treatment for CL [9], and parenterally it is being aggressively pursued as a highly effective treatment for human visceral leishmaniasis [10]. As a class, aminoglycosides accumulate in lysosomes [11] where *Leishmania* multiply, and offer the potential to be highly effective therapeutics.

In topical preparations, paromomycin is a component of two antileishmanial products currently available outside the International Conference of Harmonization zone (ICH for USA, EC, and Japan). The first, developed by El-On [12] and marketed in Israel as Leshcutan, contains 15% paromomycin and 12% methyl-benzenthonium chloride (MBCL) in white soft paraffin. It has demonstrated good efficacy in treating CL [13],[14] but its usefulness is hampered by increased incidence of dermatologic irritation attributable to the MBCL [13],[14],[15],[16],[17]. The second formulation combines paromomycin (no MBCL) with urea and while non-irritating its efficacy remains largely undistinguished, with reported cure rates little better than placebo in Iran (47% v. 44%) and Tunisia (27% v. 18%) [18],[19],[20].

WR279,396 is a hydrophilic formulation of paromomycin 15% plus a second aminoglycoside (gentamicin 0.5%), that was developed in order to exploit the antileishmanial efficacy of the aminoglycosides while eliminating the potential for the skin irritation caused by MBCL. In this preparation, the addition of gentamicin has been shown to increase the antileishmanial efficacy of paromomycin in rodents [21]. In a Balb/c mouse model of CL, WR279,396 cured lesions caused by *L. major* (MON-4), *L. amazonensis*, *L. mexicana*, and *L. panamensis* strains in 100% of the mice without subsequent relapse [21]. These results were recently confirmed in a C57Bl/6 L. major MON-26 model [22]. In a pilot study in humans in the New World (*L. panamensis*), WR279,396 was well tolerated and shortened cure time, but had no effect on the overall cure rate at six months [23].

Herein, we report the results of a recently completed phase 2, randomized, double blind, vehicle-controlled trial in Tunisia and France to assess the efficacy and safety of topical WR279,396 administered twice a day for 20 days for the treatment of patients with CL caused by *L. major*.

## Methods

### Study Participants

Eligible participants were from the Sidi Bouzid governorate (Central Tunisia), where *L. major* MON-25 is endemic, and travelers returning to Paris from *L. major*-endemic areas in North and Sahelian Africa, who had skin lesions that were suspected to be CL. Criteria for inclusion were age between 5 and 75 years, the presence of parasitologically confirmed CL, lesions that were primarily ulcerative (i.e., not purely verrucous or nodular) and measured ≥1 cm^2^ and ≤5 cm^2^. Criteria for exclusion were history of known or suspected hypersensitivity or idiosyncratic reactions to aminoglycosides; previous use of antileishmanial drugs (within 3 months) or nephrotoxic or ototoxic drugs; prior diagnosis of leishmaniasis; more than 5 lesions, or a lesion in the face that in the opinion of the attending dermatologist could potentially cause significant disfigurement; significant medical problems as determined by history or laboratory studies; breast feeding and pregnancy. Participants also had to have normal Romberg tests and no relevant findings on baseline audiometry. In cases where the participant presented more than one lesion, investigators treated all lesions as per protocol with the same blinded study treatment as the index lesion.

### Study Design

The study was a phase 2, randomized, double blind, vehicle-controlled, multi-center trial. Participants were randomized in a 1∶1 allocation ratio to receive either WR279,396 or placebo-vehicle, each of which was applied twice daily for 20 days and covered with an occlusive dressing (Tegaderm, 3M Laboratory, Saint Paul, MN). Investigators, who were blinded to whether participants received WR279,396 or placebo-vehicle, evaluated lesions for clinical response on D20 (i.e., the end of the treatment period), D50 (i.e., 30 days after the conclusion of treatment), D100, and D180.

A sequence of genuine random numbers for the randomization procedure was obtained from the “fourmilab.ch/hotbits” website by a member of the Department of Chemical Information, Walter Reed Army Institute of Research, Silver Spring, Maryland and purged of duplicates. The random numbers are generated by a process which takes advantage of the inherent uncertainty in the quantum mechanical laws of nature. Specifically, they are generated by timing successive pairs of radioactive decays detected by a Geiger-Müller tube interfaced to a computer. This process is better than the pseudo-random number algorithms typically used in computer programs. The randomization of the study drugs was done by an independent group, Fischer BioServices, Rockville, Maryland a contractor to The U.S. Army Medical Research Acquisition Activity (USAMRAA), Ft. Detrick, Maryland.

### Endpoints

The study protocol established the primary efficacy end point as complete clinical response (CCR), defined as complete reepithelialization (i.e., length×width of ulceration = 0×0) of the index lesion by D50 or a >50% reepithelialization by D50 followed by complete reepithelialization on or before D100 with no relapse ever having occurred from D50 through D180. Relapse was defined as an increase in the area of ulceration relative to the previous measurement. Participants who did not complete the 180-day period of observation were considered to have failed to achieve CCR because relapse could not be fully assessed. The index lesion was defined as the uppermost, primarily ulcerative, parasitologically positive lesion on the body (excluding the ears) or, if two lesions were equally uppermost, the left uppermost primary ulcerative lesion. The secondary endpoint was the safety and tolerance of WR279,396.

### Procedures

The primary performing Institutions were the Institut Pasteur in Tunis, Tunisia, and the Medical Center Institut Pasteur, in Paris, France. Investigators measured all lesions in two perpendicular directions and took photographs at the following time points: prior to therapy, at the end of therapy (D20), and at 30 days (D50), 80 days (D100), and 6 months (D180) after the end of therapy. Medical personnel applied study drug (i.e., placebo-vehicle or WR279,396) twice daily for 20 days to all CL lesions present at baseline at a dose of 0.05 ml per 1 cm^2^ of CL lesion at a primary health facility in Tunisia and at the Medical Center of the Institut Pasteur in Paris. Each CL lesion was cleaned with soap and water and sterile 0.9% saline, and then dried using sterile USP Type VII Gauze sponges before application of study drug. Next, medical personnel dispensed study drug directly onto the ulcer from a pre-loaded 1 ml syringe without a needle, and spread drug over the ulcer using the finger of a disposable glove so as to penetrate even under the ulcer's borders. Study drug was to remain undisturbed (i.e., not wiped off and not wetted) for 4 hours after each application, so the adhesive polyurethane film dressing, Tegaderm, was applied over the top of the lesion following drug application. Investigators observed each participant for 30 minutes after application of study drug. Lesions and surrounding skin were evaluated for pain, erythema, and edema each day that the topical creams were administered and at follow-up study visits. The participants were also observed and questioned daily for the occurrence of systemic side effects (e.g., vertigo, tinnitus) using a standardized questionnaire. Diminished hearing was verified with the Danplex S42 audiometer (GN Otometrics, Maarkaervej 2A, DK-2630, Taastrup, Denmark). Clinical and laboratory evidence of side effects was determined on D10 and D20 by changes from baseline in serum creatinine, hearing, and Romberg tests. A Digmatic Caliper, Mitutoyo Corporation, model No. CD-6CS with a resolution of 0.01 mm and an accuracy of ±0.002 mm was used to measured lesions size. Lesions were measured by a trained investigator that followed a Study Specific Procedure (SSP-279396-01-003) in two perpendicular directions; in its greatest dimension, and at 90 degrees to the first measurement. Patients were not given incentives to come back for follow-up visits; patients were actively followed-up.

### Ethical and Regulatory Issues

Before entry into the study, investigators obtained written informed consent from all participants or, for pediatric participants, from parents/guardians. Comparison of WR279,396 to placebo-vehicle was justified for several reasons: CL caused by *L. major* is self-limiting and heals without treatment after several months. Furthermore, the trial allowed participants whose condition worsened to withdraw from the study and receive standard therapy. In addition vehicle application provided the following advantages: (i) protection against bacterial infection by keeping the lesion(s) clean and occluded; (ii) direct access to the medical team that performed the medical history, physical exam, dermatology exam, and laboratory test ; and (iii) complete parasitological diagnosis.

The study protocol (Principal Investigator Dr. Max Grögl), case report form, and SOPs were approved in the United States by the Walter Reed Army Institute of Research, Scientific Research Committee. A second level review of the protocol, consent form and all amendments was conducted by the Human Subjects Research Review Board (HSRRB), Commanding General, U.S. Army Medical Research and Materiel Command (USAMRMC), the Medical Ethical Committee of the Institut Pasteur de Tunis, Tunisia, and the Consultation Committee for the Protection of Individuals in Biomedical Research at Hospital Tarnier-Cochin, Paris, France. The study was conducted in accordance with Good Clinical Practice (GCP) under an Investigational New Drug (IND) application submitted to FDA. The Direction de la Pharmacie et des Médicaments, Ministère de la Santé Publique, Tunisia, and the Agence Française de Sécurité Sanitaire des Produits de Santé were informed of the trial. This study was conducted in accordance with ethical principles that have their origins in the Declaration of Helsinki and the Belmont Report. The Quality Assurance Office of the U.S. Army Medical Materiel Development Activity monitored the study.

### Drugs

WR279,396 is an off-white to yellowish, thick cream containing 15% (w/w) paromomycin-sulfate (Farmitalia) and 0.5% (w/w) gentamicin-sulfate (Schering) as active components. Study drugs were manufactured by the University of Iowa, College of Pharmacy under Good Manufacturing Practice (GMPs). The placebo consisted of the vehicle without the active components and trace amounts of coloring agents to match the appearance and maintain the blind.

### Parasitologic Studies

Each lesion to be evaluated for efficacy was aspirated and/or scraped and/or biopsied. Proof of infection was documented through either the demonstration of motile promastigotes in aspirate cultures or the microscopic identification of *Leishmania* amastigotes in material obtained from CL lesions. Iso-enzyme [24] and/or PCR [25] analysis of the parasites isolated from the CL lesions was completed after study treatment had been started. Iso-enzyme and PCR analyses were carried out according to published protocols [24],[25].

### Statistical Analysis

The protocol calculated a sample size of 50 participants per group with 80 percent power and a Type I error rate of 5 percent to detect a 30 percent difference in the proportion of participants achieving CCR, assuming a CCR proportion of 35 percent in the placebo-vehicle group and 65 percent in WR279,396 participants, with a 5% expected rate of loss to follow-up.

Analyses included all randomized participants under the intention-to-treat principle and the randomization was coordinated between the two clinical sites. Continuous data were compared using the Wilcoxon rank-sum test and categorical data were compared using the Fisher's exact test. StatXact version 7 (Cytel Software Corporation, Cambridge, MA) was used to calculate 95% exact confidence intervals (CIs) of the difference in the proportion achieving CCR with the option to compute a CI on the difference of two binomial proportions based on the standardized statistic and inverting two one-sided tests. A log-rank test was used to compare time to reepithelialization without relapse. Because the trial collected data on clinical response only at discrete time-points, namely the D20, D50, D100, and D180 visits, the time-to-event analysis grouped reepithelialization times according to the visit at which investigators observed the event. To adjust for baseline differences, a linear model for the proportion of participants achieving CCR was fit for each baseline variable of interest with covariates for treatment group and the baseline variable. To examine whether the effect of WR279,396 varied between subgroups, we calculated the Breslow-Day test for homogeneity of the odds ratio

## Results

**Figure 1:**
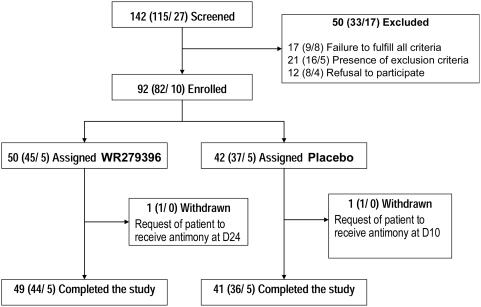
Flowchart summarizing the enrolment, randomization and follow-up of patients. Values are numbers of patients as follows: Total number of patients (Number at Tunisian site/number at French site).

Between March 2003 and January 2005, 142 participants (27 in Paris and 115 in Sidi Bouzid, Tunisia) were screened, of whom 92 (10 in Paris and 82 in Sidi Bouzid, Tunisia) underwent randomization; 50 were assigned to the WR279,396 group and 42 to the placebo-vehicle control group (Figure 1). The study was conducted over at least one entire leishmaniasis season. Figure 1 presents the distribution of participants from screening until study completion in the two treatment groups for both sites. Forty-nine of 50 participants randomized to WR279,396 and 41 of 42 participants randomized to placebo-vehicle completed the study. All participants had lesions that were parasitologically confirmed by smear, culture or both. Iso-enzyme testing of 18 isolates (8 isolates from the Paris site and 10 from Tunisia) identified *L. major* in 17 participants and *L. infantum* in one participant, who was from the French site. L. major isolates from the Paris site were MON-74, MON-26, MON-25, -, and all *L. major* isolates from Tunisia were MON-25. Fifty isolates from Tunisia were tested using PCR, which identified L. major in 49 participants and *L. tropica* in one participant. In total, *L. major* was identified in 66 of 68 isolates typed (97%). With one exception, applications of study drugs were conducted according to the protocol. In this one case, treatment was stopped after only 12 applications (6 days) due to skin irritation and conjunctivitis that resulted from inadvertent contact of study drug to the eye while sleeping. However, this participant's lesion rapidly improved without any subsequent therapy allowing follow-up evaluations to be conducted as per protocol. All 92 participants received study drug (either placebo-vehicle or WR279,396). Except for 2 participants, who withdrew voluntarily from the study during or following the 20-day treatment period to receive alternative therapy (Figure 1), no participant was lost to follow-up, and all major end-points were accessible for all.

Overall, the two treatment groups were similar in baseline demographics and disease characteristics (Table 1). A greater proportion of placebo-vehicle participants were 18 or older compared to WR279,396 participants. Because participants at the Medical Center Institut Pasteur in Paris contracted CL while traveling, a greater proportion of participants were 18 years or older compared to Tunisian participants. Fifty-four participants had a single lesion at baseline. The distributions of lesion area, both of the index lesion and of all baseline lesions, were roughly equivalent between the groups. Forty percent of participants in each treatment group had the index lesion above the belt, and all but two WR279,396 participants had the index lesion on the limbs. The median number of days before treatment since participants first noticed a baseline CL lesion/papule was 62 in both groups.

**Table 1:** Baseline Characteristics of the Participants.

	WR279,396 (N = 50)	Placebo-Vehicle (N = 42)	P Value
Center – no. (%)			
Paris, France	5 (10)	5 (12)	1.0
Sidi Bouzid, Tunisia	45 (90)	37 (88)	
Male sex – no. (%)	27 (54)	27 (64)	0.40
Age <18 years – no. (%)			
Overall	47 (94)	33 (79)	0.034
Paris, France (N = 10)	2 (40)	0 (0)	
Sidi Bouzid, Tunisia (N = 82)	45 (100)	33 (89)	
Lesions – no. (%)			
1	28 (56)	26 (62)	0.67†
2	9 (18)	7 (17)	
3	9 (18)	4 (10)	
4 or 5	4 (8)	5 (12)	
Total lesion area – mm^2^			
Median	128	154	0.52
Interquartile range	85 to 223	70 to 264	
Index lesion area – mm^2^			
Median	92	115	0.34
Interquartile range	55 to 141	50 to 172	
Index lesion on upper body – no. (%)	20 (40)	17 (40)	1.0
Index lesion on extremity – no. (%)	48 (96)	42 (100)	0.50
Days before treatment since lesion first noticed			
Median	62	62	0.96
Interquartile range	38 to 79	39 to 79	
*Leishmania* species – no. (%)			
*L. major*	32* (64)	24* (57)	0.53‡
*L. infantum*	1 (2)	0 (0)	
*L. tropica*	1 (2)	0 (0)	
Unidentified	16 (32)	18 (43)	

Comparisons of categorical variables use the Fisher's exact test for the entire study cohort, while comparisons of continuous variables use the Wilcoxon rank-sum test.

**†:** Compares the proportion of participants in each group with a sole lesion at baseline.

*: Species identification was by isoenzyme electrophoresis (18 isolates), PCR (50 isolates) or both techniques (8 isolates). All isolates identified by both techniques were from Tunisia and all belonged to the *L. major* MON-25 zymodem (the only *L. major* zymodem reported from the Maghreb). In France, identification was by isoenzyme electrophoresis in 8 cases as follows: *L. major* MON-25 (5 isolates), *L. infantum* MON-24, *L. major* MON-26, *L. major* MON-74 (1 isolate each).

**‡:** Compares the proportion of participants in each group with *L. major* identified.

### Efficacy Analysis

#### Primary analysis, complete clinical response (CCR) - index lesion

Among the 50 WR279,396-treated participants, 47 (94%) met the definition of CCR, compared with 30 of the 42 placebo-vehicle participants (71%) (p = 0.0045), resulting in an estimated difference of 23% in favor of WR279,396 participants (95% exact CI: 6, 39). The reasons for failure in the 3 participants treated with WR279,396 were: (i) two participants had less than 50% reepithelialization at D50 (although one participant completely healed 1 week later without further therapy), (ii) one participant was considered a treatment failure in the intent-to-treat analysis because he requested to be withdrawn and switched to intralesional antimony. The single WR279,396-treated participant who completed only 6 days of treatment cured despite such a short course. Among the 12 placebo-vehicle participants who failed, in 8 participants the ulcer either increased in size or decreased by less than 50% by D50, 3 participants relapsed, and 1 who withdrew voluntarily from the study was lost to follow-up and considered a failure in the intent-to-treat analysis.

#### Complete reepithelialization of index lesion without relapse

**Figure 2:**
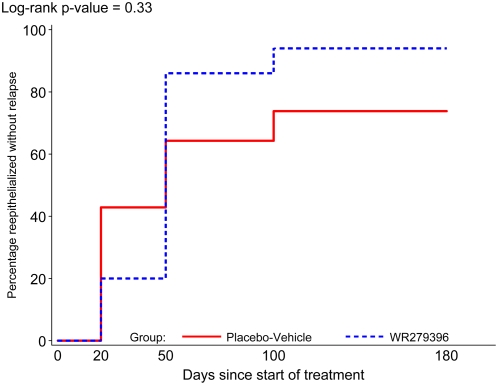
Reepithelialization of index lesion without relapse.

Figure 2 shows the time course of complete reepithelialization (i.e, 0×0 ulceration) without relapse of the index lesion in each group at D20, D50, D100, and D180. At D20 (i.e., last day of topical application), the proportion of placebo-vehicle participants achieving complete reepithelialization without subsequent relapse was initially higher than for WR279,396 participants (43 versus 20 percent). By D50, however, 86 percent of WR279,396-treated and 64 percent of placebo-vehicle participants had complete reepithelialization of the index lesion without subsequent relapse. The crossing of the time-to-event curves between D20 and D50 is reflected in the log-rank test, which failed to detect a difference in the distribution of time to reepithelialization (p = 0.33).

#### Relapses

Clinical relapse of the index lesion following completion of the 20-day course of treatment was observed in 3 participants, all from the placebo-vehicle group. No clinical relapse of the index lesion or non-index lesion occurred in the WR279,396 group.

#### Analysis by participant incorporating all treated lesions

Rather than restrict the analysis to the index lesion, we compared CCR by treatment group for all treated lesions, i.e., patient cure. Response status for the 28 (56%) WR279,396 participants and 26 (62%) placebo-vehicle participants whose index lesion was the only treated lesion are identical to that of the primary analysis. For the 38 participants who investigators treated at multiple lesions, participants are classified as having CCR only if all lesions met the definition of CCR. When considering all treated lesions, results were similar to the categorization for index lesions (Table 2).

**Table 2:** CCR at the index lesion and all lesions.

	WR279,396 (N = 50)	Placebo (N = 42)	Total (N = 92)	Fisher's exact p	Difference in % (95% CI)
Index lesion					
CCR	47 (94%)	30 (71%)	77 (84%)	0.0045	23% (6%, 39%)
No CCR	3 (6%)	12 ( 29%)	15 (16%)		
All lesions					
CCR	46 (92%)	29 (69%)	75 (82%)	0.0065	23% (6%, 40%)
No CCR	4 (8%)	13 (31%)	17 (18%)		

In summary, regardless of the criterion used to analyze the data, the proportion of lesions and participants with a positive outcome by D50 was significantly greater in the WR279,396-treated group than in the placebo-vehicle group.

### Evolution in non *L. major* Cases

Two participants were confirmed to be infected with a non-*L. major* species. One was infected with *L. infantum* and the other with *L. tropica*. Self-healing occurs less frequently with both species than with *L. major*. Both participants were in the WR279,396 group and achieved CCR, although the participant infected with *L. infantum* received only 6 days of treatment.

### Influence of Baseline Factors on Effect of Treatment

We explored CCR in subgroups defined by baseline characteristics including number (1 and >1), index lesion area (<100 and ≥100 mm^2^), location (upper and lower body), and age (<60 and ≥60 days) of lesions. After adjustment singly for each baseline factor, the statistical benefit of WR279,396 over placebo-vehicle remained (Table 3). Adjustment for age less than 18 years, however, noticeably lessened the estimate of the treatment effect, with an adjusted difference in the proportion of CCR of 16 percent (95% CI: 1, 30). The higher proportion of participants under 18 years achieving CCR combined with the greater proportion of WR279,396 participants less than 18 was responsible for this. When modeling lesion number, index lesion area, and lesion age continuously, only adjustment for index lesion area differed appreciably from the model where area was modeled categorically. Adjusting for index lesion area as a continuous covariate resulted in an estimated difference in the proportion of CCR of 19 percent (95% CI: 4, 34).

**Table 3:** Complete Clinical Response (CCR) at the Index Lesion According to Baseline Characteristics.

Subgroup	No. of Participants	% CCR		Difference in %	
		WR279,396	Placebo	Observed (95% CI)	Adjusted (95% CI)
Center					22 (7, 37)
Paris, France	10	80	40	40 (−15, 95)	
Sidi Bouzid, Tunisia	82	96	76	20 (5, 35)	
Male sex					22 (7, 37)
Yes	54	93	67	26 (6, 46)	
No	38	96	80	16 (−6, 38)	
Age <18 years					16 (1, 30)
Yes	80	96	82	14 (0, 28)	
No	12	67	33	33 (−28, 95)	
Single baseline lesion					24 (10, 38)
Yes	54	96	88	8 (−6, 22)	
No	38	91	44	47 (20, 74)	
Index lesion <100 mm^2^					22 (6, 37)
Yes	46	96	78	19 (−2, 39)	
No	46	91	67	24 (2, 47)	
Index lesion on upper body					23 (7, 38)
Yes	37	100	71	29 (8, 51)	
No	55	90	72	18 (−3, 39)	
Lesion noticed <60 days before treatment					23 (7, 38)
Yes	43	96	70	26 (4, 47)	
No	49	93	73	20 (−1, 41)	

No baseline factor appeared to modify the effect of WR279,396, as indicated by Breslow-Day homogeneity tests, all of which were above 0.20.

### Safety

Topical administration of WR279,396 was generally safe and well tolerated (Table 4). No death occurred during this clinical trial, and the only serious adverse event was an arm fracture unrelated to study medication. Overall, the number of participants experiencing adverse events was comparable, with roughly a quarter of participants in each group experiencing an adverse event. The most commonly reported event was erythema at the site of application, which occurred in 30 percent of participants who received WR279,396 and 24 percent of participants who received placebo-vehicle with onset within 30 minutes of application (p = 0.64). Mild pain within 30 minutes of application was reported in roughly 14 percent of participants in each group. No participant had an increase from baseline serum creatinine following administration of study drug (D10 and D20). Only mild increases and decreases in hearing acuity from baseline were detected on audiometry, occurring with similar frequency in both groups (28% and 21% in WR279,396 and placebo-vehicle, respectively; p = 0.63). There was no report of vertigo and no abnormal Romberg test result in participants who received WR279,396.

**Table 4:** Immediate & Delayed Local & Systemic Toxicity.

REACTION	WR279,396 Group (N = 50)		Placebo-Vehicle Group (N = 42)	
	Participant with reaction – no (%)	Mean duration (days)	Participant with reaction – no (%)	Mean duration (days)
IMMEDIATE^0^				
LOCAL PAIN^1^	7 (14.0)	3.1	6 (14.2)	3.3
Mild	7 (14.0)	3.1	6 (14.2)	3.3
Moderate	0 (0.0)	0	0 (0.0)	0
Severe	0 (0.0)	0	0 (0.0)	0
LOCAL ERYTHEMA^2^	15 (30.0)	7.7	10 (23.8)	8.8
Mild	15 (30.0)	6.4	10 (23.8)	7.7
Moderate	5 (10.0)	3.4	2 (4.8)	4.5
Severe	1 (2.0)	2.0	0 (0.0)	0
LOCAL EDEMA^2^	1 (2.0)	10.0	2 (4.8)	5.0
Mild	1 (2.0)	6.0	2 (4.8)	5.0
Moderate	1 (2.0)	2.0	0 (0.0)	0
Severe	1 (2.0)	2.0	0 (0.0)	0
SYSTEMIC REACTION	1 (2.0)	1.0	1 (2.4)	2.0
Vertigo	0 (0.0)	0	1 (2.4)	2.0
Tinnitus	1 (2.0)	1.0	0 (0.0)	0
Hearing	0 (0.0)	0	0 (0.0)	0
DELAYED^0^				
LOCAL PAIN	9 (18.0)	1.6	5 (11.9)	3.2
Mild	9 (18.0)	1.6	5 (11.9)	3.2
Moderate	0 (0.0)	0	0 (0.0)	0
Severe	0 (0.0)	0	0 (0.0)	0
LOCAL ERYTHEMA	15 (30.0)	7.8	11 (26.2)	8.0
Mild	15 (30.0)	6.5	10 (23.8)	7.8
Moderate	4 (8.0)	4.3	3 (7.1)	3.3
Severe	1 (2.0)	2.0	0 (0.0)	0
LOCAL EDEMA	1 (2.0)	11.0	3 (7.1)	3.7
Mild	1 (2.0)	7.0	3 (7.1)	3.7
Moderate	1 (2.0)	2.0	0 (0.0)	0
Severe	1 (2.0)	2.0	0 (0.0)	0
SYSTEMIC REACTION	0 (0.0)	0	2 (4.8)	1.5
Vertigo	0 (0.0)	0	2 (4.8)	1.5
Tinnitus	0 (0.0)	0	0 (0.0)	0
Hearing	0 (0.0)	0	0 (0.0)	0

0 Immediate: observed within 30 minutes of application Delayed : Observed just prior to next application.

1 Mild pain: does not interfere with daily activity, Moderate pain: interferes with daily activity, Severe pain: daily activities are interrupted.

2 Mild: barely perceptible erythema or edema, Moderate: well defined erythema or edema, Severe: very red erythema with raised >2 mm edema.

## Discussion

For a neglected disease like leishmaniasis, the development of a GMP formulation that is safe and efficacious is a step in the right direction. There is a general lack of safe, effective, and affordable pharmaceuticals worldwide to treat or prevent neglected diseases that disproportionately cause high mortality and morbidity among the world's poor in the developing world [26]. Of the many examples of neglected diseases, *L. major* CL is perhaps one disease that should have numerous good solutions by now, yet the internationally accepted standard treatment remains largely tied to antimony, even for all the problems associated with its use.

For decades, clinicians caring for patients with CL have been confronted with a difficult choice: either leave patients untreated (a common proposal for patients with five or fewer uncomplicated lesions due to *L. major*), or engage in a complex or toxic treatment for this disfiguring, but non life-threatening disease. In this study, we found that WR279,396 was well-tolerated and induced complete clinical response (CCR) in a significantly greater percentage of participants compared to placebo-vehicle in participants with CL due to *L. major*. These results raise a strong possibility that we may exit from this old quandary of how best to manage patients with *L. major* CL. Of the 49 participants treated in the WR279,396 arm, only 1 (2%) failed to achieve complete reepithelialization of his lesion in less than 2 months. If the efficacy of WR279,396 can be reproduced in subsequent phase 3 clinical studies, complex therapeutic decisions in *L. major* CL may become the exception rather than the rule.

The time-to-event analysis raised two interesting observations: First, the response seen with placebo-vehicle was markedly higher than the response reported in placebo-treated participants in a previous paromomycin-urea trial performed at the same site in 1995 (71% versus 32%) [19]. Thus, an intrinsic efficacy of the vehicle of WR279,396 on CL ulcerations may account for part of this difference, and for the unexpectedly high placebo cure rate in the trial reported here. Second, and consistent with our earlier studies evaluating WR279,396, during the 20-day drug application period (between D1 and D20), the mean ulceration area for WR279,396-treated participants decreased at a slower rate than in placebo-vehicle treated participants (Figure 2). This initial transient slowing in ulceration closure was not totally unexpected. The natural progression of the healing process in CL entails a decrease in the depth of the ulceration as the parasite load decreases followed by a reduction in the ulceration width as re-epithelialization progresses. Thus, in treated participants, the non-improvement at day 20 of the mean ulceration area may be linked to the inflammatory response as parasites are killed by WR279,396. This slower decrease of ulceration area, limited to the 20-day drug application phase followed by a significant acceleration in healing after D20, bore no negative clinical impact. Indeed, only 1 participant in each group requested to be withdrawn before the major end-point evaluation at D50. Finally, and perhaps paradoxically, in most patients, the fact that reepithelialization started after the end of the 20-day application period may actually favor compliance with this treatment schedule should the drug become widely available.

WR279,396 continued to demonstrate an excellent safety profile with very few local and no systemic adverse events observed. This trend was similar to our previous experience with this product compiled from pre-clinical, Phase 1, and two earlier phase 2 studies in the New World [23]. Importantly, this topical preparation containing two aminoglycosides displayed no detectable renal or VIIIth cranial nerve toxicity. These safety observations are in accord with the findings from a recent study of intramuscular (IM) paromomycin for visceral leishmaniasis in India, which also showed no clinically significant kidney or VIIIth nerve toxicity yet the systemic exposure in that IM study was much greater than from WR279,396 applied topically [27].

In addition to the promising efficacy observed in this study against *L. major* MON-25, data collected thus far indicate that WR279,396 will likely be broadly effective against a wider variety of leishmania species. Several key observations support such optimism. First WR279,396 was efficient not only in *L. major* MON-26 [22] , and in L. major MON-4, but also in *L. amazonensis*, *L. mexicana*, and *L. panamensis* in infected mice [21]. Second WR279,396 was active in *L. panamensis* in humans in Colombia [23]. Third, *L. tropica* is very sensitive to paromomycin in vitro [28]. And fourth, but not least, in an *L. tropica* focus of Turkey, the paromomycin+MBCL formulation induced a 37.5% cure rate at 4 weeks, suboptimal, but significantly higher than the 0% cure rate in oral ketoconazole-treated patients [29],[30].

### Conclusion

The application of WR279,396 for 20 days was found to be safe and effective (94% cure rate) in treating *L. major* CL, and offers great potential as a new, simple, easily applicable, and inexpensive topical therapy for this neglected disease [31]. *L. major* CL in North Africa, Sahelian Africa, and the Middle East, involves tens to hundreds of thousands of people each year, many of whom are children [32]. Health systems are often unable to cope with these epidemics. In this context, a simple, straightforward treatment is crucial.

## Supporting Information

Checklist S1 CONSORT Checklist
(0.06 MB DOC)

Protocol S1 (6.44 MB PDF)
