# Expression of Concern: WR279,396, a third generation aminoglycoside ointment for the treatment of *Leishmania major* cutaneous leishmaniasis: A phase 2, randomized, double blind, placebo controlled study

**DOI:** 10.1371/journal.pntd.0014047

**Published:** 2026-03-05

**Authors:** The PLOS Neglected Tropical Diseases Editors

Following the publication of this article [1], concerns were raised about the study's compliance with a Tunisian decree (n° 90-1401, chapter I Article 2) on the use of minors in clinical research.

The corresponding author provided the following statement on this matter:

“The authors state that the concerns above are incorrect, as the study was designed to include children as requested by the Tunisian Ministry of Health and approved by Tunisian ethics committees and review bodies. The study was conducted in full compliance with Tunisian and US ethical and legal standards for research with children.”

PLOS notes that the article [1] reports IRB approval from the Medical Ethical Committee of the Institut Pasteur de Tunis, the Human Subjects Research Review Board of the U.S. Army Medical Research and Materiel Command, and the Consultation Committee for the Protection of Individuals in Biomedical Research at Hospital Tarnier-Cochin, Paris, France. The authors provided documents pertaining to approval from the Tunisian Ministry of Health, but these documents did not fully clarify this matter and PLOS has been unable to confirm whether the approvals obtained met all local requirements. Therefore, the *PLOS Neglected Tropical Diseases* Editors issue this Expression of Concern.
