# Supplementary material for: WR279,396, a Third Generation Aminoglycoside Ointment for the Treatment of Leishmania major Cutaneous Leishmaniasis: A Phase 2, Randomized, Double Blind, Placebo Controlled Study
Source: PLoS Negl Trop Dis. 2009 May 5;3(5):e432. doi: 10.1371/journal.pntd.0000432 (PMC2673687; doi:10.1371/journal.pntd.0000432)
Supplement: Protocol S1 — (6.44 MB PDF) [file pntd.0000432.s002.pdf]

DEPARTMENT OF THE ARMY
US ARMY MEDICAL RESEARCH AND MATERIEL COMMAND
504 SCOTT STREET
FORT DETRICK, MARYLAND 21702-5012

January 15, 2003

REPLY TO
ATTENTION OF

Office of Regulatory
Compliance and Quality

SUBJECT: IND 50098 - WR 279,396 Paromomycin Sulfate + Gentamicin Sulfate in Aquaphilic (Serial No. 019)

Food and Drug Administration
Center for Drug Evaluation and Research
Office of Drug Evaluation IV
Division of Anti-Infective Drug Products
9200 Corporate Boulevard (HFD-520)
Rockville, Maryland 20850

Dear Sir or Madam:

Enclosed in triplicate is a new clinical protocol entitled, "Topical Treatment of Cutaneous Leishmaniasis With WR 279396: A Phase 2 Study in the Old World" (HSRRB Log No. A-9768) that will be conducted by Lieutenant Colonel Max Grogl at Walter Reed Army Institute of Research, under the subject IND. This protocol was reviewed by the Human Subjects Research Review Board on January 9, 2002 and was approved for im[illegible]nentation on January 7, 2003.

Copies have been furnished to U.S. Army Medical Research and Materiel Command, Secretary of the General Staff and U.S. Army Medical Materiel Development Activity, Pharmaceutical Systems Project Manager.

The point of contact for regulatory questions concerning this submission is Ms. Kathie Mantine at 301-619-2809, facsimile 301-619-7803, or electronic mail kathie.mantine@det.amedd.army.mil. The U.S. Army Medical Materiel Development Activity point of contact for technical questions is Major Erich Lehnert at 301-619-2053.

Sincerely,

Kut

for

Isiah M. Harper, Jr.
Lieutenant Colonel, Medical Service Corps
Chief, Regulatory Affairs

Enclosure

DEPARTMENT OF HEALTH AND HUMAN SERVICES
PUBLIC HEALTH SERVICE
FOOD AND DRUG ADMINISTRATION

INVESTIGATIONAL NEW DRUG APPLICATION (IND)

(TITLE 21, CODE OF FEDERAL REGULATIONS (CFR) PART 312)

Form Approved: OMB No. 0910-0014.
Expiration Date: November 30, 2002
See OMB Statement on Reverse.

NOTE: No drug may be shipped or clinical investigation begun until an IND for that investigation is in effect (21 CFR 312.40).

1. NAME OF SPONSER
Office of The Surgeon General, Department of the Army

2. DATE OF SUBMISSION
15 Jan 03

3. ADDRESS *(Number, Street, City, State and Zip Code)*
Commanding General, U.S. Army Medical Research and Materiel Command
ATTN: MCMR-RCQ-RA
504 Scott Street
Fort Detrick, MD 21702-5012

4. TELEPHONE NUMBER *(Include Area Code)*
301-619-2165

5. NAME(S) OF DRUG *(Include all available names: Trade, Generic, Chemical, Code)*
WR 279,396 Paromomycin Sulfate + Gentamicin Sulfate in Aquaphilic

6. IND NUMBER *(If previously assigned)*
IND 50098

7. INDICATION(S) *(Covered by this submission)*
Cutaneous leishmaniasis

8. PHASE(S) OF CLINICAL INVESTIGATION TO BE CONDUCTED:
☐ PHASE 1 ☒ PHASE 2 ☐ PHASE 3 ☐ OTHER ________ *(Specify)*

9. LIST NUMBERS OF ALL INVESTIGATIONAL NEW DRUG APPLICATIONS *(21 CFR Part 312)*, NEW DRUG OR ANTIBIOTIC APPLICATIONS *(21 CFR Part 314)*, DRUG MASTER FILES *(21 CFR Part 314.420)*, AND PRODUCT LICENSE APPLICATIONS *(21 CFR Part 601)* REFERRED TO IN THIS APPLICATION.

10. ***IND submission should be consecutively numbered. The initial IND should be numbered "Serial number: 000." The next submission (e.g., amendment, report, or correspondence) should be numbered "Serial Number: 001." Subsequent submissions should be numbered consecutively in the order in which they are submitted.***

SERIAL NUMBER
0 1 9

11. THIS SUBMISSION CONTAINS THE FOLLOWING: ***(Check all that apply)***
☐ INITIAL INVESTIGATIONAL NEW DRUG APPLICATION (IND)
☐ RESPONSE TO CLINICAL HOLD

PROTOCOL AMENDMENT(S):
☒ NEW PROTOCOL
☐ CHANGE IN PROTOCOL
☐ NEW INVESTIGATOR

INFORMATION AMENDMENT(S):
☐ CHEMISTRY/MICROBIOLOGY
☐ PHARMACOLOGY/TOXICOLOGY
☐ CLINICAL

IND SAFETY REPORT(S):
☐ INITIAL WRITTEN REPORT
☐ FOLLOW-UP TO A WRITTEN REPORT

☐ RESPONSE TO FDA REQUEST FOR INFORMATION
☐ ANNUAL REPORT
☐ GENERAL CORRESPONDENCE
☐ REQUEST FOR REINSTATEMENT OF IND THAT IS WITHDRAWN, INACTIVATED, TERMINATED OR DISCONTINUED
☐ OTHER ________ *(Specify)*

CHECK ONLY IF APPLICABLE

JUSTIFICATION STATEMENT MUST BE SUBMITTED WITH APPLICATION FOR ANY CHECKED BELOW. REFER TO THE CITED CFR SECTION FOR FURTHER INFORMATION.

☐ **TREATMENT IND 21 CFR 312.35(b)** ☐ **TREATMENT PROTOCOL 21 CFR 312.35(a)** ☐ **CHARGE REQUEST/NOTIFICATION 21 CFR312.7(d)**

FOR FDA USE ONLY

CDR/DBIND/DGD RECEIPT STAMP	DDR RECEIPT STAMP	DIVISION ASSIGNMENT:
		IND NUMBER ASSIGNED:

PSC Media Arts Branch (301) 443-1090 EF

12. **CONTENTS OF APPLICATION**

This application contains the following items: *(Check all that apply)*

- ☐ 1. Form FDA 1571 *[21 CFR 312.23(a)(1)]*
- ☐ 2. Table of Contents *[21 CFR 312.23(a)(2)]*
- ☐ 3. Introductory statement *[21 CFR 312.23(a)(3)]*
- ☐ 4. General Investigational plan *[21 CFR 312.23(a)(3)]*
- ☐ 5. Investigator's brochure *[21 CFR 312.23(a)(5)]*
- ☐ 6. Protocol(s) *[21 CFR 312.23(a)(6)]*
  - ☐ a. Study protocol(s) *[21 CFR 312.23(a)(6)]*
  - ☐ b. Investigator data *[21 CFR 312.23(a)(6)(iii)(b)]* or completed Form(s) FDA 1572
  - ☐ c. Facilities data *[21 CFR 312.23(a)(6)(iii)(b)]* or completed Form(s) FDA 1572
  - ☐ d. Institutional Review Board data *[21 CFR 312.23(a)(6)(iii)(b)]* or completed Form(s) FDA 1572
- ☐ 7. Chemistry, manufacturing, and control data *[21 CFR 312.23(a)(7)]*
  - ☐ Environmental assessment or claim for exclusion *[21 CFR 312.23(a)(7)(iv)(e)]*
- ☐ 8. Pharmacology and toxicology data *[21 CFR 312.23(a)(8)]*
- ☐ 9. Previous human experience *[21 CFR 312.23(a)(9)]*
- ☐ 10. Additional information *[21 CFR 312.23(a)(10)]*

13. IS ANY PART OF THE CLINICAL STUDY TO BE CONDUCTED BY A CONTRACT RESEARCH ORGANIZATION? ☐ YES ☒ NO

IF YES, WILL ANY SPONSOR OBLIGATIONS BE TRANSFERRED TO THE CONTRACT RESEARCH ORGANIZATION? ☐ YES ☐ NO

IF YES, ATTACH A STATEMENT CONTAINING THE NAME AND ADDRESS OF THE CONTRACT RESEARCH ORGANIZATION, IDENTIFICATION OF THE CLINICAL STUDY, AND A LISTING OF THE OBLIGATIONS TRANSFERRED.

14. NAME AND TITLE OF THE PERSON RESPONSIBLE FOR MONITORING THE CONDUCT AND PROGRESS OF THE CLINICAL INVESTIGATIONS

MAJ Erich K. Lehnert
Product Manager, Pharmaceutical Systems Division
U.S. Army Medical Materiel Development Activity

15. NAME(S) AND TITLE(S) OF THE PERSON(S) RESPONSIBLE FOR REVIEW AND EVALUATION OF INFORMATION RELEVANT TO THE SAFETY OF THE DRUG

LTC Max Grogl
Division of Communicable Diseases and Immunology
Walter Reed Army Institute of Research

I agree not to begin clinical investigations until 30 days after FDA's receipt of the IND unless I receive earlier notification by FDA that the studies may begin. I also agree not to begin or continue clinical investigations covered by the IND if those studies are placed on clinical hold. I agree that an Institutional Review Board (IRB) that complies with the requirements set fourth in 21 CFR Part 56 will be responsible for initial and continuing review and approval of each of the studies in the proposed clinical investigation. I agree to conduct the investigation in accordance with all other applicable regulatory requirements.

16. NAME OF SPONSOR OR SPONSOR'S AUTHORIZED REPRESENTATIVE	17. SIGNATURE OF SPONSOR OR SPONSOR'S AUTHORIZED REPRESENTATIVE	
JEFFREY A. GERE, COL, MS<br>Commanding, U.S. Army Medical Materiel Development Activity	[signature]	
18. ADDRESS *(Number, Street, City, State and Zip Code)*	19. TELEPHONE NUMBER *(Include Area Code)*	20. DATE
Commanding General, USAMRMC<br>504 Scott Street<br>Fort Detrick, MD 21702-5012	301-619-7643	14 Apr 03

(WARNING: A willfully false statement is a criminal offense. U.S.C. Title 18, Sec. 1001.)

Public reporting burden for this collection of information is estimated to average 100 hours per response, including the time for reviewing instructions, searching existing data sources, gathering and maintaining the data needed, and completing reviewing the collection of information. Send comments regarding this burden estimate or any other aspect of this collection of information, including suggestions for reducing this burden to:

Food and Drug Administration
CBER (HFM-99)
1401 Rockville Pike
Rockville, MD 20852-1448

Food and Drug Administration
CDER (HFD-94)
12229 Wilkins Avenue
Rockville, MD 20852

"An agency may not conduct or sponsor, and a person is not required to respond to, a collection of information unless it displays a currently valid OMB control number."

Please **DO NOT RETURN** this application to this address.

PSC Media Arts Branch (301) 443-1090 EF

Table of Contents

IRB approval Letters.	Enclosure 1
Protocol.	Enclosure 2
Appendix A: Informed Consent Documents, 7 January 2003 Approved versions and Volunteer Time and Event Schedule.	Enclosure 3
Appendix B: Volunteer Donation Form.	Enclosure 4
Appendix C: Certification of Translation.	Enclosure 5
Appendix 1: FDA Form 1572.	Enclosure 6
Appendix 2: Curriculum Vitae of Principal Investigator.	Enclosure 7
Appendix 3: Adverse Event Notification Form.	Enclosure 8
Appendix 4: Case Report Form.	Enclosure 9
Appendix 5: Monitoring Plan and Schedule of Reports	Enclosure 10
Appendix 6: Standard Operating Procedures	Enclosure 11

Mantine, Kathie G Ms USAMRMC

**rom:**	Brosch, Laura R LTC USAMRMC
**Sent:**	Tuesday, January 07, 2003 8:22 AM
**To:**	Jarboe, Daniel L COL WRAIR-Wash DC
**Cc:**	Rothman, Sara W Dr. WRAIR-Wash DC; Ference, Jody WRAIR-Wash DC; Grogl, Max LTC WRAIR-Wash DC; Sun, Jennifer S CONTRACTOR WRAIR-Wash DC; Mantine, Kathie G Ms USAMRMC; Bennett, Jodi Ms USAMRMC; Lehnert, Erich K MAJ USAMMDA
**Subject:**	A-9768 HSRRB Approval

SUBJECT: Protocol Entitled, "Topical Treatment of Cutaneous Leishmaniasis With WR 279396: A Phase 2 Study in the Old World," Submitted by LTC Max Grogl, MS, Walter Reed Army Institute of Research, Silver Spring, Maryland, Study Log Number WRAIR #813, IND 50098, Office of the Surgeon General, Award Number DAMD17-02-2-0018, HSRRB Log Number A-9768

1. Documents received on 10 May 2002, 6 December 2002 and 26 December 2002 have been reviewed and found to comply with recommendations made at the 9 January 2002 meeting of The Surgeon General's Human Subjects Research Review Board (HSRRB). There are no outstanding human subjects protection issues.

2. The study is approved for implementation.

3. In accordance with 32 Code of Federal Regulations 219, a copy of the continuing review report approved by the IRB of Record for France and Tunisia should be submitted no later than 29 August 2003 and 27 September 2003 respectively.

4. Entry into the Volunteer Registry Database is required for this study.

5. The Principal Investigator should note the responsibilities described in HSRRB Clause 13.01 (encl).

6. Point of contact for this action is Ms. Mantine at kathie.mantine@det.amedd.army.mil or at DSN 343-2809 or (301) 619-2809.

LAURA R. BROSCH
LTC, AN
Acting Chair, Human Subjects
Research Review Board

Clause
13.01.pdf

REPUBLIQUE TUNISIENNE
MINISTERE DE LA SANTE PUBLIQUE
INSTITUT PASTEUR DE TUNIS

COMITE D' ETHIQUE MEDICALE

Tunis le 03 octobre 2002

P V 02 / 02

PROCES VERBAL
DE LA REUNION DU COMITE D' ETHIQUE MEDICALE
DE L'INSTITUT PASTEUR DE TUNIS
VENDREDI 27 SEPTEMBRE 2002

Le Comité d'Ethique Médicale de l'Institut Pasteur de Tunis s'est réuni le 27 septembre 2002 à 15 heures, pour statuer sur le dossier du Projet de réalisation d'un essai thérapeutique intitulé : " Topical treatment of Cutaneous Leishmaniasis with WR279396 : a phase 2 study in the old word ".
Le protocole soumis par le Docteur Afif Ben Salah, co-investigateur du projet, a fait l'objet de deux études antérieures lors des réunions du Comité en date du 25 décembre 2001 et du 29 mars 2002 (cf. P.V. en annexe). Comme suite aux recommandations et remarques exprimées par le Comité d'Ethique de l'Institut Pasteur de Tunis, et aux termes de son accord conditionnel à la mise en œuvre de l'essai, des modifications ont été apportées par les investigateurs aux documents initialement soumis.
Le Comité a été sollicité pour donner un avis final sur le dossier modifié, soumis par le Docteur Afif Ben Salah. Ce dossier comprend les documents suivants :

1. Le protocole modifié de l' essai thérapeutique
2. Le questionnaire destiné aux patients
3. Le contrat passé entre l' Institut Pasteur de Tunis et l' Institut Pasteur-Paris relatif à la conduite du protocole de recherche.
4. La version en langue arabe du Consentement éclairé
5. La version en langue arabe des documents à remettre aux patients.
6. la brochure des investigateurs.

A été jointe au dossier une copie du Cahier des Charges relatif à l'expérimentation médicale ou scientifique des médicaments destinés à la médecine humaine, signé conjointement par les Directeurs généraux de l'Institut Pasteur de Tunis et de la Direction de la Pharmacie et du Médicament du Ministère de la Santé Publique.

Après avoir pris connaissance du dossier et discuté des documents soumis, les membres du comité ont pris bonne note des mesures prises par les investigateurs pour la mise en conformité du protocole avec les recommandations exprimées à l'occasion des deux lectures précédentes. Ils relèvent en particulier les mesures prises pour :

- éviter les risques de préjudice esthétique pour les patients volontaires
- éviter le risque de grossesse chez les patientes à inclure dans l'essai
- étendre les critères d'exclusion aux manifestations neurologiques mentionnées

- assurer l'efficacité et la transparence requises pour la procédure de signalement des effets indésirables
- garantir la confidentialité concernant l' identité des patients volontaires.
- garantir et préserver les droits des volontaires, conformément aux dispositions de la législation tunisienne en vigueur.

Au terme de l'étude du dossier ainsi modifié, les membres du Comité de l'Ethique Médicale de l'Institut Pasteur de Tunis, ne voient pas d'objection d'ordre éthique à la conduite de cet essai thérapeutique.

Pour le Comité de l' Ethique Médicale
de l' Institut Pasteur de Tunis

Le Président du Comité
Pr M. Sami BOUBAKER

PASTEUR DE

Tunisian Republic
Ministry of Public Health
Institut Pasteur de Tunis
Medical Ethical Committee

Tunis Octobre 3rd 2002

Minutes of the Meeting of the ethical committee of Institut Pasteur de Tunis
Friday 27th of Septembre 2002

The medical ethical committee of « Institut Pasteur de Tunis » has met on the 27th of Septembre 2002 at 3.00 PM, in order decide on a project of a clinical trial titeled « Topical treatment of Cutaneous Leishmaniasis with WR279396 : a phase 2 study in the old world ». The protocol submitted by Dr. Afif BEN SALAH, co-investigator in the project, was subject of two previous reviews during the meetings of the 25th of Decembre 2001 and the 29th of March 2002 (cf. minutes in annex). Following recommendations done by the ethical committee of the « Institut Pasteur de Tunis », and after its conditional agreement to undertake the trial, modifications were achieved by investigators to documents initially submitted.

The committee was asked to give a final evaluation to the updated documents submitted by Dr. Afif BEN SALAH and containing the following :

1- The modified protocol of the trial
2- The case report form
3- The contract signed between Institut Pasteur de Tunis and Institut Pasteur de Paris for the research protocol
4- The arabic translated from english of the written informed consent
5- The arabic translated from english of the documents to be given to the patients
6- Brochure of investigators

Was added to these documents a copy of an agreement , related to medical or scientific experimentation of drugs for humans and co-signed by General Directors of Institut Pasteur de Tunis and Pharmacy and drugs of the Ministry of Health.

After knowledge and discussion of transmitted documents, members of the committee well noticed measures adopted by investigators to guaranty agreement between the protocol of the study and recommandations expressed during the two previous evaluations. The particularly notice the measures adopted for :

- Avoid the dysfigurement risk for volunteers
- Avoid the risk of pregnancy among included patients in the trial
- Extend the exclusion criteria to mentioned neurological manifestations
- Assure efficacy and transparency required for transmission of side effects
- Guarantee confidentiality regarding idendity of volunteers
- Guarantee and maintain rights of volunteers, as required by the Tunisian law

At the end of the study of the whole documents modified as described, members of the medical ethical committee of Institut Pasteur de Tunis do not have any ethical objection to the process of this clinical trial.

For the medical ethical committee
Of Institut Pasteur de Tunis
Professor M. Samir BOUBAKER

CONSULTATION COMMITTEE FOR THE PROTECTION OF INDIVIDUALS IN BIOMEDICAL RESEARCH

CCPPRB PARIS-COCHIN

Hôpital TARNIER - COCHIN
89, rue d'Assas, 75006 Paris

Secretarial Office: *Tel.: 33 (1) 46-33-68-67*
Fax: 33 (1) 46-33-70-46
E-mail: ccpprb.paris-cochin@wanadoo.fr

Chairman: Doctor C. GUERIN

Paris, 29 August 2002

REF: To be given in all correspondence

File No. 1791

CG/LG/CC 2002 393

An application was made to the Committee on 18/06/2002

by **Doctor Pierre Buffet**

for its opinion on the following research project:

No.: IND-50-98: "Topical treatment of cutaneous leishmaniasis with WR279396, a phase 2 study in the old world",

Research with Direct Individual Benefit

sponsored by: **INSTITUT PASTEUR**

The Committee examined the information relating to this project during the session dated

27 JUNE 2000

which was held in accordance with the stipulations in article R 2015 of the French Public Health Code.
The names of the Committee members who took part in this session are given in the list attached.

The Committee adopted the following decision: **FAVOURABLE OPINION**

Comments: the responses, dated 04/10/2001, 05/11/2001, 15/07/2002, 08/08/2002 and 29/08/2002, to the Committee's requests dated 24/08/2000, 06/11/2001, 22/07/2002, were taken into account for the favourable opinion.

Signed: Chairman

Doctor C. GUERIN

This favourable opinion if only valid if article 209.7 and articles R. 2047 to R. 2053 are observed.
The committee would be happy to be kept informed of any incidents arising during the application of this clinical research project and the closing date of your trial.

COMITE CONSULTATIF DE PROTECTION DES PERSONNES DANS LA RECHERCHE BIOMEDICALE

CCPPRB PARIS - COCHIN

Le Président :
Docteur Corinne GUERIN

Paris, le 30.08.2002

Secrétariat CCPPRB :
Hôpital TARNIER-COCHIN
89, rue d'Assas 75006 Paris
Téléphone : 33 (01) 46-33-68-67
Fax : 33 (01) 46-33-70-46
E-mail : ccpprb.paris@wanadoo.fr

AVIS DE TRANSMISSION DE
FAX TRANSMISSION SHEET

A l'attention de : Docteur Pierre Bassot
To : **n° FAX :** 01-40-61-30-19

Expéditeur : Docteur C.GUERIN
From :

Ce document comprend (y compris la feuille de transmission) : 3 **page(s)**
This document includes (including this transmission sheet) :

A rappeler dans toute correspondance :

**REF : Dossier N°** 1791	**Protocole N°** JND-5098

Suite à votre demande, veuillez trouver, ci-joint, la copie de l'avis favorable et la liste des membres, correspondant à l'examen du dossier (référencé ci-dessus) par le CCPPRB PARIS-COCHIN.

par courrier interne

L'original a déjà été adressé à l'investigateur principal par courrier Recommandé avec A-R.

Meilleures salutations.

p.o Secrétariat du CCPPRB
Laurence Galbert

L. Galbert

COMITE CONSULTATIF DE PROTECTION DES PERSONNES DANS LA RECHERCHE BIOMEDICALE

CCPPRB PARIS-COCHIN

Hôpital TARNIER - COCHIN
89, rue d'Assas , 75006 Paris

Secrétariat : Tél : 33 (1) 46-33-68-67
Fax : 33 (1) 46 33 70 46
E-mail : ccpprb.paris-cochin@wanadoo.fr

Le Président : Docteur C. GUERIN

A Paris, le 29 août 2002

REF : A rappeler dans toute correspondance :

Dossier N° : 1791

CC/LG/CC 2002.383

Le Comité a été saisi le 18/06/2002

par le **Docteur Pierre BUFFET**

d'une demande d'avis pour le projet de recherche :

N° : IND-50-98 : "Traitement topique de la leishmaniose cutanée par le WR279396, étude de phase II dans l'ancien monde" - "Topical treatment of cutaneous leishmaniasis with WR279396, a phase 2 study in the old world",

Recherche avec Bénéfice Individuel Direct,

dont le promoteur est : **INSTITUT PASTEUR**

Le Comité a examiné les informations relatives à ce projet lors de sa séance du

27 JUIN 2000

qui s'est tenue conformément aux dispositions de l'article R 2015 du Code de la Santé Publique.
Les noms des membres du Comité participant à cette séance figurent sur la liste ci-jointe .

Le Comité a adopté la délibération suivante : **AVIS FAVORABLE**

Commentaire : les réponses, datées du 04/10/2001, 05/11/2001, 15/07/2002, 08/08/2002 et 29/08/2002, aux demandes du Comité daté du 24/08/2000, 06/11/2001, 22/07/2002, ont été prises en compte pour l'avis favorable.

Signé : Le Président

Docteur C. GUERIN

Cet avis favorable ne vaut que si l'article 209.7 et les articles R. 2047 à R. 2053 sont respectés.
Le comité serait heureux d'être tenu au courant des incidents éventuels ayant pu surgir pendant l'application de ce projet de recherche clinique et de la date de clôture de votre essai.

01 46 33 70 46

COMITE CONSULTATIF DE PROTECTION DES PERSONNES DANS LA RECHERCHE BIOMEDICALE

1 ..

Siège : Hôpital TARNIER-COCHIN 89, rue d'Assas 75006 PARIS- Tél : 01.46.33.68.67 - Fax :01.46.33.70.46

Installation : 20 juin 1991
Agrément Ministériel : 19 juillet 1991

SESSION du 27 JUIN 2000

Bureau : Président : Michel DETILLEUX
Vice-Président : Chantal ZARADE
Trésorier : Yorick DROUAULT - Trésorier adjoint : Corinne GUERIN
Secrétaire : Bernard ASSELAIN

✓ Membres présents

CAT	NOMS	QUALITE
I	**Médecins ou personnes qualifiés en matière de recherche biomédicale**	
	Titulaires : ✓ Michel DETILLEUX	Médecine interne
	✓ Josette DALL'AVA-SANTUCCI	Physiologie
	✓ Françoise DOYON	Biostatisticien
	Yvon CALMUS	Hépato-gastroentérologie
	Suppléants : Mostafa MOKHTARI	Pédiatre
	Anh Tuan DINH-XUAN	Physiologie
	✓ Bernard ASSELAIN	Biostatisticien
	Jean VARIN	Cardiologie
II	**Médecins Généralistes**	
	Titulaire : ✓ Gérard GRILLET	
	Suppléant à désigner	
III	**Pharmaciens**	
	Titulaires : Corinne GUERIN	Pharmacien hospitalier
	✓ Marie-Lucie BRUNET	"
	Suppléants : Françoise LECOURT	Pharmacien d'officine
	✓ Georges HAZEBROUCQ	Pharmacien hospitalier
IV	**Infirmiers(ières)**	
	Titulaire : ✓ Nelly MELI	Infirmière établ.hospitalier
	Suppléant : Ronan AUFFRET	"
V	**Personnes qualifiées en matière d'éthique**	
	Titulaire : ✓ José PEREZ	Courant de pensée
	Suppléant :	
VI	**Personnes qualifiées dans le domaine social**	
	Titulaire : ✓ Huguette ALASSIMONE	Assistante sociale
	Suppléant : ✓ Simone DALLE	"
VII	**Psychologues**	
	Titulaire : ✓ Jean-Pierre LECANUET	
	Suppléant : à désigner	
VIII	**Personnes qualifiées en matière juridique**	
	Titulaire : Chantal ZARADE	Avocat
	Suppléant : Anne-Marie LANGELLIER	Magistrat

CCPPRB PARIS-COCHIN
HOPITAL TARNIER
89, rue d'Assas
75006 PARIS

p.o [signature]

6

ASSURANCE: FWA00003327 - Institut Pasteur NEW SEARCH
Located at: PARIS , FRANCE
Approved: September 12, 2002 **Expires:** September 12, 2005

No Assurance Components Identified

IRBS LINKED TO THIS ASSURANCE					
Ident	Name	City	U.S. or Foreign	Location Code	
IRB00001072	CCPPRB Med Sch Necker-Enfants Malades IRB#1	PARIS	F	FR	Detail
IRB00001956	CCPPRB PARIS-COCHIN IRB #1	PARIS	F	FR	Detail

Agency Only Access

Title: Topical Treatment of Cutaneous Leishmaniasis with WR279396: A Phase 2 Study in the Old World

PROTOCOL

Version 7

IND 50,098

Log No. A-97-68

17 June 2002

1.0 GENERAL INFORMATION (SUMMARY PAGE)

Protocol title: Topical Treatment of Cutaneous Leishmaniasis with WR 279396: A Phase 2 Study in the Old World

IND/Date of submission	50,098
IRB Protocol #/Date Approved	No. 1791 / (08/29/2002) (Paris); (09/27/2002) Tunis
HSSRB Protocol #/Date Approved	Log No. A-97-68/ (01/07/2003)
Sponsor	The Office of the Surgeon General (OTSG)<br>Chief, Human Subjects Protection Division<br>U.S. Army MRMC,<br>Fort Detrick, MD 21702-5012
Co-sponsor	Institut Pasteur, Rue du Dr. Roux, Paris, France
Principal Investigator:<br>Max Grogl, Ph.D., LTC, MS	Department of Biologics Research, WRAIR<br>Phone: 301-319-9359
Associate Investigators:<br>Pierre Buffet, MD<br>Study Coordinator	Centre de Recherche Clinique de l'Institut Pasteur,<br>25-28 rue du Dr. Roux, 75015 Paris, France<br>Phone: 011-33-1-40613817 Fax: 33-6-164061-3977
Afif Ben Salah, MD<br>Study Coordinator	Institute Pasteur, Tunisia<br>Phone 011-216-71-792-429 Fax: 011-216-71-791-833
Study Monitor: (USAMRMC Reg 70-25)<br>Jonathan D. Berman, MD, COL, MC	Division, Experimental Therapeutics, WRAIR<br>Phone: 301-594-7105
Medical Monitor Paris:<br>Odile Launay, MD	Centre de Centre des essais vaccinaux Cochin-Pasteur, Hôpital Cochin, Pavillon Saint-Louis, 27 rue du Faubourg Saint-Jacques, 75679 Paris Cedex 14, France<br>Phone : 011-33-1-4407-1541<br>Fax : 011-33-1-4046-9308
Medical Monitor Tunis:<br>Hechmi Louzir, MD	Institut Pasteur de Tunis<br>13 Place Pasteur BP, 74 Belvedere 1002, Tunis, Tunisia<br>Phone: 011-216-1-783-022 #213

Product Manager:
Erich Lehnert, Ph.D., MAJ, MS

U.S. Army Medical Materiel Development Activity (USAMMDA)
622 Neiman Street, Fort Detrick, MD 21702-5009
Phone: 301-619-2053

WRAIR Consultant:
Brian G. Schuster, MD, COL, MC

Headquarters WRAIR
Phone: 301-319-9838

Study Location:
Paris, France
Tunis, Tunisia

Clinical Laboratory:
Medical Center Institut Pasteur, Paris, France

Centre Médical de l'Institut Pasteur,
25-28 rue du Dr. Roux, 75015 Paris, France

Institute Pasteur, Tunis, Tunisia

Institut Pasteur de Tunis
13 Place Pasteur BP, 74 Belvedere 1002, Tunis, Tunisia

Study Duration: 18 Months

Anticipated Start Up Date: Spring 2003

Required No. of Volunteers: 100

1.0 GENERAL INFORMATION 2

2.0 STUDY OBJECTIVE 7

3.0 STUDY SUMMARY 7

4.0 FACILITIES TO BE USED 7

5.0 BACKGROUND OF LEISHMANIASIS 7
5.1 Natural history and drug treatment of OW cutaneous leishmaniasis 7
5.1.1 Old World cutaneous leishmaniasis 7
5.1.2 Cutaneous leishmaniasis in France 8
5.1.3 Cutaneous leishmaniasis in Tunisia 9
5.2 Present topical agents 9
5.3 WR 279396 preclinical toxicity 10
5.4 Clinical efficacy of WR279396 11
5.5 Toxicity of WR279396 11
5.5.1 Preclinical toxicity of WR279396 11
5.5.2 Clinical toxicity of components of WR 279396 12
5.5.2.1 Paromomycin 12
5.5.2.2 Gentamicin 13
5.5.3 Clinical toxicity of WR279396 - Phase 1 study 14
5.5.4 Clinical toxicity of WR279396 15
5.5.4.1 Summary of a phase 2 study in the NW 15
5.5.5 Summary of toxicity 15
5.5.6 Chemistry and manufacturing 16

6.0 SUMMARY AND RATIONALE FOR PHASE 2 STUDY 16

7.0 EXPERIMENTAL PLAN 17
7.1 Trial Design 17
7.2 Trial primary endpoint and the secondary endpoint 17
7.3 Study Population/Site 18
7.4 Number and randomization of lesions 18
7.5 Inclusion criteria 18
7.6 Pre enrollment evaluation 18
7.7 Exclusion criteria 19
7.8 Duration of Study for first cohort 19
7.9 Dosage and Administration of Drugs 20
7.10 Determination of adverse reactions 20
7.10.1 Determination of side effects 20
7.10.2 Determination of laboratory evidence of side effects 21

7.11 Establishment of diagnosis 21

7.12 Definition of responses using clinical criteria 21
7.13 Parasitological results 21
7.14 Definition of cure 21
7.15 Criteria for early withdrawal from the study 22
7.16 Pharmacokinetics evaluation 22
7.17 Concomitant medications 22
7.18 Departure from the protocol 22
7.19 Procedural timetable 23

8.0 SAMPLE SIZE AND DATA ANALYSIS **23**
8.1 Sample size calculation 23
8.2 Efficacy calculation 24
8.3 Treatment randomization codes 24
8.4 Missing unused data 24

9.0 DIRECT ACCESS TO SOURCE DATA/DOCUMENTS **24**

10.0 QUALITY CONTROL AND QUALITY ASSURANCE **24**

11.0 ETHICS **24**

12.0 DATA HANDLING AND RECORDKEEPING **25**

13.0 FINANCING **25**

14.0 PUBLICATION POLICY **25**

15.0 VOLUNTEER REGISTRY DATA BASE REQUIREMENTS **25**

16.0 ASSESSMENT OF SAFETY **26**
16.1 Safety parameters / Adverse events 26
16.1.1 Adverse event 26
16.1.2 Serious adverse event 26
16.2 Recording and reporting adverse events 26
16.2.1 Documenting adverse events 27
16.2.2 Reporting adverse events 27
16.2.3 Follow-Up of adverse events 27
16.3 Study specific definitions and exceptions 27

17.0 ROLES AND RESPONSIBILITIES OF THE STUDY PERSONEL **27**

18.0 REFERENCES **28**

19.0 SIGNATURE OF PRINCIPAL INVESTIGATORS AND MONITOR **31**

APPENDIX A-1 **32**

Part A English Informed Consent Form

APPENDIX A-2 **35**
Part A French Informed Consent Form/ Consentement de participation

APPENDIX A-3 **38**
Part B English Informed Consent Explanation

APPENDIX A-4 **46**
Part B French Explanation of The Consent Form/ Formulaire d'information des Patients

APPENDIX B
English Volunteer Donation Form **59**
French Volunteer Donation Form **60**

APPENDIX C **61**
Certification of Translation

APPENDIX D **62**
Adverse or Unexpected Event Determination – side effects and grades

APPENDIX 1 - FDA FORM 1572

APPENDIX 2 - CURRICULUM VITA OF PRINCIPAL INVESTIGATOR

APPENDIX 3 - ADVERSE EVENT NOTIFICATION AND FORM

APPENDIX 4 - CASE REPORT FORMS

2.0 STUDY OBJECTIVE

Evaluation of the efficacy and tolerance of topical WR279396 in the treatment of Old World cutaneous leishmaniasis.

3.0 STUDY SUMMARY

One hundred lesions of Old World cutaneous leishmaniasis will be randomly allocated to WR279396 treatment (50 lesions) or placebo treatment (50 lesions) for 20 days. All patients will be rescued with Glucantime (intralesional or parenteral) or fluconazole if lesion does not heal spontaneously or the primary care physician drug of choice. Maximum treatment delay time in the placebo group will be 50 days. The active ingredients in WR 279396 are two aminoglycosides-- paromomycin sulfate (15%) and gentamicin sulfate (0.5%)-- in a base (AQIC).

Efficacy will be evaluated in terms of the number of lesions cured at 30 days after the end of therapy (i.e., 50 days from the start of treatment) and the number of recurrences during 6 months of observation. Toxicity will be evaluated by local adverse reactions and by laboratory signs of systemic events.

4.0 FACILITIES TO BE USED

Patients with presumed leishmaniasis would be examined at the Institut Pasteur Medical Center in Paris, or by a team from the Institut Pasteur in Tunisia. Patients will be treated as outpatients. These two centers have laboratories that routinely performs standard clinical tests (urinalysis, serum chemistries, complete blood counts). All the parasitological exams will be done at the Clinical Laboratory, Pasteur Medical Center, in Paris or at the Clinical Laboratory of the regional hospital of Sidi-Bouzid in Tunisia. The pharmacokinetic studies will be done by the Division of Experimental Therapeutics, WRAIR in Washington, DC.

5.0 BACKGROUND OF LEISHMANIASIS

5.1 NATURAL HISTORY AND DRUG TREATMENT OF OLD WORLD CUTANEOUS LEISHMANIASIS

5.1.1 Old World cutaneous leishmaniasis

Cutaneous leishmaniasis typically presents as a papule that enlarges over approximately 1 month into an ulcer with raised edges and a necrotic center (1). The mean area per ulcer in one study was 400 mm2 (2). Nevertheless, rare ulcers are 2,000 $mm^{2,}$ and a rare patient may have 20 ulcers. The spontaneous cure rate over 6-8 weeks for cutaneous leishmaniasis caused by members of the *L. major* or *L. tropica* complex in the Old World ranges from 15-45% (3,4). The most reliable figures come from *L. major* disease in Tunisia and Iran , for which rates of 20-44% are reported (3,4). Although frequently follow-up in such studies is performed for 1 year, in a recent study of 27 relapsed lesions (5), only 2 occurred between 6 and 12 months after the end of therapy, and followup for 6 months therefore seems sufficient.

Standard treatment is with intralesional injections of pentavalent antimonials. When 1-3 ml of Glucantime were administered intradermally once a week for 5 consecutive weeks to 20 patients with *L. tropica* in Syria, a cure rate of 76% was achieved (6). This regimen of Glucantime is considered successful in most *L. tropica* or *L. major* endemic areas, although these results are substantiated by only one controlled study. However, the regimen requires at least five painful intradermal injections which can hardly be performed in children and/or when lesions are located near eyes, mouth or nose. The overall cost of one intradermal injection is about $30 in France; $150 for a complete treatment course. For multiple and/or periorificial lesions that can not be injected, standard treatment is with pentavalent antimonials at a dosage of 10-20 mg antimony (Sb)/kg/day for 10-20 days. This regimen of Pentostam or Glucantime is successful in Central and South America with a ~90% cure rate with 1 year follow up. A lower cure rate (50%) of cutaneous leishmaniasis was achieved in Algeria in a *L. major* endemic area (7). The regimen requires 20 days of injections for an outpatient dermatological disease, is expensive (~US$200 per course), and is potentially or frankly toxic to the heart, liver, pancreas, hematopoietic system, and musculoskeletal system (8).

The attempt to find alternatives to the above antimonial regimen has included oral agents such as ketoconazole, fluconazole and allopurinol, and short courses of antimonials and pentamidine. The oral agents have not been shown to be >75% curative (8, 35). On the other hand, a 10 day course of Pentostam, 20 mg/kg/day intravenously for 10 days (9), and a 4-injection course of pentamidine administered over 7 days (3 mg/kg qod for 4 injections)(9) have been reported to be ~90% curative in the New World.

5.1.2 Cutaneous leishmaniasis in France

Incidence: Cutaneous leishmaniasis is a rare disease in France with about 30 reported cases per year. Of these 30 cases 80% were acquired during short trips to Old World Countries (10). In one retrospective study 3% of patients suffering with dermatosis acquired in a tropical country were diagnosed with CL (11). Recently, 15-50 cases were reported by public hospitals in Paris in 97, and 98 (PAB & CS Unpublished). It is expected that 1 to 5 patients will be enrolled in the protocol per month.

Clinical: Most lesions due to *L. major* or *L. tropica* (the two main dermotropic Old World Leishmania species) are simple cutaneous lesions with the "classical" circular leishmania ulcer, with elevated discolored borders and sharply incised central crater. Nevertheless, there are wide variations and all lesions do not have this distinctive appearances. In a survey of school children in a *L. tropica* focus in Iran, 1.3% of the children had active lesions and 14.3% had scares (12). The number of active lesions or scares per child ranges from 1 to 10. The majority (80%) had 1 lesion, 12.4% had 2 lesions, and 5.3% had 3 or more lesions. The face was the part of the body most commonly involved (63.6%), followed by the hands (20.9%), legs (12.8%) and other parts of the body (2.7%). Mucosal disease due to Old World Leishmania species is rare, probably < 1% (10). Although rare, this complication will be ruled out in this study by a physical and dermatological exam of mucosal tissues.

Facilities to be used: The Institut Pasteur Medical Center in Paris provides care to the city of Paris and surrounding counties. The Center has a laboratory that routinely performs standard Clinical tests (urinanalysis, serum chemistries, complete blood count). All the parasitological exams will be done at the Clinical Laboratory of the Pasteur Medical Center. A qualified technician, nurse or physician at the Center will perform hearing tests.

5.1.3 Cutaneous leishmaniasis in Tunisia

Incidence: Three different forms of cutaneous leishmaniasis occur in Tunisia. *L. major* cutaneous leishmaniasis, by far the most frequent, is epidemic in Central and South of Tunisia, whereas *L. infantum* cutaneous leishmaniasis is found in the North and *L. killicki* in the Southeast. From 2,000 to 3,000 cases are reported per year to the Tunisian Ministry of Health. The age distribution varies among the focuses. However, age distribution is skewed to young groups in the old foci and includes all age groups in the newly colonized ones.

Clinical: In a previous clinical trial, 75-80% of the lesions (all due to *L. major*) were at least partially ulcerative in nature.

Facilities to be used: The disease is now reemerging as an epidemic in rural areas in central Tunisia. We expect that patients will emerge from September to March with a peak in December. The medical team from Tunisia will include 10-15 patients at one time. Treatment and follow-up of the patients will be done on site. Members of the medical team will live in the study area until the end of the project.

5.2 PRESENT TOPICAL AGENTS

An alternative approach to a dermatological problem such as CL is a topical agent that can be applied directly to the lesion.

An Israeli topical formulation of paromomycin sulfate (15%) and methylbenzethonium chloride (12%) in soft white paraffin has been tried and is now marketed for Old World cutaneous leishmaniasis. Israeli *L. major* lesions treated with the formulation for 10 days cleared significantly more rapidly (100% cure at 21-30 days) than did untreated lesions on the same patients (100% cure at 51-60 days) (13). The primary agent in the topical formulation, paromomycin, is an aminoglycoside antibiotic analogous in structure to the antibacterial agent neomycin. Although neomycin is solely an antibacterial agent, paromomycin is clinically effective against protozoan and has good in vitro activity vs Leishmania amastigotes with an ED (100) of about 10 µg ml (14). The other agent in the topical formulation, methylbenzethonium chloride (MBCL), is a cationic quaternary ammonium antibacterial disinfectant present up to 0.02% concentration in shampoos that also have good in vitro antileishmanial activity [ED (100) = 5 µg ml] (15).

A different topical formulation of paromomycin sulfate (15%), 10 % urea in white soft paraffin was investigated in two randomized, double-blind, placebo controlled trials in children patients with *L. major* cutaneous leishmaniasis in Tunisia and Iran (3,4). The results were

equivalent in both trials; the paromomycin ointment was safe, well tolerated and there was clear evidenced of parasitologic efficacy. However, there was no clear clinical benefits with this formulation. The authors concluded that the ointment should be studied in longer or more frequent regimens in an effort to prevent parasitologic relapse and thus promote clinical improvement. In addition, these trials confirmed, that only placebo-controlled clinical trials could demonstrate the efficacy of a new therapeutic agent in *L. major* CL since it is a fast healing condition.

In the New World, we determined the therapeutic index of paromomycin sulfate (15%) MBCL (5%) in paraffin against cutaneous leishmaniasis (16). A 10-day course, in combination with a 3-day course of Glucantime, was administered to Colombians with *L. panamensis* disease. Eight of 19 patients (42%) cured with 12 months follow up. Since this cure rate is low, it is clear that this topical formulation alone would not have been sufficiently curative. In a second cohort, a 10-day course of topical was administered with a 7-day course of Glucantime. Eighteen of 20 patients (90%) cured. Since 7 days of Glucantime by itself cures < 40% of patients, this trial indicates that 10 days of the topical plus 7 days of Glucantime, both of which are poorly curative by themselves, are highly curative in combination. Dermatological side effects consisted of burning and pruritus in 25% of patients and vesicle formation in 15% of patients. A second clinical study by Soto et. al., 1998 reconfirmed the first study.

These are the first reports that a regimen partially comprised of topical antimicrobials can be highly effective for New World cutaneous leishmaniasis. Nevertheless, the paromomycin/ MBCL/ paraffin topical formulation suffers in terms of efficacy (7 days of adjunctive injections were required for cure) and toxicity (40% of patients reported side effects).

5.3 WR279396 PRECLINICAL TOXICITY

Preclinical efficacy of WR279396 was compared to paromomycin sulfate (15%)/ MBCL (12%)/ paraffin in the topical treatment of cutaneous disease in BALB/c mice. Sixty-day old lesions were treated twice a day for 10 days, and the response to therapy was monitored over a further 70 days (TABLE). For ulcers due to *L. major*, >90% of lesions healed by day 20 after therapy in both treatment groups. Nevertheless, on day 10, 93 % of the lesions were healed with WR279396 while only 70% of the lesions were healed with the paromomycin sulfate (15%)/ MBCL (12%)/ paraffin formulation. For ulcers due to *L. panamensis* or *L. amazonensis*, all lesions treated with WR 279396 healed and did not relapse. Less than half of lesions treated with paromomycin-MBCL-Paraffin were healed by day 30, and all lesions had relapsed by day 70 after therapy. Since in contradistinction to paromomycin/MBCL, WR 279396 cured both Old and New World cutaneous leishmaniasis in mice, the latter formulation is the only topical suggested by preclinical efficacy data to be a candidate for the treatment of both New World and Old World cutaneous leishmaniasis.

TABLE: Efficacy of topical paromomycin-gentamicin-AQIC (WR279396 = WR) compared to paromomycin-MBCL-paraffin (PM) in *L. major*, *L. panamensis*, and *L. amazonensis* infected BALB/c mice.

Day After Therapy	*L. major* % Healed		*L. panamensis* % Healed		*L. amazonensis* % Healed	
	PM	WR	PM	WR	PM	WR
0	0	0	0	0	0	0
10	70	93	10	80	13	63
20	90	100	83	100	30	100
30	100	100	47	100	37	100
40	93	100	23	100	0	100
50	96	100	23	100	0	100
60	93	100	20	100	0	100
70	82	100	0	100	0	100

5.4 CLINICAL EFFICACY OF WR279396

5.4.1 Summary of a phase 2 study in the New World

In 1999, a pilot phase 2 study was conducted in Colombia. This clinical study was randomized, double blind trial of WR279396 compared to placebo (the cream base of WR 279396), each administered twice-a-day for 20 days.

In the active group, 36 of 51 evaluable lesions cured (71%) and 5 lesions were non-evaluable. In the placebo group, 6 of 12 evaluable lesions cured (50%: p = 0.4 in comparison to the active group) and 5 lesions were non-evaluable.

There was no statistical difference between the pre-treatment sizes for cured vs failed lesions in WR 279396 patients (p = 0.7) nor in placebo patients (p = 0.8).

The 36 lesions that were treated with WR279396 were recognized as cured at the end of therapy on day 20 (20 lesions), at the first follow up on day 45 (13 lesions), and on day 90 (3 lesions). The 6 placebo lesions that cured were recognized on day 20 (1 lesion), day 45 (3 lesions), and day 90 (2 lesions). The mean cure time of 35 days for cured WR 279396 lesions was statistically different (p = 0.04) from the mean cure time of cured placebo lesions (56 days).

5.5 TOXICITY OF WR279396

5.5.1 Preclinical toxicity of WR279396

Dermatologic toxicity was evaluated in 3 GLP studies. In a study of allergic contact dermatitis (17), WR279396 was non-sensitizing. In a study of photo activated dermal irritation (18), WR279396 was non-irritative. In a study of photoallergic contact dermatitis (19). WR 279396 was concluded to have the potential to be a weak photoallergen.

Systemic toxicity after topical administration was evaluated in 1 GLP study (20). Rats (250 mg) were administered 10, 50, or 250 mg/kg of paromomycin in the form of WR279396 to abraded back twice a day for 5 days and then daily for 23 days. The change from twice daily dosing to once daily occurred on day 6 because by day 5, moderate to severe erythema (draize score of 3) was seen, primarily at the site of initial application of the material, on the mid and high dose animals. In contrast to twice-daily dosing, daily dosing did not result in clinical skin reaction. This indicates that irritation due to WR279396 in rats is rapidly (within 24 hrs) reversible. No significant abnormalities were seen on clinical pathology or histopathology, including BUN, creatinine, histopathology of the kidney, and histopathology of the sensory and vestibular hair cells of the ear, in the high dose group.

5.5.2 Clinical toxicity of components of WR279396

All chemotherapeutic agents in WR279396 (paromomycin and gentamicin) have been administered both topically and systemically to humans. Paromomycin was first isolated from a sample of Italian soil in 1959 (21), and clinical reports on its use date from the early 1960's. Gentamicin was released for general use in approximately 1968 (22). The target organs for both topical and systemic drug have therefore been identified.

5.5.2.1 Paromomycin:

a) Systemic toxicity after parenteral injection

Paromomycin injectable is licensed in Italy with a recommended dosage of 500 mg twice-a-day intramuscularly (15 mg/kg for children aged 1-12 years) for 10 days (21: page 124). Peak and trough serum levels with this regimen were 15-25 µg ml and 1-2 µg ml, respectively (21: page 88). Rat data suggested that nephrotoxicity and eighth nerve toxicity (acoustic > vestibular) may result from high dosing (21: pages 62-65). In addition, when 12 children were administered standard doses of 20-30 mg/kg/day for 15 days, there was no incidence of abnormal BUN, vestibular damage, or hearing damage (21: pages 95-96). When results from larger numbers of patients were summarized, systemic side effects in 2397 patients were: renal hypofunction (2 patients), jaundice (1), ear buzzing (2), temporary cochlear damage (1), hypacusic (7) (21: page 101).

b) Systemic toxicity after oral administration

The only formulation of paromomycin presently marketed in the United States is oral paromomycin formulated as Humatin. The indication is intestinal amebiasis treated with 25-35 mg/kg daily for 5-10 days. Since "almost 100% of the drug is recovered in the stool", paromomycin is not absorbed through intact intestinal mucosa (23). It is therefore extremely unlikely that clinically significant absorption of paromomycin or gentamicin would occur through intact skin.

c) Topical toxicity after topical administration

In the study of paromomycin (15%)/MBCL (5%)/paraffin in Colombia, 8 of 20 patients (40%) experienced local symptoms (16). Three (3) patients experienced a burning sensation and pruritus during the first 3 days of topical administration and 2 patients experienced these symptoms intermittently throughout the 10 days of topical administration. Three (3) patients experienced the more severe local side effect of an eczematous reaction with erythema and vesicle formation. Two (2) of these three patients had vesicle formation only on the first 2 days of therapy, but one patient had vesicles throughout the 10-day period of treatment. In another study, 3 of 30 patients treated with paromomycin (15%) /MBCL (12%)/paraffin had to prematurely terminate therapy due to severe pain and edema whereas 0 of 9 patients treated with paromomycin (15%)/MBCL(5%)/paraffin reported such toxicity (24). This study suggests that side effects due to the formulation might be due to MBCL rather than to paromomycin. On the other hand, 2 of 20 patients administered paromomycin (15%)/ paraffin--a paromomycin-containing topical without MBCL-- experienced pruritus, tenderness, and edema (25).

5.5.2.2 Gentamicin:

a) Systemic toxicity after parenteral injection

Gentamicin injectable is marketed in the USA with the customary recommended regimen of 1 mg/kg every 8 hours for 7-10 days (26). Peak serum levels with this regimen are approximately 5 µg/ml. Peak and trough levels beneath 12 µg/ml and 2 µg/ml are recommended to avoid systemic side effects, which primarily consist of nephrotoxity and neurotoxicity. It may be that appropriate peak levels correspond with minimization of ototoxicity and appropriate trough levels correspond with minimization of nephrotoxicity.

Nephrotoxicity is characteristically reversible. Among 71 patients given 77 courses of gentamicin, 32 demonstrated increases in BUN and serum creatinine probably due to gentamicin, and in all cases the indices of renal function returned to pretreatment values after the drug was stopped (22).

Neurotoxicity is manifested by eighth nerve dysfunction. Both vestibular damage (dizziness, vertigo) and auditory damage (high-toned tinnitus and hearing loss) are seen, but high-toned hearing loss is often the first sign of eighth nerve damage (26).

When 1,484 courses in which gentamicin was administered were examined, there was ototoxicity in 44 cases (3%) (27). Only vestibular dysfunction was seen in 2/3 of the cases; decreased vestibular and auditory function was seen in 1/6 of patients; decreased auditory function alone was seen in 1/6 of patients. All cases occurred less than 14 days after discontinuation of treatment. In 14 of 29 cases of vestibular dysfunction for which the duration of the abnormality was determined, the dysfunction was reversible. (28)

Data such as these have led to the following statements in the PDR: "The risk of toxic reactions is low in patients with normal renal function who do not receive Garamycin [gentamicin] injectable at higher doses or for longer periods of time than recommended." (22)

b) Systemic absorption and toxicity after topical administration

Stone et al (29) determined absorption of gentamicin from a 0.1% cream and a 0.1% ointment (i.e., gentamicin in bland petrolatum) applied to the skin for burn patients. Absorption was quantitated by bioassay on urine collected for 3 days after application of the topical formulation. When formulations were administered for 3 days, the maximal drug absorbance was 20% from the cream and 5% from the ointment. Absorption from the cream was highest for fresh wounds; absorption from the ointment was highest after the eschar had separated. The authors postulate that when the burn had a large water content, as occurred in the immediate postburn period with local edema, absorption from the water-miscible cream was greatest. When there was a minimal amount of exudate after the eschar separated, absorption from the ointment was maximal.

If a topical gentamicin is applied to a huge area of burned skin, absorption can be sufficient to result in typical gentamicin toxicity. Two patients with severe burns covering 60-80% of the body were treated solely with topical gentamicin, presumably the 0.1 % cream in use in 1974. Gentamicin levels in the serum reached 3.0 - 4.3 μg/ml, and both patients showed loss of hearing and of vestibular function (30).

Aminoglycosides have also been applied topically to the ear and to the eye. For Meniere's disease, intratympanic delivery of gentamicin is intended to expose the inner ear to sufficient drug to inhibit the excess activity of the vestibular apparatus that is causing vertigo. A standard protocol is to administer 1 ml of 27 mg TID (81 mg/day) for at most 4 days (31). For the intravitreal administration of aminoglycosides for endopthalmitis, the standard doses of 0.1-0.2 mg of gentamicin sulfate or 0.2-0.4 mg of amikacin sulfate may have the adverse effect of macular damage (32).

5.5.3 Clinical toxicity of WR279396: Phase 1 study (33)

The aims of the phase I study were to investigate irritancy and phototoxicity due to WR 279396. In addition, blood was drawn to determine systemic toxicity (absorption of the aminoglycosides, alteration of kidney function and auditory function). In the irritancy study, 30 volunteers were administered WR279396, placebo cream (the AQIC base that contained urea), and white petrolatum to separate areas on the upper arm in a double-blinded manner for 15 days over a 20 day period.

The results of the irritancy study are:

a) Active cream (WR279396): 12 volunteers had 1-4 erythematous papules for 1-6 of the 15 days of examined application; for 8 volunteers, the papules advanced to pustules; for 3 volunteers, the lesions evolved into erosions.

b) Placebo cream (AQIC containing urea): 29 volunteers had fine wrinkling of the skin for 1-8 of the 15 days of application; 26 had +/- erythema (faint redness) for 3-10 days; 9 had 1+

erythema (erythema and edema of entire site) for 1-2 days. No patient had 2+ or 3+ erythema (vesicular changes or bullous changes).

One volunteer had acute dermatitis that developed at the sites both of active and placebo cream, that resolved. The volunteer was rechallenged with all ingredients in WR279396 known to cause allergic contact dermatitis, with negative results.

The interpretation of the irritancy study is that WR279396 can produce follicular irritant dermatitis with erythematous papules, and less frequently pustules and erosions. In addition, the urea in the AQIC vehicle, when it decomposes and is unbuffered by the aminoglycosides in WR 279396, characteristically produces mild irritancy.

In the phototoxicity study, 10 of the volunteers also received a challenge with ultra-violet light to areas of skin to which either WR279396 or the control substance, white petrolatum, was previously applied for 30 minutes. WR279396 was no different from the control substance in the degree of erythema produced.

In terms of systemic toxicity: examination of blood drawn on the last day of application or the following day, revealed that no volunteer had measurable blood levels (> 100 ng/ml) of paromomycin or of gentamicin. There was no statistical change in a kidney function test (creatinine). In audiometric tests pre-and post- topical application, more volunteers showed increases in high-frequency hearing than showed decreases in hearing.

5.5.4 Clinical toxicity of WR279396

5.5.4.1 Summary of a phase 2 study in the New World

In the 1999, randomized, double-blind pilot study of WR279396 compared to placebo, each administered twice-a-day for 20 days in Colombia, no patient demonstrated an increase in creatinine values to higher than normal values (1.5 mg/dL) by the end of therapy. In addition, side effects in both groups consisted entirely of grade 1 local pain (describe as pain that does not interfere with daily activity). This grade 1 pain lasted a mean of 4 days in 55% of active patients and 3 days in 33% of placebo patients.

In Colombian cutaneous leishmaniasis, WR279396 was therefore demonstrated to be a non-toxic topical formulation that significantly diminished lesion cure time.

5.5.5 Summary of toxicity

The potential dermatological side effects of topical paromomycin consist of pruritus/ pain/ tenderness, erythema, edema, and vesicle formation. These side effects may result either from irritation or, particularly in persons previously sensitized to neomycin or gentamicin, allergic reactions.

Systemic side effects due to paromomycin and gentamicin are primarily due to kidney damage (increased BUN and creatinine) and 8th nerve damage (high-toned hearing loss, vertigo).

At least for gentamicin, these systemic side effects generally occur when serum concentrations greater than that achieved with customary parenteral doses are administered. There are no studies in which the toxicity of paromomycin combined with gentamicin have been compared to the toxicity of the separate aminoglycosides. However, as mentioned above, in Colombia, WRAIR's formulation show no signs of systemic side effects and prove to be safe.

.In the phase 1 study of dermatological irritancy and photoirritancy, WR 279396 caused slight follicular irritancy in 40% of volunteers when administered for 15 days over a 20-day period of time.

5.5.6 Chemistry and manufacturing

The components are synthesized under GMP by Farmitalia (Paromomycin) and by Schering (Gentamicin). Formulation of WR279396 is performed under GMP by the University of Iowa. Analysis of formulated WR279396 was within 6% of labeled paromomycin amount and 10% of labeled gentamicin amount (April 2002).

Formulation stability was determined over 8 weeks. At 45^0C, there was a 48% decrement in paromomycin and a 36% decrement in gentamicin. At 35^0 C, there was a 17% decrement in paromomycin and a 9% decrement in gentamicin. At room temperature (19-26^0C), there was a 3% decrement in paromomycin and 2% decrement in gentamicin. Therefore, WR279396 must be stored at ~25^0 C or in the refrigerator (~5^0 C).

6.0 SUMMARY AND RATIONALE FOR PHASE 2 STUDY

The overall objective of this study is to determine the efficacy and toxicity of WR279396 administered topically to lesions of Old World cutaneous leishmaniasis.

In the case of multiple and/or periorificial lesions, a topical antileishmanial preparation would eliminate the problems of parenteral administration, systemic toxicity, and cost associated with the presently effective antileishmanial regimens (20 days of Glucantime). In the case of Old World simple cutaneous leishmaniasis a topical preparation would eliminate the problems of painful intradermal injections which can hardly be performed in children and/or when lesions are located close to the eyes, mouth or nose. Cost of treatment would be significantly reduced.

WR 279396 was more active than topical paromomycin/MBCL. Since paromomycin/MBCL has been proven to be active against Old World leishmaniasis, WR279396 has a strong possibility of being curative by itself against clinical OW cutaneous leishmaniasis.

The drug regimen chosen is the maximum amount of drug that can be applied to a cutaneous lesion (0.0005 ml per mm2 of lesion, twice-a-day) for the maximum amount of time that patients would accept being treated (20 days).

If the topical is ineffective against the cutaneous lesion, failure at the lesion site should occur during treatment or the 6-months of follow up and the patient can be rescued with

Glucantime or the attending physician drug of choice. Failure in the sense of nasal metastasis (mucosal disease) does not occur.

With respect to toxicity, both the paromomycin and gentamicin components of WR 279396 have been topically and systemically administered to humans. Possible local reactions to topical paromomycin and gentamicin are pruritic, erythema, and edema. When irritancy and photoirritancy of WR279396 were assessed in normal volunteers in a phase I study, almost all volunteers experienced wrinkling of the skin and mild erythema without edema. In this phase II trial, significant dermatological side effects should be preventable by monitoring local reactions and stopping treatment if necessary.

The literature review in section 2.4 shows that the target organs of toxicity of the aminoglycosides in WR279396 are the kidney and 8th nerve. In terms of possible systemic toxicity in this study: the maximum dose a patient can receive is the amount needed to coat a cumulative total lesion surface area of 2,500 mm2 lesion twice a day for 20 days. Since application of the cream will be at the rate of 0.0005 ml per mm2, the maximum dose is 1.25 ml twice-a-day for 20 days, or 50 ml. 50 ml of WR 279396 contains approximately 6 gr paromomycin (15% paromomycin) and 0.2 gr gentamicin (0.5% gentamicin). These amounts are approximately half the total amount of paromomycin injected into adults with normal kidneys (1gr per day for 10 days = 10 g) and 1/10 of the amount of gentamicin injected into adults with normal kidneys (3 mg/kg/day x 10 days = 30 mg/kg for a 66 kg adult = 2.0 grams total). Even if 100% of both aminoglycosides are absorbed into the systemic circulation, which is very unlikely to occur, these amounts of drug are unlikely to give eighth nerve or kidney toxicity. Nevertheless, the possibility of significant systemic side effects will be monitoring by determining renal and 8th nerve function.

Success with this protocol will lead to further phase 3 trials in other *Leishmania* endemic areas of the World. Success in phase 3 trials should make WR279396 the treatment of choice for Old and New World cutaneous leishmaniasis. A treatment regimen consisting of inexpensive topical medication with hopefully modest local toxicity and no systemic toxicity will be extremely attractive for both military and civilian populations.

7.0 EXPERIMENTAL PLAN

7.1 Trial Design

This phase will be a randomized, double-blind trial of WR279396 compared to placebo, each administered twice-a-day for 20 days. All patients will be rescued if clinically required (See. 7.12 "Definition of Responses using clinical criteria") with Glucantime (either intralesional or parenteral) or the primary care physician drug of choice. Maximum treatment delay time for patients who do not show treatment improvement will be 50 days.

7.2 Trial primary endpoint and the secondary endpoint

1. Primary endpoint: Lesions size and clinical response described as re-epithelialization. Lesion size refers to ulcer size only, here, and for the rest of the protocol.

2. Secondary endpoint: Safety and tolerance of WR279396.

7.3 Study Population/Site

The study population will be selected from patients, males and females in Paris and in Tunisia, who are clinically suspected to have cutaneous leishmaniasis obtained in a region of the Old World (in Tunisia, females of child-bearing age will be excluded). Children (≥ 5 years) will be included because they can benefit the most, as a group, from topical agents due to the difficulty to perform intralesional injections in such population. Direct benefits to placebo subjects are: a) placebo effect and cosmetic benefits of treating lesion with the carrier; b) elimination of bacterial infections by keeping the lesion clean and occluded; c) direct access to a medical team that will perform a medical history, physical exam, dermatology exam, and laboratory tests; d) complete parasitology diagnosis. The age of majority in France and Tunisia is 18 years.

7.4 Number and randomization of lesions

One hundred lesions on 100 patients will be studied.

If a patient has more than 1 lesion, all lesions will be treated, but the results of only 1 lesion will be used for efficacy analysis. The lesion chosen for efficacy analysis will be the uppermost, primary ulcerative, parasitologicaly positive lesion on the body (excluding the ears) or, if two lesions are equally uppermost, the left uppermost primary ulcerative lesion.

Lesions will be randomized in the ratio 50 (Group 1: WR 279396): 50 (Group 2: placebo). Patients will be randomized to the two groups and given randomization numbers (Subject's Numbers).

7.5 Inclusion criteria

1. Age: 5 -75 years old (audiograms can not be accurately administered to children under 5 years of age).
2. Lesion character: each lesion must measure ≥ 1 cm and be primarily ulcerative (i.e., not verrucous or nodular)
3. Parasitological diagnosis: have cutaneous leishmaniasis proven parasitologically in lesion selected for inclusion in study.
4. Informed consent: have given written informed consent to participate in the study: (i.e. patient or legal representative).

7.6 Procedures to evaluate inclusion/exclusion criteria

1. Medical history, with particular attention to skin, lymphatic system & mucosal membranes

2. Physical examination
3. Dermatological examination: including measurement of leishmaniasis lesions
4. Urinalysis
5. Complete blood count (WBC, platelets, Hgb)
6. Serum chemistries: creatinine, alanine aminotransferase (ALT) or aspartate aminotransferase (AST), glucose, Na, K.
7. Hearing (8th nerve) tests: high and low tones, Romberg test.
8. Blood pregnancy test (Paris only)

7.7 Exclusion criteria

1. Drug intolerance: history of known or suspected hypersensitivity or idiosyncratic reactions to aminoglycosides in the patient or immediate family members.
2. Previous use of antileishmanial drugs (within 3 months) or present use of routinely nephrotoxic or ototoxic drugs.
3. Previous confirmed leishmaniasis.
4. Patients with tuberculosis under treatment.
5. Potential for follow up: Have less than 7 months time remaining in present address and/or plans to leave the area for more than 30 days.
6. Extent of disease: More than 5 lesions or lesion ≥ 5 cm or a lesion less than 5 cm from the eye, or a lesion in the face that in the opinion of the attending dermatologist could potentially cause significant disfigurement.
7. Location of disease: mucosal involvement.
8. Disseminated disease: clinically significant lymphadenitis with nodules that are painful and > 1 cm in size in the lymphatic drainage of the ulcer.
9. Concomitant medical problems: significant medical problems of the kidney or liver as determined by history and by the following laboratory studies:

   Kidney: clinically significant abnormalities of urine analysis, serum levels of Creatinine, BUN, total proteins > upper limit of normal for the laboratory.

   Liver: AST or ALT > upper limit of normal for the laboratory

   General: glucose, Na, K, > upper limit of normal or < lower limit of normal for the laboratory. Volunteers in whom these normal laboratory values are exceeded by less than 25% will not be automatically excluded. These volunteers will be evaluated on the basis of history, physical, as well as laboratory values.
10. Hearing and Romberg tests: abnormality
11. Schedule or ongoing pregnancy as determined by clinical and/or biological criteria.
12. Females of child-bearing age (Tunisia ONLY).
13. Presence of signs or symptoms of peripheral neuropathy, myasthenia gravis or neuromuscular block

7.8 Duration of Study

Each patient will be treated for 20 days, and be followed for 6 further months.

We anticipate that 100 lesions will be entered in one year. Therefore, the study will require approximately 12 months for patient entry and a further 6 months for patient follow up (approximately 18 months total).

7.9 Dosages and Administration of Drugs

Test articles to be used are:
-WR279396 (paromomycin sulfate 15%, gentamicin sulfate 0.5%, water to dissolve the aminoglycosides, AQIC base)
-placebo (water, AQIC base).

These topical preparations were prepared by Dr. Flanagan, University of Iowa, and distributed in 40-ml jars. The topicals will be stored at 2–8°C at the Institut Pasteur Medical Center, 25-28 Rue du Dr. Roux, 75015, Paris, France and at the Institut Pasteur in Tunis.

The method of administration of topical will be as per SOP. In brief, the lesion shall be cleaned with soap and water. Then 0.0005 ml of formulation shall be applied per 1 mm2 of lesion, twice-a-day for 20 days. Twice a day shall mean that the second administration occurs between 6 and 12 hrs after the first administration. In Tunisia, the application of formulation shall be by medical personnel who will visit the volunteer. In Paris, the application of formulation shall be by medical personnel (first application of the day) and by the patient or parent/guardian, in case of a child, (second application of the day or holidays). Only medical personnel shall record that the formulation was administered. The lesion/medication will be permanently covered with a polyurethan dressing (Tegaderm) and undisturbed (not wiped off, not wetted) for 4 hr after each application.

Iso-enzyme analysis of the parasite isolated from the lesion will be completed after treatment has been started. If the iso-enzyme analysis is *L. aethiopica*, the volunteer will be referred to his primary health care provider for the appropriate standard treatment (probably Glucantime). Should the volunteer elect to receive another treatment, the volunteer will be dropped from the study at the start of glucantime or other antileishmanial drug. Since no *L. aethiopica* has been reported in France or Tunisia in the last decade, the likelihood of a volunteer being dropped for this reason is remote.

7.10 Determination of adverse reactions

7.10.1 Determination of side effects

On each day on which topical is administered, patients will be observed/questioned for the occurrence of the following local side effects:

-pain by history	(none, mild, moderate, or severe)
-erythema	(none, mild=barely perceptible, moderate=well defined, severe=very red)

-edema	(none, mild=barely perceptible, moderate=well defined, severe=raised >2mm; life threatening = exfoliative)
-vertigo	(mild, moderate, or severe)
-tinnitus	(mild, moderate, or severe)
-diminished hearing	(mild, moderate, severe or profound)

7.10.2 Determination of laboratory evidence of side effects

-Blood will be drawn to re-determine creatinine on days 10 and 20.
-Hearing and Romberg tests will be repeated on days 10 and 20.

7.11 Establishment of diagnosis

1. Each lesion to be evaluated for efficacy will be aspirated and/or scraped and/or biopsied.
2. Proof of infection will include the demonstration of motile promastigotes in aspirate cultures or microscopic identification of *Leishmania* amastigotes (by DifQuiK or Giemsa staining) in material obtained from lesions.

7.12 Definition of responses using clinical criteria

Lesions will be measured in two perpendicular directions, and photographs will be taken, prior to therapy, at the end of therapy, and at 30 days, 80 days, and 6 months after the end of therapy. Physically identifying features if present will be hidden in photographs. Patients will be identified by initials and not by name.

1. Cure:	100% reepitheliazation without relapse by 6 months.
2. Clinical improvement:	50% reepitheliazation compared to the pre-treatment lesion size at 30 days (± 7 days) after the end of treatment.
3. Clinical failure:	a. Lack of clinical improvement at 30 days (± 7 days) after the end of treatment.<br>b. Relapse that is enlargement of the lesion compared to the previous measurement at any time after 30 days (± 7 days) post treatment.<br>c. Not demonstrating complete clinical response by 6 months.

7.13 Parasitological results

1. Parasitologic cure: inability to culture or stain for parasites.
2. Parasitologic failure: presence of culturable or stainable parasites.

7.14 Definition of cure

Determination of cure will be based on clinical criteria. A lesion (ulcer) will be defined as cured if it has undergone a complete clinical response.

7.15 Criteria for Early Withdrawal from the Study

Any one of the following criteria will be considered sufficient to cause the withdrawal of the patient from the study.

1. Adverse effects (particularly those listed in section 10.7.2) reported by the patient or medical personnel that in the opinion of the principal investigator pose a serious risk to the patient.
2. Clinical failure.

Patients who require re-treatment for failure or relapse will be removed from the protocol and treated with the standard of care, probably 0.5 – 3.0 ml of Glucantime intralesionaly once per week for a maximum of 5 weeks or in case of multiple lesions (n≥4) Glucantime at 20 mg SbV/kg /day for a maximum of 20 days) or the attending physician drug of choice.

7.16 Pharmacokinetics evaluation

The purpose of determining blood and urine levels will be to evaluate if measurable quantities of aminoglycosides are being absorbed. Blood samples (5 ml) will be obtained on days –1 (control), 10 and 20 as per SOP. On days 10 and 20, blood will be obtained both just prior to the application of drug ("trough"). and 1 hr after drug application ("peak"). Plasma will be frozen for future analysis and shipped to the analytic site as per SOP. A 24 hr urine collection will be performed on days 9 to10 and 19 to 20 to determine total absorbed drug and compared to day -1 baseline (n=4 0.5 – 1.0 ml samples shipped to the U.S.).

7.17 Concomitant medications

If concomitant medical problems occur, medications that are possibly effective against cutaneous leishmaniasis (antimony, amphotericin B, pentamidine, injectable paromomycin, fluconazole, ketoconazole, allopurinol) will not be used. If these agents must be used, the patient will be removed from the protocol.

7.18 Departure from the protocol

Additional tests/procedures may be performed for clinical management at the discretion of the attending physician.

Notification that additional tests/procedures are being performed or of desired protocol amendments will be sent to the responsible IRB(s), Clinical Monitor, Topical Antileishmanial Project Officer, and USAMMDA. In addition, departures from the approved protocol will be noted and reported to the HSRRB in a timely manner.

Changes to the approved protocol will be approved by both the local IRB and the HSRRB prior to implementation. Changes intended to eliminate an apparent immediate hazard to a subject will be immediately implemented provided the FDA and reviewing IRBs are notified.

7.19 Procedural timetable

Procedure	Therapy Period (days)				Follow-up (Days/months)#		
	-1	1	10	20	30	80	6 Months
**Informed Consent**	X						
**Demographic Details**	X						
**Medical History**	X						
**History of Leishmaniasis**	X						
**Physical Exam**	X						
**Dermatology Exam**	X						
**Hearing Test**	X		X	X			
**Romberg Test**	X		X	X			
**CBC** (WBC,platelets, Hgb)	X						
**AST or ALT**	X						
**Serum Creatinine**	X		X	X			
**Glucose, Na, K**	X						
**Blood pregnancy Test < 72 hours before D0**	X						
**Urinalysis**	X			X			
**Pharmacokinetics Blood**	X		X	X			
**Pharmacokinetics Urine**	X		X**	X**			
**Measure ulcer**	X			X	X	X	X
**Clinical Evaluation of Lesion**	X			X	X	X	X
**Photograph Lesion**	X			X	X	X	X
**Parasitologic Test**	X			X*			
**Entrance Exam and Lab Test Check List**	X						

**Drug Therapy**	Days 1 through 20
**Local Toxicity**	Days 1 through 20

* Note: Perform only on unhealed designated test lesion.
** A 24-hour urine collection will be performed on Days 9 to 10 and 19 to 20.
# Follow-up refers to time after end of therapy

8.0 SAMPLE SIZE AND DATA ANALYSIS

8.1 Sample size calculation:

If the placebo cure rate is assumed to be 35%, and we desire at least a 30% improvement in the cure rate due to WR279396, the cure rate with WR279396 has to be at least 65%. For alpha=0.05 (2-sided) and beta=0.2, 50 placebo lesions plus 50 WR279396 lesions (100 lesions total) are required for 1:1 allocation.

8.2 Efficacy calculation:

The primary criterion for treatment evaluation will be the percentage of lesions that demonstrate complete clinical responses and lesions time to heal. The response rate for the WR 279396 group will be compared with that of the placebo group by chi-square test.

8.3 Treatment randomization codes:

Each center (site) will be randomized by block randomization. The randomization list will be generated by the Division of Experimental Therapeutics and kept in a seal envelope. Only in a case of a medical emergency when it is necessary to know if the volunteer was treated with WR279396 or placebo the code will be broken. The result will be informed by phone to the attending physician.

8.4 Missing or unused data:

The repetitive nature of the Case Report Form makes it very hard to have missing data. The Case Report Form was designed that way in purpose. Check lists are common throughout the protocol. Nevertheless, if data is missing the local PI will try his best to obtain the missing data. Unused data will not be published. If a subject is lost to follow-up prior to the three-month visit (D100), the data will not be included in the analysis. If a subject is lost between the third and the six-month follow-up the PIs will have the option to use data with remarks.

9.0 DIRECT ACCESS TO SOURCE DATA/DOCUMENTS

The investigators, medical monitor, members of the Walter Reed Army Institute of Research Human Use Committee, representatives of the U.S. Army Medical Research and Materiel Command, the U. S. Food and Drug Agency, members of the Institut Pasteur in Paris and Tunisia, the French and Tunisian Minister of Health, and other government agencies both U. S. French and Tunisian as part of their duties, may access study data to monitor, audit, IRB review and pursue regulatory inspections of this trial's source data and documents as part of their responsibility to protect human subjects in research.

10.0 QUALITY CONTROL AND QUALITY ASSURANCE

USAMMDA's Quality Assurance (QA) section or a professional contract research organization's QA unit will provide in-country quality control and quality assurance in accordance with good clinical practices.

11.0 ETHICS

The risks to normal volunteers associated with participation in this study are relatively low, whereas the potential benefit to society in the successful development of an effective topical treatment for cutaneous leishmaniasis skin is high. Therefore, on balance, the study stands on solid ethical basis. The study will be explained to pediatric subjects in language they can understand to assure that they are cooperative and willing to participate in the protocol.

The conduct of this trial will follow the Helsinki Accords, FDA good clinical practices, U. S. Army Surgeon General regulations concerning clinical trials and French/Tunisian Ministry of Health policies regarding clinical trials and regulations.

Progress on this protocol will be provided to the Human Use Review Committee annually and as appropriate during the study.

12.0 DATA HANDLING AND RECORDKEEPING

A copy of the protocol, CRF(s), other source documents and regulatory records will be kept on file by the U.S. Army Medical Research and Materiel Command for 2 years after the marketing application has been approved or if no application is to be filed or if the application was not approved for such indication for 2 years after the investigation was discontinued and the FDA is notified. As a Co-sponsor, and according to the French Law, copies of the CRF(s) other source documents and regulatory records will be kept on file at the Institut Pasteur for 15 years after the investigation is discontinued.

13.0 FINANCING

The financing of this trial is to come from USAMMDA, Fort Detrick, MD through the Product Manager.

14.0 PUBLICATION POLICY

If journal articles are to be written then no volunteer will have their name or initials released, they will be identified by their volunteer number. Some of the photos may be used. It is anticipated that the results of this study will be presented to the scientific community via oral presentations at meetings and written publications in scientific journals.

15.0 VOLUNTEER REGISTRY DATA BASE REQUIREMENTS

It is the policy of the U. S. Army Medical Research and Materiel Command that data sheets (USAMRDC Form 60-R) are to be completed on all volunteers participating in research for entry into this Command's Volunteer Registry Database. The information to be entered into this Subject confidential database includes your name, address, study number, study name, and dates. The intent of the database is two-fold: first, to readily answer questions concerning an individual's participation in research sponsored by USAMRMC; and second, to ensure that USAMRMC can exercise its obligation to ensure research volunteers are adequately warned (duty to warn) of risks

and to provide new information as it becomes available. The information will be stored at USAMRMC for a minimum of 75 years. For France and Tunisia the U.S. Army Medical Research and Materiel Command will go through the Institute Pasteur in Paris and Tunis to contact the subject if necessary, as the French law and Tunisian law prohibits the release of Social Security Numbers.

16.0 ASSESSMENT OF SAFETY

16.1 Safety Parameters / Adverse Events:

The recording of adverse events is the responsibility of the investigators. Volunteers will be instructed to contact the investigators immediately in the event they develop any unusual signs or symptoms post treatment. Follow-up information will be provided for all initial reports of serious and unexpected adverse events.

16.1.1 Adverse Event

An adverse event temporally related to participation in the study should be documented, whether or not it is considered to be related to the test article. This definition includes intercurrent illnesses and injuries, or exacerbation of preexisting conditions. The following will be included in all IND safety reports: Subject identification number and initials; investigator's name and name of MTF; subject's date of birth, gender, and ethnicity; test article and dates of administration; signs/symptoms and severity; date of onset; date of resolution or death; relationship to the study drug; action taken; concomitant medication(s), including dose, route and duration of treatment, date of last dose.

16.1.2 Serious Adverse Event

A serious adverse event is any untoward medical occurrence that at any dose:

* results in death
* is life-threatening [Life threatening event is an event which presents the risk of death at the time of the event; it does not refer to an event which hypothetically might have caused death had it been more severe.]
* requires inpatient hospitalization
* results in persistent or significant disability/incapacity [Disabling/Incapacitating adverse event is any event, which may result in a substantial disruption of the volunteer's ability to carry out normal life functions. This definition is not intended to include minor cases of headache, nausea, vomiting, diarrhea, influenza, rhinorrhea, lacrimation or accidental trauma, such as a sprained ankle.]

- Important medical events that may not result in death, may not be life-threatening or require hospitalization may be considered serious when, based on the medical judgement of the investigator, they may adversely affect the subject and may require medical/surgical intervention (i.e. an allergic reaction resulting in bronchospasm requiring immediate intervention without requiring hospitalization).
-

16.2 Recording and Reporting Adverse Events:

16.2.1 Documenting Adverse Events

At the time of each visit all adverse events either observed or reported, will be documented in the CRF and in the subject's medical records when available. The investigator and clinical monitor team will evaluate each adverse event. In the event a medical diagnosis is made, the event will be reported as an AE or an SAE. If no medical diagnosis is made, individual data will be documented as the AE/SAE in the CRF. Details of any therapeutic measures taken in the event of AE/SAE will be recorded. Adverse events previously documented in the CRF will be recorded as 'ongoing', 'improved' or 'resolved' at subsequent visits. If an adverse event changes, or advances in quantity or quality, a new record of the event will be initiated.

16.2.2 Reporting Adverse Events

Reporting of serious and unexpected adverse events: Serious and unexpected adverse experiences will be immediately reported by telephone to the USAMRMC Deputy for Regulatory Compliance and Quality at 301-619-2165 during duty hours; during non-duty hours the number to call is 301-619-2165 and information will be sent by facsimile to 301-619-7803) A written report will follow the initial telephone call within 3 working days. The written report will be addressed to the U.S. Army Medical Research and Materiel Command, ATTN: MCMR-RCQ, 504 Scott Street, Fort Detrick, Maryland 21702-5012. In addition, serious and unexpected adverse experiences will be immediately reported by telephone to the Clinical Monitor Coordinator, Dr. Jonathan D. Berman (301) 594-7105, and the study Principal Investigator Dr. Max Grogl (301) 319-9359.

Routine reporting: will apply to local and non-serious systemic events.

16.2.3 Follow-Up of Adverse Events

The investigator will determine causality of the AE/SAE. This may include additional laboratory testing, follow up visits and/or histopathological examinations. All serious adverse events will be followed until resolution or SAE becomes stable. Non-serious adverse events will be followed until study conclusion. Reports relative to the subsequent course of an adverse event noted for any volunteer will be submitted to the local Medical Monitor and the study Clinical Monitor Coordinator.

16.3 Study Specific Definitions and Exceptions:

Any hospitalization following initiation of the study for an elective procedure or therapy will be reported as a 'hospitalization (Not adverse event)' on the CRF. If this hospitalization is planned prior to study participation or arises from a pre-existing condition, it will be recorded on the medical history form and the CRF. For study specific definitions side effects and grades see Appendix D.

17.0 ROLES AND RESPOSIBILITIES OF THE STUDY PERSONEL

Principal Investigator: Signs FDA Form 1572. Responsible for protocol adherence and execution, passage of protocol through U.S. protocol committees, tie-breaking blinded assessment of lesion changes in patients, data integrity, applying for and receiving approval for any modifications to the protocol or consent form in the U.S., training of associate investigators, ensuring safety of the volunteers, and reporting of any adverse events. Assures overall coordination of the study.

Associate Investigators: Responsible for protocol adherence and execution, passage of protocol through French and Tunisian protocol committees, tie-breaking blinded assessment of lesion changes in patients, data integrity, applying for and receiving approval for any modifications to the protocol or consent form, ensuring safety of the volunteers, managing and reporting of any adverse events, briefing potential volunteers, obtaining proper informed consent, determining study eligibility based on screening data and the exclusion criteria, and recording all observations and data in the individual subject records.

Medical Monitors: Responsible to provide medical care and monitoring of research subjects for conditions that may arise during the conduct of the study. The medical monitor will review all serious and unexpected adverse events (per ICH definitions) associated with the protocol and provide an unbiased written report of the event within 10 calendar days of the initial report. At a minimum, the medical monitor will comment on the outcomes of the adverse event (AE) and relationship of the AE to the test article. The medical monitor will also indicate whether he/she concurs with the details of the report provided by the study investigator.

18.0 REFERENCES

1. Berman JD. 1988. Leishmaniasis Chemotherapy: biochemical mechanisms, clinical efficacy, and future strategies. Rev Infect Dis 10:560-586.

2. Soto- Mancipe J, Grogl M, Berman JD. 1993. Evaluation of pentamidine for the treatment of cutaneous leishmaniasis in Colombia. Clin Infect Dis 16: 417-425.

3. Asilian A, Jalayer T. 1995. A randomized placebo-controlled trial of a two-week regimen of aminosidine (paromomycin) ointment for treatment of cutaneous leishmaniasis in Iran. Am J Trop Med Hyg 53(6): 648-651.

4. Ben Salah A, Zakraoui H. 1995. A randomized placebo-controlled trial in Tunisia treating cutaneous leishmaniasis with paromomycin ointment. Am J Trop Med Hyg 53(2): 162-166.

5. Soto J, Grogl M, Berman J, Olliaro P. 1994. Modest efficacy of injectable aminosidine for single-agent therapy for Colombian cutaneous leishmaniasis. Trans Roy Soc Trop Med Hyg 88: 695-698.

6. Harms G, Chehade AK. 1991. A randomized trial comparing a pentavalent antimonial drug and recombinant interferon-gamma in the local treatment of cutaneous leishmaniasis. Trans R Soc Trop Med Hyg 85(2): 214-216.

7. Bellazoug S, Neal RA. 1986. Failure of meglumine antimoniate to cure lesions due to *L. major* in Algeria. Trans R Soc Trop Med Hyg 80(4): 670-671.

8. Herwaldt BL, Berman JD. 1992. Recommendations for treating leishmaniasis with sodium stibogluconate (Pentostam) and review of pertinent clinical studies. Am J Trop Med Hyg 46: 296-306.

9. Arana BA, Navin TR, Arana FE, Berman JD, Rosenkaimer F. 1994. Efficacy of short-course (10 days), high-dose meglumine antimonate with or without interferon-gamma in treating cutaneous leishmaniasis in Guatemala. Clin Infect Dis 18:381-384.

10. Jeannel D, Tupin P. 1989. Leishmaniasis in France. Lancet 8666: 804.

11. Caumes E, Carriere J. 1995. Dermatosis associated with travel to tropical countries: a prospective study of the diagnosis and management of 269 patients presenting to a Tropical Disease Unit. Clin Infect Dis 20(3):542-548.

12. Sharifi I, Fekri R. 1998. Bull World Health Organ 76(3): 289-293.

13. El-On J, Livshin R, Evan-Paz Z, Hamburger D, Weinrauch L. 1986. Topical treatment of cutaneous leishmaniasis. J Invest Dermatol 87:284-8.

14. El-On J, Greenblatt CL. An in vitro model for testing the effect of anti-leishmanial drugs of possible use in topical treatment. 1983. Current Therapeutic Research 33:660-669.

15. El-On J, Messer G. Leishmania major: antileishmanial activity of methylbenzethonium chloride. 1986. Am J Trop Med Hyg 35: 1110-1116.

16. Soto J, Hernandez N, Mejia H, Grogl M, Berman J. 1995. Successful treatment of American CL with a combination of topical paromomycin/MBCl and injectable Glucantime. Clin Infect Dis 20: 47-51.

17. Glaza SM. 1995. Dermal sensitization study of WR 279396, an antileishmanial topical cream in Guinea Pigs-closed patch technique. HWI #40501111.

18. Glaza SM. Primary dermal photo activated irritation study of WR 279396, an antileishmanial topical cream in rabbits. HWI # 40501110.

19. Glaza SM. 1995. Photoallergic contact dermatitis study of WR 279296, an antileishmanial topical cream in Guinea Pigs (Armstrong Method). HWI # 40501112.

20. Levine BS. 1995. Four Week dermal toxicity of study of WR 279396 in CD Rats. [Verbal communication from Dr. Levine; written confirmation to be sent 23 June 95.]

21. Aminosidine [paromomycin] Product Profile. Undated. Farmitalia Carlo Erba. Milan, Italy.

22. Wilfert JN, Burke JP, Bloomer HA, Smith CB. 1971.Renal insufficiency associated with gentamicin therapy. J Infect Dis 124: S148-S153.

23. Anonymous. 1993. Humatin. Physicians Desk Reference: 1776.

24. El-On J, Halevy S, Grunwald MH, Weinrauch L. 1992. Topical treatment of Old World cutaneous leishmaniasis caused by Leishmania major: a double blind control study. J Amer Acad Dermatol 27:227-31.

25. El-Safi SH, Murphy AG, Bryceson ADM, Neal RA. 1990. A double-blind clinical trial of the treatment of cutaneous leishmaniasis with paromomycin ointment. Trans R Soc Trop Med Hyg 84: 690-691.

26. Anonymous. 1993. Garamycin. Physicians Desk Reference: 2187-2190.

27. Sande MA, Mandell GL. 1983. The Aminoglycosides. in Gilman AG, Goodman LS, Rall TW, Murad F Eds. The Pharmacological basis of Therapeutics. 7th Edition. McMillan Publ Co. NY. Chapter 51.

28. Jackson GG, Arcieri G. 1971. Ototoxicity of gentamicin in man: a survey and controlled analysis of clinical experience in the United States. J Infect Dis 124: S130-S137.

29. Stone HH, Kolb LD, Pettit J, Smith RB. 1968. The systemic absorption of an antibiotic from the burn wound surface. American Surgeon 34: 639-643.

30. Dayal VA, Smith EL, McCain G. 1974. Cochlear and vestibular gentamicin toxicity. Arch Otolaryngol 100: 338-340.

31. Parnes LS, Riddell, D. 1993. Irritative spontaneous nystagmus following intratympanic gentamicin for Meniere's disease. Laryntoscole 103 745-749

32. Campochiaro PA, Lim JI. 1994. Aminoglycoside toxicity in the treatment of endophthalmitis. Arch Ophthalmolo 112: 48-53.

33. Bonner M. 1996. Irritant and phototoxicity reactions to the topical antileishmanial WR 279396: a randomized, double-blind phase I study. WRAIR.

34. Franke E, et al. 1990. Efficacy and toxicity of sodium stibogluconate for mucosal leishmaniasis. Ann Int Med 113: 934-940.

35. Alrajhi A, et al. 2002. Fluconazole for the treatment of cutaneous leishmaniasis caused by Leishmania major. N Engl J Med 346(12): 891895.

U.S.S.P.

WRAIR [illegible]: A Phase 2 Study – Old World

19.0 SIGNATURE OF INVESTIGATORS AND MEDICAL MONITOR

I, the undersigned, have reviewed this protocol, including Appendices, and I will conduct the study as described and will adhere to the Ethical and Regulatory Considerations delineated herein. I was informed on the principles and requirements of Good Clinical Practices.

19.1 PRINCIPAL INVESTIGATOR:

Max Grogl, LTC, MS, Ph.D. Date: 10/12/02

19.2 CO-INVESTIGATORS:

Pierre Buffet, MD Date: 03/12/02

Afif Ben Salah, MD Date: 3/12/02

19.3 STUDY MONITOR:

Jonathan D. Berman, COL, MC Date: 10/12/02

19.4 MEDICAL MONITORS:

Dr. [illegible], MD Date: 5/12/2002

Dr. [illegible] Louzir, MD Date: 8/12/2002

19.0 SIGNATURE OF INVESTIGATORS AND MEDICAL MONITOR

I, the undersigned, have reviewed this protocol, including Appendices, and I will conduct the study as describe and will adhere to the Ethical and Regulatory Considerations delineated herein. I was informed on the principles and requirements of Good Clinical Practices.

19.1 PRINCIPAL INVESTIGATOR:

_________________________ Date: _____________

Max Grogl, LTC, MS, Ph.D.

19.2 CO-INVESTIGATORS:

_________________________ Date: _____________

Pierre Buffet, MD

_________________________ Date: _____________

Afid Ben Salah, MD

19.3 STUDY MONITOR:

_________________________ Date: _____________

Jonathan D. Berman, COL, MC

19.4 MEDICAL MONITORS:

_________________________ Date: _____________

Dr. Odile Launay, MD

_________________________ Date: _____________

Dr. Hechmi Louzir, MD

Appendix D (Adverse or Unexpected Event Determination – Side Effects and Grades)

1. Cutaneous adverse reactions [grade erythema separately from edema]

Grade 0: no reaction (no erythema or edema)
Grade 1: mild reaction (barely perceptible erythema or edema)
Grade 2: moderate reaction (well defined erythema or edema)
Grade 3: Severe reaction (very red erythema with raised > 2mm edema)
Grade 4: life threatening reaction (exfoliative dermatitis)

NOTE: Grade 3 reactions require immediate notification to Medical Monitor.
Grade 4 reactions require immediate termination of treatment and ADVERSE EVENT NOTIFICATION as per Appendix 3.

2. Abnormal clinical laboratory values (DAIDS/NIH scale):

Parameter(units)	Grade 1	Grade 2	Grade 3	Grade 4
Creatinine (xULN)	>1-1.5	>1.5-3	>3-6	>6
AST/ALT (XULN)	1.25-2.5	>2.5-5	>5-10	>10
WBC (1,000/mm3)	1000-1500	750-999	500-749	<500
PLT (1,000/mm3)	75-99	50-74	20-49	<20
HGB (g/dL)	8-9.4	7-7.9	6.5-6.9	<6.5

NOTE: Grade 2 require immediate notification to Medical Monitor.
Grade 3 and 4 reactions require immediate termination of treatment.
Grade 2, 3 and 4 laboratory values require evaluation of the volunteer. If the laboratory abnormality reflects clinically significant pathology, an ADVERSE EVENT report will be fill out and notification will occur as per Appendix 3.

3. Miscellaneous (associated with drug administration):

Pain:
Grade 0: none (feels no pain)
Grade 1: mild pain (does not interfere with daily activity)
Grade 2: moderate pain (interferes with daily activity)
Grade 3: severe pain (daily activities are interrupted)

NOTE: Grade 2 reactions require immediate notification to Medical Monitor.
Grade 3 reactions require immediate termination of treatment and ADVERSE EVENT NOTIFICATION as per Appendix 3.

Vertigo:
Grade 0: none (feels no vertigo)
Grade 1: mild vertigo (does not interfere with daily activities)
Grade 2: moderate vertigo (interferes with daily activity)
Grade 3: severe vertigo (daily activities are interrupted)

NOTE: Grade 2 reactions require immediate notification to Medical Monitor.
Grade 3 reactions require immediate termination of treatment and ADVERSE EVENT NOTIFICATION as per Appendix 3.

Tinnitus:
Grade 0: none (feels no ringing/buzzing/roaring etc.)
Grade 1: mild tinnitus (does not interfere with daily activities)
Grade 2: moderate tinnitus (interferes with daily activities)
Grade 3: severe tinnitus (daily activities are interrupted)

NOTE: Grade 2 reactions require immediate notification to Medical Monitor.
Grade 3 reactions require immediate termination of treatment and ADVERSE EVENT NOTIFICATION as per Appendix 3.

Diminished hearing:
Grade 0: none (stays within normal limits) Normal = -10 db to 25 db
Grade 1: mild hearing loss (26 db - 40 db)
Grade 2: moderate hearing loss (41 db - 55 db)
Grade 3: severe hearing loss (56 db - 80 db)
Grade 4: profound hearing loss (> 80 db)

NOTE: Grade 2 reactions require immediate notification to Medical Monitor.
Grade 3 and 4 reactions require immediate termination of treatment and ADVERSE EVENT NOTIFICATION as per Appendix 3.

4. Reporting of adverse events:

Serious and unexpected adverse experiences will be immediately reported by telephone to the USAMRMC Deputy for Regulatory Compliance and Quality (301-619-2165) (non-duty hours call 301-619-2165 and send information by facsimile to 301-619-7803). A written report will follow the initial telephone call within 3 working days. Address the written report to the U.S. Army Medical Research and Materiel Command, ATTN: MCMR-RCQ, 504 Scott Street, Fort Detrick, Maryland 21702-5012. Written follow-up information and the medical monitor's written review and assessment of the adverse event will be provided to the HSRRB.

(PLEASE SEE APPENDIX 3 FOR MORE DETAILS)

TITLE: Topical Treatment of Cutaneous Leishmaniasis with WR 279396: A Phase 2 Study in the Old World

Appendixes

Appendix A -1:

++

VOLUNTEER AGREEMENT AFFIDAVIT

For use of this form, see AR 70-25 or AR 40-38; the proponent agency is the Office of the Surgeon General (OTSG)

PRIVACY ACT OF 1974

Authority: 10 USC 3013, 44 USC 3101, and 10 USC 1071-1087

Principal Purpose: To document voluntary participation in the Clinical Investigation and Research Program. Study number and home address will be used for identification and locating purposes.

Routine Uses: **The study number** and home address will be used for identification and locating purposes. Information derived from the study will be used to document the study; implementation of medical programs; adjudication of claims; and for the mandatory reporting of medical conditions as required by law. Information may be furnished to Federal, State, and local agencies.

Disclosure: **The furnishing of your study number** and home address is mandatory and necessary to provide identification and to contact you if future information indicates that your health may be adversely affected. Failure to provide the information may preclude your voluntary participation in this investigational study.

PART A - VOLUNTEER AFFIDAVIT

Volunteer Subjects in Approved Department of the Army Research Studies

Volunteers under the provisions of AR 40-38 and AR 70-25 are authorized all necessary medical care for injury or disease which is the proximate result of their participation in such studies.

I, ______________________________, having full capacity to consent, and having attained my ______________ birthday, do hereby volunteer/ give consent as legal representative for ______________________ to participate in a research study entitled " TOPICAL TREATMENT OF CUTANEOUS LEISHMANIASIS WITH WR 279396: A PHASE II STUDY IN THE OLD WORLD" under the direction of Dr. Pierre Buffet and Dr. Afif Ben Salah.

Subject Initials _______ Subject Number ____________ Witness Initials _______

The implications of my voluntary participation/consent as legal representative; the duration and the purpose of the research study; the methods and means by which it is to be conducted; and the inconveniences and hazards that may reasonably be expected have been explained to me by Dr. Pierre Buffet, Dr. Afif Ben Salah or ________________________________, (Name of the other local investigator) at the Institut Pasteur Medical Center in Paris, or at the Institut Pasteur in Tunisia.

I have been given an opportunity to ask questions concerning this investigational study. Any such questions were answered to my full and complete satisfaction. Should any further questions arise concerning my rights or study-related injury, I may contact:

Dr. Pierre Buffet — Call collect: 33-1-40613817
FAX: 33-1-45688218

or

Dr. Afif Ben Salah — Call collect: 216-1-792-429
FAX: 216-1-791-833

or

Dr. Max Grogl — Call collect: 301-319-9359
FAX: 301-319-9180

I understand that I may at anytime during the course of the study revoke my consent and withdraw/have the person I represent withdrawn from the study without further penalty or loss of benefits. However, I/the person I represent may be requested to undergo certain examinations if, in the opinion of the attending physician, such examinations are necessary for my/the person I represent health or well being. My/the person I represent refusal to participate will involve no penalty or loss of benefits to which I am/the person I represent is otherwise entitled.

Note: The French laws do not require witness initials in the Consent Form. However, because the study is in collaboration with the U.S. Army the use of witness initials is authorized.

Volunteer's signature / Signature of legal guardian: ________________________________

Date: ________________________________

Volunteer's printed name / legal guardian: ________________________________

Subject Initials ______ Subject Number ____________ Witness Initials ______

Address: ____

I was present during the explanation referred to above, can attest to the volunteer's opportunity to ask questions, and hereby witness the volunteer's signature.

Witness's signature: ______________________________

Date: ________________

Witness's printed name: ____________________________

Investigator's/Subinvestigator's signature: ____________________________

Date: ________________

Investigator's/Subinvestigator's printed name: ____________________

Individual Conducting the ICF Discussion
(if it is NOT the Investigator) signature: ____________________________

Date: ________________

Individual Conducting the ICF Discussion
(if it is NOT the Investigator) printed name: ____________________________

Subject Initials ______ Subject Number ___________ Witness Initials ______

TITRE : "Traitement topique de la leishmaniose cutanée au moyen du WR 279396 : Etude de Phase 2 dans l'Ancien Monde".

ANNEXES

Appendix A - 2

ACCORD DE VOLONTARIAT PAR DECLARATION SOLENNELLE

Pour l'utilisation de ce formulaire, voir AR 70-25 ou AR 40-38, l'agence auteur de la proposition est l'OTSG

LOI SUR LES ATTEINTES A LA VIE PRIVEE DE 1974

Autorité : 10 USC 3013, 44 USC 3101 et USC 1071-1087

Objet principal : Documenter la participation volontaire aux Investigations Cliniques et au Programme de Recherche. Le numéro de Code patient et l'adresse du domicile seront utilisés à des fins d'identification et de localisation.

Usages habituels : **Le numéro de code patient** et l'adresse du domicile seront utilisés pour l'identification et la localisation. Les informations tirées de cette étude seront utilisées pour documenter l'étude, pour la réalisation de programmes médicaux, des décisions en matière de contentieux, et pour la déclaration obligatoire de situation médicale exigée par la loi. Les informations peuvent être communiquées aux agences fédérales, étatiques et locales.

Informations : **La communication de votre numéro de code patient** et de l'adresse de votre domicile est obligatoire et nécessaire afin de vous identifier et de rentrer en contact avec vous si des données ultérieures indiquent que votre santé peut être affectée de manière néfaste. La non-communication de ces informations risque de compromettre votre participation volontaire à cette étude d'investigation.

Initiales du Sujet ________ Numéro du Sujet _______ Initiales des Témoins ______

PARTIE A – DECLARATION SOLENNELLE DU VOLONTAIRE

Les sujets volontaires dans le Département Agréé des Etudes de Recherches de l'Armée

Les volontaires qui relèvent des dispositions de AR 40-38 et AR 70-25 sont admis à bénéficier de tous les soins médicaux nécessaires pour toute lésion ou maladie qui pourrait être le résultat étroit de leur participation à ces études.

Je soussigné, --, ayant la pleine capacité légale de donner mon consentement, et ayant atteint l'âge de ----------------------------, déclare être volontaire / donne mon consentement en ma qualité de représentant légal de ---------------------------------- pour participer à une étude de recherche intitulée "TRAITEMENT TOPIQUE DE LA LEISHMANIOSE CUTANEE AU MOYEN DU WR 279396 : ETUDE DE PHASE 2 DANS L'ANCIEN MONDE", sous la direction du docteur Pierre BUFFET et du docteur Afif BEN SALAH.

Les implications de ma participation / mon consentement volontaire en tant que représentant légal, la durée et l'objet de l'étude de recherche, les méthodes et les moyens qui seront utilisés pour la conduite de cette étude, et les désagréments et les risques qui peuvent raisonnablement être envisagés m'ont été expliqués par le docteur Pierre BUFFET, le docteur Afif BEN SALAH ou -------------------------------------- (nom de l'autre investigateur local) au Centre Médical de l'Institut Pasteur à Paris ou à l'Institut Pasteur en Tunisie.

On m'a donné l'occasion de poser des questions concernant cette étude d'investigation. Les réponses données à mes questions ont été totalement satisfaisantes. Si d'autres questions concernant mes droits ou concernant d'éventuelles lésions liées à cette étude me viennent à l'esprit ultérieurement, je peux contacter :

Dr. Pierre BUFFET	N° de tél. : 01- 40 61 38 17<br>N° de Fax : 01- 45 68 82 18
Ou	
Dr. Afif BEN SALAH	N° de Tél. : 216-1- 792-429<br>N° de Fax : 213-1- 791-833
Ou	
Dr. Max GROGL	N° de Tél. 00-1-301-319-9359<br>N° de Fax : 00-1-301-319-9180

J'ai été informé que je peux, à tout moment pendant le déroulement de l'étude, revenir sur mon consentement et me retirer / faire retirer la personne que je représente de cette étude sans faire l'objet de sanctions et sans perdre les bénéfices auxquels j'ai droit. Toutefois, je peux / la personne que je représente peut être sollicité(e) pour me soumettre / pour se soumettre à certains examens si, selon le médecin chargé de l'étude, de tels examens sont nécessaires pour

Initiales du Sujet ________ Numéro du Sujet _______ Initiales des Témoins ______

ma santé et mon bien-être / la santé ou le bien-être de la personne que je représente. Mon refus de participer à cette étude / le refus de la personne que je représente de participer à cette étude ne peut faire l'objet d'aucune sanction ni entraîner la perte de bénéfices auxquels j'ai / la personne que je représente a droit.

Remarque : La Loi française ne requiert pas de signature d'un témoin dans le formulaire de consentement. Cependant, dans la mesure où cette étude est réalisée avec l'armée américaine, le recours à la signature d'un témoin est autorisée.

Signature du volontaire / Signature du tuteur légal: --

Date: ----------------------------------

Nom du volontaire / tuteur légal en lettres majuscules: --

Adresse: --

J'étais présent lors de la communication des explications indiquées ci-dessus. Je peux attester que le volontaire a eu la possibilité de poser des questions et j'atteste par la présente la conformité de la signature du volontaire.

Signature du témoin: --

Date: ---

Nom du témoin en lettres majuscules: ---

Signature de l'investigateur / Sous-investigateur : ---

Date: ---

Nom de l'investigateur / Sous-investigateur en lettres majuscules : -------------------------------

Signature de la personne animant la discussion concernant le formulaire de consentement éclairé (AUTRE que l'Investigateur) :---

Date--

Nom de la personne animant la discussion concernant le formulaire de consentement éclairé (AUTRE que l'investigateur) en lettres majuscules :---

Initiales du Sujet ________ Numéro du Sujet _______ Initiales des Témoins ______

PART B - TO BE COMPLETED BY INVESTIGATOR

INSTRUCTIONS FOR ELEMENTS OF INFORMED CONSENT: *(Provide a detailed explanation in accordance with Appendix C, AR 40-38 or AR 70-25.)*

Appendix A –3 (Volunteer Affidative - Part B – Consent Form Explanation in English)

TITLE OF STUDY

TOPICAL TREATMENT OF CUTANEOUS LEISHMANIASIS WITH WR 279396: A PHASE II STUDY IN THE OLD WORLD"

CO-SPONSORS: The Office of the Surgeon General (OTSG) and The Institut Pasteur in Paris.

CO-INVESTIGATORS/PHYSICIANS RESPONSIBLE FOR THE RESEARCH
Dr. Pierre Buffet, Institut Pasteur Medical Center, Paris, Tel: 33-1-4061-3817 Fax : 33-1-4061-3799 and Dr. Afif Ben Salah, Institut Pasteur, Tunisia, Tel: 216-1-792-429, FAX: 216-1-791-833.

PARTICIPATION INFORMATION

You/your child have been diagnosed with cutaneous leishmaniasis. Cutaneous leishmaniasis is caused by parasites which are transmitted by sandfly bites. The parasites invade the cells of the skin, and create an ulcer. Cutaneous leishmaniasis characteristically heals by itself, but self-healing may require several months, and in that time the disease may spread to other parts of the skin. Rarely, the disease may spread to the nose.

The standard treatment is with injections of antimony in the form of meglumine antimonate (Glucantime). Although this drug is generally effective, the disadvantages of antimony therapy are that at least five painful intralesional injections have to be given to the patient, there are some failures and relapses, and that when used intramuscularly there may be toxicity to the heart, liver, and pancreas. The primary purpose of this study is to compare WR 279396 to placebo, and see if a cream put onto the lesion can be as effective as injections of Glucantime. An additional important purpose is to see if the cream is painful, and if it is absorbed into your/your child blood. Since lesions cure by themselves, and we have to know how many cures are specifically due to the cream, this will be a placebo-controlled double-blinded trial. You/your child will be assigned by chance to the active group or the placebo group. This means that 50% of the volunteers will get cream with no active ingredient ("placebo") whereas the other 50% of the volunteers will get the active cream (WR 279396). Neither you/your child nor your doctor will know which cream is which ("double-blinding"). The placebo and the active cream WR 279396 look the same.

Since you/your child have been asked to participate in a medical research study, it is very important that you understand the following general principles which apply to all participants in our studies, whether normal or patient volunteers: (1) Your/your child participation is entirely

Subject Initials ______ Subject Number ______ Witness Initials ______

voluntary. (2) You/your child may withdraw from participation in this study or any part of the study at any time. Refusal to participate will involve no penalty or loss of benefits to which you are otherwise entitled. (3) After you/your child read the explanation, you/your child should ask any questions needed for you/your child to understand clearly the nature of the study.

INTRODUCTION

Researchers at the Walter Reed Army Institute of Research in the United States, have developed a cream which may cure cutaneous leishmaniasis. This drug called WR 279396 cured cutaneous leishmaniasis in laboratory animals. The active ingredients in WR 279396 are 2 aminoglycosides-- paromomycin sulfate (15%) and gentamicin sulfate (0.5%)-- in a base (AQIC). In initial clinical studies, WR 279396 given over 20 days was well tolerated in healthy human volunteers, although there was some local redness. We want to give WR 279396 to patients with cutaneous leishmaniasis to see if the dose of WR 279396 used in the normal volunteers can cure the disease. If the drug is effective, the benefit to you/your child is that you/your child will be treated with a topical drug which is expected to have little toxicity rather than with a painful injection with potentially toxic antimony.

The Tunisian and French official health organizations have been informed that this study is being conducted. This study was reviewed by the Tunisian Ethical Committee and the Paris-Cochin Consultative Committee for the Protection of People in Biomedical Research, which approved the study on the 27th of September 2002 and on the 30th of August 2002 respectively.

PURPOSE OF THE STUDY

The purpose of this study is 1) To determine if WR 279396 can cure cutaneous leishmaniasis, 2) To further determine the tolerance profile of the drug in patients with cutaneous leishmaniasis in the Old World.

PROCEDURES TO BE FOLLOWED

You/your child have a primary diagnosis of cutaneous leishmaniasis. Since we have visualized parasites in your/your child lesion, you/your child are now eligible to volunteer for a clinical trial for treatment. If you/your child agree to participate in this study, you/your child will have a medical history and physical examination, and give blood and urine, to be sure you/your child qualify for the study. Your/your child lesion(s) may be sampled with a needle, and/or scraped, and/or biopsy (cut out a small piece of skin one half the size of a standard pencil eraser after deaden the nerves by injecting a small amount of local anesthetic) to further characterize the*Leishmania* parasites that are in your lesion. You/your child will also have a total of three hearing test done to make sure your hearing is normal. You/your child will then have one cream (either WR 279396 or placebo) put on each lesion twice daily for 20 days. You/your child will not have a choice about which cream is used.

Subject Initials _______ Subject Number _______ Witness Initials _______

All the lesions will receive the cream assigned to you/your child. Study personnel will apply the cream. If you/your child are a volunteer in the Paris clinical site the study personnel will apply the cream in the morning and you or your parent/guardian will apply the cream in the afternoon and holidays.

You/your child will need to stay in the area for those 20 days. You/your child will be questioned daily to determine if the cream hurts your/your child normal skin surrounding the lesion, or hurts your/your child lesion. At the end of the 20 days of treatment, and at 30 days, 80 days, and 6 months after the end of treatment, your/your child lesion (s) will be examined to see if it is better or cured. If your/your child lesion (s) is not improved by 30 days or cured by 6 months, you/your child will be removed from the protocol and offered the best treatment according to your/your child doctor. This treatment will probably be Glucantime.

At the beginning, during and at the end of treatment, blood will be drawn (5 ml) to check the number of your/your child blood cells and blood chemistry tests (5 ml). For female participants of child-bearing age female in Paris, one of the chemistry tests will be a pregnancy test. Your/your child plasma will be used to determine the amount of WR 279396 is circulating in your/your child blood. Your/your child blood will be used exclusively for the tests mentioned above. You/your child understand that there is the possibility that the parasite obtained by culturing samples from your/your child lesion, which you/your child are providing under this study, may also be used in other *Leishmania* research studies. A table will be provided to you that explain when and what procedures will be conducted during the study (Table 3 - Paris/ Table 4 Tunis).

Photographs will be taken of your/your child lesion(s) before, at the end of therapy and at 30 days, 80 days and 6 months after the end of therapy. Physically identifying features if present will be hidden in photographs. Patients will be identified by initials and not by name.

INVESTIGATIONAL MEDICATION

WR279396 is an investigational medicine and is not approved by the US Food and Drug Administration (USFDA) for general use, however the USFDA has allowed its use in this research study. This product has only been used on 57 subjects so far.

EXPECTED DURATION OF PARTICIPATION - 7 months.

RISKS, HAZARDS, DISCOMFORTS, AND SAFEGUARDS

You/your child should be aware of the risks of this study and the way the risks will be monitored. 1) The drug might irritate your/your child skin--we will monitor this by daily examination and questioning. 2) The drug might be absorbed into your/your child blood and affect your hearing or kidneys--we will monitor this by hearing and blood tests. 3) Since the drug is a local medicine, the local lesions might be cured but disease might recur further up your/your child skin or (rarely) in

Subject Initials _______ Subject Number ________ Witness Initials ________

your/your child nose. You/your child should realize that these recurrences could occur no matter how you/your child are treated: skin recurrences can occur after a lesion has healed with Glucantime therapy; nasal recurrences can occur after a lesion has healed with Glucantime therapy or without therapy. You/your child and your/your child doctor can watch for skin recurrences during the 6 months of follow-up.

The volume of blood to be taken will not cause any adverse effects. The inconveniences of drawing blood are mild pain and bruising. Less than 20 milliliters will be withdrawn prior to therapy, in the middle of therapy, at the end of therapy and 30 days after the end of therapy. On any one day, no more than 20 milliliters of blood will be taken from you/your child. During the entire study, the total amount of blood to be taken from you/your child will be approximately 75 milliliters. If you/your child are in Paris, you/your child will also have to collect urine for 24 hours two times during the study, and store it in a provided container in your refrigerator.

Another inconvenience to you/your child is that if you/your child enter the study, you/your child will be expected to be available for re-examination 30 days, 80 days, and 6 months after the end of therapy. Although this may be difficult for you/your child, it is important to insure that your/your child disease does not recur.

Your/your child participation in this study prohibits you from donating blood during treatment and follow-up.

As in any study treatment or procedures may cause risks that are currently unforeseeable.

PREGNANCY PREVENTION

Female participants should avoid becoming pregnant during the study and for at least 1 month after participation in the study. Female participants should either abstain from sexual relations or practice a method of birth control. Except for surgical removal of the uterus, birth control methods such as the use of condoms, a diaphragm or cervical cap, birth control pills, IUD, or sperm killing products are not totally effective in preventing pregnancy.

PRECAUTIONS TO BE OBSERVED BY SUBJECT BEFORE AND DURING STUDY

Some drugs and some vaccines may interfere with how your/your child Leishmania lesion will cure. You/your child should take no medications (including over-the-counter medicines, such as aspirin, acetaminophen [Tylenol] or receive any vaccines starting 2 weeks prior to the start of the study, during the study, and for at least 2 weeks after you/your child final dose of topical without discussing it with one of the study doctors. If we are notified before you/your child are given such therapy, we may be able to recommend a specific drug that will treat you/your child but will not interfere with the study. Regardless, if any drugs or vaccines are prescribed for you/your child, or if you/your child consider taking any drugs during the course of the study, it is

Subject Initials _______ Subject Number ________ Witness Initials ________

important that the study physician know about it.

SAFEGUARDS

In order to prevent or minimize risks and hazards associated with this study you/your child will be closely monitored. A study physician will always be on call at one of the phone numbers listed below.

BENEFITS

If you/your child receive WR 279396 (or placebo) and are cured, you/your child will have been cured without injections. An indirect benefit to you/your child is knowing that you/your child have helped with a scientific study that will benefit other persons who might become infected in the future. In addition, benefits to all subjects enrolled in the study include a complete parasitology diagnosis, access to medical professionals who will perform a medical history, physical and dermatology exam and laboratory tests and reduced instances of bacterial infection due to keeping the wound clean and occluded.

Direct benefits to placebo subjects are: a) placebo effect and cosmetic benefits of treating lesion with the carrier; b) elimination of bacterial infections by keeping the lesion clean and occluded; c) direct access to a medical team that will perform a medical history, physical exam, dermatology exam, and laboratory tests; d) complete parasitology diagnosis.

If WR 279396 (or placebo) does not cure you/your child, you/your child will be treated by your/your child physician in the best way he knows, probably with another standard drug for leishmaniasis Glucantime.

ALTERNATIVE TREATMENT

If you/your child choose not to participate in this study, your/your child personal physician will treat you/your child with standard procedures, which may consist of a course of Glucantime by injection either directly into the lesion(s) or intramuscularly based on the number of lesions.

ASSURANCE OF CONFIDENTIALITY OF VOLUNTEER'S IDENTITY

All of the data concerning you/your child that will be collected during the study will be made anonymous and the files linked to your/your child participation, as a research subject will remain confidential. Representatives of the Institut Pasteurs in Paris and Tunisia, the Ministry of Health of France and Tunisia, the U.S. Food and Drug Administration, or representatives of the U.S. Army, as part of their responsibility to oversee the research, may review the records. Your/your child name will not be used in any report resulting from this study. In accordance with the French law " Loi relative à l'Informatique , aux Fichiers et aux Libertés" of 6 January 1978, article 40, modified

Subject Initials ______ Subject Number _______ Witness Initials _______

on 1 July 1994, and with the Tunisian law related to clinical trials (Law N° 90-1401 of the 3d of September 1990), you/your child will have the right to review your/your child study records directly or through a physician of your/your child choice. The CNIL will be notified of this Phase 2 study. Sample retains for future use will be only identified with the volunteer number.

WITHDRAWAL OF PARTICIPATION BY THE INVESTIGATOR

The investigator may withdraw you/your child from participation in this research study, even if you/your child would like to continue, if circumstances arise which warrant such action. If you/your child experience any of the following: (1) you/your child develop health conditions which would make your/your child continued participation in this study dangerous to you/your child; and (2) other conditions which might occur that make your/your child participation detrimental to your/your child own health. The investigator will make the decision and let you/your child know if it is not possible for you/your child to continue. The decision may be made either to protect your/your child health and safety, or because it is part of the research plan to stop the participation of volunteers who develop certain medical conditions.

WITHDRAWAL BY PATIENT

If you/your child wish to withdraw from the study please contact one of the investigators.

COST OF PARTICIPATION

Medical care related to your/your child participation in this study will be provided by the sponsors of this clinical study.

PAY FOR PARTICIPATION

You/your child should not suffer economically for participating in this study. The study should cover if required your/your child transportation from work or home to the study site and for the hours you do not work because you/your child are participating in the study. The administrative staff of the study will pay you/your child at the end of the study or at intervals during the study if required.

VOLUNTEER REGISTRY DATA BASE REQUIREMENTS

It is the policy of the U. S. Army Medical Research and Materiel Command that data sheets (USAMRDC Form 60-R) are to be completed on all volunteers participating in research for entry into this Command's Volunteer Registry Database. The information to be entered into this Subject confidential database includes your/your child name, address, subject number, study name, and dates. The intent of the database is two-fold: first, to readily answer questions concerning an individual's participation in research sponsored by USAMRMC; and second, to ensure that

Subject Initials _______ Subject Number ________ Witness Initials ________

USAMRMC can exercise its obligation to ensure research volunteers are adequately warned (duty to warn) of risks and to provide new information as it becomes available. The information will be stored at USAMRMC for a minimum of 75 years.

SIGNIFICANT NEW FINDINGS.

Any significant information that may affect your/your child health and willingness to continue participation that is found during the study will be made available to you/your child. If new information is provided to you/your child, your/your child consent to continue participating in this study will be re-obtained.

NUMBER OF VOLUNTEERS IN THE STUDY

One hundred (100) patients will be enrolled in this study.

MEDICAL CARE FOR INJURY OR ILLNESS

The United States Department of Defense is funding this research project. Should you/your child be injured as a direct result of participating in this research project, you/your child will be provided medical care, at no cost to you/your child, for that injury. The Co-sponsor of this study, the Institut Pasteur in Paris is insured. The insurance will cover any possible damages resulting from your/your child participation in the study. This does not release the organizers of this research from their responsibilities. You/your child retain your/your child statutory rights under the Huriet Law of 20 December 1988. You/your child should discuss this issue thoroughly with the principal and/or Co-investigator before you/your child enroll in this study.

IDENTIFICATION OF INVESTIGATORS

In the event of a research related injury, or if you/your child experience an adverse reaction, please immediately contact one of the investigators listed below. Please contact one of the following if you/your child have any questions concerning this medical research study:

Dr. Pierre Buffet	Call collect : 01 40 61 38 17<br>FAX: 33-1-45688218
or<br>Dr. Afif Ben Salah	Call collect: 216-1-792-429<br>FAX: 216-1-791-833
or<br>Dr. Max Grogl	Call collect: 301-319-9359<br>FAX: 301-319-9180

Subject Initials _______ Subject Number _______ Witness Initials _______

or
Dr. Jonathan D. Berman Call collect: 301-594-7105

For information or answers to questions concerning your/your child rights as a research subject you/your child may contact: Dr. Odile Launay, Centre de Centre des essais vaccinaux Cochin-Pasteur, Hôpital Cochin, Pavillon Saint-Louis, 27 rue du Faubourg Saint-Jacques, 75679 Paris Cedex 14, France, Phone : 01133-1-4407-1541 Fax : 011-33-1-4046-9308, or Dr. Hechmi Louzir, Institut Pasteur de Tunis13 Place Pasteur BP, 74 Belvedere 1002, Tunis, Tunisia, Phone: 011-216-1-783-022 #213. If you become ill during the study please report to the Hospital where you were seen for this study or call Dr. Pierre Buffet (Phone: 01 40 61 38 17) or Dr. Afif Ben Salah (Phone: 216-1-792-429).

IF THERE IS ANY PORTION OF THIS CONSENT EXPLANATION SHEET THAT YOU DO NOT UNDERSTAND, ASK THE INVESTIGATOR BEFORE SIGNING.

IF THE SUBJECT IS A CHILD IT IS THE RESPONSIBILITY OF THE PI AND THE LEGAL REPRESENTATIVE TO BE SURE THAT THE CHILD IS COOPERATIVE AND WILLING TO PARTICIPATE IN THE STUDY. PLEASE USE AT ALL TIME TERMS IN ACCORDANCE TO THE SUBJECT'S SCHOOLING LEVEL. CFR 50.20, REQUIRES THAT "THE INFORMATION THAT IS GIVEN TO THE SUBJECT SHALL BE IN LANGUAGE UNDERSTANDABLE TO HIM. THIS IS ESPECIALLY IMPORTANT IF THE VOLUNTEER IS A CHILD.

I have read the information provided above. I have been given an opportunity to ask questions and all of my questions have been answered to my satisfaction. I have been given a copy of this form.

I **do** **do not** *(check one & initial)* consent to the inclusion of this form in my outpatient medical treatment record.

PRINTED NAME OF VOLUNTEER:

SIGNATURE OF VOLUNTEER	DATE	SIGNATURE OF LEGAL GUARDIAN *(if volunteer is a minor)*
PERMANENT ADDRESS OF VOLUNTEER	TYPED NAME OF WITNESS	
		DATE

REVERSE OF DA FORM 5303-R, MAY 89

PARTIE B – A REMPLIR PAR L'INVESTIGATEUR

INSTRUCTIONS CONCERNANT LES ELEMENTS CONSTITUTIFS DU CONSENTEMENT ECLAIRE (*Donner une explication détaillée conformément à l'annexe C AR 40-38 ou AR 70-25*)

Appendix A –4 **(Déclaration solennelle – Partie B- Formulaire Explicatif du Consentement en anglais)**

TITRE DE L'ETUDE : "TRAITEMENT TOPIQUE DE LA LEISHMANIOSE CUTANEE AU MOYEN DU WR 279396 : ETUDE DE PHASE 2 DANS L'ANCIEN MONDE"

CO-PROMOTEURS : The Office of the Surgeon General (OTSG : Structure du ministère de la Défense Américain centralisant la surveillance du déroulement des études impliquant les êtres humains) et l'Institut Pasteur de Paris.

CO-INVESTIGATEURS/MÉDECINS RESPONSABLES DE L'ÉTUDE

Dr. Pierre BUFFET, Centre Médical de l'Institut Pasteur à Paris. Téléphone: 33 1- 4061-3817 Fax: 33- 1- 4568-8218 et le Dr. Afif BEN SALAH, Institut Pasteur en Tunisie. Téléphone: 216-1- 792-429, Fax: 216- 1- 791-833.

INFORMATION CONCERNANT LA PARTICIPATION

Une maladie nommée leishmaniose cutanée a été diagnostiquée chez vous/chez votre enfant. La leishmaniose cutanée est causée par des parasites qui sont transmis à l'homme par la piqûre du phlébotome (petit moustique). Les parasites envahissent les cellules de la peau et provoquent un ulcère. De manière caractéristique, la leishmaniose cutanée guérit spontanément mais ceci peut prendre plusieurs mois, et entre-temps, la maladie peut s'étendre à d'autres parties de la peau. Dans de rares cas, elle peut s'étendre au nez.

Initiales du Sujet ________ Numéro du Sujet _______ Initiales des Témoins ______

Le traitement standard consiste à faire des injections d'antimoine sous forme d'antimoniate de méglumine (Glucantime). Bien que ce médicament soit généralement efficace, le traitement par antimoines présente quelques inconvénients, à savoir l'administration au patient d'au moins cinq injections douloureuses directement dans les lésions, l'occurrence d'échec et de rechutes, et, lorsque ce traitement est administré par voie intramusculaire, un risque de toxicité pour le cœur, le foie et le pancréas. Le but principal de la présente étude est de comparer le WR 279396 à un placebo et de déterminer si l'application d'une crème sur les lésions est aussi efficace que les injections de Glucantime. Un autre objectif important est de déterminer si la crème provoque des douleurs et si elle passe dans le sang. Etant donné que les lésions guérissent spontanément, et que nous avons besoin de savoir combien de guérisons sont dues spécifiquement à l'application de la crème, la présente expérimentation sera conduite en double aveugle contre placebo. Vous/votre enfant serez affecté, par désignation aléatoire, soit au groupe « substance active » soit au groupe « placebo ». Cela signifie que 50% des volontaires auront à utiliser une crème qui ne contient aucun principe actif (« le placebo ») alors que les autres 50% auront à utiliser la crème active (le WR 279396). Ni vous/votre enfant ni votre médecin ne saurez laquelle des deux crèmes vous utilisez (c'est ce qu'on appelle le « double aveugle »). Le placebo et la crème active WR 279396 ont le même aspect.

Puisque vous/votre enfant avez été choisi pour participer à une étude de recherche médicale, il est très important pour vous de comprendre les principes généraux suivants, qui s'appliquent à toutes les personnes participant à nos études, qu'il s'agisse de volontaires sains ou de volontaires malades : (1) votre participation est totalement volontaire ; (2) à tout moment, vous/votre enfant avez la possibilité de vous retirer de cette étude ou de n'importe quelle partie de celle-ci. Votre refus de participer n'impliquera aucune sanction ou perte d'avantages auxquels vous avez par ailleurs droit ; (3) après avoir lu les explications fournies, vous devrez poser toutes les questions susceptibles de vous aider à comprendre clairement la nature de cette étude.

Initiales du Sujet ________ Numéro du Sujet _______ Initiales des Témoins ______

INTRODUCTION

Les chercheurs de l'Institut de Recherche Militaire américain Walter Reed ont élaboré une crème susceptible de guérir la leishmaniose cutanée. Ce médicament, appelé WR 279396, a guéri des animaux de laboratoire atteints de leishmaniose cutanée. Les principes actifs utilisés dans le WR 279396 consistent en deux produits : le sulfate d'aminoglycosides-paromomycine (15%) et le sulfate de gentamicine (0,5%) dans une base (AQIC). Dans les études cliniques initiales, le WR 279396 administré sur 20 jours a été bien toléré par les volontaires humains en bonne santé, malgré l'apparition de quelques rougeurs localement. Nous souhaitons administrer le WR 279396 à des patients atteints de la leishmaniose cutanée pour déterminer si la dose de WR 279396 utilisée chez les volontaires sains est capable de guérir la maladie. Si le traitement est efficace, l'avantage pour vous est que vous serez traité par un médicament topique à toxicité probable faible, plutôt que par injection douloureuse d'antimoine potentiellement toxique.

Les structures officielles de santé tunisiennes et françaises ont été informées du fait que cette étude va être réalisée. La présente étude a été examinée par le Comité d'Ethique Tunisien et par le Comité Consultatif de Protection des Personnes qui se prêtent à la recherche biomédicale de Paris-Cochin qui ont rendu un avis favorable respectivement le 29 mars 2002 et le 29 août 2002.

OBJECTIF DE L'ETUDE

L'objectif de la présente étude est 1) de déterminer si le WR 279396 peut guérir la leishmaniose cutanée ; 2) de déterminer en outre le degré de tolérance du traitement chez des patients atteints de leishmaniose cutanée dans l'Ancien Monde.

PROCEDURE A SUIVRE

Le diagnostic principal posé chez vous/chez votre enfant est une leishmaniose cutanée. Comme nous avons visualisé les parasites dans votre lésion/la lésion de votre enfant, vous/votre enfant êtes maintenant éligible pour vous porter volontaire pour un essai thérapeutique. Si vous/votre enfant acceptez de participer à cette étude, vous/votre enfant ferez alors l'objet d'une visite médicale et votre passé médical/le passé médical de votre enfant sera passé en revue, des

Initiales du Sujet ________ Numéro du Sujet _______ Initiales des Témoins ______

prélèvements sanguins et d'urine seront effectués afin qu'on s'assure que vous/votre enfant êtes bien apte à participer à cette étude. Si vous/votre enfant acceptez de participer à cette étude, nous pourrions effectuer, à titre d'échantillons, des prélèvements sur votre / vos lésion(s), au moyen d'une aiguille, par grattage et/ou par biopsie (découpage d'un petit fragment de peau – grand comme la moitié d'une gomme de crayon à papier - après avoir neutralisé les sensations nerveuses par injection d'une petite quantité d'anesthésique local) pour déterminer si ces lésions contiennent vraiment les parasites du genre *Leishmania*. . Vous/votre enfant passerez également un examen d'acuité auditive afin de vérifier si celle-ci est normale. Ensuite, nous vous appliquerons une des deux crèmes (soit le WR 279396, soit le placebo) sur chaque lésion deux fois par jour pendant 20 jours. Vous ne pourrez pas choisir laquelle des deux crèmes sera utilisée. Toutes les lésions seront traitées par la crème qui vous aura été attribuée. La crème sera appliquée par le personnel de l'étude. Mais si vous/votre enfant êtes un volontaire du site clinique de Paris, la crème sera appliquée le matin par le personnel de l'étude et l'après-midi, par vous-même ou par l'un de vos parents / ou votre tuteur.

Vous/votre enfant devrez rester dans la région pendant ces 20 jours. Vous/votre enfant serez interrogé quotidiennement pour qu'on sache si la crème provoque des irritations sur la peau saine qui entoure la lésion ou si elle provoque des douleurs au niveau de votre lésion. A la fin des 20 jours de traitement, puis 30 jours, 80 jours, et 6 mois après la fin du traitement, votre / vos lésion(s) sera / seront examinée(s) pour voir s'il y a amélioration ou guérison. Si votre / vos lésion(s) ne montre(nt) aucune amélioration au bout de 30 jours ou si elle n'est pas / elles ne sont pas guérie(s) au bout de 6 mois, vous serez retiré du protocole et on vous offrira le meilleur traitement en accord avec votre médecin. Ce traitement sera probablement le Glucantime.

Au début, pendant et à la fin du traitement, une prise de sang sera effectuée (5 ml) pour examiner le nombre de vos cellules sanguines et pour effectuer une analyse biologique classique de votre sang (5 ml). Si vous êtes une femme en âge d'avoir des enfants à Paris, un des examens sanguins sera un test de grossesse. Votre plasma sera utilisé pour déterminer la quantité de WR 279396 circulant dans votre sang. Votre sang sera utilisé exclusivement pour les tests mentionnés ci-

Initiales du Sujet ________ Numéro du Sujet _______ Initiales des Témoins ______

dessus. Avec votre accord, la culture parasitaire que vous/votre enfant fournissez dans le cadre de cette étude pourra aussi être utilisée dans d'autres études de recherche sur les leishmanies. On vous fournira un tableau explicatif vous exposant la chronologie et la manière dont les procédures seront effectuées au cours de l'étude (Tableau 1 – Paris / Tableau 2 – Tunis).

Des photographies de votre / vos lésion(s) sera/seront prise(s) avant et à la fin du traitement et 30 jours, 80 jours, et 6 mois après la fin du traitement. Si des traits d'identification physique apparaissent sur ces photographies, ils seront cachés. Les patients seront identifiés par des initiales et non pas par leur nom.

MEDICATION D'INVESTIGATION

Le WR 279396 est un médicament d'investigation et il n'est pas approuvé par la FDA (Food and Drug Administration) des Etats-Unis pour une utilisation auprès du public. Toutefois, la FDA des Etats-Unis a autorisé son utilisation dans la présente étude de recherche. Ce produit a été, jusqu'à ce jour, utilisé sur 57 sujets uniquement.

DUREE PREVUE POUR LA PARTICIPATION – 7 mois

RISQUES, DANGERS, GENES ET GARANTIES

Vous/votre enfant devez être conscient des risques que présente cette étude et de la manière dont ces risques seront gérés. 1) Le médicament peut provoquer des irritations de la peau – nous gérerons cela par un examen et des questions quotidiennement. 2) Le médicament pourrait passer dans le sang et affecter ainsi votre acuité auditive ou vos reins – nous gérerons cela en vous faisant passer des tests auditifs et des test sanguins. 3) Etant donné que le médicament est un topique, les lésions locales peuvent être guéries mais la maladie, elle, peut réapparaître sur d'autres parties de la peau ou (rarement) au niveau du nez. Vous/votre enfant devrez savoir que ces récurrences peuvent se produire quelle que soit la manière dont ces lésions ont été traitées : la

Initiales du Sujet ________ Numéro du Sujet _______ Initiales des Témoins ______

réapparition de lésions sur la peau peut se manifester après qu'une lésion a été guérie avec du Glucantime ; la réapparition de lésions au niveau du nez peut se produire après que ces lésions ont été guéries avec du Glucantime ou sans traitement. Vous/votre enfant et votre médecin pouvez surveiller s'il y a réapparition de ces lésions pendant les 6 mois qui suivent.

La quantité de sang qui sera prélevée ne causera pas d'effets indésirables. Les inconvénients du prélèvement sanguin se limitent à une douleur modérée et à des ecchymoses. La quantité prélevée avant le début du traitement, au milieu, à la fin et 30 jours après la fin de celle-ci sera de moins de 20 millilitres. En une journée donnée, la quantité de sang prélevée ne dépassera pas 20 millilitres. Pendant toute la durée de l'étude, la quantité totale de sang prélevé sera d'environ 75 millilitres. Si vous/votre enfant participez à l'étude à Paris, Il vous faudra recueillir vos urines de 24 heures deux fois pendant l'étude, et les conserver au réfrigérateur dans un récipient préalablement fourni.

Un autre inconvénient pour vous / votre enfant est que si vous participez à cette étude, vous allez devoir être disponible pour subir des examens de suivi 30 jours, 80 jours, et 6 mois après la fin du traitement. Bien que ceci puisse être difficile pour vous/ votre enfant, il est important de s'assurer que votre maladie ou celle de votre enfant ne réapparaît pas.

La participation à cette étude vous interdit de pratiquer un don de sang pendant le traitement et après le le suivi du traitement.
Comme dans toute étude, le traitement ou les procédures peuvent entraîner des risques actuellement imprévisibles.

PREVENTION D'UNE GROSSESSE

Si vous êtes une femme vous devez éviter de tomber enceinte pendant la durée de l'étude et, au moins, pendant 1 mois après votre participation à cette étude. Pour éviter une grossesse, vous devez soit vous abstenir d'avoir des rapports sexuels, soit utiliser une méthode de contraception.

Initiales du Sujet ________ Numéro du Sujet _______ Initiales des Témoins ______

A l'exception d'une hystérectomie, les méthodes de contraception telles que le préservatif, le diaphragme ou le capuchon cervical, les pilules contraceptives, les dispositifs intra-utérins (D.I.U.) ou les spermicides ne sont pas totalement efficaces pour éviter une grossesse.

PRECAUTIONS A PRENDRE PAR LES SUJETS AVANT ET PENDANT L'ETUDE

Certains médicaments et vaccins peuvent interférer sur la manière dont lésions de la leishmaniose sont guéries. Vous/votre enfant ne devez prendre aucun médicament (y compris les médicaments en vente libre, tels que de l'aspirine, l'acétaminophène[1] [Tylenol]) ni recevoir de vaccins dans les deux semaines précédant le début de l'étude, pendant la durée de l'étude et, au moins, dans les deux semaines après la prise de votre dernière dose de topique, sans l'avis de l'un des médecins de l'étude. Si nous sommes informés à l'avance que vous/votre enfant êtes sous tel ou tel traitement, nous serons en mesure de vous conseiller un médicament spécifique pour vous/votre enfant traiter mais qui n'interférera pas avec l'étude. Quoi qu'il en soit, si on vous/votre enfant prescrit des médicaments ou des vaccins ou si vous/votre enfant envisagez de prendre des médicaments pendant le déroulement de l'étude, il est important que les médecins chargés de l'étude en soient informés.

GARANTIES

Afin de prévenir ou de minimiser les risques et les dangers liés à cette étude, vous/votre enfant serez attentivement suivis. Un des médecins chargés de l'étude sera joignable par téléphone en permanence aux numéros donnés ci-dessous.

AVANTAGES

Si vous/votre enfant êtes traité par le WR 279396 (ou le placebo) et que vous/votre enfant guérissez, vous/votre enfant serez guéri sans subir d'injections. Un avantage indirect sera pour vous/votre enfant de savoir que vous/votre enfant avez contribué à une étude scientifique qui bénéficiera à d'autres personnes qui pourraient être atteintes dans le futur. De plus, les avantages

Initiales du Sujet ________ Numéro du Sujet _______ Initiales des Témoins ______

[1] appelé en France paracetamol (Doliprane, Efferalgan, ...)

bénéficiant à tous les sujets impliqués dans l'étude comprennent un diagnostic parasitologique complet, un contact avec des professionnels de la santé qui rédigeront un historique médical, un examen physique et dermatologique ainsi que des essais en laboratoire et des risques réduits d'infection bactérienne en gardant la plaie propre et fermée.

Les avantages directs profitant aux sujets soumis au placebo sont : a) un effet placebo et des avantages cosmétiques par traitement de la lésion avec le véhicule ; b) l'élimination des infections bactériennes en gardant la lésion propre et fermée ; c) un contact direct avec une équipe médicale qui rédigera un historique médical, un examen physique, un examen dermatologique et des essais en laboratoire ; d) un diagnostic parasitologique complet.

Si le WR 279396 (ou le placebo) ne vous guérit pas, votre médecin traitant vous traitera selon les meilleures procédures possibles, probablement avec un autre médicament, un médicament classique pour traiter la leishmaniose, le Glucantime.

TRAITEMENT DE SUBSTITUTION

Si vous/votre enfant décidez de ne pas participer à cette étude, votre médecin traitant vous traitera avec les moyens classiques, qui peuvent consister dans une administration de Glucantime par injection directement dans la / les lésion(s) ou par injection intramusculaire selon le nombre de lésions.

GARANTIE DE LA CONFIDENTIALITE DE L'IDENTITE DES VOLONTAIRES

Toutes les données vous concernant (vous/votre enfant) qui seront recueillies au cours de l'étude seront rendues anonymes et les dossiers liés à votre participation en tant que sujet de la présente recherche demeureront confidentiels. Ces documents peuvent être consultés par des représentants de l'Institut Pasteur à Paris ou en Tunisie, du Ministère de la Santé en France et en Tunisie, de la FDA des Etats-Unis, ou de représentants de l'armée américaine dans le cadre de leur responsabilité de supervision de cette recherche. Votre nom/le nom de votre enfant ne sera utilisé dans aucun rapport issu de cette étude. Conformément à la loi française intitulée "Loi relative à

Initiales du Sujet ________ Numéro du Sujet _______ Initiales des Témoins ______

l'Informatique, aux Fichiers et aux Libertés" du 6 janvier 1978, article 40, modifié le 1er juillet 1994, et conformément à la loi tunisienne concernant les essais cliniques (Loi N° 90-1401 du 3 septembre 1990), vous/votre enfant aurez le droit de consulter vous-même directement les documents de l'étude ou de les faire consulter par un médecin de votre choix. La Commission Nationale Informatique et Libertés sera informée de cette étude de Phase 2. Les échantillons conservés pour usage futur seront identifiés uniquement par le numéro de code patient.

ANNULATION DE LA PARTICIPATION PAR L'INVESTIGATEUR

L'investigateur peut mettre fin à votre participation à la présente étude de recherche, même si vous/votre enfant souhaitez continuer, si des circonstances surviennent qui justifient cette décision. Si vous/votre enfant êtes sujet à l'une quelconque des situations suivantes : 1) vous/votre enfant développez un état de santé susceptible de rendre votre participation à la présente étude dangereuse pour vous/votre enfant ; et (2) vous/votre enfant vous retrouvez dans d'autres conditions incidentes qui rendent votre participation préjudiciable à votre état de santé. L'investigateur décidera et vous fera savoir si vous/votre enfant êtes dans l'impossibilité de continuer. Cette décision peut être prise, soit pour protéger votre santé et préserver votre sécurité, soit parce que cela fait partie du programme de recherche d'arrêter la participation de personnes qui se trouvent dans certaines situations médicales.

RETRAIT A L'INITIATIVE DU PATIENT

Vous/votre enfant êtes libre de quitter l'étude à tout moment, si vous/votre enfant le décidez. Veuillez en avertir l'un des médecins investigateurs.

COUT DE PARTICIPATION

Les soins médicaux liés à la participation à cette étude seront pris en charge par les promoteurs de cette étude clinique.

Initiales du Sujet ________ Numéro du Sujet _______ Initiales des Témoins ______

DEDOMMAGEMENT AU TITRE DE LA PARTICIPATION

Vous/votre enfant n'aurez pas à supporter de frais pour participer à la présente étude. L'étude dédommagera, si cela est nécessaire, les frais de vos déplacements entre votre lieu de travail ou votre domicile et le site de l'étude ainsi que les heures non travaillées à cause de votre participation à l'étude. Le personnel administratif de l'étude vous dédommagera soit à la fin de l'étude soit, si nécessaire, périodiquement pendant la durée de l'étude.

EXIGENCES LIEES A LA BASE DE DONNEES "REGISTRE DES VOLONTAIRES"

C'est la politique du Commandement de la Recherche Médicale et du Matériel Médical de l'armée américaine (USAMRDC) de faire compléter des formulaires de données (USAMRDC formulaire 60-R) sur tous les volontaires participant à la recherche en vue d'entrer les informations dans la base de données "registre des volontaires" du Commandement. Les informations qui doivent être entrées dans cette base de données confidentielle concernant les sujets comprennent les initiales de vos nom, adresse, numéro de code patient dans l'étude, nom et dates de l'étude. L'objectif de cette base de données est double : premièrement, répondre aisément aux questions concernant la participation d'un individu à une recherche sponsorisée par l'USAMRMC, et, deuxièmement, de garantir la possibilité pour l'USAMRMC de remplir son obligation de s'assurer que les volontaires sont correctement informés (devoir de prévenir) des risques et de fournir de nouvelles informations dès qu'elles sont disponibles. Les informations sont archivées à l'USAMRMC pour une durée minimale de 75 ans.

NOUVEAUX RESULTATS IMPORTANTS

Toute information importante, susceptible d'affecter votre santé et votre volonté de poursuivre votre participation, obtenue pendant la durée de l'étude sera mise à votre disposition. Si de nouvelles informations vous sont communiquées, votre consentement pour poursuivre votre participation à la présente étude vous sera à nouveau demandé.

NOMBRE DE VOLONTAIRES DANS LA PRESENTE ETUDE

Cent (100) patients seront inscrits pour cette étude.

Initiales du Sujet ________ Numéro du Sujet ________ Initiales des Témoins ______

SOINS MEDICAUX POUR PREJUDICE

Ce projet de recherche est financé par le Ministère de la Défense des Etats-Unis. Si vous/votre enfant subissez des blessures qui sont dues d'une manière directe à votre participation à ce projet de recherche, vous recevrez des soins médicaux, sans que cela n'entraîne pour vous/votre enfant des frais à payer. L'Institut Pasteur de Paris, co-promoteur de cette étude, a souscrit une assurance. Cette assurance couvrira tous les dommages que votre participation à l'étude pourrait entraîner. Ceci ne dégage en rien les organisateurs de la recherche de leurs responsabilités. Vous conservez tous vos droits garantis par la loi Huriet du 20 décembre 1988. Vous devez discuter cette question de manière approfondie avec l'investigateur principal avant de vous engager dans cette étude.

IDENTIFICATION DES INVESTIGATEURS

Si vous/votre enfant êtes victime de blessures liées à cette recherche ou si vous/votre enfant faites l'objet d'une réaction indésirable, veuillez contacter l'un des investigateurs cités ci-dessous. Si vous/votre enfant avez des questions concernant cette étude de recherche médicale, veuillez contacter l'une des personnes suivantes :

Dr. Pierre BUFFET — N° de tél.: 01 40 61 38 17
N° de Fax: 33 1- 45688218

Ou

Dr. Afif BEN SALAH — N° de Tél.: 216-1- 792-429
N° de Fax: 216-1- 791-833

Ou

Dr. Max GROGL — N° de Tél. 00 1 301-319-9359
N° de Fax: 00 1 301- 319-9180

Ou

Dr. Jonathan D. BERMAN — N° de tél. 00 1 301- 594-7105

Pour obtenir des informations ou des réponses à vos questions concernant vos droits en tant que

Initiales du Sujet ________ Numéro du Sujet _______ Initiales des Témoins ______

sujet de la recherche, vous pouvez contacter le Dr. Odile Launay, Centre des essais vaccinaux Cochin-Pasteur, Hôpital Cochin, Pavillon Saint-Louis, 27, rue du Faubourg Saint-Jacques, 75679 Paris Cedex 14, France, téléphone : 01133-1-4407-1541 Fax : 011-33-1-4046-9308, ou le Dr. Hechmi Louzir, Institut Pasteur de Tunis, 13 Place Pasteur BP, 74 Belvedere 1002, Tunis, Tunisie, téléphone : 011-216-1-783-022 #213. Si vous/votre enfant tombez malade pendant la durée de l'étude, veuillez vous adresser à l'hôpital où vous/votre enfant étiez reçu pour cette étude ou contacter le Dr. Pierre BUFFET (Téléphone: 01 40 61 38 17) ou le Dr. Afif BEN SALAH (Téléphone: 216-1-792-429).

S'IL Y A UNE PARTIE DE CE DOCUMENT EXPLIQUANT LE PRINCIPE DE CONSENTEMENT QUI N'EST PAS SUFFISAMMENT CLAIRE POUR VOUS, VEUILLEZ POSER VOS QUESTIONS A L'INVESTIGATEUR AVANT DE SIGNER

SI LE SUJET EST UN ENFANT, IL EST DE LA RESPONSABILITE DE L'INVESTIGATEUR PRINCIPAL (IP) ET DU REPRESENTANT LEGAL DE S'ASSURER QUE L'ENFANT SOUHAITE COOPERER ET PARTICIPER A L'ETUDE. MERCI DE BIEN VOULOIR TOUJOURS EMPLOYER DES TERMES EN ADEQUATION AVEC LE NIVEAU D'INSTRUCTION DU SUJET. L'ARTICLE 50.20 DU "CODE OF FEDERAL REGULATIONS (CFR)" REQUIERT QUE "LES INFORMATIONS COMMUNIQUEES AU SUJET LUI SOIENT DONNEES DANS UN LANGAGE COMPREHENSIBLE. CECI EST PARTICULIEREMENT IMPORTANT SI LE VOLONTAIRE EST UN ENFANT".

J'ai lu les informations communiquées ci-dessus et j'ai eu l'occasion de poser des questions. Des réponses convaincantes ont été apportées à toutes mes questions. J'ai bien reçu une copie de ce formulaire.

J'ai donné ☐ **Je n'ai pas donné** ☐ (cochez l'une des cases et paraphez) mon accord pour l'inclusion de ce formulaire dans mon dossier de traitement médical des patients externes.

Initiales du Sujet ________ Numéro du Sujet _______ Initiales des Témoins ______

NOM DU VOLONTAIRE EN MAJUSCULES

<table>
<tr><td>SIGNATURE DU VOLONTAIRE</td><td>DATE</td><td colspan="2">SIGNATURE DU TUTEUR LEGAL (si le volontaire est mineur)</td></tr>
<tr><td rowspan="2">ADRESSE PERMANENTE DU VOLONTAIRE</td><td colspan="3">NOM DU TEMOIN EN CARACTERES D'IMPRIMERIE</td></tr>
<tr><td colspan="2"></td><td>DATE</td></tr>
</table>

VERSO DU FORMULAIRE DA 5303-R, mai 89

ملحق أ - 1

ملحقات

موافقة على التطوّع / موافقة طوعيّة

بمقتضى إقرار خطي بقسم

بخصوص إستخدام هذه المطبوعة - أنظر 25 - AR70 أو 38 - AR 40 الوكالة صاحبة الإقتراح هي مكتب كبير الأطباء OTSG

قانون 1974 المتعلّق بالسرّية

السلطة : USC 10 - 3013 - 44 3101USC - 10 USC - 1087 - 1071

الغرض الأصلي : توثيق المساهمة الطوعية في الإستقصاء السريري وبرنامج البحث رقم الضمان الإجتماعي والعنوان الشخصي سيقع إستعمالهما لأغراض التعريف وتحديد الموقع.

إستعمالات عادية : أن رقم الضمان الإجتماعي والعنوان (روتينية) الشخصي سيقع إستعمالهما لأغراض التعريف وتحديد الموقع وإن المعلومات المنبثقة عن الدراسة سيقع إستعمالها لتوثيق الدراسة . وإنّ المعلومات المنبثقة عن الدراسة ستستخدم لتوثيق الدراسة ولتنفيذ البرامج الطبيّة وللقضاء في الدعاوي وللتقرير الإجباري بخصوص الظروف الطبيّة حسبما يقتضيه القانون ويمكن تقديم المعلومات للوكالات الفيدرالية والحكومية والمحلية.

الكشف عن معطيات شخصيّة : إن تقديمك لرقم ضمانك الإجتماعي

ولعنوانك الشخصي الراهن وضروري

لتمكيننا من التعرف عليك و من الإتصال بك

إذا ما أشارت معلومات في المستقبل الى أنّه قد تتضرّر صحتك بشكل خطير

إن إحجامك عن تقديم هذه المعلومات قد يحول دون مشاركتك الطوعية في هذه الدراسة الإستقصائية

قسم -أ-

إقرار خطّي بقسم لمتطوع

الافراد المتطوعون بالإدارة المعتمدة للدراسات والبحوث الخاصّة بالجيش

إن المتطوّعين على مقتضى احكام AR40-38 و AR70-25
مخوّلون للإنتفاع بكامل الرّعاية الطبيّة الضرورية بسبب أي أذى أوإصابة ناتجة مباشرة عن مشاركتهم في مثل هذه الدراسات.

إني الممضي أسفله......................، أصرّح وانا مالك لكامل قوايا العقلية وقادر تماما على الموافقة وقد بلغت من العمر ....سنة، بأني أتطوّع / أوافق بصفتي ممثلا قانونيا لـ.. للمشاركة /على أن يشارك في دراسة بحث تحت عنوان " العلاج الموضعي لداء اللايشمانيا الجلدية بواسطة WR 279396 : دراسة المرحلة II في العالم القديم " تحت إدارة الدكتور عفيف بن صالح

الاحرف الأولى لاسم الفرد　　　　رقم الفرد　　　　الأحرف الأولى لاسم الشاهد

إن تبعات مشاركتي الطّوعية / موافقتي بصفتي ممثلا قانونيا، ومدّة البحث الدراسي والغرض منه والمناهج والوسائل التي سيتمّ إنجازه بمقتضاها
ولقد وضّح لي الدكتور عفيف بن صالح أو.......................... .... (اسم القائم بالإستقصاء المحلي الآخر) بمعهد باستور بتونس المظاهر المزعجة والمخاطر المتوقّعة أو المنتظر حصولها لي.

وقد منحت فرصة طرح أية أسئلة بخصوص هذه الدراسة الإستقصائية. وقد حصلت على أجوبة شافية وضافية لهذه الأسئلة نالت رضاي وأقنعتني . وإذا ما خامرتني أيّة تساؤلات أخرى بخصوص حقوقي أو الضرّر المتصل بالدراسة فإنه بإمكاني الإتصال على النحو التالي
- الدكتور عفيف بن صالح : خطوط مجمّعة : 216-1-792-429
فاكس : 216-1-791.833

أو

- الدكتور ماكس غروغل : هاتف : 301-319-9359

فاكس : 301-319-9180

وإني أدرك أنه بإمكاني في أي وقت من الأوقات خلال مدّة البحث الدراسي أن ألغي موافقتي أو انسحب / أجعل الشخص الذي أمثله ينسحب من الدراسة دون التعرّض الى عقوبة أو فقدان المنافع.

ومع ذلك فإنه / أو الشخص الذي أمثّله يمكنني / يمكنه أن يطالب بالخضوع لبعض الفحوص - إذا ما رأى الطبيب المباشر (أو المكلف بالدراسة) أن مثل هذه الفحوص ضرورية لسلامتي الصحيّة وعافيتي / للسلامة الصحية ولعافية الشخص الذي أمثله . وإن رفضي / أو رفض الشخص الذي أمثّله المشاركة لا تنجرّ عنه عقوبة أو فقدان منافع كنت / كان الشخص الذي أمثله حقيقا بها.

امضاء المتطوّع / امضاء الوصيّ القانوني ....

الولي

التاريخ : ....

اسم المتطوع بالأحرف الغليظة / اسم الولي ....

اسم الوصي القانوني ....

الأحرف الأولى للفرد رقم الفرد الأحرف الأولى للشاهد

العنوان : ....

رقم الضمان الإجتماعي ....

التامين

لقد كنت حاضرا خلال تقديم التوضيح (التفسير) المشار اليه أعلاه وبإمكاني أن أؤكد إتاحة الفرصة للمتطوع بأن يطرح الأسئلة وأشهد هنا على ان المتطوّع قد أودع إمضاؤه بحضوري :

امضاء الشاهد : ....

التاريخ : ....

اسم الشاهد بالاحرف الغليذلة (أو المطبعية)

....

امضاء القائم بالإستقصاء : ....

التاريخ : ....

اسم القائم بالإستقصاء / مساعده بالأحرف الغليظة

....

الأحرف الأولى للفرد رقم الفرد الأحرف الأولى لاسم الشاهد

ملحق ب : (مطبوعة هبة تطوعية

عنوان الدراسة : العلاج الموضعي لداء اللايشمانيات بواسطة WR 279396 :
دراسة المرحلة الثانية

إني موافق على إستعمال الطفيليات التي تمّ تجميعها / أو عزلها خلال الدراسة في دراسات وبحوث بيولوجية طبيّة اخرى.
إن أي منتوج طبي (أو دواء) يتم الحصول عليه إنطلاقا من الأعمال الجارية ومن إستعمال هذه العينات يمكن تسويقه لذا فإنه لا يحق لي أن اطالب بأي مقابل ما يلي أتحكم من خلاله في مواصلة هذا العمل وانجازه

امضاء المريض (المتطوّع) ....

التاريخ : ....

الاسم بالأحرف الكبيرة للمريض (المتطوّع) :

إمضاء الشاهد : ....

التاريخ : ....

اسم الشاهد بالأحرف الكبيرة

امضاء القائم بالبحث / المحقّق : ....

التاريخ : ....

إسم القائم بالبحث / المحقق بالأحرف الكبيرة :..

الجزء - ب - مطلوب تعميره من القائم بالإستقصاء (المتقصّي)

(تعليمات بخصوص عناصر من الموافقة الواعية / عن إطلاع)
المرغوب تقديم توضيح مفصّل وفقا للملحق ج - AR40-38 أو AR70-25

الملحق - أ - 3 (إقرار خدّاي بعد القسم لمتطوّع - الجزء ب

توضيح مطبوعة الموافقة

عنوان الدراسة
" العلاج الموضعي لداء اللايشمانيات الجلدي بواسطة WR279396 - دراسة المرحلة II بالعالم القديم"
المتبنّون المشتركون : مكتب رئيس الاطباء (OTSG) ومعهد باستور بباريس
- المتقصّون / القائمون بالإستقصاء المشتركون / الاطبّاء المسؤولون عن البحث :
- الدكتور بيار بوفي - المركز الطبّي لمعهد باستور باريس
الهاتف ....الفاكس ....
- الدكتور عفيف بن صالح معهد باستور - تونس
الهاتف ....الفاكس ....

معلومات خاصّة بالمشاركة

إنه يشتبه في إصابتك بداء اللايشمانيات الجلدية
وتسبّب الإصابة بهذا الداء الجلدي الطفيليات المنقولة عبر عضّات ذبابة الرّمل (القرقس)
تغزو الطفيليات خلايا الجلد وتحدث قرحة (أو تقرّحا)
إنّ داء اللايشمانيات الجلدي يتميّز بانه يشفى ذاتيا لكنّ الشفاء الذاتي قد يتطلب عدّة أشهر وفي ذلك الوقت قد ينتشر المرض إلى أجزاء أخرى من الجلد ونادرا ما تنتشر الإصابة الى حدّ الانف.

يتمّ العلاج العادي بواسطة حقن "الانتيمون" في شكل انتيمونات المغلومين (غلوكانتتم) وبالرغم من انّ الدواء فعّال بصفة عامّة فإن سلبيات العلاج بالانتيمون هي انه يتعيّن إعطاء المريض خمسة حقن مؤلمة داخل الآفة (موقع الآفة) على الأقل وأن هناك بعض أوجه القصور (الإجهاد / الضعف) والانتكاس كما انه عند حقنها داخل العضلات فإنّه قد ينتج عنها خطرا تسمّم بالقلب والكبد والمعثكلة.

وإن الهدف الاساسي من هذه الدراسة هو مقارنة WR279396 مع دواء يعطى للمريض على سبيل الإرضاء (دون تاثيرات سلبية) ومعاينة ما إذا كان وضع مرهم على الآفة يمثل فعالية حقن الغلوكانتيم.

كما أنه من المهمّ جدّا معاينة ما إذا كان المرهم مؤلما أو إذا ما كان يتسرّب الى داخل الدمّ وبما أنّ الحيّات تلتئم تلقائيا وأنّه علينا أن نعرف كم من حالات الشفاء راجعة بشكل خاص الى المرهم، فإن هذه التجربة ستكون في صيغة التجربة العمياء المضاعفة المراقبة بالدواء الممنوح للمريض لإرضائه وسيقع تعيينك بمحض الصدفة (لتفاقا) إما بالمجموعة الخاضعة للمادة النشيطة أو بمجموعة الدواء المخصّص لإرضاء المريض.

وهذا يعني أنّ 50 % من المتطوعين سيوصف لهم مرهم بلا مكوّن نشيط (دواء معدّ لإرضاء المريض)
في حين أنّ 50% لآخرين من المتطوعين سيحصلون على المرهم النشيط (WR279396) ولا أنت ولا طبيبك سيعلم أيّ المرهمين إستعملت (وهذا ما يسمى (الأعمى المضاعف - التجربة العمياء المضاعفة) ذلك أن الدواء المعدّ لإرضائك والمرهم ذي المكوّن النشيط WR279396 يبدوان متشابهين تماما.

وبما أنّه طلب منك أن تشارك في بحث دراسي طبّي فإنه من المهمّ جدّا أن تدرك المبادئ العامّة التالية التي تنطبق على كل من يساهمون في دراساتنا سواء أكانو متطوّعين أصحّاء عاديين أو مرضى متطوّعين.

1- إن مساهمتك طوعية كليّا
2- يمكن أن تنسحب من هذه الدراسة أو من أي جزء من الدراسة في أي وقت. وإن رفض المشاركة لن يعرّضك لأيّة عقوبة أو حرمان من المنافع التي يحقّ لك التمتع بها.
3- بعد قراءة التوضيحات يتعيّن عليك أن تطرح أيّة أسئلة تحتاجها لكي تفهم بوضوح طبيعة الدراسة.

مقدّمة :

لقد طوّر الباحثون بمعهد والتر ريد للبحوث التابع للجيش بالولايات المتّحدة الأمريكية مرهما بإمكانه علاج داء اللايشمانيات الجلدية. إن هذا الدواء المسمّى WR279396 قد شفى من مرض اللايشمانيات الجلدية حيوانات مخبرية. إن المكوّنات النشيطة بـ WR279396 هي إثنتان :

كيريت الأمينوغليكوسيد باروموميسين (15%) وكيريت الجنتاميسين (0,5%) في قاعدة (AQIC) وخلال الدراسات السريرية الأوّلية إتضح أن إستخدام WR279396 على مدى 20 يوما قد لاقى قبولا جيدا لدى متطوّعين اصحّاء من البشر بالرغم من ظهور بعض الإحمرار الموضعي. وإننا نرغب في إعطاء WR279396 الى مرضى مصابين بداء اللايشمانيا الجلدية لنرى ما إذا كانت كمية WR279396 المستخدمة لدى المتطوّعين العاديين قادرة على شفاء المرضى. فإذا كان الدواء فعالا فإن المنفعة بالنسبة لك تتمثل في انّه سيقع علاجك بدواء موضعي ينتظر أن يكون ذاسميّة خفيفة عوضا عن علاجك بحقنة مؤلمة بالانيتموان السمّي، ولقد تمت مراجعة هذه الدراسة من طرف اللجنة الإستشارية باريس كوشين لحماية الاشخاص في البحوث البيولوجية الطبية ووافقت عليها في..........

الغرض من الدراسة :

تهدف هذه الدراسة الى :

1) تحديد ما إذا كان WR279396 قادرا على معالجة داء اللايشمانيات الجلدية

2) مزيد تحديد مدى تقبّل الدواء من طرف المرضى المصابين بداء اللايشمانيا الجلدية في العالم القديم.

الإجراءات التي سيقع إتّباعها:

إذا ما وافقت على المشاركة في هذه الدراسة فإنه سيقع أخذ عينة من حبتك أو حباتك بواسطة إبرة و/أو بواسطة الكشط أو قطع جزء صغير من الجلد في حجم نصف فسآخ قلم عادي بعد تحييد الأعصاب بحقنها بمقدار صغير من مخدّر موضعي وذلك للتثبت من حقيقة إحتوائها على طفيليات اللايشمانيا.

وإذا ما كانت الطفيليات موجودة فإنه بإمكانك أن تشارك في هذه الدراسة ويكوّن لك ملف طبّي ويجرى لك فحص بدني وتؤخذ لك عينات من دمك ومن بولك للتاكد من أنك أهل لأن تخضع لهذه الدراسة. وسيجرى لك كذلك إختبار سمعي للتاكد من كون حاسّة السمع عادية لديك. وعندما يوضع لك مرهم (إما WR279396 أو دواء يعطي لمجرّد إرضاء المريض) على كل حكة مرّتين في اليوم لمدّة 20 يوما. ولن يكون لك الإختيار بخصوص أيّ من المراهم سيقع إستعماله، فكل الحبات ستدهن بالمرهم المخصّص لك. وسيضع لك المرهم موظفو الدراسة. وسيقع سؤالك يوميا لتحديد ما إذا كان المرهم يضرّ ببشرتك العادية المحيطة بالحبة أو يضرّ بالحبة نفسها وفي نهاية مدّة العشرين يوما من العلاج وبعد مرور ثلاثين يوما (30) وثمانين يوما (80) وستّة أشهر من إنتهاء العلاج يقع فحص حبتك (حباتك) للنظر فيما إذا تحسّنت أو شفيت تماما.

وإذا ما لم تتحسّن حبتك (حبانك) في خلال ثلاثين (30) يوما أو لم تشفى بعد إنقضاء ستة أشهر (6) فإنه يقع شطبك من البروتوكول (الإتفاق ويقدّم لك أفضل علاج وفقا لما يقرّره طبيبك ومن المتوقع أن يكون هذا العلاج الغلوكانتيم.

وفي بداية العلاج ونهايته يقع أخذ عينة من دمك (5 مل) للتأكّد من عدد الخلايا بدمك وللقيام بفحوص كيميائية عليه (5 مل).

وسيقع إستعمال بلازما دمك لتحديد كمية الدواء WR279396 التي تدور بدمك. وسيقع إستخدام دمك للتجارب المبيّنة أعلاه فحسب وبطبيعة الحال ولعلمك فإن هناك إحتمالا بأن تستخدم الطفيلية الحاصلة بواسطة زرع عينات من حبتك التي تقدّمها ضمن هذه الدراسة في دراسات وبحوث أخرى خاصّة بداء اللايشمانيا وسيقع مدّك بجدول يفسّر لك الإجراءات التي سيقع القيام بها طيلة مدّة الدراسة وتوقيت إجرائها (الجدول عدد 2 - تونس).

وسيقع أخذ صور عن حبتك قبل العلاج وفي نهايته وبعد مرور 30 يوما و 80 يوما وستّة أشهر من إنتهاء العلاج . وسيقع إخفاء كل ما من شأنه أن تعرّف بك في الصور وسيقع التعريف بالمرضى من خلال الأحرف الأولى وليس الأسماء.

الدواء المستخدم للتقصّي والبحث :

WR279396 هو دواء إستثنائي وغير موافق عليه من الإدارة الأمريكية للأغذية والعقاقير (الأدوية) للإستعمال العام ومع ذلك فقد سمحت هذه الإدارة بإستخدامه في هذه الدراسة البحثية ولحدّ الآن تمّ إستعمال هذا الدواء على 57 فردا فحسب.

مدّة المشاركة المتوقّعة : سبعة (7) أشهر

المجازفات والأخطاء والإزعاج والضمانات

يتعيّن عليك أن تكون واعيا وعالما بالمجازفات التي قد تعرضك اليها هذه الدراسة وبطريقة التحكّم في هذه المجازفات.

1) إن الدواء قد يهيّج (أو يثير) بشرتك سنراقب ذلك بفحصك وبسؤالك يوميا

2) إنّ الدواء قد يمتصه دمك وقد يؤثر على سمعك أو على كليتيك سنتحكم في ذلك بإجراء إختبارات على سمعك وعلى دمك.

3) وبما أن الدواء علاج موضعي فإن الحبات الموضعية قد تشفى الا أن المرض قد يعاود الظهور على بشرتك أو (وهذا نادر) في أنفك وعليك أن تعي بأن هذه المظاهر تقع مهما كانت طريقة علاجك : (فقد تعاود التهيجات الجلدية الظهور بعد شفاء الحبة بواسطة العلاج بالغلوكانتم كما يمكن أن تعاود الإصابة بالأنف الظهور بعد شفائها عن طريق إستعمال العلاج بالغلوكانتيم أو من دون علاج وعليك انت وطبيبك أن تراقب عودة التهيجات الجلدية خلال الاشهر الستة الموالية لإستعمال الدواء (أشهر المتابعة الستة)

إن كمية الدم المأخوذة لن تسبب أية أعراض جانبية غير مرغوب فيها.

إن العواقب المزعجة لسحب الدم هي الالم الخفيف والكدمة. إنّ اقل من أربعة ملاعق شاي (4) (20 مل) سيقع سحبها قبل العلاج وفي وسط مدّة العلاج وفي نهاية العلاج وثلاثين يوما (30) بعد نهاية العلاج. لا يمكن أن يسحب منك في المرّة الواحدة ما يزيد عن 20 مللتر (قرابة ملعقة واحدة طاولة) من الدمّ. وخلال كامل مدّة الدّراسة فإنّ الكمية الجملية للدمّ الذي سيؤخذ منك ستكون تقريبا 75 مللتر (حوالي 7 ملاعق طاولة)

هناك مسالة اخرى قد تزعجك وهي انه إذا ما شاركت في الدراسة فإنّه ينتظر منك ان تكون جاهزا لإعادة فحصك 30 يوما و 80 يوما وستة اشهر بعد إنتهاء العلاج. وبالرغم من أنّ هذا قد يكون صعبا بالنسبة لك فإنه من المهمّ التأكد من عدم معاودة المرض للظهور لديك.

إنّ مساهمتك في هذه الدراسة تمنعك من التبرّع بدمك خلال مدّة العلاج والمتابعة.

وكما هي الحال في أي دراسة فإن العلاج أو الإجراءات التابعة له قد تسبب مخاطر لا يمكن توقعها مسبّقا.

إجتناب الحمل :

الإمتناع عن الحمل

يتعيّن عليك أن تتجنّبي الحمل خلال مدّة الدراسة ولمدّة شهر على الاقل بعد المشاركة في الدراسة ولإجتناب الحمل يتعين عليك إمّا الإمساك عن العلاقات الجنسية أو إستخدام وسيلة من وسائل منع الحمل. وبإستثناء إزالة الرحم جراحيا فإن وسائل منع الحمل مثل إستعمال الواقيات من الحمل مثل الحاجز الواقي من الحمل أو حاجز عنق الرحم أو أقراص منع الحمل أو اللولب الرحمي أو المراهم القاتلة للحوينات المنوية ليست فعّالة نهائيا وكليا في الوقاية من الحمل.

<u>الإحتياطات المطلوب من الأفراد الخاضعين للدراسة</u>
<u>الإلتزام بها قبل الدراسة وخلالها :</u>

إن بعض العقاقير وبعض اللقاحات قد تتدخّل في طريقة تعافيك من داء اللايشمانيا. لذا فالمطلوب منك عدم إستخدام أيّة أدوية (بما في ذلك الأدوية التي لا تحتاج لوصفة طبية مثل الاسبرين والأسيتامينوفان (تيلينول) - (المعروف بالباراسيتامول - دوليبران / إفيرالغان) أو الخضوع لأي تلقيح أسبوعين قبل بدء الدراسة البحثية وخلال الدراسة وأسبوعين على الاقل بعد الجرعة الأخيرة من العلاج الموضعي، دون النقاش في ذلك مع أحد من الأطباء المباشرين للدراسة. فإذا تمّ إعلامنا قبل حصولك على مثل هذا العلاج فإنه يمكننا أن نوصي بدواء مخصوص قادر على علاجك دون أن يتداخل مع الدراسة.

وبقطع النظر وإذا ما وصفت لك أدوية أو تلاقيح أو إذا ما إعتزمت تناول أيّة أدوية خلال مجرى الدراسة فإنه من المهمّ أن يكون الطبيب المعني بالدراسة على علم بذلك.

<u>إجراءات وقائية :</u>

لتجنب المجازفات والتقليل من حدّة المخاطر المتصلة بهذه الدراسة ستخضع لمراقبة (دقيقة) وسيكون أحد الأطباء المشرفين على الدراسة دوما مستعدّا للتحدّث معك على أحد أرقام الهواتف المبينة أسفله.

<u>المنافع</u> : إذا ما خضعت للعلاج بـ WR279396 أو (لعلاج منح لك لإرضائك) وشفيت فإنك تكون قد شفيت دون إستعمال الحقن. فإنّ منفعة غير مباشرة لك تتمثل في معرفة كونك ساعدت دراسة علمية سيمتد نفعها الى أشخاص آخرين قد تلحقهم الإصابة في المستقبل.

فإذا ما لم يشفك WR279396 (أو الدواء الممنوح لك لإرضائك) فإنه سيتم علاجك من طرف طبيبك بأحسن طريقة بلغ إليها علمه وعلى الارجح بفضل دواء (أو عقار) عادي آخر لداء اللايشمانيات، الغلوكانتيم مثلا.

<u>العلاج البديل</u> : إذا ما إخترت عدم المشاركة في هذه الدراسة فإن طبيبك الشخصي سيعالجك بإجراءات عادية قد تتمثل في الغلوكانتيم بواسطة الحقن إما مباشرة داخل الحبة أو داخل العضلات حسب عدد الحبات.

ضمان الإبقاء على سريّة هوية المتطوّع :

إن كل المعطيات الخاصّة بك والتي سيتمّ تجميعها خلال الدراسة ستجعل في ملفات بلا هوية وستبقى الملفات المتصلة بمشاركتك بإعتبارك فردا خاضعا للبحث، في كنف السريّة. وقد يراجع الملفات ممثلون عن معاهد باستور بكل من باريس وتونس وعن وزارتي الصحة بفرنسا وتونس وعن الإدارة الأمريكية للأغذية والأدوية أو ممثلون عن الجيش الامريكي بإعتبار ذلك جزءا من مسؤوليتهم في الإشراف على البحث. وسوف لن يستخدم اسمك في أي تقرير ينبثق عن هذه الدراسة. القانون الخاص بالإعلامية وبالملفات والحريات " قانون 2002 " فإنه يكون لك الحق في مراجعة ملفات الدراسة التي خضعت لها إن مباشرة أو عن طريق طبيب تختاره.

وإن العينة المخصّصة لإستعمالها مستقبلا يقع التعرّف عليها فقط بواسطة رقم المتطوّع.

سحب المشاركة من طرف القائم بالإستقصاء :

يجوز للقائم بالإستقصاء أن يسحبك من المشاركة في هذا البحث الدراسي حتى ولو رغبت في مواصلته إذا مابرزت ظروف تؤيد هذا التصرّف. إذا ماتعرّضت لما يلي مثلا :

1) تطوّرت لديك ظروف صحية من شأنها أن تجعل من إستمرارك في المشاركة في هذه الدراسة خطرا عليك

2) قد تحلّ ظروف أخرى تجعل من مشاركتك مضرّة بصحتك وسيتّخذ القائم بالإستقصاء القرار ويعلمك به إذا لم يبد ممكنا لك أن تستمر في الخضوع للدراسة. وقد يتّخذ القرار إمّا لحماية صحّتك وسلامتك، أو لأن جزء من خطة البحث وضع حدّ لمشاركة متطوّعين تظهر لديهم بعض الملابسات الدلبية.

إنسحاب المريض بنفسه :

إذا ما رغبت في الإنسحاب من الدراسة، الرجاء الإتصال بأحد القائمين بالإستقصاء

تكلفة المشاركة :

إن العناية الطبية المتّصلة بمشاركتك في هذه الدراسة سيتكفّل بها المتبنّون لهذه الدراسة السريرية.

الدفع بعنوان المشاركة :

يجب أن لا تتحمل ماديا تبعات المشاركة في هذه الدراسة ويتعيّن أن تغطي الدّراسة عند الإقتضاء مصاريف تنقّلك من عملك أو بيتك الى موقع الدراسة وخلال الساعات التي لا تعمل فيها بسبب مشاركتك في الدراسة وإن الموظفين الإداريين للدراسة سيدفعون لك في نهاية الدراسة أو على مراحل خلال مدّة الدراسة إذا إقتضى الأمر ذلك.

متطلبات متصلّة بقاعدة المعطيات لتسجيل المتطوّعين :

تقتضي سياسة (مؤسسة للبحث الطبي والمعدّات الطبية التابعة للجيش الأمريكي) أن مطبوعات المعلومات (USAMRDC FORM 60 R) يتعين تعميرها بخصوص كل المتطوعين المشاركين في البحث لإدخالها الى قاعدة المعطيات الخاصّة بتسجيل المتطوعين بالقيادة.

وإن المعلومات المطلوب إدخالها بقاعدة المعطيات السرية هذه بخصوص الأفراد تتضمّن اسمك وعنوانك ورقم تأدينك الإجتماعي (ورقم بطاقة التعريف التونسية) واسم الدراسة وتواريخها. وإن الغاية من قاعدة المعطيات مزدوجة : أوّلا الإجابة بكل إستعداد عن الأسئلة الخاصّة بمشاركة فرد ما في البحث المتبنى من مؤسسة للبحث الطبي والمعدّات الطبية التابعة للجيش الأمريكي وثانيا التأكد من كون مؤسسة للبحث الطبي والمعدّات الطبية التابعة للجيش الأمريكي قادرة على ممارسة التزامها بتأمين كون المتطوّعين للبحث قد وقع تنبيههم على النحو الملائم (واجب التنبيه) الى المخاطر، وتمكينهم من معلومات جديدة كلّما أصبح ذلك متاحا. وسيقع خزن المعلومات بمؤسسة للبحث الطبي والمعدّات الطبية التابعة للجيش الأمريكي لمدّة 75 سنة كحدّ أدنى.

إكتشافات جديدة هامّة :

إن أيّة معلومة هامّة قد تهمّ صحّتك أو إستعدادك لمواصلة المشاركة يتم التوصل اليها خلال الدراسة توضع على ذمّتك وإذا ما قدمت لك معلومات جديدة يتعين معاودة الحصول على موافقتك على مواصلة المشاركة في هذه الدراسة.

عدد المتطوّعين في هذه الدراسة :

إن مائة مريض سيقع تسجيلهم ضمن هذه الدّراسة .

العناية الطبية نتيجة للإصابة بأضرار أو المرض

إن وزارة الدفاع الأمريكية تموّل مشروع البحث هذا، فإذا ما تعرّضت لإصابة كنتيجة مباشرة للمشاركة في مشروع البحث هذا فإنه ستتوفّر لك الرعاية الطبية، بدون أن تتحمّل أيّة مصاريف، بخصوص تلك الإصابة.

وإن المتيني المشترك لهذه الدراسة وهو معهد باستور بباريس مؤمّن وكذلك معهد باستور تونس، يسيغطي التأمين أية أضرار محتملة قد تنشأ عن مشاركتك في الدراسة وهذا لا يعفي منظمي هذا البحث من مسؤولياتهم وتحتفظ انت بحقوقك القانونية التي يضمنها لك قانون هوريت المؤرخ في 20 ديسمبر 1988. وعليك أن تناقش هذه المشكلة بعمق مع القائم بالإستقصاء الرئيسي و/أو شريكه قبل أن تنضمّ الى هذه الدراسة.

التعريف بالقائمين بالإستقصاء :

في حالة حصول ضرر ناجم عن البحث أو إذا ما حصلت لك مضاعفات غير مرغوب فيها الرجاء الإتصال فورا بأحد القائمين بالإستقصاء المذكورين أسفله. فالرجاء الإتصال بأحد هؤلاء المذكورين أسفله إذا ما كانت لك أيّة أسئلة بخصوص هذا البحث الطبّي.

الدكتور عفيف بن صالح	الهاتف : 216-1-792-429<br>فاكس : 216-1-791 833
أو الدكتور بيار بوذي	الهاتف : 17 38 61 40 01<br>فاكس : 33 1- 45688218
أو الدكتور ماكس غروغل	الهاتف : 301-295-7655<br>فاكس : 301-295-7655
أو الدكتور جوناثان د. برمان	الهاتف : 301-319-9561

وللحصول على معلومات أو إجابات عن تساؤلات بخصوص حقوقك باعتبارك فردا خاضعا للبحث يمكنك الإتصال بالدكتور و رئيس إدارة خدمات المرضى - المركز الطبي لمعهد باستور بباريس أو بالدكتور الهاشمي الوزير أستاذ في علم المناعة الطبية معهد باستور - تونس.

وإذا ما أصبت بتوعك أو مرض خلال الدراسة الرجاء مراجعة المركز الصحي حيث تمّ قبولك للمشاركة في هذه الدراسة أو التحدث الى الدكتور عفيف بن صالح (216-1-792429)

وإذا ماكان أي جزء من إيضاح الموافقة هذا غير مفهوم أو يتطلب التوضيح فإنه عليك أن تسأل القائم بالإستدعاء عنه قبل التوقيع.

الأحرف الأولى للفرد الخاضع للدراسة	رقمه	الأحرف الأولى لإسم الشاهد

لقد قرأت المعلومات المقدّمة أعلاه ومنحت أي فرصة طرح الأسئلة وتلقيت أجوبة عن اسئلتي بشكل أرضاني كما منحت نسخة من هذه المطبوعة.

إني أوافق   لا أوافق   (أشطب المربع واكتب الأحرف الأولى لاسمك)
على إضافة هذه المطبوعة الى ملف علاجي الطبي بصفتي مريضا خارجيا.

اسم المتطوع بالأحرف الناليطة :

امضاء المتطوع	التاريخ	امضاء الوصيّ القانوني (إذا كان المتطوّع قاصرا)
العنوان الدائم للمتطوّع	اسم الشاهد	مطبوعا
		التاريخ

Table 4. Volunteer - Time and Event Schedule - Paris
(To be given to the Volunteer on day -1)

Day	Date	Approx. Time	Events
-1		4 hr	Consent, history and medical exam (physical & dermatology), hearing test, Romberg test, blood draw (CBC, AST, ALT, creatinine, glucose, Na, K), urine specimen, clinical evaluation & measurement of lesion(s), photograph lesion, parasitology-diagnosis, pharmacokinetics-blood & urine
10		1 hr	Hearing test, Romberg test, blood draw (creatinine), pharmacokinetics-blood & urine
20		2 hr	Hearing test, Romberg test, blood draw (creatinine), urinalysis, pharmacokinetics-blood & urine, clinical evaluation & measurement of lesion(s), photograph lesion, parasitology-diagnosis
30#		1 hr	Evaluation & measurement of lesion(s), photograph lesion
80#		1 hr	Evaluation & measurement of lesion(s), photograph lesion
6 mon#		1 hr	Evaluation & measurement of lesion(s), photograph lesion

Note: **Drug Therapy** Days 1 through 20
Local Toxicity Days 1 through 20

# Follow-up refers to time after end of therapy

Tableau 4. Volontaire – Déroulement de l'étude- Paris

(A donner au volontaire le jour -1)

Jour	Date	Temps Approximative.	Evénements
-1		4 hr	Consentement, antécédents et examen médical (physique & dermatologique), test d'audition, test de Romberg, prise de sang (NFS, SGOT, SGPT, créatinine, glucose, Na, K), analyse d'urine (Ph, leucocytes,albumine,sucre, nitrites), évaluation clinique & mesure de lésion(s), photographie de la lésion, diagnostic parasitologue-, pharmacocinétique du sang et d'urine
10		1 hr	Test d'audition, test de Romberg, prise de sang (créatinine), pharmacocinétique du sang et d'urine
20		2 hr	Test d'audition, test de Romberg, prise de sang (créatinine), analyse d'urine, évaluation clinique & mesure de lésion(s), photographie de la lésion, diagnostic parasitologue
30#		1 hr	Evaluation & mesure de lésion(s), photographie de la lésion
80#		1 hr	Evaluation & mesure de lésion(s), photographie de la lésion
6 mois#		1 hr	Evaluation & mesure de lésion(s), photographie de la lésion

Note : **Traitement avec le WR279396 :** Jours 1 à 20
~~**Toxicité locale :** Jours 1 à 20~~

# Suivi après la fin de traitement

Table 5. Volunteer - Time and Event Schedule - Tunisia

(To be given to the Volunteer on day -1)

Day	Date	Approx. Time	Events
-1		4 hr	Consent, history and medical exam (physical & dermatology), hearing test, Romberg test, blood draw (CBC, AST, ALT, creatinine, glucose, Na, K), urine specimen, clinical evaluation & measurement of lesion(s), photograph lesion, parasitology-diagnosis
10		1 hr	Hearing test, Romberg test, blood draw (creatinine
20		2 hr	Hearing test, Romberg test, blood draw (creatinine), urinalysis, clinical evaluation & measurement of lesion(s), photograph lesion, parasitology-diagnosis
30#		1 hr	Evaluation & measurement of lesion(s), photograph lesion
80#		1 hr	Evaluation & measurement of lesion(s), photograph lesion
6 mon#		1 hr	Evaluation & measurement of lesion(s), photograph lesion

Note: **Drug Therapy** Days 1 through 20
Local Toxicity Days 1 through 20

# Follow-up refers to time after end of therapy

Table 5. Planning temps et évènements - Tunisie
(A donner au volontaire au temps -1)

Jour	Date	Temps Approx.	Evénements
-1		4 hr	Consentement, antécédents et examen medical (physique & dermatologique), test auditif, test de Romberg, Sang (NFS, SGOT, SGPT, creatinine, glucose, Na, K), specimen d'urine, evaluation clinique & mesure de(s) lésion(s), photographie lésion, diagnostic parasitologique
10		1 hr	test auditif, test de Romberg, Sang (créatinine)
20		2 hr	test auditif, test de Romberg, Sang (creatinine), specimen d'urine, evaluation clinique & measure de(s) lésion(s), photographie lésion, diagnostic parasitologique
30#		1 hr	Evaluation & measure de(s) lesion(s), photographie lésion
80#		1 hr	Evaluation & measure de(s) lesion(s), photographie lésion
6 mon#		1 hr	Evaluation & measure de(s) lesion(s), photographie lésion

Note: **Application du médicament** Jours 1 à 20
Toxicité locale Jours 1 à 20

# Suivi réfère au temps après la fin du traitement

Appendix B (English Volunteer Donation Form)

TITLE OF STUDY: "TOPICAL TREATMENT OF CUTANEOUS LEISHMANIASIS WITH WR 279396: A PHASE 2 STUDY.

I give my consent for the use of the parasite collected/isolated during the study for other Biomedical research studies. Any product resulting from the work carried out using these samples may be commercialized. I claim no financial control over the carrying out of this work.

Patient's (Volunteer's) signature: ______________________________

Date: ____________________

Patient's (Volunteer's) printed name: ___________________________

Witness's signature: ____________________________________

Date: ____________________

Witness's printed name: __________________________________

Investigator's signature: __________________________________

Date: ____________________

Investigator's printed name: ________________________________

Annexe B (Formulaire en anglais de Don Volontaire)

TITRE DE L'ETUDE : « TRAITEMENT TOPIQUE DE LA LEISHMANIOSE CUTANEE AU MOYEN DU WR 279396 : ETUDE DE PHASE 2 »

Je donne consentement pour que les parasites recueillis/isolés pendant cette étude soient utilisés pour d'autres études de recherche biomédicale. Les travaux réalisés à partir de ces prélèvements pourront être commercialisés. Je ne pourrai prétendre à aucune contre-partie financière sur l'exploitation de ces travaux.

Signature du patient (Volontaire): ______________________________

Date: ______________________________

Nom du patient (Volontaire) en lettres majuscules : ______________________________

Signature du témoin: ______________________________

Date: ______________________________

Nom du témoin en lettres majuscules: ______________________________

Signature de l'investigateur: ______________________________

Date: ______________________________

Nom de l'investigateur en lettres majuscules: ______________________________

Appendix C (Certification of Translation)

I certify that the Ethical Review Forms in Section 10.1 through 10.6 of this protocol titled, "Topical Treatment of Cutaneous Leishmaniasis with WR 279396: A phase 2 Study in the Old World." Log No. __________, Version ________, dated ________________ are an accurate and true translation.

Signed Name _____________________ Date

Printed Name _____________________ Date

Address _____________________

Phone _____________________ Fax

C'est votre traduction !

CERTIFICATION OF TRANSLATION

MRS. COURTIN, a translator residing at:

ART INTERNATIONAL 26, rue Carnot 95 410 Groslay France

declares:

1. That I know well both English and French languages;

2. That I translated the attached document identified as:

Topical Treatment of Cutaneous Leishmaniasis with WR 279396: A Phase 2 Study in the Old World

from English to French.

DATE : **9th September 2002**

SIGNATURE

B.P. 18
95410 GROSLAY
Tél : 01.39.34.70.70
Fax : 01.39.34.70.77

Appendix C (Certification of Translation)

I certify that the Ethical Review Forms in Section 10.1 through 10.6 of this protocol titled, "Topical Treatment of Cutaneous Leishmaniasis with WR 279396: A phase 2 Study in the Old World." Log No. __________, Version ________, dated ________________ are an accurate and true translation.

Samia BESBES — June 13th, 2002
Signed Name — Date

SAMIA BESBES — June 13th, 2002
Printed Name — Date

63, Rue de Yougoslavie Tunis 1000
Address

+71 340 580
Phone — Fax

Samia Besbes

Pour Traduction
exacte et conforme
délivrée par nous SAMIA BESBES,
interprète de l'O.N.U (New York) installée
en TUNISIE
TUNIS, le ____________
L'Interprète.

ملحق ج : (إشهاد بصحّة التعريب)

أشهد أن مطبوعات المجلّة الأخلاقية الموجودة بالفقرات 1 الى 6 من الفصل العاشر (10) من بروتوكول الإتفاق هذا الحامل لعنوان " العلاج الموضعي لداء اللايشمانيات الجلدية بواسطة WR279396 دراسة للمرحلة II بالعالم القديم" كتاب عدد.............الطبعة :............ ....المؤرّخة في ....تعدّ ترجمة صادقة وصحيحة.

الامضاء الاسمي سامية بسباس التاريخ 13 جوان 2002

الاسم الكامل بالأحرف الكبرى سامية بسباس التاريخ 13 جوان 2002

العنوان : 63 نهج يوغسلافيا تونس

رقم الهاتف : 71340580

رقم الفاكس :..

سامية بسباس
مترجمة محلفة
63، نهج يوغسلافيا - تونس
الهاتف: 340.580

سامية بسباس

Certification of Translation

I certify that the file titled "Table 4. Volunteer - Time and Event Schedule – Paris", To be given to the Volunteer on day –1, is an accurate and true translation.

	02/12/02
Signed Name	Date
Pierre BUFFET	20/12/02
Printed Name	Date

28, rue du docteur Roux, 75724 Paris Cedex 15

Address

+33 (0)1 45 68 81 15	+33 (0)1 40 61 30 19
Phone	Fax

Certification of Translation)

I certify that Table 5 of this protocol titled, "Topical Treatment of Cutaneous Leishmaniasis with WR 279396: A phase 2 Study in the Old World." is an accurate and true translation.

[illegible] 12.12.2002

Signed Name Date

JEAN-LOUIS BELARD, M.D. 12.12.2002

Printed Name Date

MRMC / TATRC FT DETRICK, MD

Address

(301) 619 4006 (301) 619 7911

_Phone Fax

DEPARTMENT OF HEALTH AND HUMAN SERVICES<br>PUBLIC HEALTH SERVICE<br>FOOD AND DRUG ADMINISTRATION<br>**STATEMENT OF INVESTIGATOR**<br>***(TITLE 21, CODE OF FEDERAL REGULATIONS (CFR) PART 312)***<br>(See instructions on reverse side.)	Form Approved: OMB No. 0910-0014.<br>Expiration Date: September 30, 2002.<br>*See OMB Statement on Reverse.*<br><br>NOTE: No investigator may participate in an investigation until he/she provides the sponsor with a completed, signed Statement of Investigator, Form FDA 1572 (21 CFR 312.53(c)).

1. NAME AND ADDRESS OF INVESTIGATOR

LTC Max Grogl
Division of Communicable Diseases and Immunology
Walter Reed Army Institute of Research
503 Robert Grant Avenue
Silver Spring, MD 20910-7500

2. EDUCATION, TRAINING, AND EXPERIENCE THAT QUALIFIES THE INVESTIGATOR AS AN EXPERT IN THE CLINICAL INVESTIGATION OF THE DRUG FOR THE USE UNDER INVESTIGATION. ONE OF THE FOLLOWING IS ATTACHED.

☐ CURRICULUM VITAE ☐ OTHER STATEMENT OF QUALIFICATIONS

3. NAME AND ADDRESS OF ANY MEDICAL SCHOOL, HOSPITAL OR OTHER RESEARCH FACILITY WHERE THE CLINICAL INVESTIGATION(S) WILL BE CONDUCTED.

Centre de Recherche Clinique de l'Institut Pasteur,
25-28 rue du Dr. Roux, 75015 Paris, France

Institut Pasteur de Tunis
13 Place Pasteur BP, 74 Belvedere 1002, Tunis, Tunisia

4. NAME AND ADDRESS OF ANY CLINICAL LABORATORY FACILITIES TO BE USED IN THE STUDY.

Clinical Laboratory
Centre Médical de l'Institut Pasteur,
28 Rue du Docteur Roux, 75724 Paris, Cedex 15, France

Clinical Laboratory
Sidi Bouzid Regional Hospital
9100 Sidi Bouzid, Tunisia

5. NAME AND ADDRESS OF THE INSTITUTIONAL REVIEW BOARD (IRB) THAT IS RESPONSIBLE FOR REVIEW AND APPROVAL OF THE STUDY(IES).

Comite Consultatif de Protection des Personnes dans la Recherche Biomedical
Hopitel Tarnier-Cochin 39, 89 Rue d'Asess 75006, Paris, France

Comite d'ethique de l'Institut Pasteur de Tunis
13 Place Pasteur BP 74, Belvedere, 1002 Tunis, Tunisia

6. NAMES OF THE SUBINVESTIGATORS *(e.g., research fellows, residents, associates)* WHO WILL BE ASSISTING THE INVESTIGATOR IN THE CONDUCT OF THE INVESTIGATION(S).

Pierre Buffet, MD
Afif Ben Salah, MD
Gloria Morizot, RN
Anne Sophie Leguem, Chief Clinical Laboratory
Mokni Mourad, MD
Sadok Chlif, Epidemiologist
Amor Zaâtour, Senior technician,
Ben Alaya Nissaf, MD
Jlali Ali, MD
Walid Issaoui, Nurse
Ismaïl Dhay, Senior technician
Mohamed Zaher El Ahmadi, MD
Abdelkarim El Fahem, Senior technician
Zaâfouri Belgacem, Senior technician

7. NAME AND CODE NUMBER, IF ANY, OF THE PROTOCOL(S) IN THE IND FOR THE STUDY(IES) TO BE CONDUCTED BY THE INVESTIGATOR.
IND 50,098 - Topical Treatment of Cutaneous Leishmaniasis with WR279396: A Phase 2 Study in the Old World, Log No. A-97-68.

8. ATTACH THE FOLLOWING CLINICAL PROTOCOL INFORMATION:

☐ FOR PHASE 1 INVESTIGATIONS, A GENERAL OUTLINE OF THE PLANNED INVESTIGATION INCLUDING THE ESTIMATED DURATION OF THE STUDY AND THE MAXIMUM NUMBER OF SUBJECTS THAT WILL BE INVOLVED.

☐ FOR PHASE 2 OR 3 INVESTIGATIONS, AN OUTLINE OF THE STUDY PROTOCOL INCLUDING AN APPROXIMATION OF THE NUMBER OF SUBJECTS TO BE TREATED WITH THE DRUG AND THE NUMBER TO BE EMPLOYED AS CONTROLS, IF ANY; THE CLINICAL USES TO BE INVESTIGATED; CHARACTERISTICS OF SUBJECTS BY AGE, SEX, AND CONDITION; THE KIND OF CLINICAL OBSERVATIONS AND LABORATORY TESTS TO BE CONDUCTED; THE ESTIMATED DURATION OF THE STUDY; AND COPIES OR A DESCRIPTION OF CASE REPORT FORMS TO BE USED.

9. COMMITMENTS:

I agree to conduct the study(ies) in accordance with the relevant, current protocol(s) and will only make changes in a protocol after notifying the sponsor, except when necessary to protect the safety, rights, or welfare of subjects.

I agree to personally conduct or supervise the described investigation(s).

I agree to inform any patients, or any persons used as controls, that the drugs are being used for investigational purposes and I will ensure that the requirements relating to obtaining informed consent in 21 CFR Part 50 and institutional review board (IRB) review and approval in 21 CFR Part 56 are met.

I agree to report to the sponsor adverse experiences that occur in the course of the investigation(s) in accordance with 21 CFR 312.64.

I have read and understand the information in the investigator's brochure, including the potential risks and side effects of the drug.

I agree to ensure that all associates, colleagues, and employees assisting in the conduct of the study(ies) are informed about their obligations in meeting the above commitments.

I agree to maintain adequate and accurate records in accordance with 21 CFR 312.62 and to make those records available for inspection in accordance with 21 CFR 312.68.

I will ensure that an IRB that complies with the requirements of 21 CFR Part 56 will be responsible for the initial and continuing review and approval of the clinical investigation. I also agree to promptly report to the IRB all changes in the research activity and all unanticipated problems involving risks to human subjects or others. Additionally, I will not make any changes in the research without IRB approval, except where necessary to eliminate apparent immediate hazards to human subjects.

I agree to comply with all other requirements regarding the obligations of clinical investigators and all other pertinent requirements in 21 CFR Part 312.

INSTRUCTIONS FOR COMPLETING FORM FDA 1572
STATEMENT OF INVESTIGATOR:

1. Complete all sections. Attach a separate page if additional space is needed.
2. Attach curriculum vitae or other statement of qualifications as described in Section 2.
3. Attach protocol outline as described in Section 8.
4. Sign and date below.
5. FORWARD THE COMPLETED FORM AND ATTACHMENTS TO THE SPONSOR. The sponsor will incorporate this information along with other technical data into an Investigational New Drug Application (IND).

10. SIGNATURE OF INVESTIGATOR	11. DATE
[signature]	1 Oct 02

(WARNING: A willfully false statement is a criminal offense. U.S.C. Title 18, Sec. 1001.)

DEPARTMENT OF HEALTH AND HUMAN SERVICES
Public Health Service
Food and Drug Administration

DISCLOSURE: FINANCIAL INTERESTS AND ARRANGEMENTS OF CLINICAL INVESTIGATORS

Form Approved: OMB No. 0910-0396
Expiration Date: 3/31/02

TO BE COMPLETED BY APPLICANT

The following information concerning MAX GROGL (*Name of clinical investigator*), who participated as a clinical investigator in the submitted study Topical Treatment of cutaneous leishmaniasis with WR 279396 (*Name of clinical study*), is submitted in accordance with 21 CFR part 54. The named individual has participated in financial arrangements or holds financial interests that are required to be disclosed as follows:

Please mark the applicable checkboxes.

- ☐ any financial arrangement entered into between the sponsor of the covered study and the clinical investigator involved in the conduct of the covered study, whereby the value of the compensation to the clinical investigator for conducting the study could be influenced by the outcome of the study;
- ☐ any significant payments of other sorts made on or after February 2, 1999 from the sponsor of the covered study such as a grant to fund ongoing research, compensation in the form of equipment, retainer for ongoing consultation, or honoraria;
- ☑ any proprietary interest in the product tested in the covered study held by the clinical investigator; Patent
- ☐ any significant equity interest as defined in 21 CFR 54.2(b), held by the clinical investigator in the sponsor of the covered study.

Details of the individual's disclosable financial arrangements and interests are attached, along with a description of steps taken to minimize the potential bias of clinical study results by any of the disclosed arrangements or interests.

NAME	TITLE
LTC GROGL, MAX	XO, Bioproduction Facility
FIRM/ORGANIZATION: WRAIR / DCD&I U.S. Army	
SIGNATURE: [signature]	DATE: 8 August 02

Paperwork Reduction Act Statement

An agency may not conduct or sponsor, and a person is not required to respond to, a collection of information unless it displays a currently valid OMB control number. Public reporting burden for this collection of information is estimated to average 4 hours per response, including time for reviewing instructions, searching existing data sources, gathering and maintaining the necessary data, and completing and reviewing the collection of information. Send comments regarding this burden estimate or any other aspect of this collection of information to:

Department of Health and Human Services
Food and Drug Administration
5600 Fishers Lane, Room 14-72
Rockville, MD 20857

TITLE: TOPICAL TREATMENT OF CUTANEOUS LEISHMANIASIS WITH WR 279396: A PHASE 2 STUDY IN THE OLD WORLD

Appendix 2
CURRICULUM VITAE OF PRINCIPAL INVESTIGATOR

Version 7.0
IND 50,098
Log No.
17 June 2002

CURRICULUM VITAE
January 2001

LTC Grogl Max
Department of Biologics Research
Walter Reed Army Institute of Research
Walter Reed Army Medical Center
Washington, DC 20307-5100
Ph: 301-319-9359 FAX: 301-319-9180
e-mail: Max.Grogl@na.amedd.army.mil

GENERAL:

Entered Active Duty: 06 Jun 85	SSN: 237-29-2351
Assigned to WRAIR: 28 Dec 90	U.S. Passport: 900256695
MOS: 68D9C008Z	Citizenship: USA

EDUCATION:

Post-Doctoral	Harvard School of Public Health, Harvard University, Boston, 1985/ WRAIR, Washington D.C., 1984
Ph.D.	Immunology/Parasitology, Wake Forest University, 1983
M.Sc.	Microbiology, Institute of Tropical Medicine, Sao Paulo, Brazil/Andes University, Bogota, Colombia, 1976
B.Sc.	Biology, Andes University, Bogota, Colombia, 1973

LANGUAGES: Fluent in Spanish and Portuguese
Can read and understand French and Italian.

PROFESSIONAL EXPERIENCE:

1998-Present	Executive Officer WRAIR's Bioproduction Facility<br>BioSafety Officer for WRAIR/NMRC.<br>Assistant Coordinator, STEP V<br>Leishmania Skin Test Antigens Program Director<br>Leishmania Topical (WR279396) Program Director
1996-1997	U.S. Army, LTC, MS<br>Deputy Commander<br>United States Army Medical Research Unit-Brazil<br>Rio de Janeiro, R.J., Brazil
1994-1995	U.S. Army, MAJ, MS<br>Laboratory Director<br>United States Army Medical Research Unit-Brazil<br>Rio de Janeiro, R.J., Brazil

1994-Present	Visiting Professor<br>Federal University Espirito Santos<br>Vitoria, E.S., Brazil
1990-1994	Chief, Cellular Biology Section<br>Division of Experimental Therapeutics<br>Walter Reed Army Institute of Research, Washington, DC 20307
1985-1989	U.S. Army<br>Chief, Leishmaniasis Research Laboratory<br>Division of Experimental Therapeutics, WRAIR
1993-Present	Adjunct Associate Professor<br>Graduate College of Bowling Green State University, Bowling Green, OH
1993-Present	Professor<br>University New Granada, Central Military Hospital, Bogota, Colombia
1991-1994	Appointed to the Board of Directors of The American Type Culture Collection (ATCC)
1986-Present	Adjunct Assistant Professor<br>Uniformed Services University of the Health Sciences, Bethesda, MD
1986-1994	Assistant Professor, Tropical Medicine Course<br>Division of Preventive Medicine, WRAIR, Washington, DC 20307
1985	Visiting Scientist<br>Department of Tropical Public Health<br>Harvard School of Public Health, Harvard University<br>Boston, MA
1984-1985	NRC Research Associate<br>National Research Council<br>Department of Parasitology, Division of Experimental Therapeutics, WRAIR, Washington, DC 20307
1984 (Summer)	Marine Biological Laboratory<br>Woods Hole Massachusetts<br>Summer Course, Biology of Parasitism

1983-1984	Post-Doctoral Research Fellow<br>Department of Biology<br>Wake Forest University<br>U.S. Army Medical Research & Development Command
1977-1978	Director, Microbiology and Parasitology Center (LMP)<br>Bogota, Colombia (Directed all scientific research; obtained and administered grants/ Developed the Department into a service function aimed at hospital and doctors for diagnosis of tropical diseases)
1976	Coordinator, Department of Biology<br>University, Bogota, Colombia
1975-1977	Associate Professor, Parasitology<br>Department of Biology, Andes University

GRANTS FUNDED:

1995-1997	AB Foundation Grant, Kensington<br>Title: Study of parasite-host cross-reactivity in leishmaniasis.<br>Direct Cost: $35,000
1995-1997	Investigator Research Grant (ILIR), WRAIR. Title: Affect of temperature during Leishmania metacyclogenesis in sand flies on tropism.<br>Direct Cost: $35,000
	Investigator Research Grant (ILIR), WRAIR. Title: The drug sensitivity profile of free amastigotes: Development of a new model for screening drugs (Phase II).<br>Direct Cost: $105,000
1994-1996	Investigator Research Grant (ILIR), WRAIR. Title: The drug sensitivity profile of free amastigotes: Development of a new model system for screening drugs (Phase I).<br>Direct Cost: $35,000
	DoD/Department of Veterans Affairs Sharing Grant:<br>Title: Identification of the genetic factors which control tropism in Leishmania.<br>Direct Cost: $300,000

	U.S. Army Medical Research and Development Command (ILIR). Trifluralin analogs and degradative impurities with potent antiparasitic activity.<br>Direct Cost: $35,000
1993	U.S. Army Medical Research and Development Command (ILIR). The mechanism of pentostam resistance in Leishmania.<br>Direct Cost: $35,000
1992	Farmitalia. Efficacy and toxicity of paromomycin parentally against cutaneous leishmaniasis.<br>Direct Cost: $35,000.00
	SGO. Task Force L. tropica Desert Storm<br>Direct Cost: $4,800,000.00
1990	World Health Organization (Directors Initiative Funds) Efficacy of Topical Paromomycin-Methylbenzethonium Chloride in the Treatment of Simple Cutaneous Leishmaniasis in Mexico.<br>Direct Cost: $15,000.00
1987/1986/1985	USAMRDC, Grant #611101.91CLA, Molecular Biology of Drug Resistance. Direct cost: $38,000.00
1982	No. DAMD17-82-C-2229<br>Winston-Salem, NC<br>TITLE: Antigen-antibody Analyses in Leishmaniasis<br>Direct Cost; $12,000.00
1980-1981	National Science Foundation Grant #INT 8017218<br>"Antigen analysis of Trypanosoma cruzi"<br>Direct cost: $10,300.00
1976-1978	FONADE (National Fund for Development Projects) Grant<br>"Congenital Toxoplasmosis"<br>Direct cost: $33,000.00
1975-1976	Technical Aid Dutch Government Grant<br>"Standardization of serological reactions for the diagnosis of tropical parasitic diseases".<br>P.I.: Dr. C.J. Marinkelle<br>Direct cost: $55,000.

CONTRACT MONITOR:

Present - 1998	- Contracting Officer's Representative for USARDC, DAMD17-96-C 6011, Henry M. Jackson Foundation, Technical Support Services for the PBF - $3,000.000
1997-1994	-Cooperative Agreement UFES Foundation - USAMRMC Tropical Diseases - Brazil $1,300,000
1994-1990	-DAMD17-91-1001-0003/ Youngstown State University/ Isoenzyme Characterization/ $43,000
	-DAMD17-92-C-2082/ Seattle Biomedical Research Institute/Diagnostic Antigens of Leishmania/ $200,000
	-AFIP - Hematotropism of L. tropica - $120,000
	-The Salk Institute - Serology - $350,000
	-CAID - Isoenzyme Characterization - $40,000

PATENTS:

United States Patent No. 4,740,456.
Title: "Immunological methods for diagnosing neuro-cysticercosis".

Patent Application Serial No. 07/478,313
Title: "Filarizone and related thiosemicarbazones as new antifilarial agents".

Patent Application Serial No. 07/334,590
Title: "Novel derivatives of pentamidine: I) Method for treatment of malaria".

Patent Application Serial No. 07/334,730
Title: "Novel derivatives of pentamidine: II) Methods for treating leishmaniasis".

Patent Application Serial No. 07/948,533
Title: "Topical Antileishmanial".

EP Patent Application Serial No. 89119720.4
Methods for the treatment and prophylaxis of Pneumocystis carinii pneumonia and other diseases.

U.S. Patent Application - Serial No. 60/042267
Microtubule inhibitors (simple aromatic compounds) as anti-parasitic drugs.

New Use Patent Application - Submitted - 1998
WR6026 as a blood sterilizing agent.

MAJOR CONSULTANT, ADVISORY AND MEMBERSHIP ACTIVITIES:

2001 QualityAssurance Compliance, Procedures for Pharmaceutical and Biotechnology Manufacturers
HSRRB Off-Site Meeting, Ethics of DoD International research

2000 Good Clinical practice Update
GMP Training Certificate

1999 Consultant to WHO and the European Community on Topicals for the Treatment of Cutaneous Leishmaniasis - London
Good Clinical Practice Update - Certificate

1998 GMP Update - Certificate

1997 Good Clinical Practices Certificate
GMP 101 Certificate

1994-1993 Consultant to TEVA Pharmaceuticals (safety and efficacy of Paromomycin-MBCL in L. panamensis cutaneous leishmaniasis.

Consultant to the PAHO (Chagas Disease/blood transfusion)

Clinical Monitor, WHO, Safety and Efficacy of Allopurinol/Glucantime in L. panamensis CL in Colombia

1992 Advisor to OTSG, AABB, CDC and FDA in the screening and status of blood donor deferral.

Consultant and Diagnostic Program Director to OTSG Triservice Committee on Surveillance and control of L. tropica among Gulf War returnees.

Outside reviewer for Farmitalia Carlo Erba Pharmaceuticals, Aminosidineactivity and toxicity.

Consultant, Pan American Health Organization, Electronic Networking for Interamerican Microbiological Collaborative Research, Campinas, Brasil, Workshop.

1992-1994	Representative, American Society of Parasitology representative to the American Type Culture Collection Board of Directors
1991	Member, U.S. Army Medical Research and Development Command Leishmania Vaccine Steering Committee.
	Chairperson, USA/USSR Infections Diseases Teleconference, Cutaneous Leishmaniasis.
	Consultant, U.S. Navy (NAMRID), Surveillance for Drug Resistant Parasites
	Member, WRAIR Problem Definition and Assessment (PDA) Team for Operation Desert Shield
	Advisor, Armed Forces Medical Intelligence Center (AFMIC)
1990	Member, World Health Organization CHEMLEISH Steering Committee.
	Clinical Monitor, World Health Organization, Evaluation of a topical drug against cutaneous leishmaniasis, Merida, Mexico.
1989-Present	Advisor, National Research Council (#07887).
1989	Temporary Advisor, World Health Organization, Paromomycin ointment development, London, England.
	Consultant, Burroughs Wellcome Co., Efficacy and safety of allopurinol riboside and pentostam in the treatment of cutaneous leishmaniasis, Quito, Ecuador.
1988-Present	Member, U.S. Army Medical Research and Development Command Leishmania Steering Committee.
1988	Member, Epidemiology consultant service, U.S. Army Surgeon General Office, WRAIR, Guatemala City, Guatemala.
	Session Chairman, 63rd Annual Meeting of The American Society of Parasitology, Section-H (Immunology).
1987	Scientific monitor of the World Health Organization primary drug screening program in filariasis.
1987	Member, American Society of Parasitologists Special Awards Committee. Session Chairman, VIII Latin American Parasitology Congress, (Leishmaniasis).

Session Chairman, 62nd Annual Meeting of The American Society of Parasitology, Section (student paper competition).

1986-1994 Advisor in parasitic diseases to the Division of Preventive Medicine, U.S. Army Research and Development Command.

1986 Member, Scientific Advisory Committee for Parasitic Diseases, AID.

1986-1994 Director of the Walter Reed Army Institute of Research cryobank.

MANUSCRIPT REVIEWER:

--American Journal of Tropical Medicine and Hygiene
--Molecular and Biochemical Parasitology
--Infection and Immunity
--Experimental Parasitology
--American Journal of Parasitology
--Antimicrobial Agents and Chemotherapy
--Clinical Infectious Diseases
--Acta Tropica

RESEARCH INTERESTS:

--Cellular Biology of Parasitic Protozoa
--Mechanisms of Drug Resistance
--Immunoparasitology
--Epidemiology of Drug Resistance
--Host parasite relationships
--Clinical Studies
--GMP

HONORS AND RESEARCH AWARDS:

-- "A" Proficiency Designator for accomplishments in the field of Microbiology, 1999
--The Army Meritorious Service Medal, 1998
--The Army Commendation Medal, 1993
--The Army Commendation Medal, 1992
--The Army Meritorious Service Medal, 1991
--Department of the Army Research and Development Achievement Award, 1989.
--Meritorious Service Medal, 1989.
--The Army Commendation Medal, 1987.
--National Research Council, Resident and Cooperative Research Associateship Award, 1984-1985.
--Fellowship - Marine Biological Laboratory (Woods Hole, MA) - Biology of Parasitism Course, 1984.
--Elton C. Cocke Research Award, 1980.

--Fulbright-Hays Scholar, 1978-1981.
--Dutch Government Graduate Fellowship, 1972-1976.

PROFESSIONAL ORGANIZATIONS AND OFFICES:

--American Society of Tropical Medicine and Hygiene
--American Society of Protozoologists
--American Society of Parasitology
--Brazilian Microbiology Society
--Colombian Biology Society (Founding Member)
--American Association for the Advancement of Science
--Colombian Parasitology & Entomology Society (elected 1978)

PUBLICATIONS

ARTICLES:

Berman, J.D., M. King, and M. Grogl. 1989. Biochemistry of Pentostam-resistant Leishmania Am. J. Trop. Med. Hyg. 40(2):159-164.

Berman, J.D., and M. Grogl. 1988. Chemistry and biochemistry of sodium stibogluconate (Pentostam). Exp. Parasit. 67:96-103.

Hussain, R., M. Grogl, and E.A. Ottesen. 1987. IgG to antibody subclasses in human filariasis: differential subclass recognition of parasite antigens correlates with different clinical manifestations of infection. J. Immunol. 139(8):2794-2798.

Grogl, M. , M.I. Torres. 1987. Molecular Biology and Diagnosis of Leishmaniasis. The 8th Latin American Congress of Parasitology and Tropical Medicine. 1:168-175.

Anthony, R.L., M. Grogl, J.B. Sacci, and R.W. Ballou. 1987. Rapid detection of Leishmania amastigotes in fluid aspirates and biopsies of human tissues. Am. J. Trop. Med. Hyg. 37(2):271-276.

Grogl, M., E.D. Franke, P.B. McGreevy, and R.E. Kuhn. 1987. Leishmania braziliensis panamensis: protein, carbohydrate and antigen differences between log phase and stationary phase promastigotes in vitro. Exp. Parasit. 63:352-359.

Grogl, M., J.J. Estrada, G. MacDonald, and R.E. Kuhn. 1985. Antigen-antibody analyses in Neurocysticercosis. J. Parasit. 71(4):433-442.

Grogl, M., and R.E. Kuhn. 1985. Identification of relevant antigens of Trypanosoma cruzi during experimental Chagas' disease in mice. J. Parasit. 71(2):183-191.

Grogl, M., and R.E. Kuhn. 1984. Identification of antigens of Trypanosoma cruzi and

Trypanosoma rangeli recognized by sera from patients with chronic Chagas' disease. J. Parasit. 70(5):822-824.

Grogl, M., and R.E. Kuhn. 1984. Antibodies to Trypanosoma cruzi in coyotes in Texas. J. Parasit. 70(1):189-191.

Tarleton, R.L., C. Schulz, M. Grogl, and R.E. Kuhn. 1984. Diagnosis of Chagas' disease in humans using a biotin-3H-avidin radioimmunoassay. Amer. J. Trop. Med. and Hyg. 33(1):34-40.

Cunningham, D.S., M. Grogl, and R.E. Kuhn. 1980. Suppression of antibody responses in humans infected with Trypanosoma cruzi. Infection and Immunity. 30(2):496-499.

Grogl, M., C.J. Marinkelle, M.F. Suarez, F. Guhl, and N. de Sanchez. 1980. Trypanosoma magdalenae sp. n. (Protozoa: Kinetoplastida) from a freshwater Teleost Petenia kraussii in Colombia. J. Parasitol. 66(6):1022-1026.

Guhl, F., C.J. Marinkelle, N. de Sanchez, and M. Grogl. 1979. Studies on the transfer of temperature-induced humoral immunity to T. cruzi in mice. Rev. Inst. Med. Trop. Sao Paulo. 21(4):166-171.

Grogl, M., C.J. Marinkkelle, N. de Sanchez, and F. Guhl. 1979. Pigs as a potential source of Toxoplasmosis and Isosporosis in humans in Colombia. Antioquia medica. 28(1-2):14-16.

de Sanchez, N.R., C.J. Marinkelle, F. Guhl, and M. Grogl. 1979. Antibodies against T. cruzi in hospitalized patients in Bogota, Colombia. Rev. Lat. Am. de Microbiol. 21(1):27-29.

Marinkelle, C.J., N. de Sanchez, M. Grogl, and F. Guhl. 1978. Recommendations for optimizing The storage of sera by absorption in filter paper under field conditions, for the diagnosis of Chagas' disease using the indirect immunofluorescent technique. Rev. Inst. Med. Trop. Sao Paulo. 20(2):112-114.

von Prahl, H., M. Grogl, and F. Guhl. 1978. Carideos (Decapodos, Natantia, Palaemonidae) Capture on Gorgona Island.(Colombia Coast, Pacific) Cespedecia. Vol. VII, No.'s 25-26.

Guhl, F., C.J. Marinkelle, N. de Sanchez, and M. Grogl. Immunologic and serologic differences between experimental infection with Trypanosoma cruzi and T. rangeli in mice. Ant. Med. In Press.

Grogl, M., A.M.J. Oduola, L.D.C. Cordero, and D.E. Kyle. 1989. Leishmania spp: development of Pentostam-resistant clones in vitro by discontinuous drug exposure. Exp. Parasitology. 69:230-244.

Zatjtchuk, J.T., E.M. Netto, M. Grogl, R.C. Neafie, and P.D. Marsden. 1989. Mucosal leishmaniasis in Brazil. Laryngoscope. 99(9):925-939.

Armijos, R.X., M.E. Chico, R.H. Guderian, R.D. Kreutzer, J.D. Berman, M.D. Rogers,and M. Grogl. 1990. Human cutaneous leishmaniasis in Ecuador: Identification of parasites by enzyme electrophoresis. Amer. J. Trop. Med. & Hyg. 42(5):424-428.

Watt, G., G.W. Long, M. Grogl, and S.K. Martin. 1990. Reversal of multidrug resistance by calcium antagonists: potential for host toxicity. Trans. Royal. Soc. Trop. Med. Hyg. 84:187-190.

Bell, C.A., J.E. Hall, D.E. Kyle, M. Grogl, K.A. Ohemeng, and R.R. Tidwell. 1990. Structure-activity relationship of novel derivatives of pentamidine against Plasmodium falciparum and Leishmania mexicana amazonensis. Antimicrob. agents Chemother. 34(7):1381-1386.

Watt, G., G.W. Long, M. Grogl, and S.K. Martin. 1990. Reversal of drug-resistant falciparum malaria by calcium antagonists: potential for host cell toxicity. World Health Organization. WHO/MAL/90.1056, 1-10.

McHugh, C.P., M. Grogl, and S.F. Kerr. 1990. Isolation of Leishmania mexicana from Neotoma micropus collected in Texas. J. Parasit. 76(5): 741-743.

Klayman, D.L., A.J. Lin, J.W. McCall, M. Grogl, and K.E. Kinnamon. 1991. 2-Acetylpyridine thiosemicarbazones.13 Derivatives with antifilarial activity. J. Med. Chem. 34: 1422-1425.

Grogl, M., R. Alvarado, C.J. Marinkelle, F. Guhl, and N. de Sanchez. Preliminary studies on congenital toxoplasmosis conducted at two hospital centers in the city of Bogota. Submitted to Rev. Inst. Trop. Sao Paulo.

Grogl, M., R.K. Martin, A.M.J. Oduola, W.K. Milhous, and D.E. Kyle, 1991. Characteristics of multidrug resistance in Plasmodium and Leishmania: Detection of P-glycoprotein-like components. J. Amer. Soc. Trop. Med. & Hyg. 45(1): 98-111.

Guderian, R.H., M.E. Chico, M.D. Rogers, M. Grogl, and J.D. Berman. 1991. Placebo controlled treatment of Ecuadorian cutaneous leishmaniasis. Amer. J. Trop. Med. & Hyg. 45(1): 92-97.

Kreutzer, R.D., A. Corredor, G. Grimaldi, M. Grogl, E.D. Rowton, D.G. Young, A. Morales, D. McMahon-Pratt, H. Guzman, and R.B. Tesh. 1991. Characterization of Leishmania Colombiensis sp.n. (Kinetoplastida: Trypanosomatidae), a new parasite infection humans, animals and phlebotomine sand flies in Colombia and Panama. Amer. J. Trop. Med. & Hyg. 44(6): 662-675.

Grogl, M., R.D. Kreutzer, C.P. McHugh, and R.K. Martin. 1991. Characterization of a Leishmania isolate from the rodent-host Neotoma Micropus collected in Texas and a Comparison to human isolates. Amer. J. Trop. Med. & Hyg. 45(6): 714-722.

Grogl, M., Thomason T.N., and E.D. Franke. 1992. Drug-resistance in leishmaniasis: its implication in systemic chemotherapy. Amer. J. Trop. Med. & Hyg. 47(1):117-126.

Magill A.J., M. Grogl, R.A. Gasser, W. Sun, and C.N. Oster. 1992. Viscerotropic leishmaniasis In persons returning from Operation Desert Storm - 1990-1991. Morbidity and Mortality Weekly Report 41(8): 131-134.

Magill A.J., Grogl M., Gasser R.A., Wellington S., Oster C.N. 1993. Viscerotropic leishmaniasis caused by Leishmania tropica in soldiers returning from Operation Desert Storm. N. Engl. J. Med. 328(19):1383-1387.

Magill A.J., Grogl M., Gasser R.A., Oster C.N. 1993. Visceral leishmaniasis in Desert Storm veterans. N. Engl. J. Med. 329 20):1503-1504.2.

Magill A.J., Grogl M., Johnson S.C., Gasser R.A. 1994. Visceral infection caused by Leishmania tropica in a Desert Storm veteran presenting 2 years after last exposure. Clinical Infectious Diseases.

Chan M.M.Y., Grogl M., Chen C.C., Biene E.J., Fong D. 1993. Herbicides to curb human parasitic infections: In vitro and in vivo effects of trifluralin on the trypanosomatid protozoans. Proc. Natl. Acad. Sci. USA, 90:5657-5661.

Kreutzer R.D., Grogl M., Neva F.A., Magill A.J., Fryauff D.J. 1993. Identification and genetic comparison of Leishmania parasites causing viscerotropic and cutaneous disease in soldiers returning from operation Desert Storm .Am. J. Trop. Med. Hyg. 49:357-363.

McHugh C.P., and Grogl M. 1993. Isolation of Leishmania mexicana (Kinetoplastida: Trypanosomatidae) from Lutzomya anthophora (Diptera:Psychodidae) collected in Texas. Journal of Medical Entomolgy 30(3):631-633.

Grogl M., Chan M.M.Y., Rodriguez R., Gately L.J., Berman J.D., Fong D. 1993. Paromomycin resistance in Leishmania tropica: lack of correlation with mutation in the small subunit ribosomal RNA. Amer. J. Trop. Med. 51:758-766.

Soto J., Grogl M., Berman J.D. 1993. Evaluation of Pentamidine for the treatment of cutaneous leishmaniasis in Colombia. Clin. Inf. Dis. 16:417-425.

Grogl M., Daugirda J.L., Hoover D.L., Magill A.J., Berman J.D. 1993. Survivability and infectivity of viscerotropic Leishmania tropica from Desert Storm participants in human

blood products maintained under blood bank conditions. Am. J. Trop. Med. Hyg. 49:308-315.

Dietze R., Milan E.P., Berman J.D., Grogl M., G. Ksionski. 1993. Treatment of Brazilian Kala-Azar with a short course of amphocil (Amphotericin B cholesterol dispersion). Clinical Infectious Diseases 17:981-986.

Soto J., Buffet P., Grogl M., Berman J.D. 1994. Successful treatment of Colombian cutaneous leishmaniasis with four injections of Pentamidine. Am. J. Trop. Med. Hyg. 50(1):107-111.

Soto J., Grogl M., Berman J.D., Olliaro P. 1994. Limited efficacy of injectable aminosidine as single-agent tharapy for Colombia cutaneous leishmaniasis. Trans. Roy. Soc. Trop. Med. Hyg. 88:695-698.

Gasser R.A., Magill A.J., Oster C.N., Franke E., Grogl M., Berman J.D. 1994. Pentavalent antimonials induce pancreatitis in 98% of leishmaniasis patients. Clinical Infectious Disease 18:83-90.

Kreutzer R.D., Yemma J.J., Grogl M., Tesh R.B., Martin T.I. 1994. Evidence of sexual reproduction in the protozoan parasite Leishmania (Kinetoplastida: Trypanosomatidae). Am. J. trop. Med. Hyg. 51:301-307.

Chan M., Grogl M, Callahan H., Fong D. 1995. Efficacy of the herbicide Trifluralin against P glycoprotein-expressing strains of Leishmania.1995. Antimicrobial Agent and Chemotherapy 39 (7):1609 -1611.

Nuzum E., White F., Thakur C., Dietze R., Wages J., Grogl M, Berman J.D. 1995. Diagnosis of symptomatic visceral leishmaniasis by use of the polymerase chain reaction on patient blood. JID 171:751-754.

Soto J., Hernandez N., Grogl M., Berman J.D. 1995. Successful treatment of New World cutaneous leishmaniasis with a combination of topical paromomycin-MBCL and injectable meglumine antimonate. Clinical Infectious Diseases 20:47-51.

Chico M., Guderian R.H., Cooper P.J., Armijos R., Grogl, M. 1995. Evaluation of a direct immunofluorescent antibody (DIFMA) test using Leishmania genus-specific monoclonal antibody in the routine diagnosis of cutaneous leishmaniasis. Rev Soc. Bras. Med. Trop. 28(2):99-103.

Buffet P., Deray G., Grogl M., Jacobs C. 1995. Tolerance and pharmacokinetics of antimony in a patient with renal failure. Neophrology Dialysis and Transplantation 10(8):1477.

Callahan H.L., C. Kelley, T. Perreira, and M. Grogl. 1996. Microtubule inhibitors: structure activity analyses suggest rational models to identify potentially active compounds. Antimicrob. Agents Chemother 40 (4): 560-571.

Callahan, H.L., I.F. Portal, S.J. Bensinger, and M. Grogl. 1996. Leishmania spp: temperature sensitivity of promastigotes in vitro as a model for tropism in vivo. Exp. Parasit. 84: 400 409; Art (0128).

Callahan H.L., Portal A.C., and M. Grogl. 1997. An axenic amastigote system for drug screen. Antimicrobial Agents & Chemotherapy 41(4):818-822.

Velez I., Agudelo S., Hendrickx E., Puerta J., Grogl M., Modabber F., Berman J. 1997. Lack of efficacy of Allopurinol for Colombian cutaneous leishmaniasis:a randomized, controlled, double-blind clinical trial. Annals Internal Medicine 126:232-236.

Grogl M., Schuster B.G., Berman J.D., Ellis W.Y. 1999. Successful topical treatment of murine cutaneous leishmaniasis with a combination of Paromycin (Aminosidine) and Gentamicin. J. Parasitol. 85(2): 354-359.

Valli, L.C.P., V.M.A. Passos, R. Dietze, H.L. Callahan, J.D. Berman M. Grogl. 1999. Humoral immune responses among mucosal and cutaneous leishmaniasis patients caused by Leishmania braziliensis. Journal Parasitology 85:1200-1210.

Toledo, J., Gutierrez, P., Nicholls, S., Berman, J., Grogl, M. 2000. Treatment of cutaneous leishmaniasis with a topical antileishmanial drug (WR279396): Pilot study. Amer. J. Trop. Med. & Hyg. (Accepted).

Grogl M., Magill R.A., Johnson S.C. Oster C.N. Cutaneous leishmaniasis in U.S. Rangers and Marines associated with jungle warfare training in French Guina during 1992-1993. Am. J. trop. Med. Hyg. Manuscript in Preparation.

Callahan H.L., M.C. Wolfgang, and M. Grogl. Biochemical and karyotypic analysis of paromomycin resistant Leishmania tropica: rearrangements in ribosomal RNA-containing chromosomes. Mol. Biochem. Parasitol. Manuscript in preparation.

Grogl, M. and H.L. Callahan. An animal model (Mesocricetus auratus) of Leishmania tropism. J. Parasitol. Manuscript in Preparation.

Pappas, M.G., M. Grogl, J.W. Vincent, and R. Hajkowski. Characterization of a monoclonal antibody reactive with a surface antigen on promastigotes and amastigotes of L. amazonensis. Manuscript in preparation.

Kuhn, R.E., and M. Grogl. Natural antibodies in parasitic diseases. Manuscript in preparation.

Sanchez, J.L., I. Bendet, M. Grogl, J.B.P. Lima L.W. Pang et. al. Malaria in Brazilian military personnel deployed to Angola. (Submitted Amer. J .Trop. Med. Hyg.)

BOOKS:

DeNigris, E., Grogl, M., Cotelingam, J.D. 1996. Leishmaniasis. In "Pathology of Infectious Diseases" (D.H. Connor), Appleton & Lange, Philadelphia, PA.

Berman, J.D., and M. Grogl. 1989. Biochemical Mechanisms of Pentostam. In "Leishmaniasis the Current Status and New Strategies for control." (D.T. Hart, Ed.), Chapter 4, pp. 473-478. NATO ASI Series, Plenum Press, New York and London.

von Prahl, H., F. Guhl, and M. Grogl. 1979. An ecological study of Gorgona Island. Futura Grupo Editorial Ltds., Bogota; D.E.: 279 pp.

ABSTRACTS: ORAL PRESENTATIONS AND POSTER SESSIONS:

2001 World Leish II Congress, Hersonissos, Crete, 20 -24 May. Grogl, M. Invited Lecture "WRAIR's c-GMP-manufactured leishmanin skin test antigens"

1997 First World Congress of Leishmaniosis, Turkey. Grogl, M. WR279,396: A new formulation for the treatment of cutaneous leishmaniasis"

1996 12th Annaul Meeting on Basic Research in Chagas' Disease and 13th Meeeting of the Brazilian Society of Protozoology, Caxambu, MG, November 5-18. Grogl, M. Invited Lecture "New strategies for chemotherapy in leishmaniasis"

Memorias Instituto Oswaldo Cruz 91 suppl: 160. Valli, L.C., H.L. Callahan, V.M.A. Passos, R. Dietze, and M. Grogl. Humoral responses in mucocutaneous and cutaneous leishmaniasis caused by Leishmania braziliensis.

Memorias Instituto Oswaldo Cruz 91 suppl: 151. Pereira, T., H.L. Callahan, and M. Grogl. Development of animal models for the identification of the genetic factors which control tropism in Leishmania.

Memorias Instituto Oswaldo Cruz 91 suppl: 152. Portal, I., M. Grogl, and H.L. Callahan.

Development of temperature and tropism mutants in vitro and in vivo for use in the determination of the genetic basis of tropism in Leishmania.

Memorias Instituto Oswaldo Cruz 91 suppl: 202. Portal, A., M. Grogl, and H.L. Callahan. Another look at the mechanisms of action of pentavalent antimonials.

American Society of Tropical Medicine & Hygiene 55(2) suppl: 291. Buffet, P.A., H.L. Callahan, B. Larouze, M. Deniau, and M. Grogl. The development of a murine model for selecting prophylactic schedules to prevent relapse of visceral leishmaniasis in HIV patients.

American Society of Tropical Medicine & Hygiene 55(2) suppl: 189. Grogl, M., I.F. Portal, T.R. Pereira, R. Dietz, and H.L. Callahan. The development and testing of in vitro and in vivo models for use in the determination of the genetic basis of tropism in Leishmania.

American Society of Tropical Medicine & Hygiene 55(2) suppl: 293. Callahan, H.L., A.C. Portal, A.D. Matos, and M. Grogl. Another look at the mechanisms of action of pentavalent antimonials and of resistance in Leishmania.

UCLA Keystone Symposia. Callahan, H.L., M.C. Wolfgang, and M. Grogl. Paromomycin resistance in Leishmania.

1995 43th Annual Meeting of the American Society of Tropical Medicine and Hygiene. Callahan,H.L., I.F. Portal, and M. Grogl. Development of an in vitro Leishmania temperature sensitivity model: preliminary studies with strains with aberrant tropism in vivo. Am. Soc. Trop. Med. Hyg. 53(2) suppl:215.

43th Annual Meeting of the American Society of Tropical Medicine and Hygiene. Grogl, M., A.C. Portal, and H.L. Callahan. The drug sensitivity profile of free amastigotes: development of a new model system for screening drugs. Am. Soc. Trop. Med. Hyg. 53(2) suppl:186.

43th Annual Meeting of the American Society of Tropical Medicine and Hygiene. Kelley, C., H.L. Callahan, T.T.R. Pereira, and M. Grogl. Structure-function analysis: a new approach to trifluralin analogs yields results. Am. Soc. Trop. Med. Hyg. 53(2) suppl:185.

Brazilian Chagas Annual Meeting - Caxiambu. Portal, I.F., M. Grogl, and H.L. Callahan. The in vitro temperature sensitivity of Leishmania promastigotes correlates with the in vitro temperature sensitivity of amastigotes, and the temperature tropism of Leishmania in vivo. Mem. Inst. Oswaldo Cruz 90 suppl:101.

Brazilian Chagas Annual Meeting - Caxiambu. Portal, A., M. Grogl, and H.L. Callahan. The free amastigote drug screen is a better system for screening anti-leishmanials. Mem. Inst. Oswaldo Cruz 90 suppl:212.

Brazilian Chagas Annual Meeting - Caxiambu. Periera T.R., H.L. Callahan, C. Kelley, and M. Grogl. Identification of a new lead drug from trifluralin and identification of its putative mechanism of action from structure-function analysis. Mem. Inst. Oswaldo Cruz 90 suppl:213.

1994 42nd Annual Meeting of the American Society of Tropical Medicine and Hygiene. Wolfgang, M.C., Callahan, H.L., and M. Grogl. Mechanism of paromomycin resistance in Leishmania. Am. Soc. Trop. Med. Hyg. 51(3) suppl:273.

Brazilian Chagas Annual Meeting - Caxiambu. Grogl, M., J.D. Berman, and H.L. Callahan. A new topical formulation active against new world cutaneous leishmaniasis. Mem. Inst. Oswaldo Cruz 89 suppl:193.

Brazilian Chagas Annual Meeting - Caxiambu. Bensinger, S.J., Grogl, M., and H.L. Callahan. Comparison of Leishmania tropism with temperature sensitivity. Mem. Inst. Oswaldo Cruz 89 suppl:107.

Brazilian Chagas Annual Meeting - Caxiambu. Wolfgang, M.C., M. Grogl, and H.L. Callahan. A novel paromomycin resistance mechanism in Leishmania tropica. Mem. Inst. Oswaldo Cruz 89 suppl: 94.

1993 41st Annual Meeting of the American Society of Tropical Medicine and Hygiene. Grogl M. Survivability and infectivity of viscerotropic Leishmania tropica from Desert Storm participants in human blood maintained under blood bank conditions. Vol. 47 (4):184.

41st Annual Meeting of the American Society of Tropical Medicine and Hygiene. Gasser R.A., Magill A.J., Oster C.N., Franke E., Grogl M., Berman J.D. Pentavalent antimonials induce pancreatitis in patients with leishmaniasis. Vol. 47 (4):209.

41st Annual Meeting of the American Society of Tropical Medicine and Hygiene. Kreutzer R.D. and Grogl M. Identification of leishmania parasites from Desert Storm participants and a genetic comparison with other Olf World isolates. Vol. 47 (4):24

1992 626th Meeting The Helminthological Society of Washington. M. Grogl Diagnosis of patients with leishmaniasis and current developments.

Society of Air Force Physicians. A.J. Magill, M. Grogl, R. Gasser, and C.N. Oster. Viscerotropic leishmaniasis in soldiers returning from operation Desert Storm.

Electronic Networking For Interamerican Microbiological Collaborative Research Workshop, Campinas, Brasil, February 17-21, (PAHO & MSDN). M. Grogl. Overview: Leishmania Identification and characterization.

1991 40th Annual Meeting of the American Society of Tropical Medicine and Hygiene. M. Grogl, J. Mendez, W.K. Milhous, E.O. Nuzum, R.K. Martin, J.D. Berman, B.G. Schuster, and C.N. Oster. Leishmaniasis in Desert Shield/Storm.

40th Annual Meeting of the American Society of Tropical Medicine and Hygiene. E.O. Nuzum, J.M. Wempe, and M. Grogl. Canine leishmaniasis in a U.S. military working dog coming back from Desert Shield.

40th Annual Meeting of the American Society of Tropical Medicine and Hygiene. R.D. Kreutzer, R.B. Tesh, M. Grogl, F.A. Neva, and J.J. Yema. Are there naturally occurring leishmania hybrids?

1990 Proc. 39th Annual Meeting of the American Society of Tropical Medicine and Hygiene. P.D. Van Zandt, R.D. Kreutzer, M. Grogl, R.K. Martin and C.P. McHugh. Biochemical Characterization of Leishmania mexicana, Isolated in Texas from the Wood Rat, Neotoma micropus. Abstract 385, November 4-5.

Proc. 39th Annual Meeting of the American Society of Tropical Medicine and Hygiene. G. Watt, G.W. Long, M. Grogl, and S.K. Martin. Reversal of Drug-Resistant Falciparum Malaria by Calcium Antagonists: Potential for Host Cell Toxicity. Abstract 350, November 4-5.

Proc. 39th Annual Meeting of the American Society of Tropical Medicine and Hygiene. D.K. Klayman, A.J. Lin, J.W. McCall, S.Y. Wang, S. Townson, M. Grogl, and K.E. Kinnamon. Filarizone and Related Thiosemicarbazones as New Antifilarial Agents. Abstract 179, November 4-5.

Proc. 39th Annual Meeting of the American Society of Tropical Medicine and Hygiene. C.A. Bell, M. Cory, J.E. Hall, D.E. Kyle, M. Grogl, and R.R. Tidwell. Correlations Between the Antiprotozoal Activity of Analogues of Pentamidine and Their Affinity for DNA. Abstract 153, November 4-5.

Proc. 39th Annual Meeting of the American Society of Tropical Medicine and Hygiene. M. Grogl, L.L. Fleckenstein, R.K. Martin, D.E. Kyle, W.Y. Ellis, W.A. Reid, J.D. Berman, and B.G. Schuster. Topical Formulations for the Treatment of Cutaneous Leishmaniasis: Current Status. Abstract 110, November 4-5.

15th Annual Meeting of the Society of Armed Forces Medical Laboratory Scientists, Baltimore, MD. W.K. Milhous, M. Grogl, R.K. Martin, and D.E. Kyle. Anti-efflux antimicrobials. Proc. Soc. Armed Forces Med. Lab. Sci. 18:11.

15th Annual Meeting of the Society of Armed Forces Medical Laboratory Scientists, Baltimore, MD. W.K. Milhous, L. Gerena, G.T. Bass, C.L. Pryor, N.A. Edwards, R.K.

Martin, M. Grogl, and D.E. Kyle. A new look at some old antifolate antimalarials. Proc. Soc. Armed Forces Med. Lab. Sci. 18:27.

15th Annual Meeting of the Society of Armed Forces Medical Laboratory Scientists, Baltimore, MD. M. Grogl, T. Thomason, L.C. Cordero, W.K. Milhous, R.K. Martin, and D.E. Kyle. An in vitro semiautomated micromethod for drug sensitivity testing of Leishmania: clinical relevance. Proc. Soc. Armed Forces Med. Lab. Sci. 18:27.

1989 UCLA Symposium on Molecular and Cellular Biology (Parasites: Molecular Biology, Drug and Vaccine Design). R.R. Tidwell, C.A. Bell, S.K. Jones, J.E. hall, V. Linga, M.A. Allen, D.E. Kyle, and M. Grogl. Structure-activity studies on the effects of novel pentamidine derivatives against parasitic microorganisms. J. Cell. Biochem. 13E:150.

Conference on Multidrug Resistance; Molecular Biology, Washington, DC. M. Grogl,D.E. Kyle, A.M.J. Oduola, S.K. Martin, R.K. Martin, and W.K. Milhous. Detection Plasmodium and Leishmania components by C219; a monoclonal antibody specific for a conserved cytoplasmic domain of P-glycoprotein. Session I Genetics and Biology of the Multidrug Transporter (P-glycoprotein), Abstract 27.

38th Annual Meeting of the American Society of Tropical Medicine and Hygiene, Hawaii. M. Grogl, T. Thomason, D. Panisko, J.S. Keystone, and D.E. Kyle. Clinical relevance of in vitro antileishmanial susceptibility testing, Abstract 105,December 10-14.

38th Annual Meeting of the American Society of Tropical Medicine and Hygiene, Hawaii. R.K. Martin, M. Grogl, N.A. Edwards, and D.E. Kyle. Comparative analysis of differing sensitivities to pentostam of two species of Leishmania by pulse-field gel electrophoresis, Abstract 106, December 10-14.

38th Annual Meeting of the American Society of Tropical Medicine and Hygiene, Hawaii. R.D. Kreutzer, M. Grogl, D.G. Young, E.D. Rowton, R.B. Tesh, G. Grimaldi, and A. Corredor. Biochemically similar Leishmania like parasites from Colombia and Panama, Abstract 93, December 10-14.

14th Annual Meeting of the Society of Armed Forces Medical Laboratory Scientists, San Antonio, TX. M. Grogl, W.K. Milhous, R.K. Martin, and D.E. Kyle. Kits for the diagnosis of cutaneous leishmaniasis in field laboratories. Proc. Soc. Armed Forces Med. Lab. Sci. 18:22.

14th Annual Meeting of the Society of Armed Forces Medical Laboratory Scientists, San Antonio, Tx. W. K. Milhous, L. Gerena, T. Julvar, A. Bass, M. Grogl, R.K. Martin, and D.E. Kyle. Clinical relevance of in vitro antimalarial susceptibility testing. Proc. Soc. Armed Forces Med. Lab. Sci. 18:23.

1988 4th International Conference in Aids, Stockholm Sweden. D. Gluckstein, J. Ruskin, F.

Ciferri, and M. Grogl. Chagas' disease: a newly recognized cause of cerebral mass lesion in AIDS. 37th Annual Meeting of the American Society of Tropical Medicine and Hygiene.

M.Grogl, P.W. Kelley, W.R, Ballou, J.D. Berman, D. Gordon, E. Rowton, and R.D. Kreutzer. Cutaneous leishmaniasis in the Guatemalan Army, Abstract 284, December 4-8.

37th Annual Meeting of the American Society of Tropical Medicine and Hygiene .J.D.Berman, M. King, N. Edwards, and M. Grogl. Biochemistry of Pentostam-Resistant Leishmania, Abstract 154, December 4-8.

37th Annual Meeting of the American Society of Tropical Medicine and Hygiene. S.J. Wu, E.D. Rowton, P. V. Perkins, M. Grogl, and R.G. Andre. Characterization of antigen epitopes of infective stage of Leishmania major promastigotes by using monoclonal antibodies in Western Blot, Abstract 47, December 4-8.

12th International Congress for Tropical Medicine and Malaria. The Netherlands. M. Grogl, D.E. Kyle, W.K. Milhous, and A.M.J. Oduola. Characteristics of drug resistance in Leishmania, Abstract THP-5-10, September 18-23.

12 International Congress for Tropical Medicine and Malaria. D.E. Kyle, A.M.J. Oduola, M. Grogl, and W.K. Milhous. The Netherlands. Characterization of compounds that modulate resistance of Plasmodium falciparum to chloroquine in vitro, Abstract (Grogl,FRP-3-13, September 18-23.

1987 The 62nd Annual Meeting of the American Society of Parasitologists, Lincoln, Nebraska. M. Grogl and D.E. Kyle. Development of Pentostam-resistant Leishmania clones in vitro. Abstract No. 89, August 5.

The 36th Annual Meeting of the American Society of Tropical Medicine and Hygiene, Los Angeles, CA. M. Grogl, A.M.J. Oduola, D.E. Kyle, J.D. Berman, and W.K. Milhous. Accumulation of Pentostam in stable drug resistant clones of Leishmania. Abstract No.182, Dec. 1.

The 36th Annual Meeting of the American Society of Tropical Medicine and Hygiene, Los Angeles, CA. D.E. Kyle, M. Grogl, S.K. Martin, W.K. Milhous, and A.M.J. Oduola. Occurrence of P-glycoprotein on Plasmodium and Leishmania: A marker for drug resistance? Abstract No. 154, December 3.

Second Latin American Congress of Tropical Medicine. Bogota, Colombia. M. Grogl. Advances in treatment and management of patients with American cutaneous leishmaniasis. Proceedings 2(1) 45, May 25-29.

Invited paper: Proceedings of the 8th Latin American Congress of Parasitology & Tropical Medicine. Guatemala City, Guatemala. M. Grogl and M.I. Torres. Molecular Biology and Diagnosis of Leishmaniasis. November 19.

1986 Invited participant to the International Workshop on Leishmania Chemotherapy. World Health Organization, Leishmaniases Scientific Working Group, CHEM-LEISH Steering Committee. P.B. McGreevy and M. Grogl. A new formulation for the topical treatment of cutaneous leishmaniasis. May 27-28, Washington, DC.

578th Meeting of the Helminthological Society of Washington. M.Grogl. Kinetoplast DNA. March 19.

The 35th Annual Meeting of the American Society of Tropical Medicine and Hygiene. Scientific Session T, No. 153. M. Grogl, P.J. Sayles and P.B. McGreevy. Humoral immune reactions in patients with cutaneous and mucosal lesions, Abstract No. 56.

1985 VII International Congress of Protozoology, Nairobi, Kenya. M. Grogl and D.F. Wirth. DNA characterization as a taxonomic tool for identification of Kinetoplastic Flagellate Protozoans. Session P(CP13), June 28.

International Congress for Infectious Diseases, Cairo, Egypt. International Federation for Infectious and Parasitic Diseases in Collaboration with the World Health Organization. M. Grogl. Invited paper: The use of DNA probes in the identification an characterization of Leishmania spp. Section 24. April 23.

The 34th Annual Meeting of the American Society of Tropical Medicine and Hygiene. Scientific Session 5: Kinetoplastids II; Biochemistry: Immunology and Molecular Biology. M. Grogl, P.D. Marsden and D.F. Wirth. Schizodeme characterization of Leishmania braziliensis braziliensis isolated from mucosal and cutaneous lesions from Tres Bracos, Brazil. Abstract No. 328, November 7.

The 34th Annual Meeting of the American Society of Tropical Medicine and Hygiene. Scientific Session F: Kinetoplastids I: Biochemical-Pharmacology. P.B. McGreevy, J. Andujar, and M. Grogl. Topical chemotherapy for cutaneous ulcers caused by Leishmania braziliensis panamensis in Mystromys albicaudatus. Abstract No. 131, November 5.

1984 75th Annual Meeting, Federation of American Societies of Experimental Biology. M. Grogl and R.E. Kuhn. Natural antibodies to protozoan and metazoan parasites. Federation Proceedings 43(6):1421, Paper No. 30.

General Meeting, Marine Biological Laboratory, Woods Hole, Massachusetts. M. Grogl and D. Wirth. DNA characterization as a taxonomic tool for identification of Leishmania species. Session 6. Parasitology and Pathology, August 21.

NSF US-Latin American Cooperative Science Program: Conference on Immunoparasitology, Medellin, Colombia. M. Grogl and G.H. MacDonald. Antigen-antibody analysis of the larval stage of Taenia solium. July 21.

67th Annual Meeting, Federation of American Societies for Experimental Biology. M. Grogl and R.E. Kuhn. Parasite-host cross reacting antibodies during chronic Chagas' disease: An immunochemical approach. Federation Proceedings 42(4):964, Paper No.3939.

1982 66th Annual Meeting, Federation of American Societies for Experimental Biology. F. Hatcher, M. Grogl, W. Woodruff and R. Kuhn. Poly I: C-induced change in the resistance phenotype of C67BL/6 mice infected with Trypanosoma cruzi. Federation Proceedings 42(3):578.

1978-87 Annual Fancy Gap Immunoparasitology Workshops (V, VI, VII, VIII, IX, X,XI).

TITLE: TOPICAL TREATMENT OF CUTANEOUS LEISHMANIASIS WITH WR 279396: A PHASE 2 STUDY IN THE OLD WORLD

Appendix 3
ADVERSE EVENT NOTIFICATION FORM

Version 7.0
IND 50,098
Log No.
17 June 2002

Topical Treatment of Cutaneous Leishmaniasis with [illegible] 279,396: A Phase 2 Study in the Old World
Adverse Event Notification Flow Chart

Serious and/or Unexpected*

Investigator notifies Medical Monitor and Principal Investigator (PI) Immediately

Nature: Death or Life Threatening and Associated with WR279396

- Action: PI notifies Product Manager and DOCSRCQ as soon as possible
  - Vehicle: Phone or Fax Safety Report Form
    DOCSRCQ:
    Ph: 301-619-2165/6245
    Fax: 301-619-7803
    Medical Monitor:
    PH: 301-594-7105
    or 301-230-0426
    Product Manager.
    Ph: 301-619-2053
    Fax: 301-619-2304
  - Action: DOCSRCQ notifies FDA telephonically within 3 days
  - Vehicle: Phone call followed by IND Safety Report within 10 days
- Action: PI notifies IND Director and WRAIR Research Management within 24 hours
  - Vehicle: IND Director
    Ph: 301-295-7871
    Fax: 301-295-7755
    WRAIR Fax
    301-319-9961
  - Action: PI notifies Local Ethical Committees within 72 hours

ALSO ALWAYS CALL THE PI 301-319-9359/410-884-7060

Nature: Associated with WR 279396

- Action: PI notifies Product Manager and DOCSRCQ
  - Vehicle: Phone or Fax Safety Report Form
    DOCSRCQ:
    Ph: 301-619-2165/6245
    Fax: 301-619-7803
    Medical Monitor:
    PH: 301-594-7105
    or 301-230-0426
    Product Manager.
    Ph: 301-619-2053
    Fax: 301-619-2304
  - Action: DOCSRCQ notifies FDA within 10 days
  - Vehicle: Report by IND Safety Report
- Action: PI notifies IND Director and WRAIR Research Management within 24 hours
  - Vehicle: IND Director
    Ph: 301-295-7871
    Fax: 301-295-7755
    WRAIR Fax
    301-319-9961
  - Action: PI notifies Local Ethical Committees within 72 hours

Nature: Not Associated with WR 279396

- Action: PI notifies Product Manager, IND Director and WRAIR Research Management Immediately
  - Vehicle: Phone or Fax Description of Problem (Safety Report)
    DOCSRCQ :
    Ph: 301-619-2165/6295
    Fax: 301-619-7803
    Medical Monitor:
    PH: 301-594-7105
    or 301-230-0426
    Product Manager.
    Ph: 301-619-2053
    Fax: 301-619-2304
    IND Drector:
    Ph: 301-295-7871
    Fax: 301-295-7755
    WRAIR Research Management
    Fax: 301-319-9961
  - Tabulate
  - Annual Report

Expected or Non-Serious Events Associated with WR 279396

- Action: Tabulate Only
- Vehicle: Annual Report

MEDWATCH
THE FDA MEDICAL PRODUCTS REPORTING PROGRAM

For VOLUNTARY reporting by health professionals of adverse events and product problems

Page ____ of ____

Form Approved: OMB No. 0910-0291 Expires: 04/30/03
See OMB statement on reverse

FDA Use Only

Triage unit sequence #

A. Patient information

1. Patient identifier
In confidence

2. Age at time of event: ______ or Date of birth:

3. Sex
☐ female
☐ male

4. Weight
______ lbs
or
______ kgs

B. Adverse event or product problem

1. ☐ Adverse event and/or ☐ Product problem (e.g., defects/malfunctions)

2. Outcomes attributed to adverse event (check all that apply)
☐ death ______ (mo/day/yr)
☐ life-threatening
☐ hospitalization – initial or prolonged
☐ disability
☐ congenital anomaly
☐ required intervention to prevent permanent impairment/damage
☐ other: ______

3. Date of event (mo/day/yr)

4. Date of this report (mo/day/yr)

5. Describe event or problem

6. Relevant tests/laboratory data, including dates

7. Other relevant history, including preexisting medical conditions (e.g., allergies, race, pregnancy, smoking and alcohol use, hepatic/renal dysfunction, etc.)

C. Suspect medication(s)

1. Name (give labeled strength & mfr/labeler, if known)
#1
#2

2. Dose, frequency & route used
#1
#2

3. Therapy dates (if unknown, give duration) from/to (or best estimate)
#1
#2

4. Diagnosis for use (indication)
#1
#2

5. Event abated after use stopped or dose reduced
#1 ☐ yes ☐ no ☐ doesn't apply
#2 ☐ yes ☐ no ☐ doesn't apply

6. Lot # (if known)
#1
#2

7. Exp. date (if known)
#1
#2

8. Event reappeared after reintroduction
#1 ☐ yes ☐ no ☐ doesn't apply
#2 ☐ yes ☐ no ☐ doesn't apply

9. NDC # (for product problems only)
– –

10. Concomitant medical products and therapy dates (exclude treatment of event)

D. Suspect medical device

1. Brand name

2. Type of device

3. Manufacturer name & address

4. Operator of device
☐ health professional
☐ lay user/patient
☐ other: ______

5. Expiration date (mo/day/yr)

6.
model # ______
catalog # ______
serial # ______
lot # ______
other #

7. If implanted, give date (mo/day/yr)

8. If explanted, give date (mo/day/yr)

9. Device available for evaluation? (Do not send to FDA)
☐ yes ☐ no ☐ returned to manufacturer on ______ (mo/day/yr)

10. Concomitant medical products and therapy dates (exclude treatment of event)

E. Reporter (see confidentiality section on back)

1. Name & address
phone #

2. Health professional?
☐ yes ☐ no

3. Occupation

4. Also reported to
☐ manufacturer
☐ user facility
☐ distributor

5. If you do NOT want your identity disclosed to the manufacturer, place an " X " in this box. ☐

PLEASE TYPE OR USE BLACK INK

FDA

Mail to: MEDWATCH
5600 Fishers Lane
Rockville, MD 20852-9787

or FAX to:
1-800-FDA-0178

FDA Form 3500

Submission of a report does not constitute an admission that medical personnel or the product caused or contributed to the event.

129

TITLE: TOPICAL TREATMENT OF CUTANEOUS LEISHMANIASIS WITH WR 279396: A PHASE 2 STUDY IN THE OLD WORLD

Appendix 4
CASE REPORT FORM

Version 7.0
IND 50,098
Log No.
17 June 2002

VOLUNTEER IDENTIFICATION NUMBER _______

INSTRUCTIONS FOR COMPLETING THE CASE RECORD FORM

* Always use a **ball-point pen**

* Use only **black pen**

* To avoid information transferring through to other pages always place this divider in front of the next white page

* Do not leave any sections blank. The following abbreviations should be used to explain missing values:

"NA" - not applicable
"ND" - not done
"NK" - not known

Please not that "0" should be only be used to represent a numerical value

* All dates should be entered as **DD/MM/YY**

* If only part of a date is known, please enter "NK" for unknown information (I.e. NK/NK/84).

*** If an incorrect entry is made, please cross out the error with a SINGLE line. The correct entry should be written beside the original entry. Please initial and date all corrections.**

- When entering times, please use the **24 hour clock**

Note: Confidentiality must be maintained for all study subjects. The study number will be used to assure subject confidentiality.

Clarification: **Study No.: Number given to the volunteer at enrollment**
Lesion No.: Location of the lesion(s) in the "Lesion Clinical Finding Form"

THANK YOU FOR YOUR CO-OPERATION

Day -1

WR279396	Subject's Initials	Subject's Study Number
Log No.		

CONSENT FORM

Date written informed consent obtained: [ ][ ][ ][ ][ ][ ] (D D M M Y Y)

Today Date: [ ][ ][ ][ ][ ][ ] (D D M M Y Y)

Time [ ][ ] : [ ][ ] (H H : M M)

DEMOGRAPHIC DETAILS

Date of Birth [ ][ ][ ][ ][ ][ ] (D D M M Y Y)

Sex [ ] (M or F)

Volunteer's E-mail Address: ____________________

Home Address: ____________________

____

Office Address: ____________________

Volunteer's Phone Number: (Work) ____________ (Home) ____________

Name of Supervisor/Relative: ____________________
(Please Print Name) Last Name, First, MI

Supervisor/ Relative Phone Number: ____________

Address where volunteer can always be reached (e.g. Parents Address): ____________

____

Occupation/ Specialty: ____________________

WR279396	Subject's Initials	Subject's Study Number
Log No.		

MEDICAL HISTORY

A Past illnesses and Approximate Dates. If yes is checked, add approximate date(s).

	No	Yes	Approximate Date(s)
Frequent colds			
Skin problems (skin allergies)			
Sore throat			
Ear infections			
Bronchitis			
Asthma			
Allergy			
Injury			
Upset stomach			
Kidney trouble			
Liver trouble			
Heart trouble			
Rheumatic fever			
Convulsions			
TB			
Malaria			
Fungal diseases			
Diabetes			
Chagas disease			
Head injury			
Other illness (specify)			

B Childhood diseases

	No	Yes
Measles		
Mumps		
Chicken Pox (Varicella)		
Polio		
Whooping cough		
Scarlet fever		

Immunizations (give date of last booster)

	No	Yes	M	M	Y	Y
DPT						
Polio						
Measles						
TB						
Mumps						
Rubella						

Day -1

WR279396	Subject's Initials	Subject's Study Number
Log No.		

MEDICAL HISTORY (Continued)

C a Does the volunteer have a previous history of leishmaniasis? If Yes, provide comments: Yes ☐ No ☐

b Is the volunteer suffering from any other concomitant illness? If Yes, provide comments: Yes ☐ No ☐

c Is the volunteer currently taking any other medication? If yes, please complete **Concomitant Medication form** and provide brief provide comments: Yes ☐ No ☐

e Does the volunteer have allergies to any drug including aminoglycosides? If Yes, provide comments: Yes ☐ No ☐

Notes

a Do you have a specific medical problem to discuss with the medical officer? Yes ☐ No ☐

b Comments (abnormal conditions, recommended followup, medications, etc:)

Signature (Primary Medical Officer) : ______________________

Print Name (Last, First, MI): ______________________ Visit Date ☐☐☐☐☐☐

Day -1

WR279396	Subject's Initials	Subject's Study Number
Log No.		

HISTORY OF LEISHMANISIS

A Previous Leishmaniasis History

a Previous leishmaniasis lesion(s) Yes ☐ No ☐ If **No**, then go to **B**

b Antileishmanial drug used within the last 6 months Yes ☐ No ☐ If **No**, then go to **B**

c If **Yes** above, what is the, name of the drug used: ____________

d Regimen used: ____________ (mg/kg/day) Total days of treatment ☐☐

B History of Present Problem

a Date of exposure (days in endemic area): From: ☐☐☐☐☐☐ (D D M M Y Y) To: ☐☐☐☐☐☐ (D D M M Y Y)

b Date you first noticed the lesion: ☐☐☐☐☐☐ (D D M M Y Y)

c Initial characteristics of the lesion: ____________

____

d Date medical care was first sought for lesion: ☐☐☐☐☐☐ (D D M M Y Y)

e Specific geographic area: ____________

f Number of days in the area: ☐☐☐

g Was prophylaxis used against sand flies during exposure period? Yes ☐ No ☐

If g above is yes, did you use repellent ? Yes ☐ No ☐ If yes, what type ? ____________

____

Did you keep your sleeves rolled down ? Yes ☐ No ☐

Other prophylatic measures used ? Yes ☐ No ☐

If yes, explain what type was used : ____________

____

Day -1

WR279396	Subject's Initials	Subject's Study Number
Log No.		

PHYSICAL EXAMINATION

Vital Signs:

Height [ ] mts [ ][ ] cm Weight [ ][ ][ ] , [ ] kg

Pulse [ ][ ][ ] /min Temperature [ ][ ] , [ ] C

Blood Pressure Systolic [ ][ ][ ] mmHg Diastolic [ ][ ][ ] mmHg

Symptoms:

	No	Yes	Comments
Fever (elevation)			
Nausea			
Vomiting			
Dizziness			
Headache			
Diarrhea			
Weight Loss			
Other (explain)			

	Normal	Abnormal	Please tick as appropriate. If abnormal, please comment.
Eyes			
Ears			
Nose			
Thyroid			
Heart			
Lungs			
Spleen			
Kidney			
Hepatobiliary			
Gastrointestinal			
Musculus skeletal			
Skin (not lesions)			
Blood and lymphatic			
Endocrine and Metabolic			
Neurological			
Non-site specific (including allergies)			

	Yes	No
Are there any abnormalities ?		
If Yes, are there any abnormalities that are clinically significant ?		

If Yes, please comment: ______

WR279396	Subject's Initials	Subject's Study Number
Log No.		

PHYSICAL EXAMINATION (Continued)

DERMATOLOGY EXAMINATION

	Normal	Abnormal	Please tick as appropriate. If abnormal, please comment
Mucosal Tissue (Nose)	☐	☐	
Mucosal Tissue (Throat)	☐	☐	
Palate/Mouth	☐	☐	

HEARING TEST AND ROMBERG TEST

	Normal	Abnormal
Hearing Test - High Tone (250 Hz -3,000 Hz)	☐	☐

	250/256	500/512	1,000/1,024	2,000/2,048	3,000/2,896
Right					
Left					

	Normal	Abnormal
Hearing Test - Low Tone (4,000 Hz - 8,000 Hz)	☐	☐

	4,000/4,096	6,000/6,144	8,000/8,192
Right			
Left			

	Normal	Abnormal
Romberg Test	☐	☐

Comments: ______________________

Signature (Individual Conducting Hearing Test): ______________________

Print Name (Last, First, MI) : ______________________

Signature (Medical Officer/ Investigator): ______________________

Print Name (Last, First, MI): ______________________

D D M M Y Y

Day -1

WR279396	Subject's Initials	Subject's Study Number
Log No.		

LESION(s) - CLINICAL FINDINGS REPORT FORM

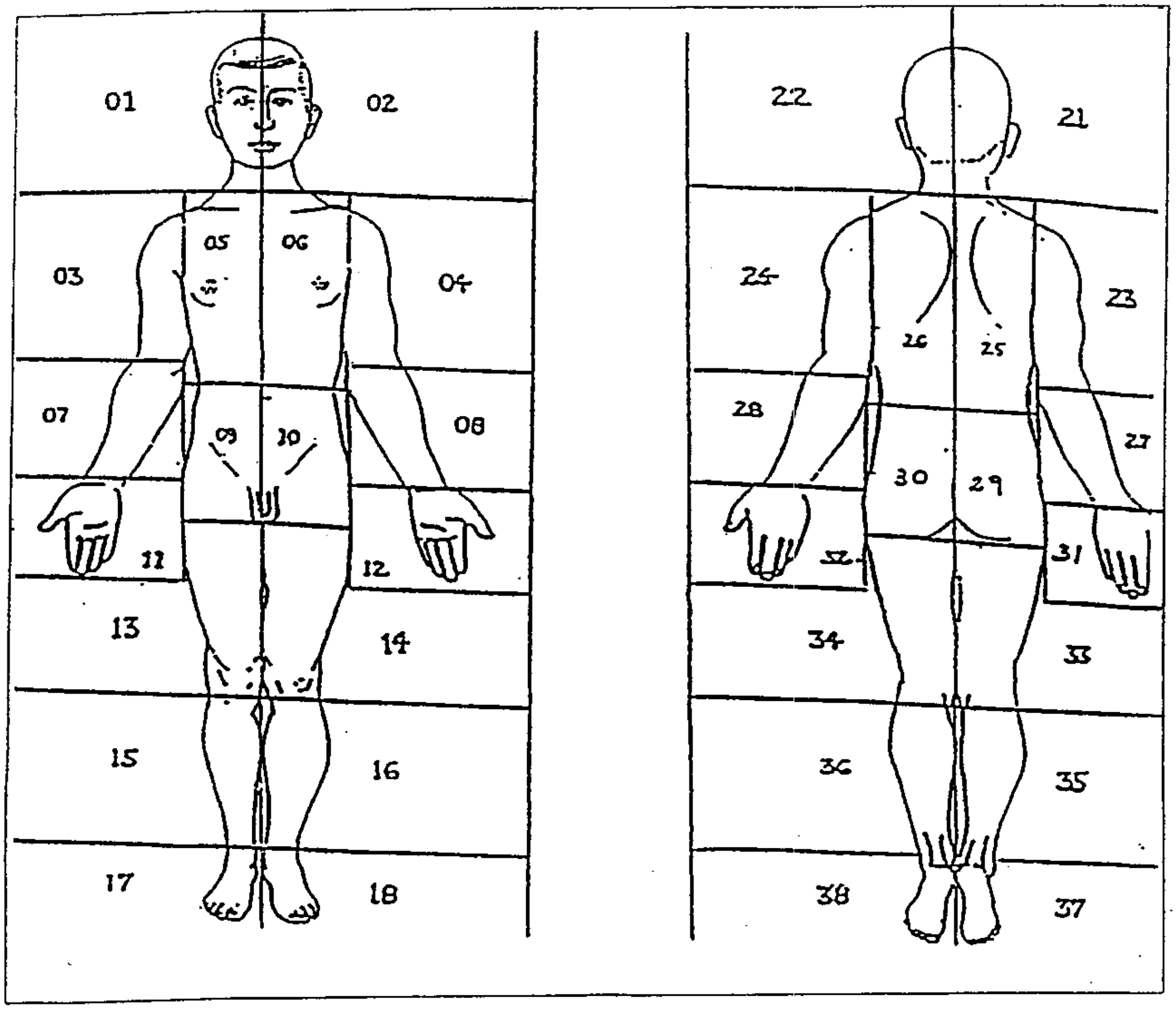

Location of lesion(s): Mark with an **X** the lesion(s) location & number the lesion on the fig. above (e.g. X1)

Lesion Number 1	Site according to the figure	
Designate type & size of lesion:		
Ulceration	________ X ________ mm.	
Induration	________ X ________ mm.	
Papule	________ X ________ mm.	
Papulo-Squamos (scaly) Lesion	________ X ________ mm.	
Lymph node ________ mm.		

WR279396	Subject's Initials	Subject's Study Number
Log No.		

LESION(s) - CLINICAL FINDINGS REPORT FORM (Continued)

Lesion Number 2	Site according to the figure ______
Designate type & size of lesion:	
Ulceration	______ X ______ mm.
Induration	______ X ______ mm.
Papule	______ X ______ mm.
Papulo-Squamos (scaly) Lesion	______ X ______ mm.
Lymph node ______ mm.	

Lesion Number 3	Site according to the figure ______
Designate type & size of lesion:	
Ulceration	______ X ______ mm.
Induration	______ X ______ mm.
Papule	______ X ______ mm.
Papulo-Squamos (scaly) Lesion	______ X ______ mm.

Lesion Number 4	Site according to the figure ______
Designate type & size of lesion:	
Ulceration	______ X ______ mm.
Induration	______ X ______ mm.
Papule	______ X ______ mm.
Papulo-Squamos (scaly) Lesion	______ X ______ mm.
Lymph node ______ mm.	

Lesion Number 5	Site according to the figure ______
Designate type & size of lesion:	
Ulceration	______ X ______ mm.
Induration	______ X ______ mm.
Papule	______ X ______ mm.
Papulo-Squamos (scaly) Lesion	______ X ______ mm.
Lymph node ______ mm.	

WR279396	Subject's Initials	Subject's Study Number
Log No.		

PARASITOLOGY LABORATORY
PATIENT DATA REPORT

Specimen(s) obtained: Yes: ______ No: ______

Date ☐☐☐☐☐☐ (D D M M Y Y) Time ☐☐ : ☐☐ (H H : M M)

Specimen(s) obtained by: ______________________________
Last name, First Name MI

Specimen(s) logged into (lab): Time ☐☐ : ☐☐ (H H M M) Lab ID Number: ______________

Source of specimen: (mark one only)

Lesion: ☐ Lymph Node: ☐ Other: ☐

Lesion Number: ______________ Lesion Site from Figure: ______________

Parasitology Laboratory Assessment:

Culture:

		Date Positive	Species Identified	Initials
SCH 1	D D M M Y Y	D D M M Y Y	______	______
SCH 2	D D M M Y Y	D D M M Y Y	______	______
SCH 3	D D M M Y Y	D D M M Y Y	______	______

	Neg	1+	2+	3+	4+	5+	Initials	
DQ smear	☐	☐	☐	☐	☐	☐	______	D D M M Y Y

Comments: ______________________________

____

____

Signature (Investigator/Subinvestigator): ______________ Date: ☐☐☐☐☐☐ (D D M M Y Y)

Note: If the species is *L. aethiopica* the volunteer will be referred to his primary health care physician as per protocol. Physician Signature: ______________________

WR279396	Subject's Initials	Subject's Study Number
Log No.		

PARASITOLOGY LABORATORY
PATIENT DATA REPORT

Specimen(s) obtained: Yes: ______ No: ______

Date |_|_|_|_|_|_| (D D M M Y Y) Time |_|_| : |_|_| (H H : M M)

Specimen(s) obtained by: ______________________________
Last name, First Name MI

Specimen(s) logged into (lab): Time |_|_| : |_|_| (H H M M) Lab ID Number: ______________

Source of specimen: (mark one only)

Lesion: [ ] Lymph Node: [ ] Other: [ ]

Lesion Number: ______________ Lesion Site from Figure: ______________

Parasitology Laboratory Assessment:

Culture:

		Date Positive	Species Identified	Initials
SCH 1	D D M M Y Y	D D M M Y Y	______	______
SCH 2	D D M M Y Y	D D M M Y Y	______	______
SCH 3	D D M M Y Y	D D M M Y Y	______	______

	Neg	1+	2+	3+	4+	5+	Initials	
DQ smear	[ ]	[ ]	[ ]	[ ]	[ ]	[ ]	______	D D M M Y Y

Comments: ______________________________

Signature (Investigator/Subinvestigator): ______________ Date: |_|_|_|_|_|_| (D D M M Y Y)

Note: If the species is *L. aethiopica* the volunteer will be referred to his primary health care physician as per protocol. Physician Signature: ______________________

WR279396	Subject's Initials	Subject's Study Number
Log No.		

PARASITOLOGY LABORATORY
PATIENT DATA REPORT

Specimen(s) obtained: Yes: ______ No: ______

Date [| | | | |] D D M M Y Y Time [|] H H : [|] M M

Specimen(s) obtained by: ______________________________
Last name, First Name MI

Specimen(s) logged into (lab): Time [|] H H : [|] M M Lab ID Number: ______________

Source of specimen: (mark one only)

Lesion: [ ] Lymph Node: [ ] Other: [ ]

Lesion Number: ______________ Lesion Site from Figure: ______________

Parasitology Laboratory Assessment:

Culture:

		Date Positive	Species Identified	Initials
SCH 1	D D M M Y Y	D D M M Y Y	______	______
SCH 2	D D M M Y Y	D D M M Y Y	______	______
SCH 3	D D M M Y Y	D D M M Y Y	______	______

	Neg	1+	2+	3+	4+	5+	Initials	
DQ smear	[ ]	[ ]	[ ]	[ ]	[ ]	[ ]	______	D D M M Y Y

Comments: ______________________________

____

____

Signature (Investigator/Subinvestigator): ______________ Date: [| | | | |] D D M M Y Y

Note: If the species is *L. aethiopica* the volunteer will be referred to his primary health care physician as per protocol. Physician Signature: ______________________

WR279396	Subject's Initials	Subject's Study Number
Log No.		

PARASITOLOGY LABORATORY
PATIENT DATA REPORT

Specimen(s) obtained: Yes: ______ No: ______

Date D D M M Y Y Time H H : M M

Specimen(s) obtained by: ______________________
Last name, First Name MI

Specimen(s) logged into (lab): Time H H : M M Lab ID Number: ____________

Source of specimen: (mark one only)

Lesion: ☐ Lymph Node: ☐ Other: ☐

Lesion Number: ____________ Lesion Site from Figure: ____________

Parasitology Laboratory Assessment:

Culture:

		Date Positive	Species Identified	Initials
SCH 1	D D M M Y Y	D D M M Y Y	____________	____
SCH 2	D D M M Y Y	D D M M Y Y	____________	____
SCH 3	D D M M Y Y	D D M M Y Y	____________	____

	Neg	1+	2+	3+	4+	5+	Initials	
DQ smear	☐	☐	☐	☐	☐	☐	____	D D M M Y Y

Comments: ______________________

____

____

Signature (Investigator/Subinvestigator): ____________ Date: D D M M Y Y

Note: If the species is *L. aethiopica* the volunteer will be referred to his primary health care physician as per protocol. Physician Signature:

WR279396	Subject's Initials	Subject's Study Number
Log No.		

PARASITOLOGY LABORATORY
PATIENT DATA REPORT

Specimen(s) obtained: Yes: ______ No: ______

Date |_|_|_|_|_|_| D D M M Y Y Time |_|_| : |_|_| H H : M M

Specimen(s) obtained by: ______________________________
Last name, First Name MI

Specimen(s) logged into (lab): Time |_|_| : |_|_| H H M M Lab ID Number: ______________

Source of specimen: (mark one only)

Lesion: ☐ Lymph Node: ☐ Other: ☐

Lesion Number: ______________ Lesion Site from Figure: ________

Parasitology Laboratory Assessment:

Culture:

		Date Positive	Species Identified	Initials
SCH 1	D D M M Y Y	D D M M Y Y	______	______
SCH 2	D D M M Y Y	D D M M Y Y	______	______
SCH 3	D D M M Y Y	D D M M Y Y	______	______

	Neg	1+	2+	3+	4+	5+	Initials	
DQ smear	☐	☐	☐	☐	☐	☐	______	D D M M Y Y

Comments: ______________________________________

____

____

Signature (Investigator/Subinvestigator): ______________ Date: |_|_|_|_|_|_| D D M M Y Y

Note: If the species is *L. aethiopica* the volunteer will be referred to his primary health care physician as per protocol. Physician Signature:

Day -1

WR279396	Subject's Initials	Subject's Study Number
Log No.		

ENTRANCE EXAM & LABORATORY TEST CHECK LIST

HEARING TEST AND ROMBERG TEST

Was the hearing (eighth nerve) test high and low tones conducted?
If yes, have the results been recorded in the Physical Examination report form? If not, PLEASE DO IT !

Initial When Completed ☐

Was the Romberg test conducted?
If yes, have the results been recorded in the Physical Examination report form? If not, PLEASE DO IT !

Initial When Completed ☐

HEMATOLOGY AND BIOCHEMISTRY

Has a blood sample been taken for hematological and biochemical analysis?
If yes, have the results been recorded in the Hematology, Biochemistry & Urinalysis Patient data report form. If not, PLEASE DO IT !
Hematology: 5ml/Purple Top (EDTA)   Biochemistry: 10ml/Red Top

Initial When Completed ☐

URINALYSIS

Has a urine sample been taken for urinalysis? .
If yes, have the results been recorded in the Hematology, Biochemistry & Urinalysis Patient data report form. If not, PLEASE DO IT !
Urinalysis: 50-100 ml urine sample.

Initial When Completed ☐

BLOOD PHARMACOKINETIC SAMPLES

Has the baseline blood sample been taken?
If yes, have the n=3 1ml samples been deposited in the freezer, and the samples logged in the Pharmacokinetics Samples Log List report form? If not, PLEASE DO IT!
5ml/Purple Top

Initial When Completed ☐

URINE PHARMACOKINETIC SAMPLES

Has the baseline urine sample been taken?
If yes, have the n=3 1ml samples been deposited in the freezer, and the samples logged in the Pharmacokinetics Samples Log List report form? If not, PLEASE DO IT !
50-100 ml clean catch, transfer n=4 1ml samples

Initial When Completed ☐

LESION MEASUREMENTS (ULCER / LYMPH NODE)

Has the lesion(s) measurements been taken?
If yes, have the results been recorded in the Lesion(s) Clinical Findings report form? If not, PLEASE DO IT !

Initial When Completed ☐

CLINICAL EVALUATION OF THE LESION(S)

Was the lesion(s) clinically evaluated? If yes, have the results been recorded in the Lesion(s) Clinical Findings report form? If not, PLEASE DO IT !

Initial When Completed ☐

PHOTOGRAPH(S) OF PARASITOLOGIC-LESION(S)

Has a photograph(s) of the leishmaniasis lesion(s) been taken?
If yes, have the results been recorded in the WR 279396 Photograph Lesion Patient Data report form? If not, PLEASE DO IT !

Initial When Completed ☐

PARASITOLOGIC -LEISHMANIASIS TESTS

Has tissue samples been taken for amastigote detection (visualization), and/or cultures done?
If yes, have the results been recorded in the Parasitology Laboratory Patient Data report form? If not, PLEASE DO IT!
Biopsy: 4mm tissue sample

Initial When Completed ☐

WR279396	Subject's Initials	Subject's Study Number
Log No.		

CHECK LIST

**CONSENT FORM**<br>Has a consent form been obtained?	Yes ☐	No ☐
**DEMOGRAPHIC DETAILS**<br>Have the deomographic details been obtained?	Yes ☐	No ☐
**MEDICAL HISTORY**<br>Has the medical history been completed?	Yes ☐	No ☐
**HISTORY OF LEISHMANIASIS**<br>Has the history of leishmaniasis been completed?	Yes ☐	No ☐
**PHYSICAL EXAMINATION**<br>Has the physical examination been completed?	Yes ☐	No ☐
**DERMATOLOGY EXAM**<br>Was a dermatology exam conducted?	Yes ☐	No ☐
**HEARING TEST AND ROMBERG TEST**<br>Was the hearing (eighth nerve) test high and low tones conducted?	Yes ☐	No ☐
Was the Romberg test conducted?	Yes ☐	No ☐
**HEMATOLOGY AND BIOCHEMISTRY**<br>Has a blood sample been taken for hematological and biochemical analysis?<br>Hematology: 5 ml/ Purple Top (DTA) Biochemistry: 10 ml / Red Top<br>Pregnancy test (Women ONLY)	Yes ☐	No ☐
**URINALYSIS**<br>Has a urine sample been taken for urinalysis?<br>Urinalysis: 50 - 100 ml urine.	Yes ☐	No ☐

WR279396	Subject's Initials	Subject's Study Number
Log No.		

CHECK LIST

BLOOD PHARMACOKINETIC SAMPLES

Has the baseline blood sample been taken? Yes ☐ No ☐

Purple Top collection tube (5ml with EDTA)/ n=3 0.5 ml samples

URINE PHARMACOKINETIC SAMPLES

Has the baseline urine sample been taken? Yes ☐ No ☐

n=4 1ml samples

LESION MEASUREMENTS (ULCER)

Have the lesion(s) measurements been taken? Yes ☐ No ☐

CLINICAL EVALUATION OF THE LESION(S)

Was the lesion(s) clinically evaluated? Yes ☐ No ☐

PARASITOLOGIC-LESION(S) PHOTOGRAPH(S)

Has a photograph(s) of the leishmaniasis lesion(s) been taken? Yes ☐ No ☐

PARASITOLOGIC -LEISHMANIASIS TESTS

Have tissue samples been taken for amastigote detection (visualization)? Yes ☐ No ☐

WR279396	Subject's Initials	Subject's Study Number
Log No.		

INCLUSION / EXCLUSION CHECKLIST

		Yes	No
**A.**	**Consent Forms**		
1.	**Consent Form** signed?	☐	☐
2.	**Consent Explanation** signed?	☐	☐
3.	**Volunteer Donation Form** signed?	☐	☐
**B.**	**Inclusion Criteria**		
1.	Is the volunteer between 5 - 75 years of age?	☐	☐
2.	Is the lesion primarily of ulcerative type (i.e. NOT verrucous or nodular)?	☐	☐
3.	Have cutaneous leismaniasis been proven parasitologically?	☐	☐

If the answer to any of the above inclusion questions is NO the volunteer is not eligible for the study and must not continue. Please complete the **Study Completion Page**.

		Yes	No
**C.**	**Exclusion Criteria (Explain below):**		
1.	Drug intolerance?<br>History of known or suspected hypersensitivity or idiosyncratic reactions to aminoglycosides.	☐	☐
2.	Taken other antileishmanial drugs?<br>Previous use (within six months) of antileishmanial drugs or present use of routinely nephrotoxic or ototoxic drugs.	☐	☐
3.	Previous confirmed leishmaniasis?	☐	☐
4.	Potential for follow-up?<br>Have less than seven months time remaining in the area.	☐	☐
5.	Extent of disease<br>Total cumulative surface area of all lesions > 2000 mm^2	☐	☐
6.	Locations of disease<br>Lesions near the eye, mucosal involvement.	☐	☐
7.	Disseminated disease?<br>Clinical significant lymphadenitis or when nodules are painful and greater than 1 cm in size in the lymphatic drainage of the ulcer.	☐	☐
8.	Concomitant medical problems?<br>Significant medical problems of the kidney, liver or other organ systems.	☐	☐
9.	Hearing and Romberg tests abnormalities?	☐	☐
10.	Pregnant?	☐	☐

Explain any exclusion criteria checked "Yes": ______________________

____

If the answer to any of the above exclusion questions is YES, then the volunteer is not eligible for the study and must not continue. Please complete the **Study Completion Page**

	Yes	No
Is the volunteer eligible to continue the study?	☐	☐

Investigator's Signature: ______________________

148

Day 1

WR279396	Subject's Initials	Subject's Study Number
Log No.		

Visit Date |_|_|_|_|_|_| D D M M Y Y

Has the volunteer felt unwell since his/her last visit? Yes ☐ No ☐

Comments: ______________________

Has the volunteer used any medication apart from the study medication since the last visit? If yes, please record details in the concomitant medication section. Yes ☐ No ☐

DRUG ADMINISTRATION

	Date	Time: hh:mm	Time: hh:mm	Drug Vial No.
DAY 1	|_|_|_|_|_|_| D D M M Y Y			

Was the drug administration card reviewed? Yes ☐ No ☐

Investigator's Signature: ______________________ |_|_|_|_|_|_| D D M M Y Y

Has the volunteer had side effects? In either case, please record the toxicity grades below. Yes ☐ No ☐

	LOCAL TOXICITY			SYSTEMIC TOXICITY			
Immediate Toxicity Time: hh:mm	Pain Grade	Erythema Grade	Edema Grade	Vertigo Grade	Tinnitus Grade	Hearing Grade	Initials
	☐	☐	☐	☐	☐	☐	____
Delayed Toxicity Time: hh:mm	Pain Grade	Erythema Grade	Edema Grade	Vertigo Grade	Tinnitus Grade	Hearing Grade	Initials
	☐	☐	☐	☐	☐	☐	____

Pain	None 0	Mild 1	Moderate 2	Severe 3	
Erythema	None 0	Mild (barely perceptible) 1	Moderate (well defined) 2	Severe (very red) 3	
Edema	None 0	Mild (barely perceptible) 1	Moderate (well defined) 2	Severe (raised >2mm) 3	Exfoliative dermatitis 4
Vertigo	None 0	Mild 1	Moderate 2	Severe 3	
Tinnitus	None 0	Mild 1	Moderate 2	Severe 3	
Diminished hearing	None 0	Mild 1	Moderate 2	Severe 3	Profound 4

Note: For **Erythema & Edema**, a Grade 3 reaction require immediate notification to medical monitor. Grade 4 reactions require immediate termination of treatment and an ADVERSE EVENT NOTIFICATION. **Forvariables not explained directly in the form**, please refer to the PROTOCOL **10.4.** for Grade clarification, requirements for immediate notification to medical monitor, and ADVERSE EVENT NOTIFICATION.

Day 2

WR279396	Subject's Initials	Subject's Study Number
Log No.		

Visit Date ⎣ ⎣ ⎣ ⎣ ⎣ ⎣ ⎦ D D M M Y Y

	Yes	No
Has the volunteer felt unwell since his/her last visit? If yes, comment on page 61.	☐	☐
Has the volunteer used any medication apart from the study medication since the last visit? If yes, please record details in the Concomitant Medication section.	☐	☐
Has the volunteer had side effects? In either case, please record the toxicity grades below.	☐	☐

	LOCAL TOXICITY			SYSTEMIC TOXICITY			
	Pain Grade	Erythema Grade	Edema Grade	Vertigo Grade	Tinnitus Grade	Hearing Grade	Initials
Immediate Toxicity Time: hh:mm	☐	☐	☐	☐	☐	☐	____
Delayed Toxicity Time: hh:mm	☐	☐	☐	☐	☐	☐	____

Pain	None 0	Mild 1	Moderate 2	Severe 3	
Erythema	None 0	Mild (barely perceptible) 1	Moderate (well defined) 2	Severe (very red) 3	
Edema	None 0	Mild (barely perceptible) 1	Moderate (well defined) 2	Severe (raised >2mm) 3	Exfoliative dermatitis 4
Vertigo	None 0	Mild 1	Moderate 2	Severe 3	
Tinnitus	None 0	Mild 1	Moderate 2	Severe 3	
Diminished hearing	None 0	Mild 1	Moderate 2	Severe 3	Profound 4

Note: For **Erythema & Edema**, a Grade 3 reaction requires immediate notification to medical monitor. Grade 4 reactions require immediate termination of treatment and an ADVERSE EVENT NOTIFICATION. For **variables not explained directly in the form**, please refer to the PROTOCOL **10.4.** for Grade clarification, requirements for immediate notification to medical monitor, and ADVERSE EVENT NOTIFICATION.

DRUG ADMINISTRATION

	Date	Time: hh:mm	Time: hh:mm	Drug Vial No.
DAY 2	⎣ ⎣ ⎣ ⎣ ⎣ ⎣ ⎦ D D M M Y Y			

Was the drug administration card reviewed? Yes ☐ No ☐

Investigator's Signature: ____________________ ⎣ ⎣ ⎣ ⎣ ⎣ ⎣ ⎦ D D M M Y Y

Please make an appointment for the volunteer to come back tomorrow.

Day 3

WR279396	Subject's Initials	Subject's Study Number
Log No.		

Visit Date |_|_|_|_|_|_| D D M M Y Y

	Yes	No
Has the volunteer felt unwell since his/her last visit? If yes, comment on page 61.	☐	☐
Has the volunteer used any medication apart from the study medication since the last visit? If yes, please record details in the Concomitant Medication section.	☐	☐
Has the volunteer had side effects? In either case, please record the toxicity grades below.	☐	☐

	LOCAL TOXICITY			SYSTEMIC TOXICITY			
Immediate Toxicity Time: hh:mm	Pain Grade	Erythema Grade	Edema Grade	Vertigo Grade	Tinnitus Grade	Hearing Grade	Initials
	☐	☐	☐	☐	☐	☐	____
Delayed Toxicity Time: hh:mm	Pain Grade	Erythema Grade	Edema Grade	Vertigo Grade	Tinnitus Grade	Hearing Grade	Initials
	☐	☐	☐	☐	☐	☐	____

Pain	None 0	Mild 1	Moderate 2	Severe 3	
Erythema	None 0	Mild (barely perceptible) 1	Moderate (well defined) 2	Severe (very red) 3	
Edema	None 0	Mild (barely perceptible) 1	Moderate (well defined) 2	Severe (raised >2mm) 3	Exfoliative dermatitis 4
Vertigo	None 0	Mild 1	Moderate 2	Severe 3	
Tinnitus	None 0	Mild 1	Moderate 2	Severe 3	
Diminished hearing	None 0	Mild 1	Moderate 2	Severe 3	Profound 4

Note: For **Erythema & Edema**, a Grade 3 reaction requires immediate notification to medical monitor. Grade 4 reactions require immediate termination of treatment and an ADVERSE EVENT NOTIFICATION. **Forvariables not explained directly in the form**, please refer to the PROTOCOL **10.4.** for Grade clarification, requirements for immediate notification to medical monitor, and ADVERSE EVENT NOTIFICATION.

DRUG ADMINISTRATION

	Date	Time: hh:mm	Time: hh:mm	Drug Vial No.
DAY 3	|_|_|_|_|_|_| D D M M Y Y			

Was the drug administration card reviewed? Yes ☐ No ☐

Investigator's Signature: ______________________ |_|_|_|_|_|_| D D M M Y Y

Please make an appointment for the volunteer to come back tomorrow

Day 4

WR279396	Subject's Initials	Subject's Study Number
Log No.		

Visit Date |__|__|__|__|__|__| D D M M Y Y

	Yes	No
Has the volunteer felt unwell since his/her last visit? If yes, comment on page 61.	☐	☐
Has the volunteer used any medication apart from the study medication since the last visit? If yes, please record details in the Concomitant Medication section.	☐	☐
Has the volunteer had side effects? In either case, please record the toxicity grades below.	☐	☐

	LOCAL TOXICITY			SYSTEMIC TOXICITY			
	Pain Grade	Erythema Grade	Edema Grade	Vertigo Grade	Tinnitus Grade	Hearing Grade	Initials
Immediate Toxicity Time: hh:mm	☐	☐	☐	☐	☐	☐	____
	Pain Grade	Erythema Grade	Edema Grade	Vertigo Grade	Tinnitus Grade	Hearing Grade	Initials
Delayed Toxicity Time: hh:mm	☐	☐	☐	☐	☐	☐	____

Pain	None 0	Mild 1	Moderate 2	Severe 3	
Erythema	None 0	Mild (barely perceptible) 1	Moderate (well defined) 2	Severe (very red) 3	
Edema	None 0	Mild (barely perceptible) 1	Moderate (well defined) 2	Severe (raised >2mm) 3	Exfoliative dermatitis 4
Vertigo	None 0	Mild 1	Moderate 2	Severe 3	
Tinnitus	None 0	Mild 1	Moderate 2	Severe 3	
Diminished hearing	None 0	Mild 1	Moderate 2	Severe 3	Profound 4

Note: For **Erythema & Edema**, a Grade 3 reaction requires immediate notification to medical monitor. Grade 4 reactions require immediate termination of treatment and an ADVERSE EVENT NOTIFICATION. **For variables not explained directly in the form**, please refer to the PROTOCOL **10.4.** for Grade clarification, requirements for immediate notification to medical monitor, and ADVERSE EVENT NOTIFICATION.

DRUG ADMINISTRATION

	Date	Time: hh:mm	Time: hh:mm	Drug Vial No.
DAY 4	|__|__|__|__|__|__| D D M M Y Y			

Was the drug administration card reviewed? Yes ☐ No ☐

Investigator's Signature: ______________________ |__|__|__|__|__|__| D D M M Y Y

Please make an appointment for the volunteer to come back tomorrow

Day 5

WR279396	Subject's Initials	Subject's Study Number
Log No.		

Visit Date |_|_|_|_|_|_| D D M M Y Y

	Yes	No
Has the volunteer felt unwell since his/her last visit? If yes, comment on page 61.	☐	☐
Has the volunteer used any medication apart from the study medication since the last visit? If yes, please record details in the Concomitant Medication section.	☐	☐
Has the volunteer had side effects? In either case, please record the toxicity grades below.	☐	☐

	LOCAL TOXICITY			SYSTEMIC TOXICITY			
Immediate Toxicity Time: hh:mm	Pain Grade	Erythema Grade	Edema Grade	Vertigo Grade	Tinnitus Grade	Hearing Grade	Initials
	☐	☐	☐	☐	☐	☐	____
Delayed Toxicity Time: hh:mm	Pain Grade	Erythema Grade	Edema Grade	Vertigo Grade	Tinnitus Grade	Hearing Grade	Initials
	☐	☐	☐	☐	☐	☐	____

Pain	None 0	Mild 1	Moderate 2	Severe 3	
Erythema	None 0	Mild (barely perceptible) 1	Moderate (well defined) 2	Severe (very red) 3	
Edema	None 0	Mild (barely perceptible) 1	Moderate (well defined) 2	Severe (raised >2mm) 3	Exfoliative dermatitis 4
Vertigo	None 0	Mild 1	Moderate 2	Severe 3	
Tinnitus	None 0	Mild 1	Moderate 2	Severe 3	
Diminished hearing	None 0	Mild 1	Moderate 2	Severe 3	Profound 4

Note: For **Erythema & Edema**, a Grade 3 reaction requires immediate notification to medical monitor. Grade 4 reactions require immediate termination of treatment and an ADVERSE EVENT NOTIFICATION. For **variables not explained directly in the form**, please refer to the PROTOCOL **10.4.** for Grade clarification, requirements for immediate notification to medical monitor, and ADVERSE EVENT NOTIFICATION.

DRUG ADMINISTRATION

	Date	Time: hh:mm	Time: hh:mm	Drug Vial No.
DAY 5	|_|_|_|_|_|_| D D M M Y Y			

Was the drug administration card reviewed? Yes ☐ No ☐

Investigator's Signature: ____________________ |_|_|_|_|_|_| D D M M Y Y

Please make an appointment for the volunteer to come back tomorrow

Day 6

WR279396	Subject's Initials	Subject's Study Number
Log No.		

Visit Date |_|_|_|_|_|_| D D M M Y Y

	Yes	No
Has the volunteer felt unwell since his/her last visit? If yes, comment on page 61.	☐	☐
Has the volunteer used any medication apart from the study medication since the last visit? If yes, please record details in the Concomitant Medication section.	☐	☐
Has the volunteer had side effects? In either case, please record the toxicity grades below.	☐	☐

	LOCAL TOXICITY			SYSTEMIC TOXICITY			
	Pain Grade	Erythema Grade	Edema Grade	Vertigo Grade	Tinnitus Grade	Hearing Grade	Initials
Immediate Toxicity Time: hh:mm	☐	☐	☐	☐	☐	☐	____
Delayed Toxicity Time: hh:mm	☐	☐	☐	☐	☐	☐	____

Pain	None 0	Mild 1	Moderate 2	Severe 3	
Erythema	None 0	Mild (barely perceptible) 1	Moderate (well defined) 2	Severe (very red) 3	
Edema	None 0	Mild (barely perceptible) 1	Moderate (well defined) 2	Severe (raised >2mm) 3	Exfoliative dermatitis 4
Vertigo	None 0	Mild 1	Moderate 2	Severe 3	
Tinnitus	None 0	Mild 1	Moderate 2	Severe 3	
Diminished hearing	None 0	Mild 1	Moderate 2	Severe 3	Profound 4

Note: For **Erythema & Edema**, a Grade 3 reaction requires immediate notification to medical monitor. Grade 4 reactions require immediate termination of treatment and an ADVERSE EVENT NOTIFICATION. For **variables not explained directly in the form**, please refer to the PROTOCOL **10.4.** for Grade clarification, requirements for immediate notification to medical monitor, and ADVERSE EVENT NOTIFICATION.

DRUG ADMINISTRATION

	Date	Time: hh:mm	Time: hh:mm	Drug Vial No.
DAY 6	|_|_|_|_|_|_| D D M M Y Y			

Was the drug administration card reviewed? Yes ☐ No ☐

Investigator's Signature: ______________________ |_|_|_|_|_|_| D D M M Y Y

Please make an appointment for the volunteer to come back tomorrow

WR279396	Subject's Initials	Subject's Study Number
Log No.		

Visit Date ⌊ ⌊ ⌊ ⌊ ⌊ ⌊ ⌋
D D M M Y Y

	Yes	No
Has the volunteer felt unwell since his/her last visit? if yes, comment on page 61.	☐	☐
Has the volunteer used any medication apart from the study medication since the last visit? If yes, please record details in the Concomitant Medication section.	☐	☐
Has the volunteer had side effects? In either case, please record the toxicity grades below.	☐	☐

	LOCAL TOXICITY			SYSTEMIC TOXICITY			
Immediate Toxicity Time: hh:mm	Pain Grade	Erythema Grade	Edema Grade	Vertigo Grade	Tinnitus Grade	Hearing Grade	Initials
Delayed Toxicity Time: hh:mm	Pain Grade	Erythema Grade	Edema Grade	Vertigo Grade	Tinnitus Grade	Hearing Grade	Initials

Pain	None 0	Mild 1	Moderate 2	Severe 3	
Erythema	None 0	Mild (barely perceptible) 1	Moderate (well defined) 2	Severe (very red) 3	
Edema	None 0	Mild (barely perceptible) 1	Moderate (well defined) 2	Severe (raised >2mm) 3	Exfoliative dermatitis 4
Vertigo	None 0	Mild 1	Moderate 2	Severe 3	
Tinnitus	None 0	Mild 1	Moderate 2	Severe 3	
Diminished hearing	None 0	Mild 1	Moderate 2	Severe 3	Profound 4

Note: For **Erythema & Edema**, a Grade 3 reaction requires immediate notification to medical monitor. Grade 4 reactions require immediate termination of treatment and an ADVERSE EVENT NOTIFICATION. For**variables not explained directly in the form**, please refer to the PROTOCOL **10.4.** for Grade clarification, requirements for immediate notification to medical monitor, and ADVERSE EVENT NOTIFICATION.

DRUG ADMINISTRATION

	Date	Time: hh:mm	Time: hh:mm	Drug Vial No.
DAY 7	⌊ ⌊ ⌊ ⌊ ⌊ ⌊ ⌋<br>D D M M Y Y			

Was the drug administration card reviewed? Yes ☐ No ☐

Investigator's Signature: ______________________ ⌊ ⌊ ⌊ ⌊ ⌊ ⌊ ⌋
D D M M Y Y

Please make an appointment for the volunteer to come back tomorrow

Day 8

WR279396	Subject's Initials	Subject's Study Number
Log No.		

Visit Date |_|_|_|_|_|_| D D M M Y Y

	Yes	No
Has the volunteer felt unwell since his/her last visit? If yes, comment on page 61.	☐	☐
Has the volunteer used any medication apart from the study medication since the last visit? If yes, please record details in the Concomitant Medication section.	☐	☐
Has the volunteer had side effects? In either case, please record the toxicity grades below.	☐	☐

	LOCAL TOXICITY			SYSTEMIC TOXICITY			
	Pain Grade	Erythema Grade	Edema Grade	Vertigo Grade	Tinnitus Grade	Hearing Grade	Initials
Immediate Toxicity Time: hh:mm	☐	☐	☐	☐	☐	☐	____
Delayed Toxicity Time: hh:mm	☐	☐	☐	☐	☐	☐	____

Pain	None 0	Mild 1	Moderate 2	Severe 3	
Erythema	None 0	Mild (barely perceptible) 1	Moderate (well defined) 2	Severe (very red) 3	
Edema	None 0	Mild (barely perceptible) 1	Moderate (well defined) 2	Severe (raised >2mm) 3	Exfoliative dermatitis 4
Vertigo	None 0	Mild 1	Moderate 2	Severe 3	
Tinnitus	None 0	Mild 1	Moderate 2	Severe 3	
Diminished hearing	None 0	Mild 1	Moderate 2	Severe 3	Profound 4

Note: For **Erythema & Edema**, a Grade 3 reaction requires immediate notification to medical monitor. Grade 4 reactions require immediate termination of treatment and an ADVERSE EVENT NOTIFICATION. For **variables not explained directly in the form**, please refer to the PROTOCOL **10.4.** for Grade clarification, requirements for immediate notification to medical monitor, and ADVERSE EVENT NOTIFICATION.

DRUG ADMINISTRATION

	Date	Time: hh:mm	Time: hh:mm	Drug Vial No.
DAY 8	|_|_|_|_|_|_| D D M M Y Y			

Was the drug administration card reviewed? Yes ☐ No ☐

Investigator's Signature: ____________________ |_|_|_|_|_|_| D D M M Y Y

Please make an appointment for the volunteer to come back tomorrow

Day 9

WR279396	Subject's Initials	Subject's Study Number
Log No.		

Visit Date ☐☐☐☐☐☐ (D D M M Y Y)

	Yes	No
Has the volunteer felt unwell since his/her last visit? If yes, comment on page 61.	☐	☐
Has the volunteer used any medication apart from the study medication since the last visit? If yes, please record details in the Concomitant Medication section.	☐	☐
Has the volunteer had side effects? In either case, please record the toxicity grades below.	☐	☐

	LOCAL TOXICITY			SYSTEMIC TOXICITY			
	Pain Grade	Erythema Grade	Edema Grade	Vertigo Grade	Tinnitus Grade	Hearing Grade	Initials
Immediate Toxicity Time: hh:mm	☐	☐	☐	☐	☐	☐	
Delayed Toxicity Time: hh:mm	☐	☐	☐	☐	☐	☐	

Pain	None 0	Mild 1	Moderate 2	Severe 3	
Erythema	None 0	Mild (barely perceptible) 1	Moderate (well defined) 2	Severe (very red) 3	
Edema	None 0	Mild (barely perceptible) 1	Moderate (well defined) 2	Severe (raised >2mm) 3	Exfoliative dermatitis 4
Vertigo	None 0	Mild 1	Moderate 2	Severe 3	
Tinnitus	None 0	Mild 1	Moderate 2	Severe 3	
Diminished hearing	None 0	Mild 1	Moderate 2	Severe 3	Profound 4

Note: For **Erythema & Edema**, a Grade 3 reaction requires immediate notification to medical monitor. Grade 4 reactions require immediate termination of treatment and an ADVERSE EVENT NOTIFICATION. For**variables not explained directly in the form**, please refer to the PROTOCOL **10.4.** for Grade clarification, requirements for immediate notification to medical monitor, and ADVERSE EVENT NOTIFICATION.

DRUG ADMINISTRATION

	Date	Time: hh:mm	Time: hh:mm	Drug Vial No.
DAY 9	☐☐☐☐☐☐ (D D M M Y Y)			

Was the drug administration card reviewed? Yes ☐ No ☐

Investigator's Signature: ______________________ ☐☐☐☐☐☐ (D D M M Y Y)

Please make an appointment for the volunteer to come back tomorrow.

WR279396	Subject's Initials	Subject's Study Number
Log No.		

Visit Date D D M M Y Y

	Yes	No
Has the volunteer felt unwell since his/her last visit? If yes, comment on page 61.	☐	☐
Has the volunteer taken any medication apart from the study medication since the last visit? If yes, please record details in the Concomitant Medication section.	☐	☐
Has the volunteer had side effects? In either case, please record the toxicity grades below.	☐	☐

	LOCAL TOXICITY			SYSTEMIC TOXICITY			
Immediate Toxicity Time: hh:mm	Pain Grade	Erythema Grade	Edema Grade	Vertigo Grade	Tinnitus Grade	Hearing Grade	Initials
Delayed Toxicity Time: hh:mm	Pain Grade	Erythema Grade	Edema Grade	Vertigo Grade	Tinnitus Grade	Hearing Grade	Initials

Pain	None 0	Mild 1	Moderate 2	Severe 3	
Erythema	None 0	Mild (barely perceptible) 1	Moderate (well defined) 2	Severe (very red) 3	
Edema	None 0	Mild (barely perceptible) 1	Moderate (well defined) 2	Severe (raised >2mm) 3	Exfoliative dermatitis 4
Vertigo	None 0	Mild 1	Moderate 2	Severe 3	
Tinnitus	None 0	Mild 1	Moderate 2	Severe 3	
Diminished hearing	None 0	Mild 1	Moderate 2	Severe 3	Profound 4

Note: For **Erythema & Edema**, a Grade 3 reaction requires immediate notification to medical monitor. Grade 4 reactions require immediate termination of treatment and an ADVERSE EVENT NOTIFICATION. For **variables not explained directly in the form**, please refer to the PROTOCOL **10.4.** for Grade clarification, requirements for immediate notification to medical monitor, and ADVERSE EVENT NOTIFICATION.

Was the hearing eighth nerve test: high & low tones conducted ?
If not, ! DO IT!

Initial When Completed

HEARING TEST

	Normal	Abnormal
Hearing Test - High Tone	☐	☐
Hearing Test - Low Tone	☐	☐

	250/256	500/512	1,000/1,024	2,000/2,048	3,000/2,896
Right					
Left					

	4,000/4,096	6,000/6,144	8,000/8,192
Right			
Left			

WR279396	Subject's Initials	Subject's Study Number
Log No.		

Was the Romberg test conducted ? If not, DO IT!

Initial When Completed ☐

ROMBERG TEST

Romberg Test	☐	☐

Comments: ______________________

Signature (Audiologist): ______________________

Print Name (Last, First, MI): ______________________

Initial When Completed ☐

Has a blood sample been taken for biochemical analysis (creatinine)? Red top (10 ml) and processed as per SOP. If yes, please record the results in the Hematology and Biochemistry report form.
If not, DO IT !

Initial When Completed ☐

Has a blood sample been taken for pharmacokinetic analysis ?
Just prior to the next application ("trough") and one hour after drug application ("peak"). Use a Purple Top collection tube (5ml with EDTA). Has the n=3 0.5ml samples been deposited in the freezer, and the samples logged in the Pharmokinetics Sample Log List report form. If not, DO IT !

Initial When Completed ☐

Has a 24 hour urine collection been taken to determine total absorbed drugs by taking 50-100 ml clean catch and transfering n=4 1ml samples into cryotubes tubes ? Have the n=4 1ml samples been deposited in the freezer, and the samples logged in the Pharmokinetics Samples Log List report form ? If not, DO IT !

DRUG ADMINISTRATION

	Date	Time: hh:mm	Time: hh:mm	Drug Vial No.
DAY 10	_ _ _ _ _ _<br>D D M M Y Y			

Was the drug administration card reviewed ? Yes ☐ No ☐

Investigator's Signature: ______________________ _ _ _ _ _ _
D D M M Y Y

Please make an appointment for the volunteer to come back tomorrow.

Day 11

WR279396	Subject's Initials	Subject's Study Number
Log No.		

Visit Date [| | | | |] D D M M Y Y

	Yes	No
Has the volunteer felt unwell since his/her last visit? If yes, comment on page 61.	☐	☐
Has the volunteer used any medication apart from the study medication since the last visit? If yes, please record details in the Concomitant Medication section.	☐	☐
Has the volunteer had side effects? In either case, please record the toxicity grades below.	☐	☐

	LOCAL TOXICITY			SYSTEMIC TOXICITY			
	Pain Grade	Erythema Grade	Edema Grade	Vertigo Grade	Tinnitus Grade	Hearing Grade	Initials
Immediate Toxicity Time: hh:mm	☐	☐	☐	☐	☐	☐	____
Delayed Toxicity Time: hh:mm	☐	☐	☐	☐	☐	☐	____

Pain	None 0	Mild 1	Moderate 2	Severe 3	
Erythema	None 0	Mild (barely perceptible) 1	Moderate (well defined) 2	Severe (very red) 3	
Edema	None 0	Mild (barely perceptible) 1	Moderate (well defined) 2	Severe (raised >2mm) 3	Exfoliative dermatitis 4
Vertigo	None 0	Mild 1	Moderate 2	Severe 3	
Tinnitus	None 0	Mild 1	Moderate 2	Severe 3	
Diminished hearing	None 0	Mild 1	Moderate 2	Severe 3	Profound 4

Note: For **Erythema & Edema**, a Grade 3 reaction requires immediate notification to medical monitor. Grade 4 reactions require immediate termination of treatment and an ADVERSE EVENT NOTIFICATION. For **variables not explained directly in the form**, please refer to the PROTOCOL **10.4.** for Grade clarification, requirements for immediate notification to medical monitor, and ADVERSE EVENT NOTIFICATION.

DRUG ADMINISTRATION

	Date	Time: hh:mm	Time: hh:mm	Drug Vial No.
DAY 11	[| | | | |] D D M M Y Y			

Was the drug administration card reviewed? Yes ☐ No ☐

Investigator's Signature: ____________________ [| | | | |] D D M M Y Y

Please make an appointment for the volunteer to come back tomorrow

Day 12

WR279396	Subject's Initials	Subject's Study Number
Log No.		

Visit Date |_|_|_|_|_|_| D D M M Y Y

	Yes	No
Has the volunteer felt unwell since his/her last visit? If yes, comment on page 61.	☐	☐
Has the volunteer used any medication apart from the study medication since the last visit? If yes, please record details in the Concomitant Medication section.	☐	☐
Has the volunteer had side effects? In either case, please record the toxicity grades below.	☐	☐

	LOCAL TOXICITY			SYSTEMIC TOXICITY			
	Pain Grade	Erythema Grade	Edema Grade	Vertigo Grade	Tinnitus Grade	Hearing Grade	Initials
Immediate Toxicity Time: hh:mm	☐	☐	☐	☐	☐	☐	______
Delayed Toxicity Time: hh:mm	☐	☐	☐	☐	☐	☐	______

Pain	None 0	Mild 1	Moderate 2	Severe 3	
Erythema	None 0	Mild (barely perceptible) 1	Moderate (well defined) 2	Severe (very red) 3	
Edema	None 0	Mild (barely perceptible) 1	Moderate (well defined) 2	Severe (raised >2mm) 3	Exfoliative dermatitis 4
Vertigo	None 0	Mild 1	Moderate 2	Severe 3	
Tinnitus	None 0	Mild 1	Moderate 2	Severe 3	
Diminished hearing	None 0	Mild 1	Moderate 2	Severe 3	Profound 4

Note: For **Erythema & Edema**, a Grade 3 reaction requires immediate notification to medical monitor. Grade 4 reactions require immediate termination of treatment and an ADVERSE EVENT NOTIFICATION. For **variables not explained directly in the form**, please refer to the PROTOCOL **10.4.** for Grade clarification, requirements for immediate notification to medical monitor, and ADVERSE EVENT NOTIFICATION.

DRUG ADMINISTRATION

	Date	Time: hh:mm	Time: hh:mm	Drug Vial No.
DAY 12	|_|_|_|_|_|_| D D M M Y Y			

Was the drug administration card reviewed? Yes ☐ No ☐

Investigator's Signature: ______________________ |_|_|_|_|_|_| D D M M Y Y

Please make an appointment for the volunteer to come back tomorrow

Day 13

WR279396	Subject's Initials	Subject's Study Number
Log No.		

Visit Date ☐☐☐☐☐☐ (D D M M Y Y)

	Yes	No
Has the volunteer felt unwell since his/her last visit? If yes, comment on page 61.	☐	☐
Has the volunteer taken any medication apart from the study medication since the last visit? If yes, please record details in the Concomitant Medication section.	☐	☐
Has the volunteer had side effects? In either case, please record the toxicity grades below.	☐	☐

	LOCAL TOXICITY			SYSTEMIC TOXICITY			
Immediate Toxicity Time: hh:mm	Pain Grade	Erythema Grade	Edema Grade	Vertigo Grade	Tinnitus Grade	Hearing Grade	Initials
	☐	☐	☐	☐	☐	☐	____
Delayed Toxicity Time: hh:mm	Pain Grade	Erythema Grade	Edema Grade	Vertigo Grade	Tinnitus Grade	Hearing Grade	Initials
	☐	☐	☐	☐	☐	☐	____

Pain	None 0	Mild 1	Moderate 2	Severe 3	
Erythema	None 0	Mild (barely perceptible) 1	Moderate (well defined) 2	Severe (very red) 3	
Edema	None 0	Mild (barely perceptible) 1	Moderate (well defined) 2	Severe (raised >2mm) 3	Exfoliative dermatitis 4
Vertigo	None 0	Mild 1	Moderate 2	Severe 3	
Tinnitus	None 0	Mild 1	Moderate 2	Severe 3	
Diminished hearing	None 0	Mild 1	Moderate 2	Severe 3	Profound 4

Note: For **Erythema & Edema**, a Grade 3 reaction requires immediate notification to medical monitor. Grade 4 reactions require immediate termination of treatment and an ADVERSE EVENT NOTIFICATION. For **variables not explained directly in the form**, please refer to the PROTOCOL **10.4.** for Grade clarification, requirements for immediate notification to medical monitor, and ADVERSE EVENT NOTIFICATION.

DRUG ADMINISTRATION

	Date	Time: hh:mm	Time: hh:mm	Drug Vial No.
DAY 13	☐☐☐☐☐☐ (D D M M Y Y)			

Was the drug administration card reviewed? Yes ☐ No ☐

Investigator's Signature: ____________________ ☐☐☐☐☐☐ (D D M M Y Y)

Please make an appointment for the volunteer to come back tomorrow

Day 14

WR279396	Subject's Initials	Subject's Study Number
Log No.		

Visit Date |_|_|_|_|_|_| D D M M Y Y

	Yes	No
Has the volunteer felt unwell since his/her last visit? If yes, comment on page 61.	☐	☐
Has the volunteer taken any medication apart from the study medication since the last visit? If yes, please record details in the Concomitant Medication section.	☐	☐
Has the volunteer had side effects? In either case, please record the toxicity grades below.	☐	☐

	LOCAL TOXICITY			SYSTEMIC TOXICITY			
	Pain Grade	Erythema Grade	Edema Grade	Vertigo Grade	Tinnitus Grade	Hearing Grade	Initials
Immediate Toxicity Time: hh:mm	☐	☐	☐	☐	☐	☐	____
Delayed Toxicity Time: hh:mm	☐	☐	☐	☐	☐	☐	____

Pain	None 0	Mild 1	Moderate 2	Severe 3	
Erythema	None 0	Mild (barely perceptible) 1	Moderate (well defined) 2	Severe (very red) 3	
Edema	None 0	Mild (barely perceptible) 1	Moderate (well defined) 2	Severe (raised >2mm) 3	Exfoliative dermatitis 4
Vertigo	None 0	Mild 1	Moderate 2	Severe 3	
Tinnitus	None 0	Mild 1	Moderate 2	Severe 3	
Diminished hearing	None 0	Mild 1	Moderate 2	Severe 3	Profound 4

Note: For **Erythema & Edema**, a Grade 3 reaction requires immediate notification to medical monitor. Grade 4 reactions require immediate termination of treatment and an ADVERSE EVENT NOTIFICATION. For**variables not explained directly in the form**, please refer to the PROTOCOL **10.4.** for Grade clarification, requirements for immediate notification to medical monitor, and ADVERSE EVENT NOTIFICATION.

DRUG ADMINISTRATION

	Date	Time: hh:mm	Time: hh:mm	Drug Vial No.
DAY 14	|_|_|_|_|_|_| D D M M Y Y			

Was the drug administration card reviewed? Yes ☐ No ☐

Investigator's Signature: ______________________ |_|_|_|_|_|_| D D M M Y Y

Please make an appointment for the volunteer to come back tomorrow

Day 15

WR279396	Subject's Initials	Subject's Study Number
Log No.		

Visit Date |_|_|_|_|_|_| D D M M Y Y

	Yes	No
Has the volunteer felt unwell since his/her last visit? If yes, comment on page 61.	☐	☐
Has the volunteer used any medication apart from the study medication since the last visit? If yes, please record details in the Concomitant Medication section.	☐	☐
Has the volunteer had side effects? In either case, please record the toxicity grades below.	☐	☐

	LOCAL TOXICITY			SYSTEMIC TOXICITY			
Immediate Toxicity Time: hh:mm	Pain Grade	Erythema Grade	Edema Grade	Vertigo Grade	Tinnitus Grade	Hearing Grade	Initials
	☐	☐	☐	☐	☐	☐	____
Delayed Toxicity Time: hh:mm	Pain Grade	Erythema Grade	Edema Grade	Vertigo Grade	Tinnitus Grade	Hearing Grade	Initials
	☐	☐	☐	☐	☐	☐	____

Pain	None 0	Mild 1	Moderate 2	Severe 3	
Erythema	None 0	Mild (barely perceptible) 1	Moderate (well defined) 2	Severe (very red) 3	
Edema	None 0	Mild (barely perceptible) 1	Moderate (well defined) 2	Severe (raised >2mm) 3	Exfoliative dermatitis 4
Vertigo	None 0	Mild 1	Moderate 2	Severe 3	
Tinnitus	None 0	Mild 1	Moderate 2	Severe 3	
Diminished hearing	None 0	Mild 1	Moderate 2	Severe 3	Profound 4

Note: For **Erythema & Edema**, a Grade 3 reaction requires immediate notification to medical monitor. Grade 4 reactions require immediate termination of treatment and an ADVERSE EVENT NOTIFICATION. For **variables not explained directly in the form**, please refer to the PROTOCOL **10.4.** for Grade clarification, requirements for immediate notification to medical monitor, and ADVERSE EVENT NOTIFICATION.

DRUG ADMINISTRATION

	Date	Time: hh:mm	Time: hh:mm	Drug Vial No.
DAY 15	|_|_|_|_|_|_| D D M M Y Y			

Was the drug administration card reviewed? Yes ☐ No ☐

Investigator's Signature: ______________________ |_|_|_|_|_|_| D D M M Y Y

Please make an appointment for the volunteer to come back tomorrow

Day 16

WR279396	Subject's Initials	Subject's Study Number
Log No.		

Visit Date [ ][ ][ ][ ][ ][ ] D D M M Y Y

	Yes	No
Has the volunteer felt unwell since his/her last visit? If yes, comment on page 61.	[ ]	[ ]
Has the volunteer used any medication apart from the study medication since the last visit? If yes, please record details in the Concomitant Medication section.	[ ]	[ ]
Has the volunteer had side effects? In either case, please record the toxicity grades below.	[ ]	[ ]

	LOCAL TOXICITY			SYSTEMIC TOXICITY			
	Pain Grade	Erythema Grade	Edema Grade	Vertigo Grade	Tinnitus Grade	Hearing Grade	Initials
Immediate Toxicity Time: hh:mm							
Delayed Toxicity Time: hh:mm							

Pain	None 0	Mild 1	Moderate 2	Severe 3	
Erythema	None 0	Mild (barely perceptible) 1	Moderate (well defined) 2	Severe (very red) 3	
Edema	None 0	Mild (barely perceptible) 1	Moderate (well defined) 2	Severe (raised >2mm) 3	Exfoliative dermatitis 4
Vertigo	None 0	Mild 1	Moderate 2	Severe 3	
Tinnitus	None 0	Mild 1	Moderate 2	Severe 3	
Diminished hearing	None 0	Mild 1	Moderate 2	Severe 3	Profound 4

Note: For **Erythema & Edema**, a Grade 3 reaction requires immediate notification to medical monitor. Grade 4 reactions require immediate termination of treatment and an ADVERSE EVENT NOTIFICATION. For **variables not explained directly in the form**, please refer to the PROTOCOL **10.4.** for Grade clarification, requirements for immediate notification to medical monitor, and ADVERSE EVENT NOTIFICATION.

DRUG ADMINISTRATION

	Date	Time: hh:mm	Time: hh:mm	Drug Vial No.
DAY 16	[ ][ ][ ][ ][ ][ ] D D M M Y Y			

Was the drug administration card reviewed? Yes [ ] No [ ]

Investigator's Signature: ______________________ [ ][ ][ ][ ][ ][ ] D D M M Y Y

Please make an appointment for the volunteer to come back tomorrow

Day 17

WR279396	Subject's Initials	Subject's Study Number
Log No.		

Visit Date _ _ _ _ _ _ (D D M M Y Y)

	Yes	No
Has the volunteer felt unwell since his/her last visit? If yes, comment on page 61.	☐	☐
Has the volunteer used any medication apart from the study medication since the last visit? If yes, please record details in the Concomitant Medication section.	☐	☐
Has the volunteer had side effects? In either case, please record the toxicity grades below.	☐	☐

	LOCAL TOXICITY			SYSTEMIC TOXICITY			
	Pain Grade	Erythema Grade	Edema Grade	Vertigo Grade	Tinnitus Grade	Hearing Grade	Initials
Immediate Toxicity Time: hh:mm							
Delayed Toxicity Time: hh:mm							

Pain	None 0	Mild 1	Moderate 2	Severe 3	
Erythema	None 0	Mild (barely perceptible) 1	Moderate (well defined) 2	Severe (very red) 3	
Edema	None 0	Mild (barely perceptible) 1	Moderate (well defined) 2	Severe (raised >2mm) 3	Exfoliative dermatitis 4
Vertigo	None 0	Mild 1	Moderate 2	Severe 3	
Tinnitus	None 0	Mild 1	Moderate 2	Severe 3	
Diminished hearing	None 0	Mild 1	Moderate 2	Severe 3	Profound 4

Note: For **Erythema & Edema**, a Grade 3 reaction requires immediate notification to medical monitor. Grade 4 reactions require immediate termination of treatment and an ADVERSE EVENT NOTIFICATION. For **variables not explained directly in the form**, please refer to the PROTOCOL **10.4.** for Grade clarification, requirements for immediate notification to medical monitor, and ADVERSE EVENT NOTIFICATION.

DRUG ADMINISTRATION

	Date	Time: hh:mm	Time: hh:mm	Drug Vial No.
DAY 17	_ _ _ _ _ _ (D D M M Y Y)			

Was the drug administration card reviewed? Yes ☐ No ☐

Investigator's Signature: ____________________ _ _ _ _ _ _ (D D M M Y Y)

Please make an appointment for the volunteer to come back tomorrow.

Day 18

WR279396	Subject's Initials	Subject's Study Number
Log No.		

Visit Date _ _ _ _ _ _ (D D M M Y Y)

	Yes	No
Has the volunteer felt unwell since his/her last visit? If yes, comment on page 61.	☐	☐
Has the volunteer used any medication apart from the study medication since the last visit? If yes, please record details in the Concomitant Medication section.	☐	☐
Has the volunteer had side effects? In either case, please record the toxicity grades below.	☐	☐

	LOCAL TOXICITY			SYSTEMIC TOXICITY			
Immediate Toxicity Time: hh:mm	Pain Grade	Erythema Grade	Edema Grade	Vertigo Grade	Tinnitus Grade	Hearing Grade	Initials
Delayed Toxicity Time: hh:mm	Pain Grade	Erythema Grade	Edema Grade	Vertigo Grade	Tinnitus Grade	Hearing Grade	Initials

Pain	None 0	Mild 1	Moderate 2	Severe 3	
Erythema	None 0	Mild (barely perceptible) 1	Moderate (well defined) 2	Severe (very red) 3	
Edema	None 0	Mild (barely perceptible) 1	Moderate (well defined) 2	Severe (raised >2mm) 3	Exfoliative dermatitis 4
Vertigo	None 0	Mild 1	Moderate 2	Severe 3	
Tinnitus	None 0	Mild 1	Moderate 2	Severe 3	
Diminished hearing	None 0	Mild 1	Moderate 2	Severe 3	Profound 4

Note: For **Erythema & Edema**, a Grade 3 reaction requires immediate notification to medical monitor. Grade 4 reactions require immediate termination of treatment and an ADVERSE EVENT NOTIFICATION. For **variables not explained directly in the form**, please refer to the PROTOCOL **10.4.** for Grade clarification, requirements for immediate notification to medical monitor, and ADVERSE EVENT NOTIFICATION.

DRUG ADMINISTRATION

	Date	Time: hh:mm	Time: hh:mm	Drug Vial No.
DAY 18	_ _ _ _ _ _ (D D M M Y Y)			

Was the drug administration card reviewed? Yes ☐ No ☐

Investigator's Signature: ____________________ _ _ _ _ _ _ (D D M M Y Y)

Please make an appointment for the volunteer to come back tomorrow

Day 19

WR279396	Subject's Initials	Subject's Study Number
Log No.		

Visit Date |_|_|_|_|_|_|
D D M M Y Y

	Yes	No
Has the volunteer felt unwell since his/her last visit? If yes, comment on page 61.	☐	☐
Has the volunteer used any medication apart from the study medication since the last visit? If yes, please record details in the Concomitant Medication section.	☐	☐
Has the volunteer had side effects? In either case, please record the toxicity grades below.	☐	☐

	LOCAL TOXICITY			SYSTEMIC TOXICITY			
Immediate Toxicity Time: hh:mm	Pain Grade	Erythema Grade	Edema Grade	Vertigo Grade	Tinnitus Grade	Hearing Grade	Initials
	☐	☐	☐	☐	☐	☐	____
Delayed Toxicity Time: hh:mm	Pain Grade	Erythema Grade	Edema Grade	Vertigo Grade	Tinnitus Grade	Hearing Grade	Initials
	☐	☐	☐	☐	☐	☐	____

Pain	None 0	Mild 1	Moderate 2	Severe 3	
Erythema	None 0	Mild (barely perceptible) 1	Moderate (well defined) 2	Severe (very red) 3	
Edema	None 0	Mild (barely perceptible) 1	Moderate (well defined) 2	Severe (raised >2mm) 3	Exfoliative dermatitis 4
Vertigo	None 0	Mild 1	Moderate 2	Severe 3	
Tinnitus	None 0	Mild 1	Moderate 2	Severe 3	
Diminished hearing	None 0	Mild 1	Moderate 2	Severe 3	Profound 4

Note: For **Erythema & Edema**, a Grade 3 reaction requires immediate notification to medical monitor. Grade 4 reactions require immediate termination of treatment and an ADVERSE EVENT NOTIFICATION. For **variables not explained directly in the form**, please refer to the PROTOCOL **10.4.** for Grade clarification, requirements for immediate notification to medical monitor, and ADVERSE EVENT NOTIFICATION.

DRUG ADMINISTRATION

	Date	Time: hh:mm	Time: hh:mm	Drug Vial No.
DAY 19	|_|_|_|_|_|_| D D M M Y Y			

Was the drug administration card reviewed? Yes ☐ No ☐

Investigator's Signature: ____________________ |_|_|_|_|_|_|
D D M M Y Y

Please make an appointment for the volunteer to come back tomorrow.

Day 20

WR279396	Subject's Initials	Subject's Study Number
Log No.		

Visit Date |_|_|_|_|_|_| D D M M Y Y

	Yes	No
Has the volunteer felt unwell since his/her last visit? If yes, comment on page 61.	☐	☐
Has the volunteer used any medication apart from the study medication since the last visit? If yes, please record details in the concomitant medication section.	☐	☐
Has the volunteer had side effects? In either case, please record the toxicity grades below.	☐	☐

	LOCAL TOXICITY			SYSTEMIC TOXICITY			
	Pain Grade	Erythema Grade	Edema Grade	Vertigo Grade	Tinnitus Grade	Hearing Grade	Initials
Immediate Toxicity Time: hh:mm	☐	☐	☐	☐	☐	☐	____
Delayed Toxicity Time: hh:mm	☐	☐	☐	☐	☐	☐	____

Pain	None	0	Mild	1	Moderate	2	Severe	3		
Erythema	None	0	Mild (barely perceptible)	1	Moderate (well defined)	2	Severe (very red)	3		
Edema	None	0	Mild (barely perceptible)	1	Moderate (well defined)	2	Severe (raised >2mm)	3	Exfoliative dermatitis	4
Vertigo	None	0	Mild	1	Moderate	2	Severe	3		
Tinnitus	None	0	Mild	1	Moderate	2	Severe	3		
Diminished hearing	None	0	Mild	1	Moderate	2	Severe	3	Profound	4

Note: For **Erythema & Edema**, a Grade 3 reaction requires immediate notification to medical monitor. Grade 4 reactions require immediate termination of treatment and an ADVERSE EVENT NOTIFICATION. For **variables not explained directly in the form**, please refer to the PROTOCOL **10.4.** for Grade clarification, requirements for immediate notification to medical monitor, and ADVERSE EVENT NOTIFICATION.

Was the hearing eighth nerve test: high & low tones conducted ?
If not, ! DO IT!

Initial When Completed ☐

HEARING TEST

	Normal	Abnormal
Hearing Test - High Tone	☐	☐
Hearing Test - Low Tone	☐	☐

	250/256	500/512	1,000/1,024		3,000/2,896
Right					
Left					

	4,000/4,096	6,000/6,144	8,000/8,192
Right			
Left			

WR279396	Subject's Initials	Subject's Study Number
Log No.		

ROMBERG TEST

Was the Romberg test conducted? If not, DO IT! — Initial When Completed ☐

Romberg Test ☐ ☐ Comments: ______________________

Signature (Individual Conducting the Test): ______________________

Print Name (Last, First, MI): ______________________

Has a blood sample been taken for biochemical analysis (creatinine)? Red top (10 ml) and process as per SOP. If yes, please record the results in the Hematology and Biochemistry report form.
If not, DO IT ! — Initial When Completed ☐

Has a urine sample been collected for urinalysis ? 50 ml of urine. If yes, have the results been recorded in the Hematology, Biochemistry & Urinalysis Patient Data report form. If not, DO IT ! — Initial When Completed ☐

Has a blood sample been taken for pharmacokinetic analysis ?
[just prior to the next application of drug ("trough") and one hour after drug application ("peak")]. Use a Purple Top collection tube (5ml with EDTA). Has the n=3 0.5ml samples been deposited in the freezer, and the samples logged in the Pharmokinetics Sample Log List report form. If not, DO IT ! — Initial When Completed ☐

Has a 24 hour urine collection been taken to determine total absorved drugs by taking 50-100 ml clean catch and transfering n=4 1ml samples into cryotubes ? Have the n=4 1ml samples been deposited in the freezer, and the samples logged in the Pharmokinetics Samples Log List report form ? If not, DO IT ! — Initial When Completed ☐

Has the lesion(s) (ulcers and lymph nodes) measurement been taken? If yes, have the results been recorded in the Lesion(s) - Clinical Findings report form. If not, DO IT ! — Initial When Completed ☐

Has the lesion(s) been clinicaly evaluated? If yes, please record the results in the Lesion(s) - Clinical Findings report form. If not, Do IT ! — Initial When Completed ☐

Has a photograph of leishmaniansis lesion(s) been taken ? If yes, has the results been recorded in the WR279396 Photograph Lesion Patient Data report form. If not, DO IT ! — Initial When Completed ☐

Has a lesion(s) tissue sample been taken for amastigote detection (visuilization) from an unhealed designated test lesion ? If yes, please record the results in the Parasitology Laboratory Patient Data report form. If not, DO IT ! — Initial When Completed ☐

DRUG ADMINISTRATION

	Date	Time: hh:mm	Time: hh:mm	Drug Vial No.
DAY 20	D D M M Y Y			

Was the drug administration card reviewed ? Yes ☐ No ☐

Investigator's Signature: ______________________ D D M M Y Y

Please make an appointment for the volunteer to come back for the 1.0 month appointment in 30 days

WR279396	Subject's Initials	Subject's Study Number
Log No.		

PHYSICAL EXAMINATION

HEARING TEST AND ROMBERG TEST

	Normal	Abnormal
Hearing Test - High Tone (250 Hz -3,000 Hz)	☐	☐
Hearing Test - Low Tone (4,000 Hz - 8,000 Hz)	☐	☐
Romberg Test	☐	☐

	250/256	500/512	1,000/1,024	2,000/2,048	3,000/2,896
Right					
Left					

	4,000/4,096	6,000/6,144	8,000/8,192
Right			
Left			

Comments: ______________________

Signature (Individual Conducting the Test): ______________________

Print Name (Last, First, MI) : ______________________

Signature (Medical Officer/ Investigator): ______________________

Print Name (Last, First, MI): ______________________

D D M M Y Y

Day 20 (continued)

WR279396	Subject's Initials	Subject's Study Number
Log No.		

LESION(s) - CLINICAL FINDINGS REPORT FORM

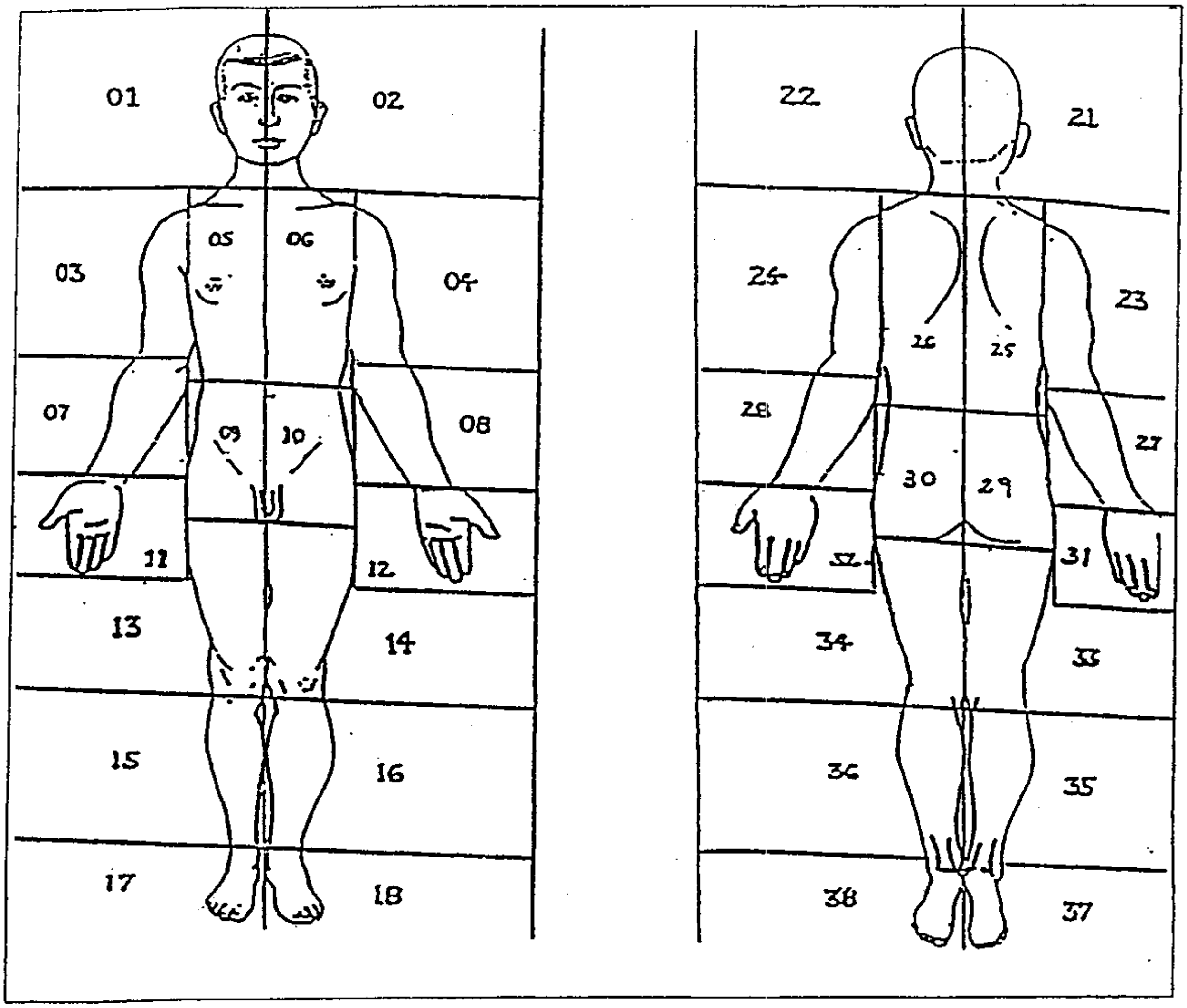

Location of lesion(s): Mark with an **X** the lesion(s) location & number the lesion on the fig. above (e.g. X1)

Lesion Number 1	Site according to the figure ________
Designate type & size of lesion:	
Ulceration	____________ X __________ mm.
Induration	____________ X __________ mm.
Papule	____________ X __________ mm.
Papulo-Squamos (scaly) Lesion	__________ X _________ mm.
Lymph node ________ mm.	

Day 20 (continued)

WR279396	Subject's Initials	Subject's Study Number
Log No.		

LESION(s) - CLINICAL FINDINGS REPORT FORM (Continued)

Lesion Number 2	Site according to the figure ________
Designate type & size of lesion:	
Ulceration	________ X ________ mm.
Induration	________ X ________ mm.
Papule	________ X ________ mm.
Papulo-Squamos (scaly) Lesion	________ X ________ mm.
Lymph node ________ mm.	

Lesion Number 3	Site according to the figure ________
Designate type & size of lesion:	
Ulceration	________ X ________ mm.
Induration	________ X ________ mm.
Papule	________ X ________ mm.
Papulo-Squamos (scaly) Lesion	________ X ________ mm.

Lesion Number 4	Site according to the figure ________
Designate type & size of lesion:	
Ulceration	________ X ________ mm.
Induration	________ X ________ mm.
Papule	________ X ________ mm.
Papulo-Squamos (scaly) Lesion	________ X ________ mm.
Lymph node ________ mm.	

Lesion Number 5	Site according to the figure ________
Designate type & size of lesion:	
Ulceration	________ X ________ mm.
Induration	________ X ________ mm.
Papule	________ X ________ mm.
Papulo-Squamos (scaly) Lesion	________ X ________ mm.
Lymph node ________ mm.	

Day 20 (continued)

WR279396	Subject's Initials	Subject's Study Number
Log No.		

PARASITOLOGY LABORATORY
PATIENT DATA REPORT

Specimen(s) obtained: Yes: ______ No: ______

Date __ __ __ __ __ __ (D D M M Y Y) Time __ __ : __ __ (H H : M M)

Specimen(s) obtained by: ______________________________
Last name, First Name MI

Specimen(s) logged into (lab): Time __ __ : __ __ (H H M M) Lab ID Number: ______________

Source of specimen: (mark one only)

Lesion: ☐ Lymph Node: ☐ Other: ☐

Lesion Number: ______________ Lesion Site from Figure: ______________

Parasitology Laboratory Assessment:

Culture:

		Date Positive	Species Identified	Initials
SCH 1	D D M M Y Y	D D M M Y Y	______________	______
SCH 2	D D M M Y Y	D D M M Y Y	______________	______
SCH 3	D D M M Y Y	D D M M Y Y	______________	______

	Neg	1+	2+	3+	4+	5+	Initials	
DQ smear	☐	☐	☐	☐	☐	☐	______	D D M M Y Y

Comments: ____

____

____

Signature (Investigator/Subinvestigator): ______________ Date: __ __ __ __ __ __ (D D M M Y Y)

Note: If the species is *L. aethiopica* the volunteer will be referred to his primary health care physician as per protocol. Physician Signature: ______________________

WR279396	Subject's Initials	Subject's Study Number
Log No.		

PARASITOLOGY LABORATORY
PATIENT DATA REPORT

Specimen(s) obtained: Yes: ______ No: ______

Date |_|_|_|_|_|_| (D D M M Y Y) Time |_|_| : |_|_| (H H : M M)

Specimen(s) obtained by: ____________________
Last name, First Name MI

Specimen(s) logged into (lab): Time |_|_| : |_|_| (H H M M) Lab ID Number: ____________

Source of specimen: (mark one only)

Lesion: [ ] Lymph Node: [ ] Other: [ ]

Lesion Number: ____________ Lesion Site from Figure: ____________

Parasitology Laboratory Assessment:

Culture:

		Date Positive	Species Identified	Initials
SCH 1	D D M M Y Y	D D M M Y Y		
SCH 2	D D M M Y Y	D D M M Y Y		
SCH 3	D D M M Y Y	D D M M Y Y		

	Neg	1+	2+	3+	4+	5+	Initials	
DQ smear	[ ]	[ ]	[ ]	[ ]	[ ]	[ ]	______	D D M M Y Y

Comments: ____________________

____

____

Signature (Investigator/Subinvestigator): ____________ Date: |_|_|_|_|_|_| (D D M M Y Y)

Note: If the species is *L. aethiopica* the volunteer will be referred to his primary health care physician as per protocol. Physician Signature: ____________________

WR279396	Subject's Initials	Subject's Study Number
Log No.		

PARASITOLOGY LABORATORY
PATIENT DATA REPORT

Specimen(s) obtained: Yes: ______ No: ______

Date D D M M Y Y Time H H : M M

Specimen(s) obtained by: ______________________
Last name, First Name MI

Specimen(s) logged into (lab): Time H H : M M Lab ID Number: ______________

Source of specimen: (mark one only)

Lesion: ☐ Lymph Node: ☐ Other: ☐

Lesion Number: ______________ Lesion Site from Figure: ______________

Parasitology Laboratory Assessment:

Culture:

		Date Positive	Species Identified	Initials
SCH 1	D D M M Y Y	D D M M Y Y		
SCH 2	D D M M Y Y	D D M M Y Y		
SCH 3	D D M M Y Y	D D M M Y Y		

	Neg	1+	2+	3+	4+	5+	Initials	
DQ smear	☐	☐	☐	☐	☐	☐	______	D D M M Y Y

Comments: ______________________

Signature (Investigator/Subinvestigator): ______________ Date: D D M M Y Y

Note: If the species is *L. aethiopica* the volunteer will be referred to his primary health care physician as per protocol. Physician Signature: ______________

WR279396	Subject's Initials	Subject's Study Number
Log No.		

PARASITOLOGY LABORATORY
PATIENT DATA REPORT

Specimen(s) obtained: Yes: ______ No: ______

Date __ __ __ __ __ __ (D D M M Y Y) Time __ __ : __ __ (H H : M M)

Specimen(s) obtained by: ______________________________
Last name, First Name MI

Specimen(s) logged into (lab): Time __ __ : __ __ (H H M M) Lab ID Number: ______________

Source of specimen: (mark one only)

Lesion: ☐ Lymph Node: ☐ Other: ☐

Lesion Number: ______________ Lesion Site from Figure: ______________

Parasitology Laboratory Assessment:

Culture:

		Date Positive	Species Identified	Initials
SCH 1	D D M M Y Y	D D M M Y Y		
SCH 2	D D M M Y Y	D D M M Y Y		
SCH 3	D D M M Y Y	D D M M Y Y		

	Neg	1+	2+	3+	4+	5+	Initials	
DQ smear	☐	☐	☐	☐	☐	☐	______	D D M M Y Y

Comments: ______________________________________

Signature (Investigator/Subinvestigator): ______________ Date: __ __ __ __ __ __ (D D M M Y Y)

Note: If the species is *L. aethiopica* the volunteer will be referred to his primary health care physician as per protocol. Physician Signature: ______________________

WR279396	Subject's Initials	Subject's Study Number
Log No.		

PARASITOLOGY LABORATORY
PATIENT DATA REPORT

Specimen(s) obtained: Yes: ______ No: ______

Date [ ][ ][ ][ ][ ][ ] D D M M Y Y Time [ ][ ] : [ ][ ] H H : M M

Specimen(s) obtained by: ______________________
Last name, First Name MI

Specimen(s) logged into (lab): Time [ ][ ] : [ ][ ] H H M M Lab ID Number: ____________

Source of specimen: (mark one only)

Lesion: [ ] Lymph Node: [ ] Other: [ ]

Lesion Number: ____________ Lesion Site from Figure: ____________

Parasitology Laboratory Assessment:

Culture:

		Date Positive	Species Identified	Initials
SCH 1	D D M M Y Y	D D M M Y Y	____________	______
SCH 2	D D M M Y Y	D D M M Y Y	____________	______
SCH 3	D D M M Y Y	D D M M Y Y	____________	______

	Neg	1+	2+	3+	4+	5+	Initials	
DQ smear	[ ]	[ ]	[ ]	[ ]	[ ]	[ ]	______	D D M M Y Y

Comments: ______________________

____

____

Signature (Investigator/Subinvestigator): ______________ Date: [ ][ ][ ][ ][ ][ ] D D M M Y Y

Note: If the species is *L. aethiopica* the volunteer will be referred to his primary health care physician as per protocol. Physician Signature: ______________

WR279396	Subject's Initials	Subject's Study Number
Log No.		

PARASITOLOGY LABORATORY
PATIENT DATA REPORT

Specimen(s) obtained: Yes: ______ No: ______

Date |_|_|_|_|_|_| (D D M M Y Y) Time |_|_| : |_|_| (H H : M M)

Specimen(s) obtained by: ______________________
Last name, First Name MI

Specimen(s) logged into (lab): Time |_|_| : |_|_| (H H M M) Lab ID Number: ____________

Source of specimen: (mark one only)

Lesion: ☐ Lymph Node: ☐ Other: ☐

Lesion Number: ____________ Lesion Site from Figure: ________

Parasitology Laboratory Assessment:

Culture:

		Date Positive	Species Identified	Initials
SCH 1	D D M M Y Y	D D M M Y Y	________	____
SCH 2	D D M M Y Y	D D M M Y Y	________	____
SCH 3	D D M M Y Y	D D M M Y Y	________	____

	Neg	1+	2+	3+	4+	5+	Initials	
DQ smear	☐	☐	☐	☐	☐	☐	____	D D M M Y Y

Comments: ______________________________________

____

____

Signature (Investigator/Subinvestigator): ______________ Date: |_|_|_|_|_|_| (D D M M Y Y)

Note: If the species is *L. aethiopica* the volunteer will be referred to his primary health care physician as per protocol. Physician Signature: ______________________

30 day - First Follow-up

WR279396	Subject's Initials	Subject's Study Number
Log No.		

Visit Date |_|_|_|_|_|_|
D D M M Y Y

Has the volunteer felt unwell since his/her last visit? If yes, comment on page 61. Yes ☐ No ☐

Has the volunteer used any medication apart from the study medication since the last visit? If yes, please record details in the Concomitant Medication section. Yes ☐ No ☐

Has the lesion(s) (ulcers and lymph nodes) measurement been taken? If yes, please record the results in the Lesion(s) - Clinical Findings report form. If not, DO IT ! Yes ☐ No ☐

Has the lesion(s) been clinicaly evaluated? If yes, please record the results in the Lesion(s) - Clinical Findings report form. If not, DO IT ! Yes ☐ No ☐

Has a photograph of the leishmaniansis lesion been taken ? Has the photo been attached to the WR279396 Photograph Lesion Patient Data report form. If not, DO IT ! Yes ☐ No ☐

Please make an appointment for the volunteer to come back for the 80 days appointment in 40 days.

Date of return: |_|_|_|_|_|_|
D D M M Y Y

80 day - Second Follow-up

WR279396	Subject's Initials	Subject's Study Number
Log No.		

Visit Date |_|_|_|_|_|_|
D D M M Y Y

Has the volunteer felt unwell since his/her last visit? If yes , comment on page 61.	Yes	☐	No	☐
Has the volunteer used any medication apart from the study medication since the last visit? If yes, please record details in the Concomitant Medication section.	Yes	☐	No	☐
Has the lesion(s) (ulcers and lymph nodes) measurement been taken? If yes, please record the results in the Lesion(s) - Clinical Findings report form. If not, DO IT !	Yes	☐	No	☐
Has the lesion(s) been clinicaly evaluated? If yes, please record the results in the Lesion(s) - Clinical Findings report form. If not, DO IT !	Yes	☐	No	☐
Has a photograph of the leishmaniansis lesion been taken ? Has the photo been attached to the WR279396 Photograph Lesion Patient Data report form. If not, DO IT !	Yes	☐	No	☐

Please make an appointment for the volunteer to come back for the 6 month appointment in 100 days.

Date of return: |_|_|_|_|_|_|
D D M M Y Y

6 months- Third Follow-up

WR279396	Subject's Initials	Subject's Study Number
Log No.		

Visit Date |_|_|_|_|_|_|
D D M M Y Y

Has the volunteer felt unwell since his/her last visit? If yes , comment on page 61.	Yes	☐	No	☐
Has the volunteer used any medication apart from the study medication since the last visit? If yes, please record details in the Concomitant Medication section.	Yes	☐	No	☐
Has the lesion(s) (ulcers and lymph nodes) measurement been taken? If yes, please record the results in the Lesion(s) - Clinical Findings report form. If not, DO IT !	Yes	☐	No	☐
Has the lesion(s) been clinicaly evaluated? If yes, please record the results in the Lesion(s) - Clinical Findings report form. If not, DO IT !	Yes	☐	No	☐
Has a photograph of the leishmaniansis lesion been taken ? Has the photo been attached to the WR279396 Photograph Lesion Patient Data report form. If not, DO IT !	Yes	☐	No	☐

Study Period: All

WR279396	Subject's Initials	Subject's Study Number
Log No.		

HEMATOLOGY, BIOCHEMISTRY AND URINALYSIS
PATIENT DATA REPORT FORM

TEST	Day -1 (Screening)	Day 10	Day 20
Date: (DD/MM/YY)	| | | | |	| | | | |	| | | | |
Haematocrit: (L/L)		NA	NA
Haemoglobin (g/dL)		NA	NA
RBC (x 10^6/L)		NA	NA
WBC (x 10^3/L)		NA	NA
Granulocytes [N, E & B] (%)		NA	NA
Lymphocytes (%)		NA	NA
Monocytes (%)		NA	NA
Platelets (x 10^3/L)		NA	NA
ALT (U/L) (SGPT)		NA	NA
AST (U/L) (SGOT)		NA	NA
Creatinine serum (mg/L)			
BUN (mg/L)		NA	NA
Total Protein (gm/L)		NA	
Na mEq/L		NA	
K mEq/L		NA	
Glucose gm/L		NA	

Please comment on abnormal results, and state whether clinically significant or not.

VISIT	TEST	COMMENT

Note: Please attach original laboratory print-outs to the back of this page.

Study Period: All

WR279396	Subject's Initials	Subject's Study Number
Log No.		

	PHARMACOKINETICS SAMPLES<br>LOG LIST REPORT FORM
SCREENING<br>Day -1	Blood Sample plasma "Baseline" levels<br>Date sample taken [D D M M Y Y] Time [H H] : [M M]<br>Location: Box ________ Space ________ Temperature ______
SCREENING<br>Day -1	Base-line urine sample<br>Date sample taken [D D M M Y Y] Time [H H] : [M M]<br>Location: Box ________ Space ________ Temperature ______
DAY 10	Blood Sample plasma "Trough " levels<br>Date sample taken [D D M M Y Y] Time [H H] : [M M]<br>Location: Box ________ Space ________ Temperature ______
DAY 10	Blood Sample plasma "Peak" levels<br>Date sample taken [D D M M Y Y] Time [H H] : [M M]<br>Location: Box ________ Space ________ Temperature ______
DAY 10	Twenty-four hour urine sample<br>Date sample taken [D D M M Y Y] Time [H H] : [M M]<br>Location: Box ________ Space ________ Temperature ______
DAY 20	Blood Sample plasma "Trough " levels<br>Date sample taken [D D M M Y Y] Time [H H] : [M M]<br>Location: Box ________ Space ________ Temperature ______
DAY 20	Blood Sample plasma "Peak" levels<br>Date sample taken [D D M M Y Y] Time [H H] : [M M]<br>Location: Box ________ Space ________ Temperature ______
DAY 20	Twenty-four hour urine sample<br>Date sample taken [D D M M Y Y] Time [H H] : [M M]<br>Location: Box ________ Space ________ Temperature ______

Study Period: All

WR279396	Subject's Initials	Subject's Study Number
Log No.		

WR 279396 PHOTOGRAPH LESION PATIENT DATA REPORT

Day -1 ________ **Lesion Number:**

Day 20 ________ **Lesion Number:**

WR279396	Subject's Initials	Subject's Study Number
Log No.		

WR 279396 PHOTOGRAPH LESION PATIENT DATA REPORT (Continued)

30 Days **Lesion Number:** ________

80 Days **Lesion Number:** ________

Study Period: All

WR279396	Subject's Initials	Subject's Study Number
Log No.		

WR 279396 PHOTOGRAPH LESION PATIENT DATA REPORT (Continued)

6 Months	Lesion Number:

Study Period: All

WR279396	Subject's Initials	Subject's Study Number
Log No.		

CONCOMITANT MEDICATION

Medication	Dose	Frequency	Start Date dd/mm/yy	Stop Date dd/mm/yy	Indication

Note: For each entry, please write your initials in the space right of the INDICATION BOX so QA knows who recorded the data.

Study Period: All

WR279396	Subject's Initials	Subject's Study Number
Log No.		

CONCOMITANT MEDICATION

Medication	Dose	Frequency	Start Date dd/mm/yy	Stop Date dd/mm/yy	Indication

Note: For each entry, please write your initials in the space right of the INDICATION BOX so QA knows who recorded the data.

Study Period: All

WR279396	Subject's Initials	Subject's Study Number
Log No.		

ADVERSE EXPERIENCE

Describe the adverse event (unusual illness that has appeared / worsened since drug application). Complete this form whether or not you believe the event was related to the drug. Record desecrate events individually, two per form, signs and symptoms known to be associated should be grouped together as a syndrome.

Description	1	2
Onset Date (dd/mm/yy)	__/__/__	__/__/__
Nature of Event	☐ Constant<br>☐ Single Episode<br>☐ Multiple Episode	☐ Constant<br>☐ Single Episode<br>☐ Multiple Episode
Stop Date (dd/mm/yy)	__/__/__	__/__/__
Was Event Serious or Life Threatening?	☐ Yes*<br>☐ No	☐ Yes*<br>☐ No
Intensity	☐ Mild (noticeable, but can be easily be ignored<br>☐ Moderate (discomfort, but does not interfere with activities of daily living)<br>☐ Severe (interferes with activities of daily living)	☐ Mild (noticeable, but can be easily be ignored<br>☐ Moderate (discomfort, but does not interfere with activities of daily living)<br>☐ Severe (interferes with activities of daily living)
Action Taken	☐ None<br>☐ Administered Symptomatic Therapy<br>☐ Hospitalization*<br>☐ Trial Drug discontinued/interrupted	☐ None<br>☐ Administered Symptomatic Therapy<br>☐ Hospitalization*<br>☐ Trial Drug discontinued/interrupted
Resolution	☐ Event completely resolved<br>☐ Event has not resolved to date<br>☐ Event has resolved, but residual effects persist<br>☐ Death*	☐ Event completely resolved<br>☐ Event has not resolved to date<br>☐ Event has resolved, but residual effects persist.<br>☐ Death*
Relationship to Study Drug	☐ Not Related<br>☐ Possibly<br>☐ Probably<br>☐ Definitely	☐ Not Related<br>☐ Possibly<br>☐ Probably<br>☐ Definitely
Was Event Unexpected	☐ Yes*<br>☐ No	☐ Yes*<br>☐ No
Did the volunteer receive any concomitant medication. If yes , please complete the **CONCOMITANT MEDICATION** section.	☐ Yes<br>☐ No	☐ Yes<br>☐ No
Investigator's Comments		

By my signature below, I attest that the information contained herein is accurate and complete.

Investigator's Signature ______________________________ | | | | | | | d d m m y y

*For a Serious Adverse Experience, **Appendix 3 - IND Safety Data** must be completed and **Adverse event** [illegible]

WR279396	Subject's Initials	Subject's Study Number
Log No.		

ADVERSE EXPERIENCE

Describe the adverse event (unusual illness that has appeared / worsened since drug application). Complete this form whether or not you believe the event was related to the drug. Record desecrate events individually, two per form, signs and symptoms known to be associated should be grouped together as a syndrome.

Description	1	2
Onset Date (dd/mm/yy)		
Nature of Event	☐ Constant<br>☐ Single Episode<br>☐ Multiple Episode	☐ Constant<br>☐ Single Episode<br>☐ Multiple Episode
Stop Date (dd/mm/yy)		
Was Event Serious or Life Threatening?	☐ Yes*<br>☐ No	☐ Yes*<br>☐ No
Intensity	☐ **Mild** (noticeable, but can be easily be ignored<br>☐ **Moderate** (discomfort, but does not interfere with activities of daily living)<br>☐ **Severe** (interferes with activities of daily living)	☐ **Mild** (noticeable, but can be easily be ignored<br>☐ **Moderate** (discomfort, but does not interfere with activities of daily living)<br>☐ **Severe** (interferes with activities of daily living)
Action Taken	☐ None<br>☐ Administered Symptomatic Therapy<br>☐ Hospitalization*<br>☐ Trial Drug discontinued/interrupted	☐ None<br>☐ Administered Symptomatic Therapy<br>☐ Hospitalization*<br>☐ Trial Drug discontinued/interrupted
Resolution	☐ Event completely resolved<br>☐ Event has not resolved to date<br>☐ Event has resolved, but residual effects persist<br>☐ Death*	☐ Event completely resolved<br>☐ Event has not resolved to date<br>☐ Event has resolved, but residual effects persist.<br>☐ Death*
Relationship to Study Drug	☐ Not Related<br>☐ Possibly<br>☐ Probably<br>☐ Definitely	☐ Not Related<br>☐ Possibly<br>☐ Probably<br>☐ Definitely
Was Event Unexpected	☐ Yes*<br>☐ No	☐ Yes*<br>☐ No
Did the volunteer receive any concomitant medication. If yes, please complete the **CONCOMITANT MEDICATION** section.	☐ Yes<br>☐ No	☐ Yes<br>☐ No
Investigator's Comments		

By my signature below, I attest that the information contained herein is accurate and complete.

Investigator's Signature ______________________________ |_|_|_|_|_|_| d d m m y y

*For a Serious Adverse Experience, **Appendix 3 - IND Safety Data** must be completed and **Adverse event** [illegible]

Study Period: All

WR279396	Subject's Initials	Subject's Study Number
Log No.		

ADVERSE EXPERIENCE

Describe the adverse event (unusual illness that has appeared / worsened since drug application). Complete this form whether or not you believe the event was related to the drug. Record desecrate events individually, two per form, signs and symptoms known to be associated should be grouped together as a syndrome.

Description	1	2
Onset Date (dd/mm/yy)	| | | | | |	| | | | | |
Nature of Event	☐ Constant<br>☐ Single Episode<br>☐ Multiple Episode	☐ Constant<br>☐ Single Episode<br>☐ Multiple Episode
Stop Date (dd/mm/yy)	| | | | | |	| | | | | |
Was Event Serious or Life Threatening?	☐ Yes*<br>☐ No	☐ Yes*<br>☐ No
Intensity	☐ Mild (noticeable, but can be easily be ignored<br>☐ Moderate (discomfort, but does not interfere with activities of daily living)<br>☐ Severe (interferes with activities of daily living)	☐ Mild (noticeable, but can be easily be ignored<br>☐ Moderate (discomfort, but does not interfere with activities of daily living)<br>☐ Severe (interferes with activities of daily living)
Action Taken	☐ None<br>☐ Administered Symptomatic Therapy<br>☐ Hospitalization*<br>☐ Trial Drug discontinued/interrupted	☐ None<br>☐ Administered Symptomatic Therapy<br>☐ Hospitalization*<br>☐ Trial Drug discontinued/interrupted
Resolution	☐ Event completely resolved<br>☐ Event has not resolved to date<br>☐ Event has resolved, but residual effects persist<br>☐ Death*	☐ Event completely resolved<br>☐ Event has not resolved to date<br>☐ Event has resolved, but residual effects persist.<br>☐ Death*
Relationship to Study Drug	☐ Not Related<br>☐ Possibly<br>☐ Probably<br>☐ Definitely	☐ Not Related<br>☐ Possibly<br>☐ Probably<br>☐ Definitely
Was Event Unexpected	☐ Yes*<br>☐ No	☐ Yes*<br>☐ No
Did the volunteer receive any concomitant medication. If yes, please complete the **CONCOMITANT MEDICATION** section.	☐ Yes<br>☐ No	☐ Yes<br>☐ No
Investigator's Comments		

By my signature below, I attest that the information contained herein is accurate and complete.

Investigator's Signature ______________________ \| \| \| \| \| \| \|
d d m m y y

*For a Serious Adverse Experience, **Appendix 3 - IND Safety Data** must be completed and **Adverse event** [illegible]

WR279396	Subject's Initials	Subject's Study Number
Log No.		

ADVERSE EXPERIENCE

Describe the adverse event (unusual illness that has appeared / worsened since drug application). Complete this form whether or not you believe the event was related to the drug. Record desecrate events individually, two per form, signs and symptoms known to be associated should be grouped together as a syndrome.

Description	1	2
Onset Date (dd/mm/yy)		
Nature of Event	☐ Constant<br>☐ Single Episode<br>☐ Multiple Episode	☐ Constant<br>☐ Single Episode<br>☐ Multiple Episode
Stop Date (dd/mm/yy)		
Was Event Serious or Life Threatening?	☐ Yes*<br>☐ No	☐ Yes*<br>☐ No
Intensity	☐ **Mild** (noticeable, but can be easily be ignored<br>☐ **Moderate** (discomfort, but does not interfere with activities of daily living)<br>☐ **Severe** (interferes with activities of daily living)	☐ **Mild** (noticeable, but can be easily be ignored<br>☐ **Moderate** (discomfort, but does not interfere with activities of daily living)<br>☐ **Severe** (interferes with activities of daily living)
Action Taken	☐ None<br>☐ Administered Symptomatic Therapy<br>☐ Hospitalization*<br>☐ Trial Drug discontinued/interrupted	☐ None<br>☐ Administered Symptomatic Therapy<br>☐ Hospitalization*<br>☐ Trial Drug discontinued/interrupted
Resolution	☐ Event completely resolved<br>☐ Event has not resolved to date<br>☐ Event has resolved, but residual effects persist<br>☐ Death*	☐ Event completely resolved<br>☐ Event has not resolved to date<br>☐ Event has resolved, but residual effects persist.<br>☐ Death*
Relationship to Study Drug	☐ Not Related<br>☐ Possibly<br>☐ Probably<br>☐ Definitely	☐ Not Related<br>☐ Possibly<br>☐ Probably<br>☐ Definitely
Was Event Unexpected	☐ Yes*<br>☐ No	☐ Yes*<br>☐ No
Did the volunteer receive any concomitant medication. If yes, please complete the **CONCOMITANT MEDICATION** section.	☐ Yes<br>☐ No	☐ Yes<br>☐ No
Investigator's Comments		

By my signature below, I attest that the information contained herein is accurate and complete.

Investigator's Signature ______________________ d d m m y y

*For a Serious Adverse Experience, **Appendix 3 - IND Safety Data** must be completed and **Adverse event** [illegible]

WR279396	Subject's Initials	Subject's Study Number
Log No.		

STUDY TERMINATION RECORD

Did the Patient Complete the Study? ☐ YES ☐ NO

If NO, Indicate Primary and Secondary reasons for discontinuation.

Reason(s) for Discontinuation

	Primary Reason (check only one)	Secondary Reason (check as many as applicable)
Adverse Reaction<br>-Specify on the ADVERSE EXPERIENCE form	☐	☐
Other Medical Event<br>-Specify on the ADVERSE EXPERIENCE form	☐	☐
Failed to Return<br>-Specify reason if known<br>____________________	☐	☐
Unsatisfactory Response - Efficacy	☐	☐
Protocol Violation<br>-Specify<br>____________________	☐	☐
Patient Died	☐	☐
Other Nonmedical Event<br>-Specify<br>____________________	☐	☐
Patient Request - other than recorded above<br>-Specify<br>____________________	☐	☐

I, ____________________________, certify that I have examined all of the pages of this case report set for this patient and found them to be complete and accurate.

|_|_|_|_|_|_|
D D M M Y Y

____

Investigator's Signature

TITLE: **TOPICAL TREATMENT OF CUTANEOUS LEISHMANIASIS WITH WR 2379396: A PHASE 2 STUDY IN THE OLD WORLD**

Appendix 5
MONITORING PLAN AND SCHEDULE OF REPORTS

VERSION 7.0
IND 50,098
LOG NO.
17 June 2002

1. **Monitoring Plan.** The study will be monitor by the Principal Investigator and personnel from the Department of Quality Assurance at USAMMDA. At least 3 on-site visits will be conducted to assess the site, progress of the trial and the accuracy of the data collected: a) Pre-investigational Site Visit/Study Initiation Visit, b) Routine Monitoring Visit - middle visit, and c) study closure visit.

2. **Report Schedule.** As per SOP a weekly, quarterly and a final report are required. The weekly report will follow the attached format. (FAX to 301-319-9180)

1. STUDY SUMMARY

# Screen	# Enrolled	Parasites* Post/Negt	Size-Pre	Size 20D	Size 30D	80D	6M

* C= Culture/ DK=DifQuik/ LSTA=Leishmania Skin Test Antigen

2. WEEK SUMMARY

Pt. No.	Volunteer/Subject No.	Exclusion Criteria

3. WEEKLY TOXICITY

Volunt. No.	Day	Pain	Erythema	Edema	Vertigo	Tinnitus	Hearing

4. WEEKLY ADMINISTRATION

Clinical Tests	Parasit. Tests	Administration	Travel	Other

5. WEEKLY COMMENTS.

PROCEDURAL TIMETABLE SUMMARY FOR INITIALS AS PER SOP NO. 6

Procedure	Therapy Period (days)				Follow-up (Days/months)#		
	-1	1	10	20	30	80	6 Months
**Informed Consent**	X						
**Demographic Details**	X						
**Medical History**	X						
**History of Leishmaniasis**	X						
**Physical Exam**	X						
**Dermatology Exam**	X						
**Hearing Test**	X		X	X			
**Romberg Test**	X		X	X			
CBC (WBC,platelets, Hgb)	X						
**AST or ALT**	X						
**Serum Creatinine**	X		X	X			
**Glucose, Na, K**	X						
**Blood pregnancy Test < 72 hours before D0**	X						
**Urinalysis**	X			X			
**Pharmacokinetics Blood**	X		X	X			
**Pharmacokinetics Urine**	X		X**	X**			
**Measure ulcer**	X			X	X	X	X
**Clinical Evaluation of Lesion**	X			X	X	X	X
**Photograph Lesion**	X			X	X	X	X
**Parasitologic Test**	X			X*			
**Entrance Exam and Lab Test Check List**	X						

**Drug Therapy**	Days 1 through 20
**Local Toxicity**	Days 1 through 20

* Note: Perform only on unhealed designated test lesion.

** A 24-hour urine collection will be performed on Days 9 to 10 and 19 to 20.

Follow-up refers to time after end of therapy

TITLE: TOPICAL TREATMENT OF CUTANEOUS LEISHMANIASIS WITH WR 279396: A PHASE 2 STUDY IN THE OLD WORLD

Appendix 6
STANDARD OPERATING PROCEDURES

Version 7.0
IND 50,098
Log No.
17 June 2002

TOPICAL TREATMENT OF CUTANEOUS LEISHMANIASIS WITH WR 279396: A PHASE 2 STUDY IN THE OLD WORLD (Log. No.)

SOP #1: SOP For Development and Review of Standard Operating Procedures (SOPs)

Date Approved: ____________________ Version #: ______

Next Review Due: ____________________

Author(s): ____

____

Reviewed by: ____

____

Purpose: The purpose of this SOP is to establish a procedure for the format, writing, approval, implementation and periodic review of Standard Operating Procedures (SOPs).

Procedure:

1. Each SOP should be written in a similar format to this example, clearly indicating title, date of approval, version #, date revision is due, author(s), purpose, and exact procedure.

2. This procedure is effective at the date of approval

3. Each SOP should be reviewed by the appropriate study personnel; this SOP should be reviewed by the study Co-Principal Investigators (Co-PIs).

4. The persons who should use the SOP should be indicated; this SOP should be utilized by any study personnel involved in authoring, reviewing or approving study SOPs.

5. The SOP should be signed and dated by the study Co-PIs.

6. An appropriate period for future review should be established for each SOP; this SOP should be reviewed annually.

C0-Principal Investigators' signatures:

__________________________ Date: __________________

Dr. Max Grogl

__________________________ Date: __________________

Dr. Pierre Buffet

__________________________ Date: __________________

Dr. Afif Ben Salah

Users: I have read & understood SOP No.1 (Signature & Date):

TOPICAL TREATMENT OF CUTANEOUS LEISHMANIASIS WITH WR 279396: A PHASE 2 STUDY IN THE OLD WORLD (Log. No.)

SOP #2: SOP For Test Article Accountability

Date Approved: ____________________ Version #: ______
Next Review Due: ____________________

Author(s): ____

____

Reviewed by: ____

____

Purpose: The purpose of this SOP is to establish a procedure for: form of transportation, receipt, storage, identification, handling, and return/ disposal/ disposition.

Procedure:

1. Fill out the Investigational Drug Request Form.

2. Obtain shipment authorization from William Y. Ellis, Chief, Chemical Handling and Data Analysis Branch, Division Experimental Therapeutics, WRAIR.

3. Obtain Importation Permit from the French Government.

4. Request and obtain letter from the Pasteur Institute to the Chief of Sanitary Surveillance at Paris and Tunisia International Airport, to expedite liberation of the drug by French or Tunisian customs. Packages should be identified as: " WITHOUT COMMERCIAL VALUE AND FOR CLINICAL RESEARCH ONLY".

4. Use the Drug-Chain Custody Form to sign-out and sign-in the drug. Drug-Chain Custody Form subtitles:

A. Drug Stored At:
a. Organization, location, condition, temperature.
b. Drug received from: signature, signed out by, signature.
c. Time drug signed out
d. Amount of drug signed out for
e. Transported by, signature
f. Transportation conditions, method, temperature, total number of hrs to destination, immediate destination, final destination

B. Drug Received By:
a. Signature
b. Drug received from, signature
c. Amount of drug received
d. Time of drug received
e. Drug store at: receiving organization, location, condition , temperature
f. Final destination

5. During the flight, the drug will be carried in the passengers compartment to prevent high temperatures.

6. At arrival in Paris and Tunis immediately secure the drug at 4°C in the refrigerator, assigned for this purpose, at the Pasteur Institute Medical Center in Paris and at the Unit of Epidemiology at the Pasteur Institute in Tunis.

7. The respective rooms will be locked when not in use.

8. At the Pasteur Institute in Paris, the refrigerator in the Clinical Research Center serves as a backup. In case of total power failure, keep door close; the clinic generator should be activated within 30 sec. In case that the generator is not operational, immediately call Dr. Pierre Buffet at 01-40613817 or 01-43610684 (he will gladly assure you a space at the Necker Hospital). At the Pasteur Institute in Tunis, the refrigerator at the

Immunology laboratory serves as the backup. In case of total power failure, keep door close; the laboratory generator should be activated within 30 sec. In case that the generator is not operational, immediately call Dr. Afif Ben Salah at 71792429 or 71865121 (he will gladly assure you a space at the LEEP). In case of a city blackout, keep refrigerator door close, buy dry ice, and rapidly drop 2-3 blocks inside the refrigerator.

9. Please use the Drug Application Record to document the handling of WR279396. It is required that at the time the drug is removed from the refrigerator the following questions (i.e., boxes in the Drug Application Record) are answer: date, time, jar number, volume used, and initials of the person applying the topical. The Drug Application Record has 2 columns with identical questions; Column No. 1 refers to the morning application, column No. 2 refers to the afternoon application.

10. Only the Investigators, or a person designated by the investigator will be allowed to remove the study drug.

11. WR279396 jars when in use will be kept inside a plastic bag. The bag will contain 1 WR279396 jar, a 1cc syringe to apply the topical drug, and the loading 3 cc syringe. The bag will be transparent, made out of plastic, and identify clearly on both sides with the number corresponding to the study Subject No. (i.e., volunteer No. 1, will be Subject No. 1, and will be treated with jar No. 1).

12. Identify syringes in use, with the study Subject No., and the volunteer's initials.

13. Return all unused supplies of drug to the sponsor. All (n=100) jars (empty or full) will be kept secured in the refrigerator as per No. 6.

14. At the end of the study all 100 jars will be returned to William Y. Ellis, Chief, Chemical Handling and Data Analysis Branch, Division Experimental Therapeutics, WRAIR for disposition.

15. To sign-out the 100 jars use the Drug-Chain Custody Form.

Use: This SOP should be utilized by any study personnel involved in transporting, receiving, storage, handling, and returning drug to the sponsor.

Note:
a. Drug administration requires daily investigator's signature.
b. Drug should be administer to subjects under the investigator's personal supervision or under the supervision of a subinvestigator responsible to the investigators.

C0-Principal Investigators' signatures:

________________________ Date: ________________
Dr. Max Grogl

________________________ Date: ________________
Dr. Pierre Buffet

________________________ Date: ________________
Dr. Afif Ben Salah

Users: I have read & understood SOP No.2 (Signature & Date):

TOPICAL TREATMENT OF CUTANEOUS LEISHMANIASIS WITH WR 279396: A PHASE 2 STUDY IN THE OLD WORLD (Log. No.)

SOP #3: SOP For Labeling WR279396 containers.

Date Approved: ____________________ Version #: ______
Next Review Due: ____________________

Author(s): ____

____

Reviewed by: ____

____

Purpose: The purpose of this SOP is to establish a procedure for the labeling of WR279396 jars.

Procedure:

1.The immediate package of an investigational new drug intended for human use shall bear a label with the statement "Caution: New Drug--Limited by Federal (or United States) law to investigational use."

2. The label or labeling of an investigational new drug shall not bear any statement that is false or misleading in any particular and shall not represent that the investigational new drug is safe or effective for the purposes for which it is being investigated.

3. Label information:

a. WRAIR's compound number - WR279396
b. WRAIR's bottle No. (for example) - BN85873
c. Jar (container) #
d. WRAIR's Protocol No.

e. Other Information:
Apply twice daily
Topical use only
f. Caution: New Drug - Limited by Federal law to investigational use.

4. The standard label from the AFSSAPS will be added to the jar in Paris as requested by the French law.

Use: This SOP should be utilized by any study personnel involved in labeling the jars to hold the topical drug.

Co-Principal Investigators' signatures:

____________________ Date: ________________
Dr. Max Grogl

____________________ Date: ________________
Dr. Pierre Buffet

____________________ Date: ________________
Dr. Afif Ben Salah

Users: I have read & understood SOP No.3 (Signature & Date):

TOPICAL TREATMENT OF CUTANEOUS LEISHMANIASIS WITH WR 279396: A PHASE 2 STUDY IN THE OLD WORLD (Log. No.)

SOP #4: SOP For Obtaining Valid Informed Consent

Date Approved: ____________________ Version #: ______
Next Review Due: ____________________

Author(s): ____

____

Reviewed by: ____

____

Purpose: The purpose of this SOP is to establish a procedure for obtaining valid informed consent.

Procedure:

1. The consent document itself is a written summary of the information that should be provided to the subject.

2. To be effective, provide ample opportunity for you (the Investigator) and the subject to exchange information and ask questions.

3. Use the French or the Arabic Explanation of the Consent Form as a guide for the verbal explanation of the study and informed consent process.

4. Please emphasize that: a. refusal to participate in the study, will result in NO penalty or LOSS of benefits to which the subject is otherwise entitled, and b. the subject (volunteer) may at anytime during the course of the study revoke his consent, and withdraw from the study, WITHOUT penalty or LOSS of benefits.

5. Be sure that the subject has a clear understanding of the "Volunteer Registry Data Base Requirements" and the "Medical Care for Research Related Injury" policy.

6. Use at all time terms in accordance to the subject's schooling level. CFR 50.20, requires that "the information that is given to the subject shall be in language understandable to him."

7. Be sure that the subject has comprehended the information.

8. Respond to all subject's questions.

9. Provide the subject with adequate opportunity to consider all options.

10. It is your obligation, after obtaining the subject's voluntary agreement to continue providing information as the subject or situation requires.

11. Give a copy of the French or Arabic Informed Consent Form to the volunteer to provide a continuing reference for contacts.

12. Attach signed & initialized Informed Consent Form, French or Arabic Explanation of the Consent Form, and French Volunteer Donation Form to the source document folder (BROWN FOLDER).

C0-Principal Investigators' signatures:

______________________ Date: ______________

Dr. Max Grogl

______________________ Date: ______________

Dr. Pierre Buffet

______________________ Date: ______________

Dr. Afif Ben Salah

Users: I have read & understood SOP No.4 (Signature & Date):

TOPICAL TREATMENT OF CUTANEOUS LEISHMANIASIS WITH WR 279396: A PHASE 2 STUDY (Log. No. A-8225)

SOP #5: SOP For Handling and Transporting Blood and Urine Specimens

Date Approved: ____________________ Version #: ______
Next Review Due: ____________________

Author(s): ____

____

Reviewed by: ____

____

Purpose: The purpose of this SOP is to establish a procedure for specimen handling and transporting samples: specimen collection, identification, storage, analysis / evaluation, shipment, and disposition.

Procedure:

1. This is the responsibility of the Clinical Laboratory Director and persons drawing the blood and urine attached to the Clinical Laboratory.

2. All blood tubes and urine containers should be properly labeled. The following labels are stored in the office cabinet of the Clinical Lab for tagging purposes:

a. Hematology/Purple Top 1 - Day -1
b. Biochemistry/Red Top - Day -1, Day 10 & Day 20
c. Urinalysis (50-100 ml urine sample) - Day -1 & Day 20
d. Blood-kinetics - Day -1, Day 10, Day 20
e. Urine-kinetics (50-100 ml clean catch) - Day -1, Day 10 & Day 20

There are labels for all the 1.5 ml cryobank storage tubes; for all the Purple/Red Top tubes, and the plastic containers to collect the clean catch.

3. Protocol page 22 (Procedural Timetable) and page 89 (Entrance Exam & Laboratory Test Check List) will tell you when, how and volume of blood and urine to collect (In addition, see Table Below): Specimens.

**DAY**	**TOTAL No.**	**METHOD**	**PURPOSE**
**-1**	2	5ml Purple Top	a.1 tube for **Hematology** &<br>Samples: n=3 1ml sample<br>b. 1 tube for **Blood Kinetic.**<br>Samples: n=3 1ml sample
	1	10ml Red Top	**Biochemistry**
	1	50-100ml urine sample<br>(clean catch)	**Urinalysis**: 30 mls<br>**Urine kinetic**: n=8 1ml<br>Sample
**10**	2 (AM)	5ml Purple Top<br>**application)**	a. 1 tube for **Fr/Tu's** **(1hr**<br>Samples: n=3 1ml sample<br>b. 1 tube **Blood-kinetic.**<br>Samples: n=3 1ml sample
	2 (PM)	5ml Purple Top<br>**(Prior to next application)**	"
	1	10ml Red Top	**Serum creatinine**
	1	50-100 ml urine sample<br>(clean catch) - **24 hr**	**Urine Kinetic**: n=8 1ml<br>Sample
**20**	2 (AM)	5ml Purple Top	a. 1 tube for **Fr/Tu's**<br>b. 1 tube for **Blood Kinetic.**<br>Samples: n=3 1ml sample

2 (PM) **(Prior to next application)**	5ml Purple Top	"
1	10ml Red Top	**Serum creatinine**
1	50-100ml urine sample (clean catch) **- 24 hr**	**Urinalysis**: 30 mls **Urine kinetic**: n=8 1ml Sample

---

IMPORTANT:
1. On days 10 and 20, blood will be obtained for both 1 hr after drug application ("peak") and just prior to the next application of drug ("trough").
2. A 24 hr urine collection will be performed on days 10 and 20 to determine total absorbed drug and compared to day -1 baseline.

4. Samples will be storage as per protocol using the Pharmacokinetics Samples Log List Report Form (protocol page 128) in liquid nitrogen. Use the small labels for the 1.5 ml cryobank storage tubes. Secure and protect labels from liquid nitrogen with tape. In addition, use a SANFORD Sharpie permanent marker to identity tube with: SUBJECT No., DAY, and PEAK or TROUGH level (i.e. SNo.1/D10/PEAK). If for some reason the cryobank is not operational please get in touch with Dr. Pierre Buffet at 40613817 or 43074777, he has a REVCO (-70°C) that you can use. During the weekends if you can not get in touch with Dr. Pierre Buffet, please use the freezer (-20°C)/ upper part of the refrigerator that is located in the office at the Clinical Laboratory.

5. Specimens will be carried by study personnel returning to the U.S. LTC Grogl will supply you with WRAIR's CDC permit to transport specimens. DO NOT release/transport the samples without an inventory. You can use the Drug-Chain Custody Form as an example. Please list samples by: Subject Number, volunteer's initials, day, "Peak" o "Trough",

date collected, total number of vials, and volume per vial. The person receiving the specimens should signed-out the samples; keep copy of the inventory in the Specimens Inventory folder located in the office cabinet. Plasma and urine vials will be transported frozen, using Re-Freez-R-Brix foam bags, from Polar Tech Industries. Foam bags are stored in the upper part of the Office refrigerator at -20°C. During the flight, specimens will be transported in the passenger compartment. At arrival in Washington please deliver the samples to COL Jonathan Berman, Division of Experimental Therapeutics, Building 500, Silver Spring, MD (W- 319-9561). He will forward the specimens frozen to the analytic site.

6. The French and Tunisian samples are the responsibility of the Pasteur Institute in Paris and Tunis respectively.

C0-Principal Investigators' signatures:

____________________ Date: ________________
Dr. Max Grogl

____________________ Date: ________________
Dr. Pierre Buffet

____________________ Date: ________________
Dr. Afif Ben Salah

Users: I have read & understood SOP No.5 (Signature & Date):

________________ ________________ ____________

________________ ________________ ____________

TOPICAL TREATMENT OF CUTANEOUS LEISHMANIASIS WITH WR 279396: A PHASE 2 STUDY IN THE OLD WORLD (Log. No. A-97-68)

SOP #6: SOP For Data Management.

Date Approved: ____________________ Version #: ______

Next Review Due: ____________________

Author(s): ______________________________

____

Reviewed by: ______________________________

____

Purpose: The purpose of this SOP is to establish a procedure for data management: data handling, storage, and retrieval.

Procedure:

A. Data forms/sheets/records.

1. This is the overall responsibility of the Co-PIs and study coordinator.

2. The Co-PIs will be assisted by other team members especially but not limited to the data entry personnel.

3. All data will be entered in ball-point pen (preference in black) onto the data forms by the respective members of the research team.

4. All data should be entered clearly and legibly.

5. Do not leave any sections blank. The following abbreviations should be used to explain missing values: **NA - not applicable**
ND - not done
NK - not known

All dates should be entered as **DD/MM/YY**
If only part of a date is known, please enter NK for unknown information (i.e. NK/NK/84).

6. **If an incorrect entry/error is made, please cross the error with a SINGLE line. The correct entry should be written beside the original entry. Please initial and date all corrections.**

7. When entering times, please use the **24 hour clock.**

Note: Reminder; See Protocol Page 74.v

B. The checking of data forms/sheets/records.

1. To ensure drug compliance, and daily drug administration:

a. the Drug Administration Box will be signed daily by 1 of the Investigators;
b. the Drug Application Record form will be initialized by the applying Health provider;
c. on CRF Page 64 (last page) , study personnel will initial each task done each day; an Investigator and the Study Coordinator will revise the schedule each day.

2. The Medical history will be signed by the Primary Medical Officer. Dr. Buffet is responsible to obtain and attach a photocopy of the Patient's Fiche to the GREEN FOLDER. Please do not forget to initial page 64.

3. The Dermatology Examination (Day -1), and all Hearing Tests (Day 10 and Day 20), will be signed by Dr. Buffet in Paris and Dr. Ben Alaya Nissaf in Tunisia. Please do not forget to initial CRF page 64 and page 16 (Entrance Exam & Laboratory Test Check List) as soon as you finish performing the exam/test.

4. The Parasitology Laboratory Patient Data Report (PLPDR) will be reviewed by the responsible subinvestigator (Dr. Anne-Sophie Leguem in Paris and Mr Amor Zaatour in Tunisia) on a weekly basis. When a culture is found positive, the subinvestigator will immediately initial the PLPDR. The point of contact (POC) for Iso-Enzyme results is Nathalie Jolly. She will communicate directly with Dr. Francine Pratlong responsible for running the Iso-Enzyme test. Nathalie Jolly will be responsible to inform Dr. Buffet or Dr. Afif Ben Salah if the species is L. aethiopica. Dr. Buffet or Dr. Afif Ben Salah will be responsible to sign the bottom of the Parasitology Laboratory Patient Data Report and to referred the volunteer to his primary health care physician. Please do not forget to initial CRF page 64 and page 16 (Entrance Exam & Laboratory Test Check List) on time.

5. It is the final responsibility of the Investigators to ensure that all inclusion criteria are met. For this reason, only the Investigators are authorized to sign the Inclusion/Exclusion Checklist (CRF page 19). Before signing the form, please answer the check list on CRF page 17 and page 18.

6. It is the responsibility of the Clinical Laboratory Director and Study Monitor to initial, when completed, the hematology and biochemistry tests, on CFR page 16. In addition, it is their responsibility to initial CRF page 64 on time, and completes the Hematology, Biochemistry & Urinalysis Patient Data Form (CRF page 53).
It is also, their responsibility to attach to page 53 the original laboratory printouts; and to attach to the GREEN FOLDER, copy of the Clinical Laboratory test results, given to the volunteers.
At the end of the 20 days treatment period, the Study Coordinator will remove the original laboratory printouts and consolidate source documents in the DARK BROWN FOLDER.

7. It is the final responsibility of Nathalie Jolly in Paris, and Dr. Ben Alaya Nissaf to log-in the photographs taken, in the Laboratory Photo Log Book. They will also be responsible to develop/print, and organize all photos, in the WR 279396 Photograph Lesion patient Data Report (Protocol pages: 129 -131). Please do not forget to initial CRF page 64 and page 16 (Entrance Exam &

Laboratory Test Check List) on time. There will be weekly informal checks of the WR 279396 Photograph Lesion patient Data Report by the Study Coordinator. Each photograph should be identify with the Subject No. and date taken. Use a plastic ruler in mm as a scale beside the lesion.

8. Concomitant medications will be listed by the Primary Medical Officer using the Concomitant Medication Form (CRF page 58). An Investigator or the Study Coordinator (by the request of an investigator) will revise the form weekly. This can be done very easily by reviewing
Question No. 2 of the volunteer's Daily Visit Form (days 1-20) i.e. "Has the volunteer taken any medication apart from the study medication since the last visit?"; YES or NO. A positive answer (YES) should correlate to an entry in the Concomitant Medication Form.

9. The Adverse Event form (Appendix 3) will be filled out by the MD who saw the patient in conjunction with the PI. This report must be sent within 72 hours of the event. For a detail Safety, and Tolerance IND Safety Reporting please consult the Adverse Event Reporting flow chart for WR279396 also in Appendix 3. The Adverse Experience form (CRF page 59 or copies) will help you organize your ideas and facts for communicating the Adverse Event and writing the report.

10. Dr. Buffet in Paris and Dr. Afif Ben Salah in Tunisia will be responsible to fill out the Adverse Experience form (CRF page 59 or copies). This is necessary every time the volunteer answer YES on Question No.1 of the volunteer's Daily Visit Form (days 1-20).

11. A summary of those failing to complete the study i.e. the Study Termination Record (CRF page 63) will be filled in and signed by Dr. Buffet in Paris and Dr. Afif Ben Salah in Tunisia. This information will be sent to Dr. Max Grögl on an approximately weekly basis.

C. Storage of data forms/sheets/records.

1. Each study subject will have his own file.

2. These will be stored in the office filing cabinet at the Pasteur Institute in Paris and in Sidi Bouzid in Tunisia.

3. The file will contain completed forms.

4. The following forms will be in the subject's file:
   a. BROWN Folder: French Informed Consent Form; clinical Laboratory results (Day -1, Day 10, Day 20): i.e. original slips (printouts) from the different analyzers (equal to copy given to volunteer; Drug Application Record.
   b. Case Report Form (source document).
   c. GREEN Folder copy of the volunteer Medical Fiche

5. The File Folder and the BROWN Folder will be labeled by the Study Coordinator with:
   a. Subject's No.
   b. Subject's ID Number
   c. Subject's Initials

D. Record Books/ Slide Box(S)

1. The photograph record book will be filled out by Nathalie Jolly in Paris and Dr. Ben Alaya in Tunis :
   a. Subject's no.
   b. Date
   c. Day of Study (Day -1, Day 20, Day 30, Day 80 and 6 months)
   d. Lesion No.
   e. Exposure No.s (From: __ To: __)
   f. Signature

2. The Photograph Record Book will be stored in the filing cabinet.

3. The Slide Box(s) will be organized and stored in the filling cabinet.
4. Slides will be labeled with:
   a. Subject's no.
   b. Lesion No. (i.e., L2 = lesion 2)
   c. Date
   d. Subject's Initials

E. Data Storage and Security.

1. All data forms/sheets/records used in the study will be stored in the office filing cabinet at the Pasteur Institute in Paris and at Sidi Bouzid in Tunisia. Always, secure the cabinet, and the office front door, after working hours.

2. At the end of the study, data forms/sheets/records will be checked by the PI, investigator and subinvestigators.

3. The subject's forms/sheets/records will be kept in their respective files.

4. The subject's forms/sheets/records and all record books will be photocopied twice:

a. the originals to be sent to USAMMDA/WRAIR
b. one copy to remain at the Pasteur Institute in Paris and Tunis
c. if requested by the health authorities one copy will remain at Sidi Bouzid in Tunisia

F. Accounting for Missing and Spurious Data.

1. Routine monitoring of the study documents will occur to evaluate missing data elements and establish a standard for missing data. All attempts will be made to ensure the completeness of data and missing or spurious data will not be included in the data analysis or included in publications.

G. Results Pending.

1. The results of the following tests may not be available at the end of the study:
a. WR279396 levels- these will be done by Dr. Peter Lim, SRI International.
b. Culture
c. Iso-Enzyme profiles

C0-Principal Investigators' signatures:

_____________________________ Date: ____________________

Dr. Max Grogl

_____________________________ Date: ____________________

Dr. Pierre Buffet

_____________________________ Date: ____________________

Dr. Afif Ben Salah

Users: I have read & understood SOP No.6 (Signature & Date):

____________________ ____________________ ____________________

____________________ ____________________ ____________________

____________________ ____________________ ____________________

TOPICAL TREATMENT OF CUTANEOUS LEISHMANIASIS WITH WR 279396: A PHASE 2 STUDY IN THE OLD WORLD (Log. No.)

SOP #7: SOP For Application of Study Drug

Date Approved: ____________ Version #: ______

Next Review Due: ____________

Author(s): ____________

____

Reviewed by: ____________

____

Purpose: The purpose of this SOP is to establish a procedure for the application of WR279396 topical cream.)

Generalities:

1. Only the investigators are authorized to complete the inclusion/exclusion list and enrolled volunteers. In Tunisia, the study drug will be administered by health workers (Investigators/subinvestigators, never by the patient, and only at the request of the investigators. In Paris the study drug will be administered once a day by a health worker. The second application of the day will be self administered by the volunteer. However, in both sites only a health worker will record the application of the drug in the Drug Application Form. That means that, in Paris, the health worker will question the volunteer on how and when the drug was administered. The health provider will also inspect the jar containing the topical and the syringe to determine if the correct volume was administered by the patient. As in Tunisia, the study drug will be administered only at the request of the investigators.

2. After drug administration the health worker will sign the "Drug Administration Box" and the "Drug Administration Card". Any subject not found at the time of drug administration will be sought after. If he is found within 12 hrs, he will be given his cream. We want to give the cream twice a day for 20 days. If someone must be away, then delay the start of the medicine.

3. This is a leishmania drug study. It is very important that the correct amount and cream be given to the correct volunteer. Careful attention to detail is necessary to do this important job correctly.

4. There are 2 different drug groups (WR279396) and (placebo), but all medicine "creams" looks alike. For the purposes of the study, each volunteer is his own group. He or she can only receive medicine from the medicine with his study number on the medicine unless approved by the investigators. Approval of a different number should only be given when the number was used to pre-enrolled a volunteer ; the volunteer is not enrolled and the jar was never used. In case that more than 1 jar is necessary (multiple large lesions) the investigators will ask Washington through COL Berman for another number/jar that is identical to the jar used. Only the investigators are authorize to request an additional jar/number.

5. It is critical to check volunteer and cream/jar identification. Even if you know the volunteer well, ask for his number and name to be absolutely sure. Even if you are familiar with the drug bags, make sure you confirm that the proper numbered drug goes to the correct volunteer.

Procedure:

1. WR279396 and WR279396 placebo ("study drug/cream") will be contained in 50 ml plastic jars with screw caps. These containers will be stored refrigerated (4-6°C). The container will be pre-labelled with:

Subject's randomization number
Protocol WR
Lot #
Apply twice daily
Caution: New Drug - Limited by Federal law to investigational use
Topical use only

2. The plunger of a 3 ml plastic syringe will be removed, and study drug will be mechanically added to the empty syringe barrel using a spatule made out of wood. Pre-mixing of the medication in the 50 ml jar will be done once a week (monday morning) or when condensation (drops) are noticed on the surface cream. Wood spatulas will be used only once. The plunger will be reinserted so the cream is situated between the exit of the 3 ml syringe and the end of the plunger. The filled syringe will be stored refrigerated (4-6°C). The syringe will be labelled with the subject's randomization number and the subject's initials.

3. Each day, cream will be added to a 1ml syringe from the 3 ml loading syringe. The mechanism to achieve this is to fit a two-way stop cock to exits of both the loading syringe and the 1ml syringe, and press down on the plunger of the loading syringe. This will drive cream out the loading syringe, through the stop cock and into the 1ml syringe. The 1ml syringe will be labelled with the subject's randomization number.

4. WR279396 jars when in use will be kept inside a zip-lock bag. The bag will contain one WR279396 jar, the loading 3cc syringe, and a 1cc syringe to apply the cream. The bag will be transparent, made out of plastic, and identify clearly on both sides with the number corresponding to the study Subject's No. (i.e., volunteer No. 1, will be Subject No. 1, and will be treated with jar No. 1); the subject's ID number; and the subject's initials. Only the Investigators, or a person designated by the investigator will be allowed to remove the study drug.

5. The cream in the 1ml syringe will be used for the two applications of treatment. After the morning treatment, the 1ml syringe will be immediately returned to the bag and to the refrigerator for storage until the afternoon treatment.

6. The lesion shall be carefully cleaned with soap and water, and sterile 0.9% saline solution before application of cream. Sterile USP Type VII Gauze sponges will be used to clean the lesions after washed with soap. Never apply cream without cleaning the lesion i.e. lesions will be cleaned twice a day.

7. Dry gently the lesion before applying the study drug. Also, use a sterile gauze to dry the lesion of lymph if necessary before applying the cream.

8. The cream shall be dispensed directly onto the lesion from the 1ml syringe, then spread over the lesion with a disposable finger glove (a new one for each patient). Rub the cream in with a circular motion. Cream should penetrate under the lesion's borders.

9. The lesion will be cover with a transparent polyurethane adhesive dressing until the next application. A new dressing will be used with each application.

10. Apply cream twice-a-day for 20 days. Twice a day shall mean that the second administration occurs between 6 and 16 hrs after the first administration.

11. The amount of cream to be added per application approximates 0.0005 ml per mm2:

Lesion Area (mm2)	ml Cream per Application (ml)	<400
400-600	0.20	
600-800	0.30	
800-1000	0.40	
1000-1200	0.50	
1200-1400	0.60	
1400-1600	0.70	
1600-1800	0.80	
1800-2000	0.90	

Note : As an example a 500 mm2 lesion is 2 cm x 2.5 cm, and would require 0.2O ml of cream.

12. Documentation of application will be performed using the Case Report Form daily "Drug Administration" box, and the daily "Drug Administration Card".

13. The application of the cream shall be by health service personnel (ie, not by the patient himself except situation described in 1.), and the medical personnel shall record that the formulation was administered.

14. Observe the volunteer for 30 min after cream is administered.

15. Any alteration observed or reported by the volunteer should be annotated in the Case Report Form daily "local and systemic toxicity" boxes and communicated immediately to Dr. Buffet/Dr. Ben Salah then communicated to Dr. Grögl (this is the responsibility of ALL THE STUDY TEAM MEMBERS: KEEP ALERT, KEEP THINKING, KEEP INFORMING, KEEP COMMUNICATING).

16. The only persons with access (key) to the storage refrigerator will be the investigators, and study coordinator.

Co-Principal Investigators' signatures:

____________________ Date: ________________

Dr. Max Grögl

____________________ Date: ________________

Dr. Pierre Buffet

____________________ Date: ________________

Dr. Afif Ben Salah

I have read & understood SOP No.7 (Signature & Date):

________________ ________________ ____________

________________ ________________ ____________

________________ ________________ ____________

TOPICAL TREATMENT OF CUTANEOUS LEISHMANIASIS WITH WR 279396: A PHASE 2 STUDY IN THE OLD WORLD (Log. No.)

SOP #8: SOP For Lesion Size Measurement and Recording

Date Approved: ____________________ Version #: ______

Next Review Due: ____________________

Author(s): ____

____

Reviewed by: ____

____

Purpose: The purpose of this SOP is to establish a procedure for measuring and recording the lesion size.

Procedure:

1. Lesions will be measured in two perpendicular directions.

2. The lesion shall be measured in its greatest dimension, and at 90 degrees to the first measurement.

3. The distances between the margins of the ulcer (area of the lesion without epidermis after removing the crust) shall be measured. The distances between the borders of induration (measured using the pen Sokal's technique) will be used to determine if the ulceration represents at least 50% of the whole lesion surface.

4. Measurement shall be with the Digimatic Caliper in mm.

5. Turn ON/OFF switch to On.

6. Turn Inch/mm switch to mm.

7. Setting the Origin: After turning on the power, close the jaws and hold down the ORIGIN switch for more than one second. The "0.00" display appears, indicating Origin (zero point) setting is complete.

8. Use thumb roler to measure the lesion.

9. The mm X mm values shall be entered into the "Lesion(s)-Clinical Report Form", ie, 12.3 X 6.0 (largest value should be recorded first). One decimal shall be used. Please round-up the second decimal mathematically.

Note: IMPORTANT: Set up the origin of the caliper after installing a new battery.

Co-Principal Investigators' signatures:

__________________________ Date: __________________

Dr. Max Grogl

__________________________ Date: __________________

Dr. Pierre Buffet

__________________________ Date: __________________

Dr. Afif Ben Salah

I have read & understood SOP No.8 (Signature & Date):

TOPICAL TREATMENT OF CUTANEOUS LEISHMANIASIS WITH WR 279396: A PHASE 2 STUDY IN THE OLD WORLD (Log. No.)

SOP #9: SOP For Romberg Test

Date Approved: ____________________ Version #: ______

Next Review Due: ____________________

Author(s): ____

____

Reviewed by: ____

____

Purpose: The purpose of this SOP is to establish a procedure for the Romberg Test.

Procedure:

1. This is the responsibility of Dr. Pierre Buffet in Paris and Dr. Ben Alaya in Tunisia

2. Put subject at ease.

3. Insure the subject understands task.

4. Use these instructions:
I am going to ask you to please stand up, keep your feet close together and close your eyes. I am going to evaluate your body oscillations. The oscillations could be in any direction.

5. Conduct the test.
Without touching the subject extend your arms/hands and position them in front of you about 10 inches from the right and left shoulder of the subject (i.e., frame the subject). This will help you determine if the subject is oscillating .

6. Put the subject at ease.

7. Insure the subject understands the task.

8. Use these instructions:
I am going to repeat the exam. However, this time please position your right foot directly in front of your left foot.

9. Conduct the test.
Without touching the subject extend your arms/hands and position them in front of you about 20-30 centimeters from the right and left shoulder of the subject (i.e., frame the subject). This will help you determine if the subject is oscillating.

Co-Principal Investigators' signatures:

______________________ Date: ______________
Dr. Max Grogl

______________________ Date: ______________
Dr. Pierre Buffet

______________________ Date: ______________
Dr. Ben Salah

I have read & understood SOP No.9 (Signature & Date):

______________ ______________ ______________

______________ ______________ ______________

TOPICAL TREATMENT OF CUTANEOUS LEISHMANIASIS WITH WR 279396: A PHASE 2 STUDY IN THE OLD WORLD (Log. No.)

SOP #10: SOP For Indirect Immunofluorescence Antibodies (IFA)

Date Approved: ____________________ Version #: ______
Next Review Due: ____________________

Author(s): ____

Purpose: The purpose of this SOP is to establish a procedure for detecting Leishmania specific antibodies (titer) in sera.

Procedure:

1. Preparation of Antigen:

a. *L. panamensis*promastigotes are grown in Schneider's medium and harvested in log phase (about 300ml). The parasites are washed 3 times for 5 min in 0.15M PBS pH7.2 by centrifugation at 2,000 rpm for 10 min.

b. Promastigotes are fixed with 1% formaldehyde in PBS for 1hr and washed by centrifugation 3-5 times in PBS as in a.

c. The pellet after the last wash is resuspended in 5 ml of PBS.

d. 10ul of the fixed antigen is placed in 2-3 wells in a immunofluorescence slide.

e. The optimal number of parasites per field is determined using the same microscope and objective as the one to be utilized for the IFA test. Standardize the antigen by selecting the dilution that gives you the maximum number of parasites per field with space between the promastigotes.

f. If the antigen is too concentrated please dilute again accordingly and repeat e.

g. Applied 10 ul per well of the optimum antigen dilution to the IFA glass slides (10ul/well - 8wells/slide = 80 ul of antigen per slide).

h. The slides are air dried overnight at room temperature or for 1hr inside the hood; individually wrapped; and stored inside a Zip-bag (tightly sealed) at -20oC for several months.

i. Before use in the IFA, the slides are allowed to reach room

temperature.

2. Controls:

a. Positive Control: Use a positive human control serum with a high titer (Titer: 1:512)

b. Negative Control: Use a U.S. servicemen (Titer: Negative or 1:2).

c. Conjugate Control: PBS

Note: The controls should be run every time the IFA test is done. If the titer are different to the titer above you have a problem. Please repeat the assay.

3. IFA Test:

a. In a 96 wells microtiter plate, please make two fold serial dilutions in PBS, pH7.2 starting at (1:2, 1:4, 1:8, 1:16, 1:32, 1:64, 1:128, 1:256). Be sure to identify clearly the samples by writing in the border of the plate (C+, C-, PBS for the controls, and the Subject's No. for the test samples).

b. Use 1 IFA slide per volunteer/control sample (again be sure to identify the slide with the volunteer's study number and C+, C-, PBS). Use 20 ul/well of the test dilutions to cover the entire well. Remember to start with 1:256 (more diluted) and move your way up to 1:2.

c. Place the slides in a moist chamber with cover. Let the antibodies if present bind to the antigen for 45 min at room temperature.

d. Unbound antibody is washed off by 3 immersions in fresh PBS of 5 minutes each.

e. Remove the slides and drain the excess buffer onto a piece of absorbent paper. Wipe the back of the slides. Before adding the conjugate, the edges of the slides are carefully dried up to prevent overflow of the conjugate, being careful not to dry the spots/wells containing the antigen.

f. Add 20ul per well of the conjugate (FITC labeled affinity purified -goat-anti-human IgG) (Catolog No. 02-10-02 Kirkegaard & Perry Laboratories, Gaithersburg, MD) at an optimal dilution of 1:20 in 0.01% Evan's blue PBS.

Note: Remember to add PBS to the conjugate (second antibody control) NOT CONJUGATE.

g. The slides are incubated for 45 min at room temperature within a moist chamber .

h. Unbound antibody is washed off by 3 immersions in fresh PBS of 5 minutes each.

i. Remove the slides and drain the excess buffer onto a piece of absorbent paper. Wipe the back of the slides. Mount slides with 2 or 3 drops of buffered glycerine and a cover slide, and analyzed by fluorescence microscopy.

j. Examine first the control slides; check the titer. The PBS second antibody control should be negative.

k. A good antigen should give you a clear break between positive and negative; for example:

1:2	1:4	1:8	1:16	1:32	1:64	1:128	1:256
+	+	+	+	+/-	-	-	-

The titer is 1:32

Note: A deviation greater than +/- 1 titer is NOT acceptable.

Co-Principal Investigators' signatures:

________________________ Date: ________________

Dr. Max Grogl

________________________ Date: ________________

Dr. Pierre Buffet

I have read & understood SOP No.10 (Signature & Date):

________________ ________________ ____________

________________ ________________ ____________

TOPICAL TREATMENT OF CUTANEOUS LEISHMANIASIS WITH WR 279396: A PHASE 2 STUDY IN THE OLD WORLD (Log. No.)

SOP #11: SOP For Rapid Detection of Leishmania Amastigotes in Human Tissues (DIFMA)

Date Approved: ____________________ Version #: ______
Next Review Due: ____________________

Author(s): ____

Purpose: The purpose of this SOP is to establish a procedure for detecting Leishmania amastigotes in tissue biopsies.

Procedure:

1. Use 4mm punch biopsies and/or needle aspirates from the margin of the lesion, avoid the open area of ulceration.

2. Make impression smears (n=4) of the biopsy on glass slides and allow to air dry. Identify the glass slides with the volunteer's study No., initials and date. Impressions are made by securing the tissue with a tweezers and pressing the tissue firmly against the glass (center of the slide) with a circular motion.

3. In case of a needle aspirate, concentrate the fluid by centrifugation for 5 min at 800xg using a cytospin 2 centrifuge (Shadon Southern Instruments, Inc.) and allow to air dry.

4. When performing a test please use a positive and negative control slide. Positive and negative control slides are kept at -20°C.

5. Flood control and test slides with 25-30 ul of the Leishmania genus specific monoclonal antibody.

6. Incubate for 45 min at room temperature.

7. Eliminate unreactive antibodies by thorough washing in PBS, pH7.2 (3 times 5 min per wash); use fresh PBS in each wash.

8. Add 25-30ul of an affinity-purified F(ab')2 fragment of goat anti-mouse IgG + IgM serum labeled with fluorescein isothiocyanate (FITC); Jackson Immuno-Research Laboratories, Avondale, PA. Autofluorescence is kept at a minimum by inclusion of a 1:1,000 dilution of Pentachrome violet.

9. Incubate for 45 min at room temperature.

10. Remove unbound conjugate as in 6.

11. Mount slides with coverslip in buffered glycerol.

12. Examine at least 100 fields (X43) before finalizing the report as negative.

Note: An amastigote will be apple green (look for the characteristic oval/round shape and size); bright plasma membrane with green cytoplasm and less stained "vacuole" = nucleus.

Co-Principal Investigators' signatures:

________________________ Date: ________________

Dr. Max Grogl

________________________ Date: ________________

Dr. Pierre Buffet

I have read & understood SOP No.10 (Signature & Date):

________________ ________________ ____________

________________ ________________ ____________

TOPICAL TREATMENT OF CUTANEOUS LEISHMANIASIS WITH WR 279396: A PHASE 2 STUDY IN THE OLD WORLD (Log. No.)

SOP #12: SOP For Threshold Audiogram

Date Approved: ____________________ Version #: ______
Next Review Due: ____________________

Author(s): ______________________________________

Purpose: The purpose of this SOP is to establish a procedure for how to conduct a threshold audiogram.

Procedure:

1. Step 1 - Preparing Subject for test:
    a. Put subject at ease.
    b. Insure that subject understands task.
    c. Use these instructions:
        "I am going to place these earphones over your ears. You will hear a variety of tones - some high, some low, some loud, some very soft. Whenever you hear, or think you hear one of those sounds, raise your hand. Lower your hand when you no longer hear the sound. Remember that though some of the tones will be easy to hear, others will be very faint. Therefore, you should listen very carefully and raise your hand whenever you think you hear the tone."

2. Step 2 - Preparing Subject for Test
    a. Eliminate all obstruction between earphones and subject.
    b. Place headband solidly on crown of subject's head.
    c. Center earphones carefully over both ears.

3. Step 3 - Conducting the Threshold Test

A. Familiarize subject with test and determine starting point:

a. Start with "better" or RIGHT ear.
b. Demonstrate tone for subject using 1000 Hz at 40 dB HL.
c. Set HL control to -10 dB.
d. Hold present bar down and gradually increase intensity until a response occurs. Switch tone off and present again in two seconds. If subject responds again, this is "start" point. If subject does not respond again, repeat the step.

B. Determine Threshold:

e. Present tone 10 dB below "start" point.
f. Present tone for one to two seconds. Time between tones can vary, but should not be shorter than test tone.
g. With each response, decrease 10 dB more for next presentation.
h. After each failure to respond, increase signal 5 dB until first response occurs.
i. Continue with DOWN 10 dB, UP 5 dB until threshold is reached. Threshold=minimum dial setting at which a response has occurred two times on ascending scale.
j. Record threshold on audiogram.

Note: Repeat from step five for each tone setting in the following order: 1,000/2,000/3,000/4,000/6,000/8,000/1,000/500/250 Hz.

If there is a different of 20 dB or more between two successive octaves, test the inter-octave frequencies:750, 1,500, 3,000 Hz.

Co-Principal Investigators' signatures:

____________________ Date: ________________

Dr. Max Grogl

____________________ Date: ________________

Dr. Pierre Buffet

Page 3 SOP #12: Audiogram.

I have read & understood SOP No.10 (Signature & Date):

______________________ ______________________ ______________

______________________ ______________________ ______________
